# Identification of a Novel Tumor Microenvironment–Associated Eight-Gene Signature for Prognosis Prediction in Lung Adenocarcinoma

**DOI:** 10.3389/fmolb.2020.571641

**Published:** 2020-09-23

**Authors:** Chao Ma, Huan Luo, Jing Cao, Xiangyu Zheng, Jinjun Zhang, Yanmin Zhang, Zongqiang Fu

**Affiliations:** ^1^Charité – Universitätsmedizin Berlin, Corporate Member of Freie Universität Berlin, Humboldt-Universität zu Berlin, and Berlin Institute of Health, Berlin, Germany; ^2^Berlin Institute of Health Center for Regenerative Therapies and Berlin-Brandenburg Center for Regenerative Therapies (BCRT), Charité – Universitätsmedizin Berlin, Berlin, Germany; ^3^Klinik für Augenheilkunde, Charité – Universitätsmedizin Berlin, Corporate Member of Freie Universität Berlin, Humboldt-Universität zu Berlin, and Berlin Institute of Health, Berlin, Germany; ^4^Department of Human Anatomy, College of Basic Medical Sciences, Zhengzhou University, Zhengzhou, China; ^5^Department of Laboratory Medicine, the Second Affiliated Hospital of Henan University of Chinese Medicine, Zhengzhou, China

**Keywords:** lung adenocarcinoma, tumor microenvironment, tumor immunity, gene signature, risk score, prognosis, biomarkers

## Abstract

**Background:**

Lung cancer has become the most common cancer type and caused the most cancer deaths. Lung adenocarcinoma (LUAD) is one of the major types of lung cancer. Accumulating evidence suggests the tumor microenvironment is correlated with the tumor progress and the patient’s outcome. This study aimed to establish a gene signature based on tumor microenvironment that can predict patients’ outcomes for LUAD.

**Methods:**

Dataset TCGA-LUAD, downloaded from the TCGA portal, were taken as training cohort, and dataset GSE72094, obtained from the GEO database, was set as validation cohort. In the training cohort, ESTIMATE algorithm was applied to find intersection differentially expressed genes (DEGs) among tumor microenvironment. Kaplan–Meier analysis and univariate Cox regression model were performed on intersection DEGs to preliminarily screen prognostic genes. Besides, the LASSO Cox regression model was implemented to build a multi-gene signature, which was then validated in the validation cohorts through Kaplan–Meier, Cox, and receiver operating characteristic curve (ROC) analyses. In addition, the correlation between tumor mutational burden (TMB) and risk score was evaluated by Spearman test. GSEA and immune infiltrating analyses were conducted for understanding function annotation and the role of the signature in the tumor microenvironment.

**Results:**

An eight-gene signature was built, and it was examined by Kaplan–Meier analysis, revealing that a significant overall survival difference was seen. The eight-gene signature was further proven to be independent of other clinico-pathologic parameters via the Cox regression analyses. Moreover, the ROC analysis demonstrated that this signature owned a better predictive power of LUAD prognosis. The eight-gene signature was correlated with TMB. Furthermore, GSEA and immune infiltrating analyses showed that the exact pathways related to the characteristics of eight-genes signature, and identified the vital roles of Mast cells resting and B cells naive in the prognosis of the eight-gene signature.

**Conclusion:**

Identifying the eight-gene signature (INSL4, SCN7A, STAP1, P2RX1, IKZF3, MS4A1, KLRB1, and ACSM5) could accurately identify patients’ prognosis and had close interactions with Mast cells resting and B cells naive, which may provide insight into personalized prognosis prediction and new therapies for LUAD patients.

## Introduction

Lung cancer ranks among the top cancer-related deaths worldwide (Bray et al., 2018). Histologically, 15% of patients are classified as small cell lung cancer (SCLC), whereas the other 85% of patients are classified as non-small-cell lung cancer (NSCLC) (Gridelli et al., 2015). In NSCLC, lung adenocarcinoma (LUAD) is the most common subtype (Cancer Genome Atlas Research and Network, 2014). Over the past few decades, extensive genomic studies have identified several high-frequency genetic changes in LUAD, such as EGFR, KRAS mutations, and ALK rearrangements, which may be related to the occurrence and development of LUAD and have contributed to EGFR targeting drug development (Herbst et al., 2018). However, a large number of patients with advanced LUAD have no targeted mutations. For these patients, studies on immune checkpoints like programmed death 1 (PD-1) and cytotoxic T lymphocyte–associated antigen-4 (CTLA-4) have demonstrated the effectiveness and safety of established treatments (Hellmann et al., 2017; Xu et al., 2018), which highlights the importance of tumor microenvironment on the clinical outcomes of LUAD patients.

More and more studies have shown that the occurrence and development of tumors are the results of the dynamic interaction between tumor cells and various components (including immune cells and stromal cells) within the tumor microenvironment (Hui and Chen, 2015; Verrecchia and Redini, 2018; Shi et al., 2020). Therefore, the tumor microenvironment may become a promising target for cancer treatment (Alsaab et al., 2017). In Yoshihara et al. (2013) designed an algorithm called ESTIMATE (Estimation of STromal and Immune cells in MAlignant Tumor tissues using Expression data). The algorithm analyses specific gene expression characteristics of immune and stromal cells and calculates immune and stromal scores to predict non-tumor cell infiltration (Yoshihara et al., 2013). Considering that previous research has mainly focused on one or two types of immune cells and key genes, it may create a bias against the LUAD microenvironment. Finding multiple genes from the tumor microenvironment to build a gene signature can obtain a better accuracy of the prognostic potential (Liu et al., 2019a,b; Zhou et al., 2019; Bi et al., 2020; Guo et al., 2020).

Here, we conduct comprehensive mining of The Cancer Genome Atlas (TCGA) and Gene Expression Omnibus (GEO) databases to determine the minimum number of potentially robust genes from tumor microenvironment that can be used to predict the prognosis of LUAD patients. Importantly, we used the LASSO algorithm, which can effectively analyze high-dimensional sequencing data (Tibshirani, 1997). Besides, we assessed the accuracy of this eight-gene signature and validated it by comparing with tumor mutational burden (TMB) and testing in a validation cohort. Moreover, GSEA and immune infiltrating analyses were conducted to explore the role of the signature in the tumor microenvironment.

## Materials and Methods

### Data Mining From TCGA and GEO

The gene expression profiles of LUAD from 515 patients, along with their clinical and survival data, were downloaded from TCGA Xena Hub^1^ with cohort name: TCGA-LUAD. Besides, we selected dataset GSE72094 from the GEO database^2^, which contains 442 LUAD cases, for the study. In our research, TCGA-LUAD was used as the training cohort, whereas GSE72094 was taken as the validation cohort.

### Immune and Stromal Scores

Immune scores and stromal scores of each case of the training cohort calculated by the ESTIMATE algorithm were downloaded from the MD Anderson Cancer Center ESTIMATE Results download page^3^ (Supplementary Table S1; Yoshihara et al., 2013).

### Identification of the Intersection Differentially Expressed Genes (DEGs) Among Immune and Stromal Scores

All cases in the training cohort were divided into groups of high and low scores according to the median. DEGs were identified between high and low immune/stromal score groups using “limma” R package (Ritchie et al., 2015), with a cutoff of | log2(fold-change)| > 1 and false discovery rate (FDR) < 0.05. “pheatmap” R package was applied to produce heatmaps and clustering of DEGs. Genes that were upregulated in both high immune and stromal scores groups were defined as intersection-upregulated DEGs. Genes that were downregulated in both high immune and stromal scores groups were taken as intersection-downregulated DEGs. A combination of these two intersection DEGs was the intersection DEGs.

### Identification and Validation of the Prognostic Gene Signature

To continue, Kaplan–Meier analysis was performed in the training cohort, to screen for potential prognostic genes from the intersection DEGs identified in the previous step based on overall survival. Only genes with *p* values < 0.05 in the log-rank test were considered as significant to pass Kaplan–Meier analysis screening. Also, univariate Cox regression analysis was performed on the training cohort to look for prognostic genes from the intersection DEGs with *p* values < 0.05. Same as before, only genes that showed significance in the overall survival analysis were considered to pass univariate Cox regression analysis screening.

The genes that passed both Kaplan–Meier and univariate Cox analyses were then entered into the LASSO Cox regression model analysis, which was implemented in the training cohort utilizing R software and the “glmnet” package. Ten-times cross-validations were applied to detect the best penalty parameter lambda (Tibshirani, 1997; Sauerbrei et al., 2007; Friedman et al., 2010; Goeman, 2010). According to the best lambda value, a list of prognostic genes with coefficients was obtained from the gene expression and patients’ survival data. Moreover, the risk score of each patient could be calculated based on the expression level of each prognostic gene and its corresponding coefficient.

Using the median risk score as the cutoff point, the patients in the training cohort were distributed to high-risk or low-risk groups, and Kaplan–Meier analysis was applied to evaluate the survival difference between the two groups. Besides, Cox and ROC analyses were conducted to further assess the prognostic value of the gene signature in the training cohort. Furthermore, the prognostic gene signature was validated in the validation cohort. The same formula was conducted to compute risk scores like that in the training cohort. Kaplan–Meier, Cox, and ROC analyses were implemented as described earlier.

### TMB Correlation With the Gene Signature Risk Score

Tumor mutational burden is defined as the total number of non-synonymous mutations per coding area of a tumor genome. Recently, a high TMB has been identified as a genetic signature that is associated with a favorable outcome for immune checkpoint inhibitor therapy (Alexandrov et al., 2013; Gibney et al., 2016; Yuan et al., 2016). The mutation data of LUAD patients were obtained from the TCGA Xena Hub mentioned earlier. The TMB score for each LUAD patient was calculated by the formula as follows (Robinson et al., 2017; Liu J. et al., 2020): TMB = (total mutation/total covered bases) × 10^6^. The Spearman rank correlation coefficient was applied to assess the correlation between TMB and risk score, further evaluating the prognostic value of the gene signature identified in this study and the possibility of immune checkpoint inhibitor therapy targeting risk score. *P* value < 0.05 was considered statistically significant.

### Gene Set Enrichment Analysis

The Hallmark (v7.1) and C7 (v7.1) gene set collections were downloaded from the Molecular Signatures Database v7.1 download page^4^. GSEA was performed based on the downloaded gene set collections using GSEA software (v4.0.3)^5^. The training cohort was taken for GSEA to reveal the functions and pathways in the DEGs between high-risk and low-risk groups. Only gene sets with |NES| > 1, NOM *p*-val < 0.05, and FDR *q*-val < 0.25 were considered significant.

### Correlation of Risk Score With the Proportion of 22 Kinds of Tumor-Infiltrating Immune Cells (TICs)

The CIBERSORT calculation method was used to estimate the 22 kinds of TICs abundance distribution of all tumor samples in the training cohort, and then quality filtering was performed. In total, 515 LUAD samples with *p* value < 0.05 were selected for the following analysis. The correlations between 22 kinds of TICs were examined by Pearson coefficient test. Spearman coefficient test was used for the correlation test between the TICs’ proportion and risk score. The Wilcoxon rank-sum test verified the differentiation of 22 kinds of immune cells between low- and high-risk groups. Besides, based on the 22 TICs’ infiltration volume and survival data of each of the 515 patients, we used the univariate Cox and Kaplan–Meier methods to screen TICs with prognostic significance. Together with the result of correlations between risk score and 22 TICs, we further assessed which kind of immune cells play a role in the prognostic zone of the gene signature identified in this study. *P* value < 0.05 was a statistically significant threshold.

### Statistical Analysis

All statistical calculations were performed in R software. Kaplan–Meier analysis was conducted to check the prognosis difference between groups, along with the *p* value, which was examined in the log-rank test. Univariate and multivariate Cox proportional hazard regression analyses were conducted to assess the association between risk score and prognosis. The ROC analysis was applied to examine the sensitivity and specificity of survival prediction using the gene signature risk score. An area under the ROC curve (AUC) served as a pointer of prognostic accuracy. The R package “pROC” was used for ROC analysis, and the “delong” method is used to study the significant differences among ROC curves. In addition to noted before, all analyses *p* value < 0.05 was a statistically significant threshold.

## Results

### Clinical Characteristics

**FIGURE 1:**
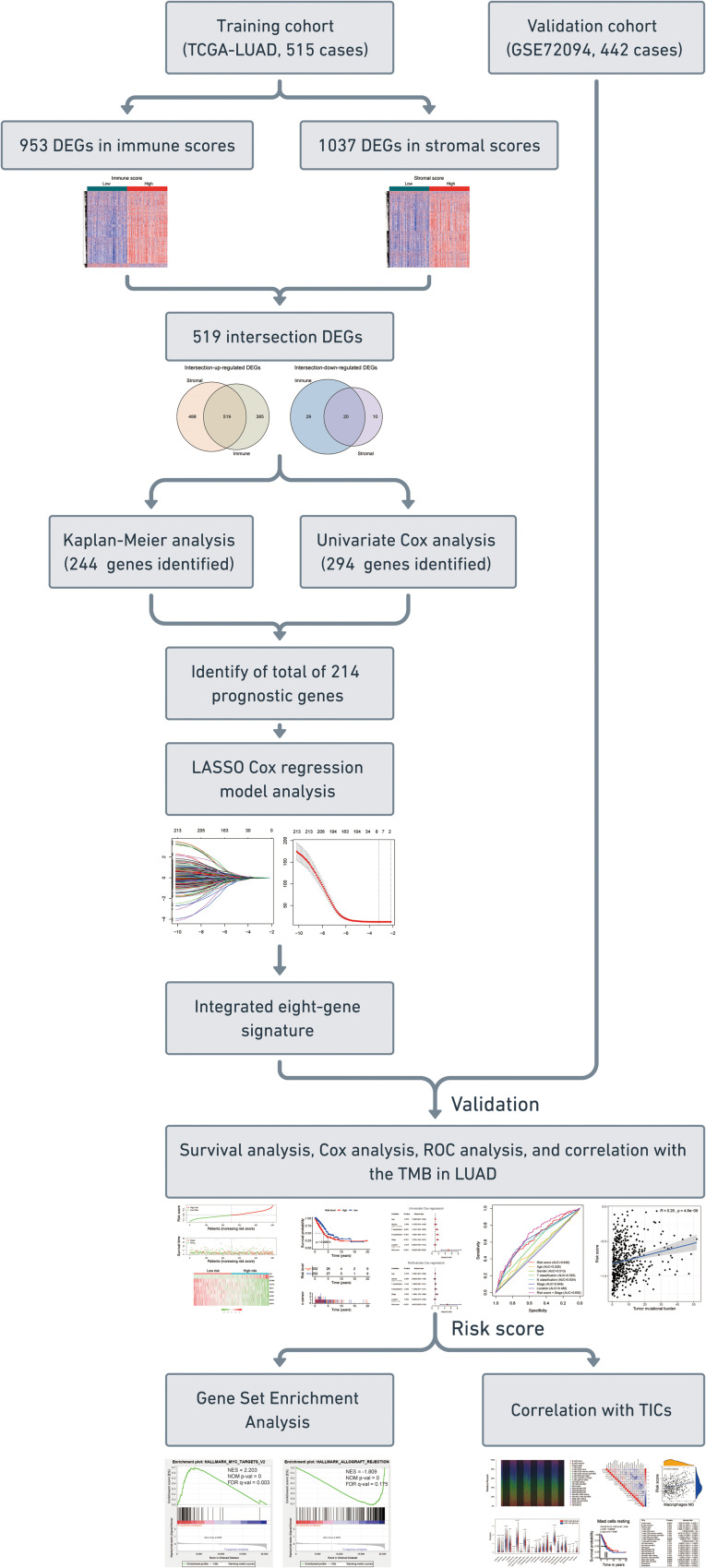
Flow chart of the study. The study was carried out in TCGA-LUAD and GSE72094 cohorts. The training cohort was used to identify prognostic genes. The prognosis analysis was validated in the validation cohort. DEGs, differentially expressed genes; LASSO, the least absolute shrinkage and selection operator Cox regression model; ROC, receiver operating characteristic; TMB, tumor mutational burden; LUAD, lung adenocarcinoma; TICs, tumor-infiltrating immune cells.

The flowchart of the present research is shown in Figure 1. 515 LUAD cases that came from TCGA-LUAD were taken as the training cohort. The dataset GSE72094 with 442 LUAD patients was used as the validation cohort. The detailed clinical characteristics of both cohorts are summarized in Table 1.

**TABLE 1:** Clinical characteristics of patients involved in the study.

**Characteristics**	**Training cohort (TCGA-LUAD, *n* = 515)**	**Validation cohort (GSE72094, *n* = 442)**
**Age at diagnosis, years**		
<60	138 (26.80%)	60 (13.57%)
≥60	363 (70.49%)	361 (81.67%)
Unknown	14 (2.72%)	21 (4.75%)
**Gender**		
Female	269 (52.23%)	240 (54.30%)
Male	232 (45.05%)	202 (45.70%)
Unknown	14 (2.72%)	0 (0.00%)
**Stage**		
I	268 (52.04%)	265 (59.95%)
II	119 (23.11%)	69 (15.61%)
III	80 (15.53%)	63 (14.25%)
IV	26 (5.05%)	17 (3.85%)
Unknown	22 (4.27%)	28 (6.33%)
**T classification**		
T1	171 (33.20%)	NA
T2	263 (51.07%)	NA
T3	45 (8.74%)	NA
T4	19 (3.69%)	NA
Unknown	17 (3.30%)	NA
**N classification**		
N0	323 (62.72%)	NA
N1	94 (18.25%)	NA
N2	70 (13.59%)	NA
N3	2 (0.39%)	NA
Unknown	26 (5.05%)	NA
**M classification**		
M0	332 (64.47%)	NA
M1	25 (4.85%)	NA
Unknown	158 (30.68%)	NA
**Tumor location**		
Right	295 (57.28%)	NA
Left	194 (37.67%)	NA
Unknown	26 (5.05%)	NA
**Race**		
White	NA	399 (90.27%)
Other	NA	18 (4.07%)
Unknown	NA	25 (5.66%)
**Kras_status**		
Wild type	NA	288 (65.16%)
Mutant	NA	154 (34.84%)
Unknown	NA	0 (0.00%)
**Egfr_status**		
Wild type	NA	395 (89.37%)
Mutant	NA	47 (10.63%)
Unknown	NA	0 (0.00%)
**Stk11_status**		
Wild type	NA	374 (84.62%)
Mutant	NA	68 (15.38%)
Unknown	NA	0 (0.00%)
**Tp53_status**		
Wild type	NA	331 (74.89%)
Mutant	NA	111 (25.11%)
Unknown	NA	0 (0.00%)

### Intersection DEGs Based on Immune and Stromal Scores

**FIGURE 2:**
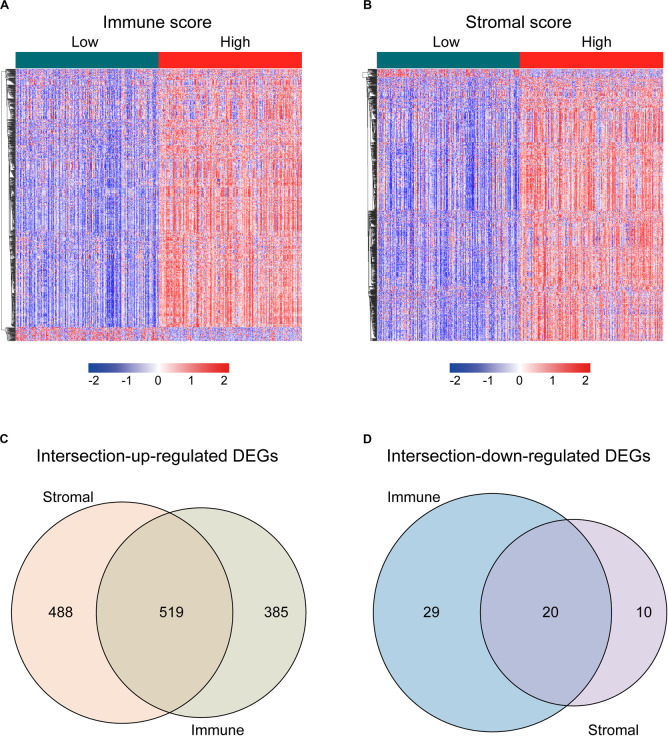
Identification of the intersection DEGs among immune and stromal scores in LUAD. **(A)** Heatmap of the DEGs of immune scores of top half (high score) vs. bottom half (low score). Cutoff: | log2(fold-change)| > 1, FDR < 0.05. **(B)** Heatmap of the DEGs of stromal scores of top half (high score) vs. bottom half (low score). Cutoff: | log2(fold-change)| > 1, FDR < 0.05. **(C,D)** Venn diagrams showing the number of intersection-upregulated DEGs (**C**) or intersection-downregulated DEGs (**D**) in stromal and immune score groups. Heatmaps were drawn based on the average method and correlation distance measurement method. Genes with higher expression are shown in red, lower expression are shown in blue, genes with the same expression level are in white. DEGs, differentially expressed genes; LUAD, lung adenocarcinoma; FDR, false discovery rate.

For identifying the DEGs among immune and stromal scores, cases in the training cohort were divided into groups of high and low scores according to their scores based on the median, and the DEG analysis was performed using the “limma” R package. Figure 2A shows a heatmap of 953 DEGs between immune score groups. Figure 2B displays a heatmap consisting of 1037 DEGs between stromal score groups. Via integrated bioinformatics analysis, we identified 519 intersection-upregulated DEGs (Figure 2C) and 20 intersection-downregulated DEGs (Figure 2D). Our subsequent analysis focused on these 539 intersection DEGs.

### Construction of Prognostic Signature From the Training Cohort

Kaplan–Meier and univariate Cox regression analyses were performed on 515 LUAD patients in the training cohort to assess the prognostic relationship between the intersection DEGs and overall survival. A total of 244 genes were extracted from the Kaplan–Meier analysis (Supplementary Table S2), while 294 genes were identified as significant in the Cox regression analysis (Supplementary Table S3). In summary, the 214 genes at the intersection of the two results are defined as potential prognostic genes for the next analysis (Supplementary Table S4).

**FIGURE 3:**
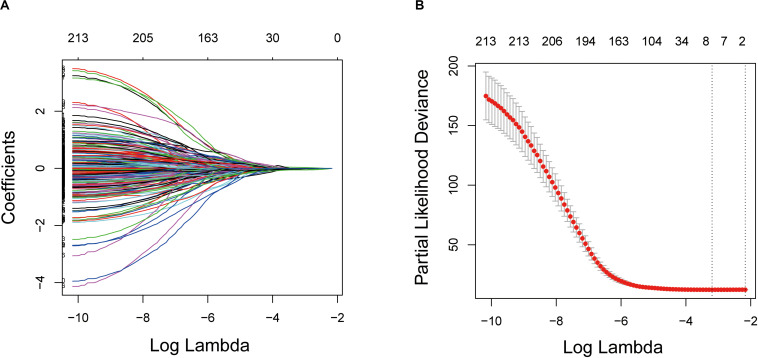
Establishment of prognostic gene signature by LASSO regression analysis. **(A)** LASSO coefficient profiles of the 214 genes in the training cohort. **(B)** A coefficient profile plot was generated against the log (lambda) sequence. Selection of the optimal parameter (lambda) in the LASSO model for the training cohort. LASSO: the least absolute shrinkage and selection operator Cox regression model.

These genes were then subjected to LASSO Cox regression analysis, and regression coefficients were calculated. The coefficient of each gene is illustrated in Figure 3A. When eight genes were included, the model achieved the best performance (Figure 3B). These genes and corresponding coefficients are shown in Table 2.

**TABLE 2:** Genes in the prognostic gene signatures.

**Gene symbol**	**Description**	**Risk coefficient**
KLRB1	Killer Cell Lectin Like Receptor B1	−0.028631486
INSL4	Insulin Like 4	0.034674929
ACSM5	Acyl-CoA Synthetase Medium Chain Family Member 5	−0.056685884
SCN7A	Sodium Voltage-Gated Channel Alpha Subunit 7	−0.007784834
P2RX1	Purinergic Receptor P2X 1	−0.018937089
MS4A1	Membrane Spanning 4-Domains A1	−0.025293606
STAP1	Signal Transducing Adaptor Family Member 1	−0.012745735
IKZF3	IKAROS Family Zinc Finger 3	−0.020045496

### Prognostic Value of the Eight-Gene Signature in the Training and Validation Cohorts

According to the gene expression level, and the risk coefficient of each gene, the risk score of each patient was calculated, which is a linear combination of the expression level of each gene weighted by its multivariate LASSO regression coefficient. The median risk score was the cutoff value for assigning patients to high-risk or low-risk groups. The prognostic value of the risk score was evaluated by comparing the survival differences between the high-risk group and the low-risk group.

The distribution of risk scores and overall survival status and the expression profiles of the eight-gene signature of the patients in the training cohort are plotted in Supplementary Figure S1A. As shown in the figure, there are more deceased in high-risk patients, and the survival time is shorter than that of low-risk patients. The heat map shows that INSL4 was highly expressed in high-risk patients, whereas KLRB1, INSL4, ACSM5, SCN7A, P2RX1, MS4A1, STAP1, and IKZF3 were under-expressed in high-risk patients. Also, we examined the performance of this eight-gene signature in predicting overall survival in the validation cohort. As shown in Supplementary Figure S1B, in the high-risk group, more death happened, and shorter survival time gained. The pattern is consistent with that in the training cohort.

**FIGURE 4:**
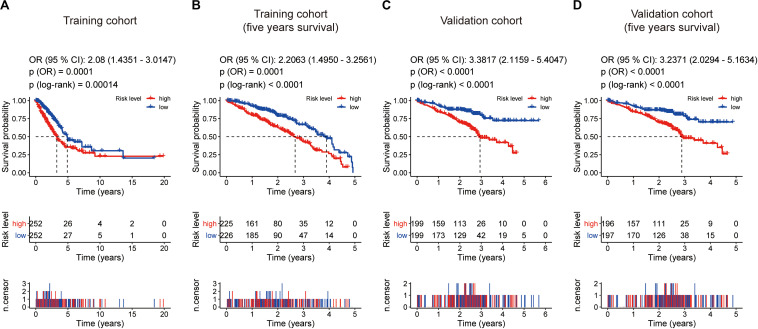
Kaplan–Meier survival analyses based on the eight-gene signature. **(A)** Training cohort based on overall survival. **(B)** training cohort based on 5-year overall survival. **(C)** Validation cohort based on overall survival. **(D)** Validation cohort based on 5-year overall survival. The differences between the two curves were determined by the two-side log-rank test with a *p* value < 0.05. The odds ratio (OR) and its 95% confidence interval (95% CI) were calculated accordingly. The number of patients at risk is listed in the middle of each plot.

**FIGURE 5:**
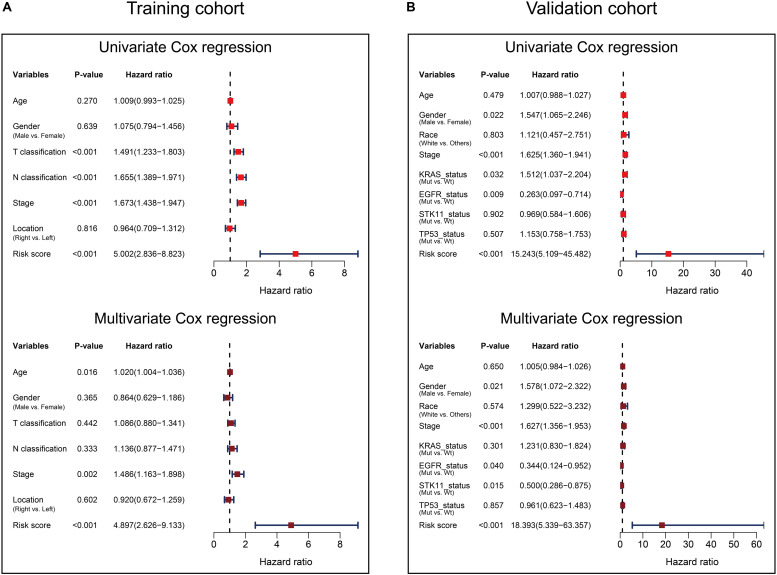
Forest plot summary of Cox analyses of prognosis. Univariate **(upper)** and multivariate **(bottom)** analyses based on the eight-gene signature and clinical covariates in training cohort based on overall survival **(A)** and validation cohort based on overall survival **(B)**. The colored solid squares represent the hazard ratio (HR), and the transverse lines through HR represent 95% confidence intervals (CI). All *p* values were calculated using the Cox regression hazards analysis.

Kaplan–Meier survival analysis showed that patients in the high-risk group were associated with a weak overall survival trend (*p* = 0.00014, Figure 4A) and an unfavorable 5-year prognosis (*p* < 0.0001, Figure 4B) in the training cohort. To confirm the efficacy of the eight-gene signature in predicting prognosis in LUAD patients, we examined the eight-gene signature in the validation cohort. Using the same classification method as before, the patients were divided into high-risk and low-risk groups according to the median risk score. Consistent with previous results, patients in the high-risk groups showed significantly worse prognosis (*p* < 0.0001, Figure 4C) and 5-year outcome (*p* < 0.0001, Figure 4D) than patients in the low-risk groups.

Univariate and multivariate Cox analyses were performed in the training and validation cohorts based overall survival, using the available co-variables including risk score, age, gender, T classification, N classification, tumor stage, tumor location, race, KRAS status, EGFR status, STK11 status, or TP53 status to confirm whether the prognostic capacity of our eight-gene signature was independent from the clinico-pathologic characteristics. In the training cohort, both univariate and multivariate Cox regression analyses indicated that the eight-gene signature was a powerful variable associated with overall survival (HR 5.002, 95% CI 2.836–8.823, *p* < 0.001, and HR 4.897, 95% CI 2.626–9.133, *p* < 0.001, respectively; Figure 5A). Consistent with that in the training cohort, the eight-gene signature also displayed pronounced capability in the validation cohort in predicting overall survival (Figure 5B). These results proved that the eight-gene signature was to be a strong and independent variable.

**FIGURE 6:**
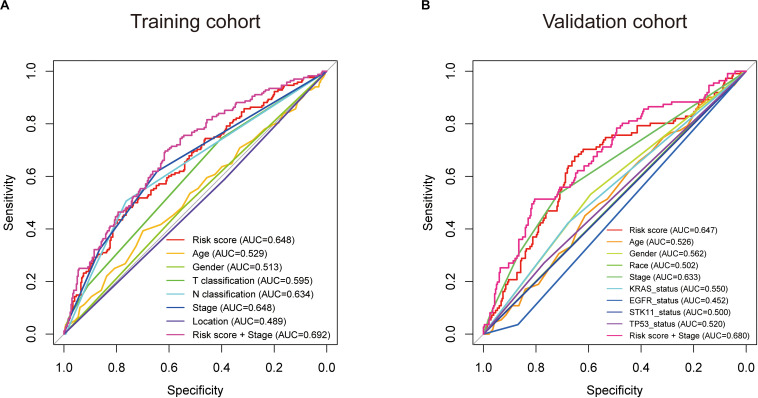
Receiver operating characteristic (ROC) analysis of the eight-gene signature risk score. ROC analysis of the sensitivity and specificity of the prognosis prediction by the eight-gene risk score, age, gender, race, T classification, N classification, tumor stage, tumor location, KRAS status, EGFR status, STK11 status, TP53 status, or risk score + tumor stage in training cohort **(A)** and validation cohort **(B)** based on overall survival. AUC, area under the ROC curve.

Subsequently, we conducted ROC analyses to assess how the eight-gene signature could behave in predicting prognosis. As shown in Figure 6A, the AUC of the eight-gene risk score model performed on overall survival in the training cohort was 0.648, which was equal to tumor stage, but superior to those of age, gender, T classification, N classification, and tumor location (0.529, 0.513, 0.595, 0.634, and 0.489, respectively). When combined risk score and tumor stage, the AUC could reach 0.692. Consistently, in the prediction model of overall survival predicted in the validation cohort, the eight-gene signature risk score also showed the best ability with AUC = 0.647, among other factors (Figure 6B). When putting risk score and tumor stage together for a diagnosis, the AUC was 0.680.

### TMB Correlation With the Gene Signature Risk Score

Furthermore, we performed correlation analyses to assess the relationship between the eight-gene signature and LUAD TMB. Spearman test was used to assess the correlation between the TMB score and the risk score. The results showed that the eight-gene signature was significantly positively correlated with TMB (*R* = 0.26, *p* = 4.8e−09, Supplementary Figure S2), further, revealing that the risk score could potentially reflect the characteristics and performance of TMB in tumors.

### Gene Set Enrichment Analysis With the Eight-Gene Signature

**FIGURE 7:**
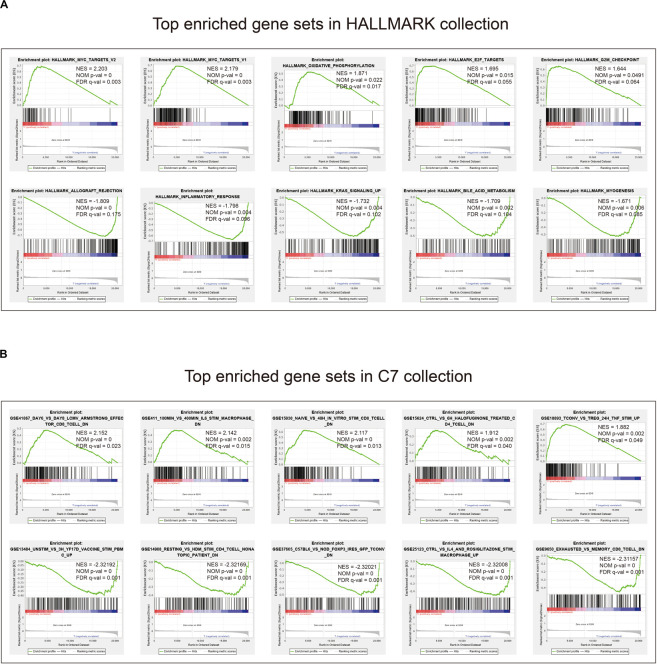
Gene set enrichment analysis. **(A)** Top enriched gene sets annotated by the HALLMARK collection between the high- and low-risk groups in the training cohort. **(B)** Top enriched gene sets annotated by the C7 collection between the high- and low-risk groups in the training cohort. Only gene sets with | NES| > 1, NOM *p*-val < 0.05, and FDR *q*-val < 0.25 were considered significant.

Given the negative correlation between the level of the eight-gene signature risk score and the prognosis of LUAD patients, the GSEA was conducted between the high and the low-risk groups. As displayed in Figure 7A and Supplementary Tables S5, S6, gene sets of HALLMARK collection were mainly enriched in pathways related to MYC, E2F, G2M, and oxidative phosphorylation in the high-risk group, and KRAS and inflammatory response in the low-risk group. For C7 collection defined by the Molecular Signatures Database, gene sets related to TNF, CD8, IL6, and CD4 were significantly enriched in the high-risk score group, whereas enrichments regarding mononuclear, CD4, CD8, macrophage, and FOXP3 were mainly seen in the low-risk score group (Figure 7B and Supplementary Tables S7, S8).

### Correlation of Risk Score With the Proportion of Tumor-Infiltrating Immune Cells (TICs)

**FIGURE 8:**
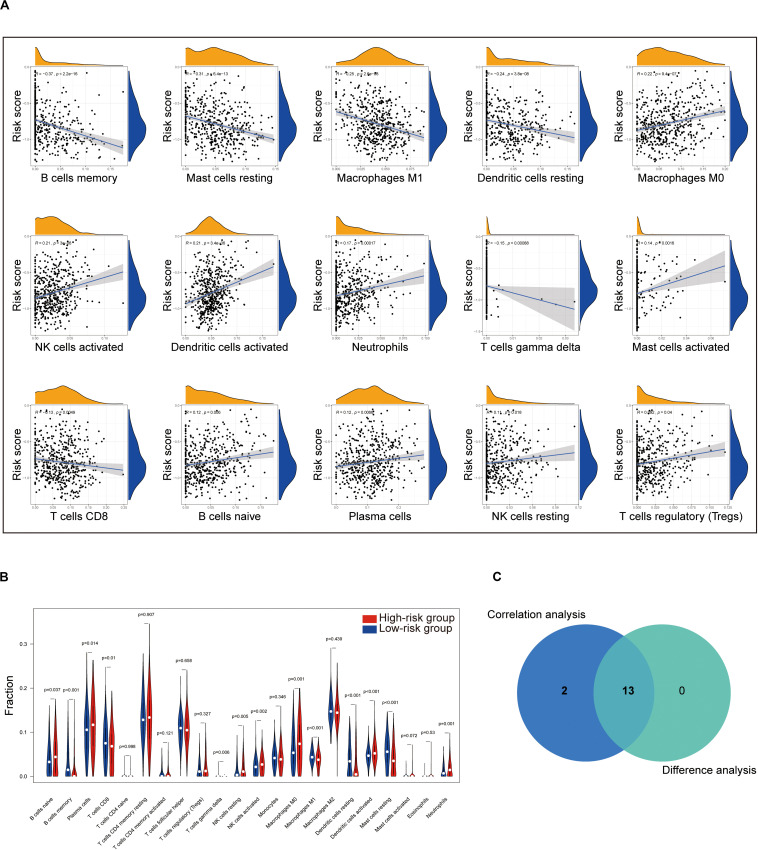
Correlation of TICs proportion with eight-gene signature risk score in the training cohort. **(A)** Only significantly correlated TICs were plotted. The blue line in each plot was fitted linear model indicating the proportion tropism of the immune cell along with risk score. The shade around the blue line represents the 95% confidence interval. The Spearman coefficient was used for the correlation test. **(B)** The violin plot shows the ratio differentiation of 22 kinds of immune cells between LUAD tumor samples with the low- or high-risk group to the median of risk score, and the Wilcoxon rank sum was used for the significance test. **(C)** Venn plot displaying 13 kinds of TICs correlated with risk score co-determined by difference and correlation tests shown in violin and scatter plots, respectively. *P* value < 0.05 is the cutoff. TIC, tumor-infiltrating immune cell; LUAD, lung adenocarcinoma.

To further check the correlation between the risk score and the immune microenvironment, as shown in Supplementary Figure S3, we used the CIBERSORT algorithm to analyze the proportion of tumor-infiltrating immune subpopulations and constructed 22 immune cell profiles in LUAD samples. Combining the results of correlation analysis (Figure 8A and Supplementary Table S9) and difference analysis (Figure 8B), a total of 13 TICs were associated with the eight-gene signature risk score (Figure 8C). Among them, NK cells resting, plasma cells, B cells naive, neutrophils, dendritic cells activated, NK cells activated, and macrophages M0 were positively correlated with risk score, whereas B cells memory, Mast cells resting, macrophages M1, dendritic cells resting, T cells gamma delta, and T cells CD8 were negatively correlated with risk score.

**FIGURE 9:**
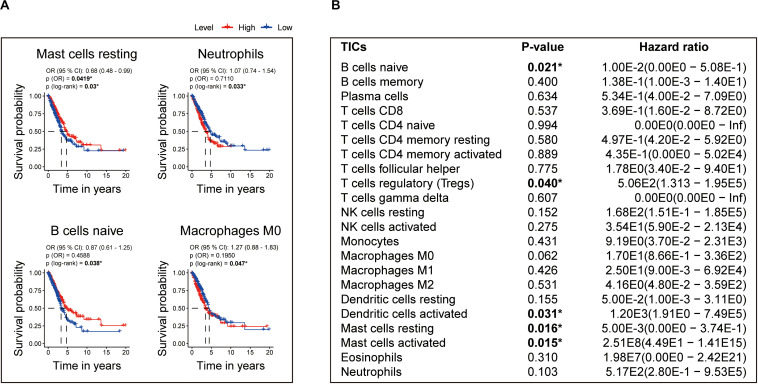
Identification of the prognostic value of each TIC based on the 22 TICs’ infiltration volume and survival data. **(A)** Kaplan–Meier survival curves. Only curves with *p* value < 0.05 in the log-rank test were plotted. The odds ratio (OR) and its 95% confidence interval (95% CI) were calculated accordingly. **(B)** Univariate Cox analysis based on each of the 22 TICs’ infiltration volume and overall survival. All *p* values were calculated using the Cox regression hazards analysis. *P value is statistically significant.

Moreover, to assess the prognostic value of each TIC, we performed univariate Cox and Kaplan–Meier analyses based on the 22 TICs’ infiltration volume and survival data, finding Mast cells resting, neutrophils, B cells naive, and macrophages M0 pronounced predicting the overall survival in Kaplan–Meier analyses (Figure 9A), whereas Mast cells activated, Mast cells resting, B cells naive, dendritic cells activated, and T cells regulatory (Tregs) have a significant prognosis value in the univariate Cox regression (Figure 9B). From these survival analyses results, Mast cells resting and B cells naive had potential prognostic value for LUAD patients.

The aforementioned results revealed that Mast cells resting and B cells naive show not only prognostic value in LUAD but also have significant correlations with the risk score. Thus, the significant infiltration with Mast cells resting and B cells naive potentially acted as one of the critical factors that the eight-gene signature holds to influence the outcome of LUAD pronounced.

## Discussion

In the present study, we built a LUAD prognostic signature by comprehensively analyzing the TCGA and GEO. By discovering the DEGs among tumor microenvironment and investigating the potential prognosis of DEGs using Kaplan–Meier, univariate Cox analyses, and the LASSO Cox regression model in the training cohort, we obtained an eight-gene signature that was pronounced related to outcome. By applying this signature in the training cohort, statistical significance was observed in univariate and multivariate Cox analysis, ROC analysis, and Kaplan–Meier curve between high-risk and low-risk groups. The prognostic ability of the eight-gene signature was also validated in the validation cohort, showing the broadness and effectiveness of the eight-gene signature in predicting LUAD prognosis. Then the GSEA and immune infiltration analyses revealed the significant pathways that linked to the eight-gene signature and the critical role that Mast cells resting and B cells naive potentially played supporting the eight-gene signature holds to influence the outcome of LUAD significantly. For research in the gene signature of LUAD, we are the first to combine tumor microenvironment scores, double screening (Kaplan–Meier and univariate Cox methods), and LASSO for training and introduce TMB for gene signature examination. What is more, we found vital immune cells that regulate the LUAD outcome among our eight-gene signature, 22 TICs, and the prognosis of 22 TICs. Such work we have done aimed to guide future research in LUAD.

After we constructed the eight-gene signature, we first confirmed its capacity to distinguish the prognosis of patients effectively. As shown in Supplementary Figure S1A, the high-risk zone not only counted more deaths but also the patients in it presented a shorter survival time than that in the low-risk zone. Moreover, the heatmap indicated that each of these eight genes had a differential expression pattern between the low- and high-risk groups. Importantly, this eight-gene signature also owned pronounced performance in the validation cohort for predicting overall survival (Supplementary Figure S1B).

In addition, we examined the prognostic value of the eight-gene signature by Kaplan–Meier analysis in the training cohort and the validation cohort based on overall survival and 5-year outcome, finding all of its predicting ability to be significant in LUAD patients (Figure 4). Furthermore, univariate and multivariate Cox analyses were performed in the two cohorts to confirm whether our eight-gene signature can be independent from other variables in predicting LUAD outcome. As plotted in Figure 5, regardless of the training cohort or validation cohort, and whether it is univariate or multivariate Cox regression analysis, the variable of risk score was always statistically significant. The results verified the predictive ability of the risk score and its independence.

To further assess the predictive power of this eight-gene signature, ROC analysis was conducted. In diagnostic tests, we can use AUC to check the accuracy and determine the predictive capacity of biomarkers (Hanley and McNeil, 1982). ROC analysis indicated that the AUC of the eight-gene signature stayed superior to other factors and could enhance the diagnostic ability when combined with the tumor stage (Figure 6). These ROC results again suggested that our signature might strengthen the predictive accuracy of prognosis in LUAD.

Our signature was composed of eight genes, which were INSL4, SCN7A, STAP1, P2RX1, IKZF3, MS4A1, KLRB1, and ACSM5, respectively. In the signature model, INSL4 was the unfavorable genes for the outcome, whereas other genes presented protective function on the prognosis of LUAD patients. INSL4 is a member of the insulin/IGF/relaxin superfamily that is restrictively expressed in the placenta (Laurent et al., 1998; Lu et al., 2005; Pollak, 2012). INSL4 upregulation was previously identified in a breast cancer cell subclone with increased invasiveness through *in vitro* selection (Brandt et al., 2002, 2005). INSL4 was also discovered as a novel target downstream of LKB1 deficiency (Yang et al., 2018). INSL4 signaling is a potential vital for LKB1-inactivated non-small-cell lung carcinoma, and its introduction helps to develop novel and effective anti-tumor strategies (Yang et al., 2018). SCN7A encodes a voltage-dependent sodium channel of the excitable membrane, and it is reported to be downregulated in colorectal carcinoma at the mRNA level (Ostasiewicz et al., 2016). Recently, a new comprehensive bioinformatics study showed that low expression of SCN7A in non-small-cell lung cancer patients was associated with poor survival (Liu Y. et al., 2020). STAP1 is a relatively unknown protein and recruits signaling proteins to receptor tyrosine kinases (Steeghs et al., 2018). STAP1 is reported to be a docking protein downstream of Tec protein tyrosine kinase, which is involved in the B-cell receptor signaling (Steeghs et al., 2018). However, the involvement of the STAP1 protein in cancer thus far remains elusive. The P7 receptor family consists of seven ionotropic P2X receptors (Adriaensen and Timmermans, 2004). P2X receptors have been specifically described in many immune cells (including platelets, lymphocytes, and macrophages), where P2X receptors are involved in the regulation of multiple functions, including platelet aggregation, apoptosis, migration, and cytokine release (Wareham et al., 2009). Wareham et al. (2009) confirmed the presence of functional P2RX1 in LAD2 cells and human lung mast cells. With the development of leukemia, the expression of P2RX1 in splenic macrophages increases. However, how P2RX1 affects lung cancer remains unclear (Chen et al., 2014). IKZF3 is a member of the Ikaros family of transcription factors, which are the crucial regulators of lymphoid differentiation (Menezes et al., 2013). IKZF3 expressions were associated with longer median progression-free survival and overall survival in multiple myeloma patients (Pourabdollah et al., 2016). IKZF3 expression also was a favorable indicator of multiple myeloma patients who received lenalidomide-based therapy (Pourabdollah et al., 2016). The IKZF3 mutation has been shown to exist in the chronic phase and blast crisis of chronic myeloid leukemia, indicating the potential role of this gene in myeloid leukemia (Menezes et al., 2013). Early lung cancer was found to have high IKZF3 expression, which indicates that IKZF3 induction often occurs before the clinical detection of lung cancer (Li et al., 2014; Terada and Liu, 2014). The MS4A1 gene located on 11q12 encodes a member of the membrane-spanning 4 domains, subfamily A, B lymphocyte antigen CD20, which plays a role in the differentiation of B lymphocytes into plasma cells (Tam et al., 2008). Also, it has been reported that MS4A1 plays a vital role in the apoptosis of B-cell lymphoma Ramos cells (Kawabata et al., 2013). In one recent comprehensive bioinformatics research, MS4A1 was confirmed to be involved in the tumor microenvironment and TMB, and low expression of MS4A1 was related to the poor prognosis of ovarian cancer (Fan et al., 2020). A study confirmed that the expression of CD20 stromal lymphocytes led to the occurrence of MS4A1 dysregulation in asbestos-related lung squamous cell carcinoma (Wright et al., 2012). The KLRB1 gene encodes the CD161 receptor in natural killer cells. The gene is also expressed in the NKT cells (Pleshkan et al., 2007). The expression of KLRB1 was most frequently associated with favorable outcomes (Gentles et al., 2015). KLRB1 has been shown to play an essential prognostic role in pan-cancer research (Gentles et al., 2015). Gentles et al. (2015) found that expression of favorably prognostic gene KLRB1 largely reflects tumor-associated leukocytes. Also, Pleshkan reported that KLRB1 gene expression was suppressed in tumor tissues in 68% of patients with non-small-cell lung cancer in his study (Pleshkan et al., 2007). ACSM5 is a protein-coding gene. Among its related pathways are Cytochrome P450 – arranged by substrate type and metabolism. Gene Ontology annotations related to this gene include GTP binding and butyrate-CoA ligase activity. An important paralog of ACSM5 is ACSM4 (Stelzer et al., 2016). The role of ACSM5 in tumor progression is still in the initial stage of research and is worthy of further exploration. In the field of lung cancer or oncology, the genes in our eight-gene signature have not been extensively studied. However, the eight-gene signature has a significant role in predicting and diagnosing LUAD in our research, indicating it or each gene in it may be the potential specific directions for future research on LUAD.

Tumor mutational burden is defined as the total number of non-synonymous mutations in tumors, and it is an emerging independent indicator of the outcome of immunotherapy treatment of multiple tumor types (including lung cancer) (Carbone et al., 2017; Chalmers et al., 2017; Le et al., 2017; Yarchoan et al., 2017; Rizvi et al., 2018). A high mutation load correlates with an immunogenic tumor microenvironment with increased expression of tumor-specific neo-antigens that can be targeted by activated immune cells (Schumacher and Schreiber, 2015; Kim and Chen, 2016). According to reports, the relationship between TMB and prognosis of many cancers is not clear, and there are still many unknowns (Owada-Ozaki et al., 2018; Wu et al., 2019). For most cancer histologies, a higher TMB is pertinent to improved survival in patients receiving immune checkpoint inhibitors, although the definition of high TMB, or the cutpoints, varies markedly between diverse tumors (Goodman et al., 2017; Lin et al., 2020). Returning to our research, we found that the risk score had a weak relationship with TMB (*p* = 4.8e−09, *R* = 0.26, Supplementary Figure S2), which further measured the connection between our eight-gene signature and LUAD, and also provided more potential breakthroughs and inspiration for tumors TMB targeted therapies.

The GSEA found that gene sets enriched in HALLMARK collection in the high-risk group mainly related to MYC, E2F, and G2M checkpoint. For the C7 collection, we found that CD4, CD8, TNF, and IL 6 were mostly seen in the enriched gene sets. MYC is a family of regulatory genes and proto-oncogenes that encode transcription factors. It is often amplified in cells grown from lung tumors, and its transfection can enhance the *in vitro* proliferation and agar cloning of human small cell lung cancer cells (Barr et al., 2000). E2F is a group of genes that encodes a family of transcription factors (TF) in higher eukaryotes (Sun et al., 2018). Evidence from cell lines, mouse models, and human tissues indicates that TFs are implicated in lung cancer tumorigenesis (Sun et al., 2018). The G2M DNA damage checkpoint is an important cell cycle checkpoint in eukaryotes, ensuring that cells will not trigger mitosis until damaged or incompletely copied DNA is fully repaired (Lobrich and Jeggo, 2007). Deficient G2M checkpoints are associated with increased lung cancer risk (Xing et al., 2007). These results elaborated on the ways and means of eight-gene signature to participate in LUAD, which can help future targeted therapy research.

In addition, the TICs’ analysis based on the CIBERSORT algorithm found that Mast cells resting and B cells naive have prognostic value in LUAD, and a significant correlation with the risk score, indicating that the infiltration of Mast cells resting and B cells naive play potential roles influencing the prognostic capacity of the eight-gene signature. Mast cells are immune cells that accumulate in the tumors and their microenvironment during disease progression (Maciel et al., 2015). Mast cells play an essential role in type I hypersensitivity and also during the early stages of the innate immune response to pathogens (Campillo-Navarro et al., 2014). Mast cells can trigger different mechanisms that contribute to the homeostasis and adequate function of the lungs (Campillo-Navarro et al., 2014). In 2000, Imada reported that stage I LUAD patients had an increased number of mast cells in tumor areas around blood vessels, and those patients with higher mast cell counts had a worse prognosis (Imada et al., 2000). B cells infiltrating lung cancer have a unique role in anti-tumor immunity. Recent studies have shown that proliferating B cells can be observed in approximately 35% of lung cancers (Gottlin et al., 2011). Besides, B lymphocytes that infiltrate the tumor can be observed at all stages of human lung cancer development (Dieu-Nosjean et al., 2014), and their performance differs between stage and histological subtype (Banat et al., 2015; Kurebayashi et al., 2016), indicating that B cells play a crucial role in the development of lung cancer (Wang et al., 2019). From our results, Mast cells resting and B cells naive have the potential to target the eight-gene signature for the treatment of LUAD, and more efforts need to be implemented to validate our results further.

Our research also has some limitations. The eight-gene signature came from retrospective data, and more prospective data are needed for proving the clinical utility of it. Also, because of the limited clinical characteristics of patients included in the TCGA cohort, we could not perform specific clinical subgroup analyses. Besides, there is currently no wet experimental data explaining the relationship between these eight genes and their mechanism in LUAD samples. Therefore, between the eight-gene signature and the prognosis of LUAD, more effort is needed to clarify the potential relationship.

## Conclusion

In conclusion, our research defined a robust eight-gene signature in LUAD. This signature was related to the prognosis of LUAD and can accurately identify the prognostic risk of patients. Notably, we evaluated the reliability and accuracy of the signature by examining in a validation cohort and determined the crucial roles of Mast cells resting and B cells naive in the prognosis of the gene signature, which could potentially promote the development of new therapies for LUAD treatment.

## Data Availability Statement

≪b≫Publicly available datasets were analyzed in this study. These data can be found here: TCGA: https://portal.gdc.cancer.gov/; GEO: https://www.ncbi.nlm.nih.gov/geo/.

## Author Contributions

CM organized and wrote the article. HL produced figures and visualized the data. JC revised the article. XZ, JZ, YZ, and ZF contributed to the literature search for the article. All authors contributed to the article and approved the submitted version.

## Conflict of Interest

The authors declare that the research was conducted in the absence of any commercial or financial relationships that could be construed as a potential conflict of interest.

## Footnotes

1 https://tcga.xenahubs.net

2 https://www.ncbi.nlm.nih.gov/geo/

3 https://bioinformatics.mdanderson.org/estimate/index.html

4 https://www.gsea-msigdb.org/gsea/downloads.jsp

5 https://www.gsea-msigdb.org/

## Supplementary Material

Supplementary Figure S1 Characteristics of the eight-gene signature. **(Upper and middle)** The distribution of risk score and patient’s survival time, as well as status for training cohort based on overall survival **(A)** and validation cohort based on overall survival **(B)**. The black dotted line is the median cutoff dividing patients into low-risk and high-risk groups. **(Bottom)** Heatmap of the eight-gene expression profiles in prognostic signature for training cohort based on overall survival **(A)** and validation cohort based on overall survival **(B)**.

Supplementary Figure S2 The correlation between the eight-gene signature and tumor mutational burden in LUAD. The blue line in each plot was fitted linear model indicating the proportion tropism of the copy number along with risk score. The shade around the blue line represents the 95% confidence interval. The Spearman coefficient was used for the correlation test. LUAD, lung adenocarcinoma.

Supplementary Figure S3 TIC profile in tumor samples in the training cohort and correlation analysis. **(A)** Bar plot showing the proportion of 22 kinds of TICs in LUAD tumor samples in the training cohort. The column names of the plot were sample ID. **(B)** Heatmap showing the correlation between 22 kinds of TICs. The numeric and shade of each small color box indicate the coefficient between two kinds of cells. X shape covered coefficient is not statistically significant. The Pearson coefficient was used for the significance tests. *P* value < 0.05 is the cutoff. TIC, tumor-infiltrating immune cell; LUAD, lung adenocarcinoma.

Supplementary Table S1 Immune and stromal scores of each sample of the TCGA-LUAD cohort.

Supplementary Table S2 Two hundred and forty-four genes were significantly predicting prognosis of LUAD patients by Kaplan–Meier analysis (*p* < 0.05).

Supplementary Table S3 Two hundred and ninety-four genes were significantly predicting the prognosis of LUAD patients by univariate Cox regression analysis (*p* < 0.05).

Supplementary Table S4 Two hundred and fourteen genes were significantly predicting the prognosis of LUAD patients by both Kaplan–Meier and univariate Cox regression analyses (*p* < 0.05).

Supplementary Table S5 Enriched gene sets in HALLMARK collection in high-risk group (| NES| > 1, NOM *p*-val < 0.05, and FDR *q*-val < 0.25).

Supplementary Table S6 Enriched gene sets in HALLMARK collection in low-risk group (| NES| > 1, NOM *p*-val < 0.05, and FDR *q*-val < 0.25).

Supplementary Table S7 Enriched gene sets in C7 collection in high-risk group (| NES| > 1, NOM *p*-val < 0.05, and FDR *q*-val < 0.25).

Supplementary Table S8 Enriched gene sets in C7 collection in low-risk group (| NES| > 1, NOM *p*-val < 0.05, and FDR *q*-val < 0.25).

Supplementary Table S9 Correlations of risk score with 22 kinds of TICs.
