# Supplementary figures and images for: Identification of a Novel Tumor Microenvironment–Associated Eight-Gene Signature for Prognosis Prediction in Lung Adenocarcinoma
Source: Front Mol Biosci. 2020 Sep 23;7:571641. doi: 10.3389/fmolb.2020.571641 (PMC7546815; doi:10.3389/fmolb.2020.571641)

**A**

## Training cohort

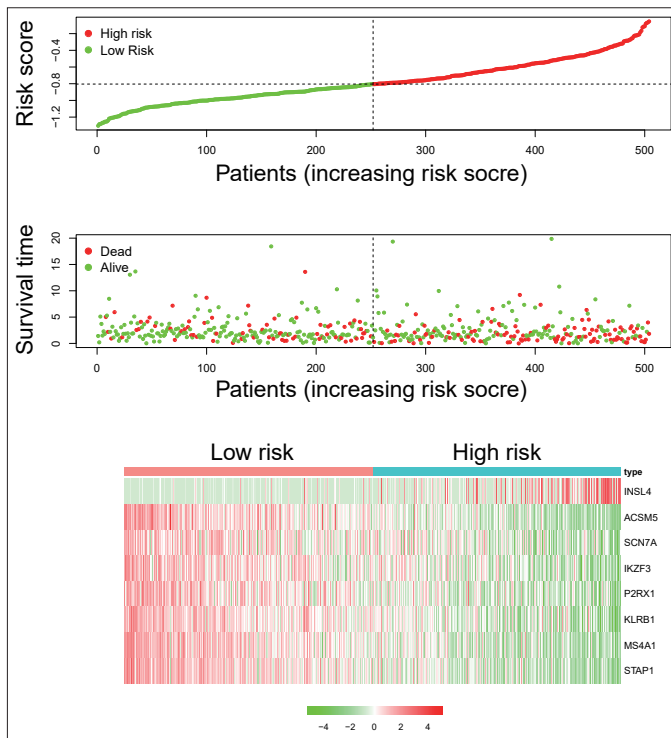**B**

## Validation cohort

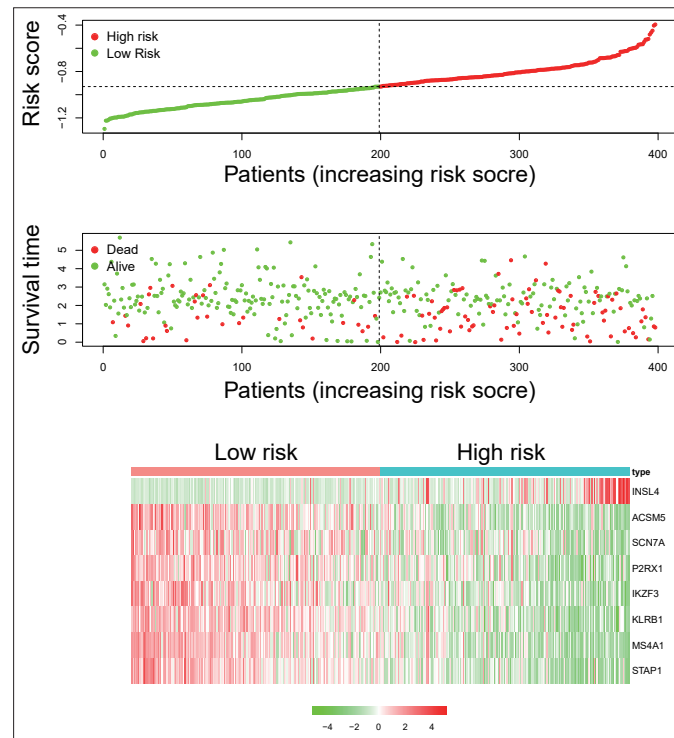

Supplement: Supplementary Figure S1 — Characteristics of the eight-gene signature. (Upper and middle) The distribution of risk score and patient’s survival time, as well as status for training cohort based on overall survival (A) and validation cohort based on overall survival (B). The black dotted line is the median cutoff dividing patients into low-risk and high-risk groups. (Bottom) Heatmap of the eight-gene expression profiles in prognostic signature for training cohort based on overall survival (A) and validation cohort based on overall survival (B). [file Image_1.pdf]

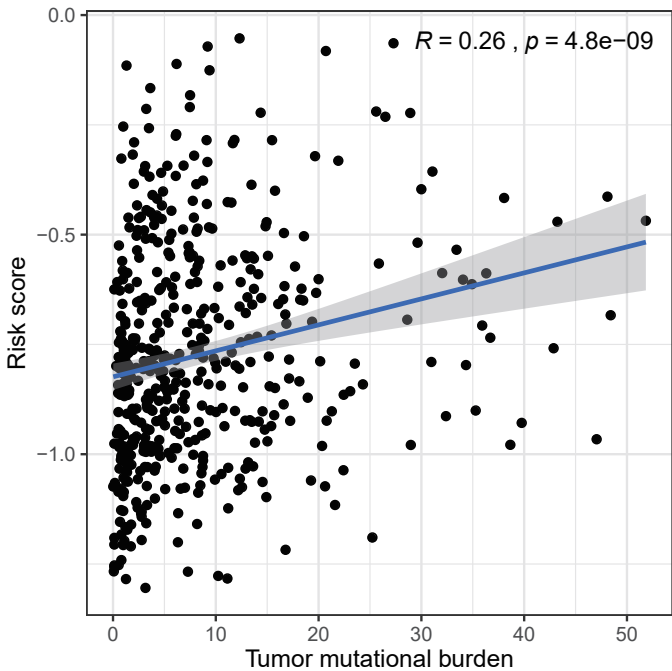

Supplement: Supplementary Figure S2 — The correlation between the eight-gene signature and tumor mutational burden in LUAD. The blue line in each plot was fitted linear model indicating the proportion tropism of the copy number along with risk score. The shade around the blue line represents the 95% confidence interval. The Spearman coefficient was used for the correlation test. LUAD, lung adenocarcinoma. [file Image_2.pdf]

### The proportion of 22 kinds of TICs in LUAD samples

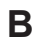

## The correlation between 22 kinds of TICs

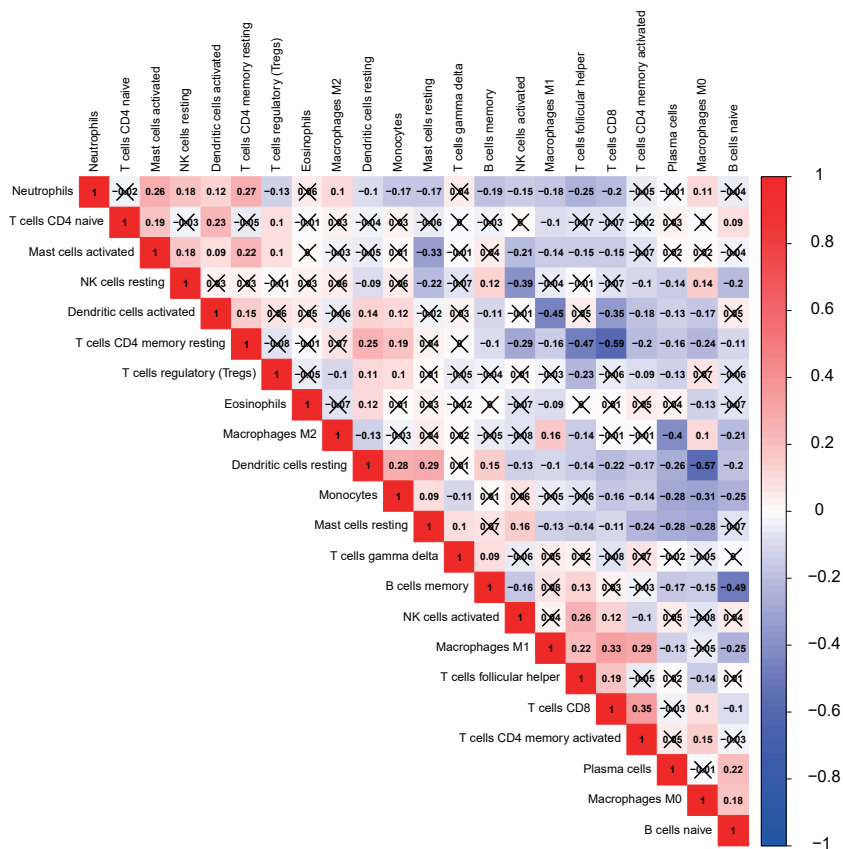

Supplement: Supplementary Figure S3 — TIC profile in tumor samples in the training cohort and correlation analysis. (A) Bar plot showing the proportion of 22 kinds of TICs in LUAD tumor samples in the training cohort. The column names of the plot were sample ID. (B) Heatmap showing the correlation between 22 kinds of TICs. The numeric and shade of each small color box indicate the coefficient between two kinds of cells. X shape covered coefficient is not statistically significant. The Pearson coefficient was used for the significance tests. P value < 0.05 is the cutoff. TIC, tumor-infiltrating immune cell; LUAD, lung adenocarcinoma. [file Image_3.pdf]
