# Supplementary material for: Identification of a Novel Tumor Microenvironment–Associated Eight-Gene Signature for Prognosis Prediction in Lung Adenocarcinoma
Source: Front Mol Biosci. 2020 Sep 23;7:571641. doi: 10.3389/fmolb.2020.571641 (PMC7546815; doi:10.3389/fmolb.2020.571641)
Supplement: Supplementary Table S1 — Immune and stromal scores of each sample of the TCGA-LUAD cohort. [file Table_1.pdf]

SUPPLEMENTARY TABLES

Identification of a novel tumor microenvironment associated eight-gene signature for prognosis prediction in lung adenocarcinoma

Xiangyu Zheng [1], Jinjun Zhang [1], Yanmin Zhang [1], Zongqiang Fu [1], Jing Cao [2], Huan Luo [3, 4], and Chao Ma [3, 5, #, *]

[1] Department of Laboratory Medicine, the Second Affiliated Hospital of Henan University of Chinese Medicine, Zhengzhou, China

[2] Department of Anatomy, College of Basic Medicine, Zhengzhou University, Zhengzhou, China

[3] Charité – Universitätsmedizin Berlin, corporate member of Freie Universität Berlin, Humboldt-Universität zu Berlin, and the Berlin Institute of Health, Berlin, Germany

[4] Klinik für Augenheilkunde, Charité – Universitätsmedizin Berlin, Corporate Member of Freie Universität Berlin, Humboldt-Universität zu Berlin, and Berlin Institute of Health, Berlin, Germany

[5] Charité - Universitätsmedizin Berlin, BCRT - Berlin Institute of Health Center for Regenerative Therapies, Berlin, Germany

[#]ORCID: Chao Ma, http://orcid.org/0000-0003-1444-4668

*Correspondence to Chao Ma, chao.ma@charite.de, 0049-015255800671, Charité - Universitätsmedizin Berlin, BCRT - Berlin Institute of Health Center for Regenerative Therapies, Berlin, Germany

Email addresses:

xiangyu003@126.com (Xiangyu Zheng)

282378685@qq.com (Jinjun Zhang)

zhangyanmin2008@163.com (Yanmin Zhang)

zongqiangfu@qq.com (Zongqiang Fu)

caojing73@126.com (Jing Cao)

huan.luo@charite.de (Huan Luo)

chao.ma@charite.de (Chao Ma)

Table S1. Immune and stromal scores of each sample of TCGA-LUAD cohort.

ID	Stromal_score	Immune_score	ESTIMATE_score
TCGA-05-4244-01	-470.99	933.74	462.76
TCGA-05-4249-01	-0.14	1033.35	1033.21
TCGA-05-4250-01	263.1	1184.84	1447.95
TCGA-05-4382-01	1205.42	1641.81	2847.24
TCGA-05-4384-01	-75.43	767.77	692.34
TCGA-05-4389-01	-397.74	1456.58	1058.84
TCGA-05-4390-01	124.48	76.91	201.39
TCGA-05-4395-01	-620.75	854.14	233.39
TCGA-05-4396-01	-714.18	-204.96	-919.14
TCGA-05-4397-01	-688.16	463.66	-224.49
TCGA-05-4398-01	362.65	1514.83	1877.48
TCGA-05-4402-01	208.5	848.38	1056.88
TCGA-05-4403-01	92.1	1327.2	1419.3
TCGA-05-4405-01	873.93	845.61	1719.54
TCGA-05-4410-01	-103.47	1449.18	1345.71
TCGA-05-4415-01	-1124.07	52.89	-1071.18
TCGA-05-4417-01	1292.02	1580.98	2873
TCGA-05-4418-01	-111.7	1233.33	1121.62
TCGA-05-4420-01	-421.95	-179.48	-601.43
TCGA-05-4422-01	-693	1425.83	732.83
TCGA-05-4424-01	509.42	1169.53	1678.95
TCGA-05-4425-01	440.03	1600.66	2040.69
TCGA-05-4426-01	-590.25	695.94	105.7
TCGA-05-4427-01	77.18	823.37	900.55
TCGA-05-4430-01	1057.05	1304.67	2361.71
TCGA-05-4432-01	-140.24	822.09	681.85
TCGA-05-4433-01	-305.36	1499.08	1193.72
TCGA-05-4434-01	617.81	1881.89	2499.7
TCGA-05-5420-01	644.13	2704.93	3349.06
TCGA-05-5423-01	90.11	1820.79	1910.9
TCGA-05-5425-01	165.33	1753.64	1918.97
TCGA-05-5428-01	93.84	831.02	924.87
TCGA-05-5429-01	-495.96	-66.98	-562.94
TCGA-05-5715-01	457.88	944.86	1402.74
TCGA-35-3615-01	-422.99	405.82	-17.17
TCGA-35-4122-01	744.23	2295.05	3039.28
TCGA-35-4123-01	622.04	2138.23	2760.27
TCGA-35-5375-01	-225.2	1039.07	813.87
TCGA-38-4625-01	-138.22	964.93	826.71
TCGA-38-4626-01	1082.46	1926.47	3008.93
TCGA-38-4627-01	2098.77	1512.22	3610.98
TCGA-38-4628-01	336.32	720.64	1056.96

TCGA-38-4629-01	918.97	1883.89	2802.86
TCGA-38-4630-01	-412.46	-361.37	-773.82
TCGA-38-4631-01	-828.13	254.82	-573.31
TCGA-38-4632-01	60.86	1531.5	1592.36
TCGA-38-6178-01	305.45	409.24	714.69
TCGA-38-7271-01	1191.36	2743.79	3935.15
TCGA-38-A44F-01	426.69	1875.62	2302.31
TCGA-44-2655-01	-437.38	861.14	423.77
TCGA-44-2656-01	731.06	2175.91	2906.97
TCGA-44-2657-01	679.86	2387.9	3067.76
TCGA-44-2659-01	641.16	1273.63	1914.79
TCGA-44-2661-01	1019.75	2577.1	3596.85
TCGA-44-2662-01	819.69	1702.24	2521.93
TCGA-44-2665-01	1496.26	1324.81	2821.06
TCGA-44-2666-01	-715.25	135.45	-579.79
TCGA-44-2668-01	959.18	1861.47	2820.64
TCGA-44-3396-01	1133.45	1885.83	3019.28
TCGA-44-3398-01	1230.85	1896.59	3127.44
TCGA-44-3918-01	1092.2	2304	3396.2
TCGA-44-3919-01	452.54	1640.92	2093.46
TCGA-44-4112-01	543.91	626.49	1170.4
TCGA-44-5643-01	-1181.08	412.41	-768.67
TCGA-44-5644-01	-1149.84	-640.82	-1790.66
TCGA-44-5645-01	204.95	1680.22	1885.17
TCGA-44-6145-01	686.36	1890.66	2577.02
TCGA-44-6146-01	-944.86	-208.62	-1153.48
TCGA-44-6147-01	552.01	1026.48	1578.49
TCGA-44-6148-01	452.62	689.74	1142.35
TCGA-44-6774-01	1888.92	1020.11	2909.03
TCGA-44-6775-01	1357.55	1481.59	2839.15
TCGA-44-6776-01	-897.75	-274.42	-1172.17
TCGA-44-6777-01	1986.65	2264.99	4251.64
TCGA-44-6778-01	643.57	2425.86	3069.43
TCGA-44-6779-01	612.95	1886.98	2499.92
TCGA-44-7659-01	-593.67	614.81	21.15
TCGA-44-7660-01	-805.51	255.7	-549.8
TCGA-44-7661-01	858.57	1668.3	2526.87
TCGA-44-7662-01	1127.28	1013.44	2140.72
TCGA-44-7667-01	-642.18	-314.04	-956.22
TCGA-44-7669-01	-163.71	737.61	573.9
TCGA-44-7670-01	-1226.8	-288.38	-1515.17
TCGA-44-7671-01	39.83	-185.72	-145.89
TCGA-44-7672-01	1021.73	2095.52	3117.25
TCGA-44-8117-01	145.48	286.52	432
TCGA-44-8119-01	941.34	726.62	1667.96

TCGA-44-8120-01	371.86	557.79	929.65
TCGA-44-A479-01	945.27	2044.8	2990.07
TCGA-44-A47A-01	-232.64	1396.49	1163.85
TCGA-44-A47B-01	-220.14	800.34	580.2
TCGA-44-A47G-01	848.83	2129.05	2977.88
TCGA-44-A4SS-01	500.03	1623.66	2123.69
TCGA-44-A4SU-01	-214.02	560.74	346.72
TCGA-49-4486-01	-1555.62	-260.68	-1816.3
TCGA-49-4487-01	518.91	1750.25	2269.17
TCGA-49-4488-01	-538.81	952.61	413.79
TCGA-49-4490-01	69.22	422.16	491.37
TCGA-49-4494-01	-441.17	896.17	455
TCGA-49-4501-01	373.71	1166.92	1540.63
TCGA-49-4505-01	409.47	1593.88	2003.35
TCGA-49-4506-01	-377.23	1325.99	948.76
TCGA-49-4507-01	-390.59	1596.48	1205.89
TCGA-49-4510-01	-383.28	134.65	-248.62
TCGA-49-4512-01	741.51	946.42	1687.93
TCGA-49-4514-01	-809.02	736.9	-72.12
TCGA-49-6742-01	-1066.96	-310.81	-1377.77
TCGA-49-6743-01	360.33	706.77	1067.1
TCGA-49-6744-01	1055.95	1972.73	3028.68
TCGA-49-6745-01	602.25	1535.71	2137.96
TCGA-49-6761-01	-197.34	1072.12	874.78
TCGA-49-6767-01	-478.74	1054.45	575.71
TCGA-49-AAQV-01	-703.28	893.75	190.47
TCGA-49-AAR0-01	-179.27	1253.03	1073.76
TCGA-49-AAR2-01	-802.72	814.21	11.49
TCGA-49-AAR3-01	602.12	2039.42	2641.54
TCGA-49-AAR4-01	-102.76	1933.89	1831.13
TCGA-49-AAR9-01	-961.13	-428.43	-1389.55
TCGA-49-AARE-01	-239.25	487.32	248.08
TCGA-49-AARN-01	-334.94	559.96	225.02
TCGA-49-AARO-01	574.25	1748.28	2322.53
TCGA-49-AARQ-01	-1111.21	617	-494.21
TCGA-49-AARR-01	437.88	1279.55	1717.43
TCGA-4B-A93V-01	-899.27	386.93	-512.34
TCGA-50-5044-01	148.32	1010.87	1159.18
TCGA-50-5045-01	1430.36	2418.37	3848.73
TCGA-50-5049-01	1639.69	2846.07	4485.76
TCGA-50-5051-01	-757.94	-204.87	-962.81
TCGA-50-5055-01	1252.77	2735.92	3988.69
TCGA-50-5066-01	913.2	2207.84	3121.04
TCGA-50-5066-02	-90.17	423.83	333.66
TCGA-50-5068-01	515.4	2162.42	2677.82

TCGA-50-5072-01	-172.59	86.92	-85.67
TCGA-50-5930-01	484.25	1171.53	1655.78
TCGA-50-5931-01	-593.62	-703.47	-1297.09
TCGA-50-5932-01	-718.49	-21.09	-739.58
TCGA-50-5933-01	1425.35	1453.04	2878.39
TCGA-50-5935-01	35	938.7	973.7
TCGA-50-5936-01	147.12	577.59	724.71
TCGA-50-5939-01	457.22	1354.23	1811.45
TCGA-50-5941-01	569.04	2174.4	2743.45
TCGA-50-5942-01	-30.52	501.69	471.17
TCGA-50-5944-01	650.65	562.64	1213.29
TCGA-50-5946-01	-1100.38	-559.22	-1659.61
TCGA-50-5946-02	960.71	324.6	1285.31
TCGA-50-6590-01	337.99	1747.56	2085.55
TCGA-50-6591-01	-917.66	-1355.85	-2273.51
TCGA-50-6592-01	298.71	1411.86	1710.57
TCGA-50-6593-01	767.06	1117.37	1884.43
TCGA-50-6594-01	-318.95	378.12	59.16
TCGA-50-6595-01	1052.86	1101.63	2154.49
TCGA-50-6597-01	-461.69	1239.12	777.44
TCGA-50-6673-01	265.73	552.94	818.66
TCGA-50-7109-01	85.6	683.67	769.27
TCGA-50-8457-01	665.15	1706.71	2371.86
TCGA-50-8459-01	1794.24	1991.35	3785.59
TCGA-50-8460-01	89.89	1476.01	1565.9
TCGA-53-7624-01	-962.99	134.43	-828.56
TCGA-53-7626-01	654.18	2014.96	2669.14
TCGA-53-7813-01	-958.2	321.87	-636.33
TCGA-53-A4EZ-01	-1114.01	-158.97	-1272.99
TCGA-55-1592-01	-83.44	678.65	595.2
TCGA-55-1594-01	-560.24	388.8	-171.44
TCGA-55-1595-01	492.12	657.33	1149.45
TCGA-55-1596-01	-776.21	115.69	-660.52
TCGA-55-5899-01	201.5	546.84	748.34
TCGA-55-6543-01	1.75	1030.03	1031.78
TCGA-55-6642-01	1331.89	1015.45	2347.34
TCGA-55-6712-01	344.08	1724.57	2068.65
TCGA-55-6968-01	-141.36	1004.19	862.82
TCGA-55-6969-01	765.58	1553.53	2319.11
TCGA-55-6970-01	-108.48	1165.87	1057.39
TCGA-55-6971-01	590.76	2188.12	2778.88
TCGA-55-6972-01	-1958.42	-890.33	-2848.75
TCGA-55-6975-01	856.13	320.2	1176.33
TCGA-55-6978-01	1379.97	2104.05	3484.02
TCGA-55-6979-01	971.94	2374.28	3346.23

TCGA-55-6980-01	731.14	1570.8	2301.94
TCGA-55-6981-01	-197.04	377.39	180.35
TCGA-55-6982-01	904.48	1098.55	2003.03
TCGA-55-6983-01	385.8	1311.47	1697.27
TCGA-55-6984-01	-977.46	317.67	-659.8
TCGA-55-6985-01	507.82	1333.03	1840.85
TCGA-55-6986-01	-389.05	795.08	406.03
TCGA-55-6987-01	512.27	2592.94	3105.21
TCGA-55-7227-01	722.58	1529.54	2252.12
TCGA-55-7281-01	625.98	1660.24	2286.22
TCGA-55-7283-01	-340.51	769.03	428.52
TCGA-55-7284-01	203.86	1150.73	1354.59
TCGA-55-7570-01	-830.19	-760.68	-1590.87
TCGA-55-7573-01	176.48	1275.68	1452.16
TCGA-55-7574-01	1026.57	1929.01	2955.58
TCGA-55-7576-01	154.81	812.72	967.54
TCGA-55-7724-01	673.85	1534.79	2208.64
TCGA-55-7725-01	-280.89	1556.43	1275.54
TCGA-55-7726-01	969.53	645.05	1614.58
TCGA-55-7727-01	-396.88	1009.84	612.96
TCGA-55-7728-01	-198.83	2426.3	2227.46
TCGA-55-7815-01	867.86	1254.93	2122.79
TCGA-55-7816-01	1655.48	1701.33	3356.81
TCGA-55-7903-01	-645.51	827.74	182.23
TCGA-55-7907-01	236.51	1114.45	1350.96
TCGA-55-7910-01	-465.83	-32.1	-497.93
TCGA-55-7911-01	-270.08	1628.37	1358.29
TCGA-55-7913-01	-1508.49	-333.2	-1841.7
TCGA-55-7914-01	-133.73	755.31	621.57
TCGA-55-7994-01	-374.59	1675.55	1300.97
TCGA-55-7995-01	-69.72	1934.22	1864.5
TCGA-55-8085-01	-253.73	1308.46	1054.73
TCGA-55-8087-01	-375.95	283.83	-92.11
TCGA-55-8089-01	572.96	2495.84	3068.8
TCGA-55-8090-01	-43.21	771.29	728.08
TCGA-55-8091-01	973.34	1378.71	2352.05
TCGA-55-8092-01	152.69	1472.48	1625.17
TCGA-55-8094-01	-1959.31	-1004.32	-2963.63
TCGA-55-8096-01	944.87	1211.05	2155.92
TCGA-55-8097-01	-64.86	660.76	595.9
TCGA-55-8203-01	-70.47	935.04	864.57
TCGA-55-8204-01	-140.39	834.84	694.45
TCGA-55-8205-01	647.93	2213.07	2861.01
TCGA-55-8206-01	313.09	1882.97	2196.07
TCGA-55-8207-01	931.94	1218.22	2150.16

TCGA-55-8208-01	1229.39	2493.47	3722.86
TCGA-55-8299-01	1285.57	2096.41	3381.98
TCGA-55-8301-01	632.28	2126.21	2758.49
TCGA-55-8302-01	-497.37	760.01	262.65
TCGA-55-8505-01	147.09	-273.52	-126.43
TCGA-55-8506-01	-78.06	812.03	733.97
TCGA-55-8507-01	-301.67	282.23	-19.43
TCGA-55-8508-01	-88.69	728.07	639.38
TCGA-55-8510-01	608.95	1830.03	2438.98
TCGA-55-8511-01	549.38	1367.24	1916.62
TCGA-55-8512-01	-591.69	215.31	-376.38
TCGA-55-8513-01	503.88	1754.91	2258.79
TCGA-55-8514-01	-466.73	640.12	173.39
TCGA-55-8614-01	23.52	236.29	259.81
TCGA-55-8615-01	-727.37	-272.08	-999.45
TCGA-55-8616-01	-419.49	278.38	-141.1
TCGA-55-8619-01	542.51	2150.43	2692.94
TCGA-55-8620-01	-1030.18	433.59	-596.58
TCGA-55-8621-01	979.96	2115.34	3095.3
TCGA-55-A48X-01	86.77	1495.84	1582.61
TCGA-55-A48Y-01	518.75	421.03	939.78
TCGA-55-A48Z-01	562.93	791.06	1353.99
TCGA-55-A490-01	234.04	683.14	917.18
TCGA-55-A491-01	122.44	1166.68	1289.12
TCGA-55-A492-01	-782.79	173.43	-609.36
TCGA-55-A493-01	84.21	1603.38	1687.58
TCGA-55-A494-01	-1324.01	-1014.81	-2338.82
TCGA-55-A4DF-01	-429.06	1137.51	708.45
TCGA-55-A4DG-01	-512.14	904.58	392.44
TCGA-55-A57B-01	248.14	918.24	1166.37
TCGA-62-8394-01	-517.26	375.35	-141.91
TCGA-62-8395-01	286.13	175.31	461.43
TCGA-62-8397-01	-240.22	895.35	655.14
TCGA-62-8398-01	-240.51	695.22	454.71
TCGA-62-8399-01	-73.15	107.36	34.22
TCGA-62-8402-01	-807.52	1313.22	505.7
TCGA-62-A46O-01	-1647.23	-1098.74	-2745.97
TCGA-62-A46P-01	-619.26	-47.42	-666.68
TCGA-62-A46R-01	-93.64	1204.85	1111.21
TCGA-62-A46S-01	-464.19	641.82	177.64
TCGA-62-A46U-01	-5.09	2534.86	2529.77
TCGA-62-A46V-01	103.57	185.23	288.8
TCGA-62-A46Y-01	-332.99	1483.53	1150.54
TCGA-62-A470-01	-703.08	133.7	-569.38
TCGA-62-A471-01	-1446.62	-186.32	-1632.94

TCGA-62-A472-01	-680.93	859.27	178.34
TCGA-64-1676-01	534.96	1720.12	2255.08
TCGA-64-1677-01	-888.39	802.42	-85.97
TCGA-64-1678-01	-1092.84	-609.99	-1702.82
TCGA-64-1679-01	1064.08	877.76	1941.84
TCGA-64-1680-01	-915.82	226.42	-689.4
TCGA-64-1681-01	99.19	948.01	1047.2
TCGA-64-5774-01	-692.62	-529.23	-1221.85
TCGA-64-5775-01	-130.35	407.23	276.87
TCGA-64-5778-01	-537.66	1864.53	1326.87
TCGA-64-5779-01	360.85	725.43	1086.28
TCGA-64-5781-01	6.68	1368.25	1374.93
TCGA-64-5815-01	1457.51	1464.83	2922.34
TCGA-67-3770-01	-189.5	1238.99	1049.5
TCGA-67-3771-01	271.68	1066.71	1338.39
TCGA-67-3772-01	771.41	1171.82	1943.23
TCGA-67-3773-01	422.7	1548.55	1971.25
TCGA-67-3774-01	-6.81	1124.26	1117.45
TCGA-67-4679-01	349.41	1172.87	1522.28
TCGA-67-6215-01	-825.59	693.22	-132.37
TCGA-67-6216-01	-337.91	1315.55	977.64
TCGA-67-6217-01	50.99	1251.38	1302.36
TCGA-69-7760-01	-678.68	-457.5	-1136.18
TCGA-69-7761-01	373.6	1822.97	2196.56
TCGA-69-7763-01	645.59	558.97	1204.56
TCGA-69-7764-01	-412.83	330.91	-81.91
TCGA-69-7765-01	1086.21	1079.11	2165.32
TCGA-69-7973-01	-201.31	-142.75	-344.06
TCGA-69-7974-01	437.33	1577.74	2015.07
TCGA-69-7978-01	939.94	2205.25	3145.2
TCGA-69-7979-01	-199.09	-181.52	-380.61
TCGA-69-7980-01	151.19	1096.91	1248.11
TCGA-69-8253-01	-689.81	379.46	-310.36
TCGA-69-8254-01	-565.74	1026.02	460.27
TCGA-69-8255-01	-987.02	1350.23	363.21
TCGA-69-8453-01	421.26	2188.01	2609.27
TCGA-69-A59K-01	-349.33	761.1	411.77
TCGA-71-6725-01	-953.41	141.19	-812.22
TCGA-71-8520-01	432.86	309.91	742.77
TCGA-73-4658-01	1336.99	1917.98	3254.97
TCGA-73-4659-01	435.09	698.1	1133.19
TCGA-73-4662-01	64.72	1359.37	1424.09
TCGA-73-4666-01	-87.83	1814.71	1726.88
TCGA-73-4668-01	-31.24	191.44	160.2
TCGA-73-4670-01	-34.45	182.59	148.14

TCGA-73-4675-01	174.63	381.76	556.39
TCGA-73-4676-01	-284.68	537.66	252.98
TCGA-73-4677-01	-462.7	605.39	142.7
TCGA-73-7498-01	186.29	685.92	872.21
TCGA-73-7499-01	-102.58	1678.33	1575.74
TCGA-73-A9RS-01	-1064.24	-104.38	-1168.62
TCGA-75-5122-01	688.81	3286.67	3975.48
TCGA-75-5125-01	255.7	1493.59	1749.29
TCGA-75-5126-01	615.01	2175.77	2790.78
TCGA-75-5146-01	-228.78	1050.63	821.85
TCGA-75-5147-01	56.27	1108.3	1164.56
TCGA-75-6203-01	744.6	2188.81	2933.42
TCGA-75-6205-01	1321.03	2536.32	3857.35
TCGA-75-6206-01	150.48	649.07	799.55
TCGA-75-6207-01	-644.98	241.76	-403.22
TCGA-75-6211-01	-179	-73.49	-252.49
TCGA-75-6212-01	-1.06	1540.49	1539.42
TCGA-75-6214-01	-1118.06	-91.39	-1209.45
TCGA-75-7025-01	356.8	1211.78	1568.58
TCGA-75-7027-01	299.69	-73.13	226.57
TCGA-75-7030-01	904.53	1186.74	2091.27
TCGA-75-7031-01	-586.62	883.23	296.62
TCGA-78-7143-01	-856.84	728.44	-128.39
TCGA-78-7145-01	-19.3	431.58	412.28
TCGA-78-7146-01	-724.67	239.26	-485.41
TCGA-78-7147-01	-1030.99	401.51	-629.49
TCGA-78-7148-01	-572.54	420.29	-152.26
TCGA-78-7149-01	-255.7	-147.69	-403.39
TCGA-78-7150-01	-67.35	-407.19	-474.54
TCGA-78-7152-01	-289.63	980.35	690.72
TCGA-78-7153-01	-774.99	31.06	-743.93
TCGA-78-7154-01	-567.99	-166.77	-734.76
TCGA-78-7155-01	-1206.67	-639.02	-1845.69
TCGA-78-7156-01	-841.28	-228.36	-1069.64
TCGA-78-7158-01	-790.49	69.42	-721.07
TCGA-78-7159-01	-92.51	104.02	11.51
TCGA-78-7160-01	555.45	1564.1	2119.55
TCGA-78-7161-01	-365	-559.96	-924.96
TCGA-78-7162-01	235.08	1047.91	1283
TCGA-78-7163-01	-1926.5	-204.63	-2131.13
TCGA-78-7166-01	-1109.82	187.51	-922.31
TCGA-78-7167-01	-754.65	-380.76	-1135.42
TCGA-78-7220-01	-881.72	-429.82	-1311.53
TCGA-78-7535-01	-226	1204.4	978.4
TCGA-78-7536-01	-511.46	653.59	142.13

TCGA-78-7537-01	-17.33	243.82	226.49
TCGA-78-7539-01	-313.48	1514.13	1200.65
TCGA-78-7540-01	-910.95	401.41	-509.54
TCGA-78-7542-01	-1411.15	335.17	-1075.98
TCGA-78-7633-01	-653.54	-337.96	-991.5
TCGA-78-8640-01	-842.08	495.31	-346.77
TCGA-78-8648-01	1939.43	2471.95	4411.38
TCGA-78-8655-01	12.6	1264.9	1277.49
TCGA-78-8660-01	-104.42	1661.9	1557.48
TCGA-78-8662-01	-1050.79	-446.71	-1497.51
TCGA-80-5607-01	-310.11	1154.88	844.77
TCGA-80-5608-01	-833.11	284.49	-548.62
TCGA-80-5611-01	-350.78	1564.17	1213.39
TCGA-83-5908-01	212.01	1830.63	2042.64
TCGA-86-6562-01	700.02	577.68	1277.7
TCGA-86-6851-01	304.41	2246.5	2550.9
TCGA-86-7701-01	561.46	1485.1	2046.57
TCGA-86-7711-01	540.5	1246.27	1786.76
TCGA-86-7713-01	-1116.3	-568.26	-1684.57
TCGA-86-7714-01	-56.39	860.95	804.56
TCGA-86-7953-01	32.37	1306.8	1339.18
TCGA-86-7954-01	349.27	1723.88	2073.15
TCGA-86-7955-01	-1774.01	-934.73	-2708.74
TCGA-86-8054-01	-676.66	-621.28	-1297.94
TCGA-86-8055-01	1229.16	898.35	2127.52
TCGA-86-8056-01	-96.79	817.3	720.51
TCGA-86-8073-01	333.15	529.26	862.41
TCGA-86-8074-01	639.36	867.19	1506.56
TCGA-86-8075-01	1090.75	912.49	2003.24
TCGA-86-8076-01	66.86	1860	1926.86
TCGA-86-8278-01	381.27	731.87	1113.14
TCGA-86-8279-01	241.37	74.03	315.4
TCGA-86-8280-01	611.91	1600.49	2212.41
TCGA-86-8281-01	-402.9	159.13	-243.78
TCGA-86-8358-01	-617.74	-263.38	-881.12
TCGA-86-8359-01	-369.79	1166.42	796.63
TCGA-86-8585-01	-118.7	1648.05	1529.35
TCGA-86-8668-01	716.37	1089.62	1806
TCGA-86-8669-01	-388.39	744.53	356.14
TCGA-86-8671-01	1394.5	2905.3	4299.81
TCGA-86-8672-01	457.64	1380.47	1838.11
TCGA-86-8673-01	-138.92	521.22	382.3
TCGA-86-8674-01	-1110.3	-506.36	-1616.66
TCGA-86-A456-01	128.48	1206.48	1334.96
TCGA-86-A4D0-01	-1233.81	-279.35	-1513.16

TCGA-86-A4JF-01	-412.06	1148.44	736.38
TCGA-86-A4P7-01	468.31	1483.82	1952.13
TCGA-86-A4P8-01	914.62	2466.21	3380.83
TCGA-91-6828-01	522.58	1559.91	2082.49
TCGA-91-6829-01	1133.05	334.11	1467.16
TCGA-91-6830-01	662.42	1121.7	1784.12
TCGA-91-6831-01	623.64	675.8	1299.44
TCGA-91-6835-01	889.87	2383.01	3272.87
TCGA-91-6836-01	-1044.37	-40.84	-1085.21
TCGA-91-6840-01	-353.97	644.69	290.72
TCGA-91-6847-01	-1800.3	-975.56	-2775.86
TCGA-91-6848-01	1015.64	1540.41	2556.05
TCGA-91-6849-01	-66.35	1208.09	1141.74
TCGA-91-7771-01	646.44	1633.44	2279.88
TCGA-91-8496-01	-7.83	1772.54	1764.71
TCGA-91-8497-01	460.48	1619.55	2080.03
TCGA-91-8499-01	-485.42	399.84	-85.58
TCGA-91-A4BC-01	962.48	2279.43	3241.92
TCGA-91-A4BD-01	-707.39	1485.39	778
TCGA-93-7347-01	874	2212.91	3086.91
TCGA-93-7348-01	423.65	737.06	1160.72
TCGA-93-8067-01	-717.86	45.21	-672.65
TCGA-93-A4JN-01	173.2	727.37	900.58
TCGA-93-A4JO-01	344.7	1898.74	2243.44
TCGA-93-A4JP-01	370.73	1175.01	1545.74
TCGA-93-A4JQ-01	805.11	1754.53	2559.64
TCGA-95-7039-01	-15.51	326.9	311.39
TCGA-95-7043-01	-660.62	-624.74	-1285.36
TCGA-95-7562-01	-149.93	681.77	531.84
TCGA-95-7567-01	36.85	754.17	791.01
TCGA-95-7944-01	24.14	1836.14	1860.28
TCGA-95-7947-01	-866.7	621.22	-245.48
TCGA-95-7948-01	-575.76	138.89	-436.87
TCGA-95-8039-01	-71.44	1382	1310.56
TCGA-95-8494-01	-581.14	867.64	286.5
TCGA-95-A4VK-01	-519.51	707.03	187.52
TCGA-95-A4VN-01	149.1	1743.76	1892.86
TCGA-95-A4VP-01	127.65	984.41	1112.07
TCGA-97-7546-01	659.77	1221.68	1881.45
TCGA-97-7547-01	47.88	616.86	664.73
TCGA-97-7552-01	311.03	2267.1	2578.12
TCGA-97-7553-01	807.24	2375.74	3182.98
TCGA-97-7554-01	1054.4	837.9	1892.29
TCGA-97-7937-01	-299.04	-38.36	-337.39
TCGA-97-7938-01	-103.95	385.76	281.81

TCGA-97-7941-01	5.96	697.48	703.44
TCGA-97-8171-01	-1263.52	-404.22	-1667.74
TCGA-97-8172-01	798.9	1642.24	2441.15
TCGA-97-8174-01	759.14	1072.77	1831.91
TCGA-97-8175-01	-354.97	1120.1	765.14
TCGA-97-8176-01	-468.26	528.15	59.89
TCGA-97-8177-01	890.52	1682.16	2572.68
TCGA-97-8179-01	-203.94	238.11	34.17
TCGA-97-8547-01	933.31	816	1749.31
TCGA-97-8552-01	-56.58	1425.01	1368.44
TCGA-97-A4LX-01	1073.04	2312.56	3385.61
TCGA-97-A4M0-01	-353.83	1550.94	1197.11
TCGA-97-A4M1-01	-62.73	1148.37	1085.64
TCGA-97-A4M2-01	310.71	2085.83	2396.55
TCGA-97-A4M3-01	-96.53	-49.79	-146.32
TCGA-97-A4M5-01	455.52	1140.02	1595.54
TCGA-97-A4M6-01	323.17	1743.83	2067
TCGA-97-A4M7-01	351.63	1686.79	2038.42
TCGA-99-7458-01	734.9	1603.9	2338.8
TCGA-99-8025-01	-12.83	124.59	111.77
TCGA-99-8028-01	1305.02	2756.73	4061.75
TCGA-99-8032-01	507.44	341.74	849.18
TCGA-99-8033-01	-311.45	1082.74	771.29
TCGA-99-AA5R-01	876.07	2313.11	3189.18
TCGA-J2-8192-01	1487.82	1558.13	3045.95
TCGA-J2-8194-01	119.69	630.25	749.95
TCGA-J2-A4AD-01	-432.07	-399.89	-831.96
TCGA-J2-A4AE-01	-605.53	1057.3	451.77
TCGA-J2-A4AG-01	334.08	1381.48	1715.55
TCGA-L4-A4E5-01	-707.04	26.44	-680.6
TCGA-L4-A4E6-01	934.25	2309.16	3243.41
TCGA-L9-A443-01	190.21	473.85	664.06
TCGA-L9-A444-01	598.66	2247.22	2845.88
TCGA-L9-A50W-01	-443.11	673.3	230.19
TCGA-L9-A5IP-01	-713.87	503.8	-210.07
TCGA-L9-A743-01	601.71	1926.79	2528.5
TCGA-L9-A7SV-01	-1057.04	-241.17	-1298.21
TCGA-L9-A8F4-01	85.67	1507.06	1592.73
TCGA-MN-A4N1-01	-670.72	-248.15	-918.87
TCGA-MN-A4N4-01	554.3	502.62	1056.92
TCGA-MN-A4N5-01	-654.03	911.88	257.85
TCGA-MP-A4SV-01	-31.72	1142.36	1110.64
TCGA-MP-A4SW-01	-55.39	1282.55	1227.16
TCGA-MP-A4SY-01	514.21	460.25	974.46

TCGA-MP-A4T4-01	709.1	1941.73	2650.84
TCGA-MP-A4T6-01	-1165.17	843.52	-321.65
TCGA-MP-A4T7-01	-689.28	760.09	70.82
TCGA-MP-A4T8-01	-62.11	-250.01	-312.11
TCGA-MP-A4T9-01	291.21	1080.11	1371.32
TCGA-MP-A4TA-01	-430.12	584.79	154.67
TCGA-MP-A4TC-01	876.36	1139.87	2016.23
TCGA-MP-A4TD-01	746.26	764.72	1510.98
TCGA-MP-A4TE-01	-1233.68	-792.11	-2025.79
TCGA-MP-A4TF-01	-1257.44	188.26	-1069.18
TCGA-MP-A4TH-01	106.63	1736.31	1842.94
TCGA-MP-A4TI-01	1180.44	2588.41	3768.84
TCGA-MP-A4TJ-01	912.74	2446.84	3359.58
TCGA-MP-A4TK-01	1259	1478.45	2737.45
TCGA-MP-A5C7-01	-1179.69	-553.01	-1732.69
TCGA-NJ-A4YF-01	-802.78	-168.6	-971.38
TCGA-NJ-A4YG-01	127.1	1076.06	1203.16
TCGA-NJ-A4YI-01	80.5	936.15	1016.64
TCGA-NJ-A4YP-01	746.63	594.12	1340.75
TCGA-NJ-A4YQ-01	-63.7	2022.22	1958.52
TCGA-NJ-A55A-01	197.29	1419.34	1616.63
TCGA-NJ-A55O-01	-339.44	922.95	583.51
TCGA-NJ-A55R-01	-437.48	-189.12	-626.6
TCGA-NJ-A7XG-01	-1710.14	-282.7	-1992.84
TCGA-O1-A52J-01	-297.9	1265.87	967.97
TCGA-S2-AA1A-01	735.65	1916.74	2652.39

Table S2. two hundred and forty-four genes were significantly predicting prognosis of LUAD patients by Kaplan-Meier analysis (p-value < 0.05).

Gene	P-value
CXorf21	3.67E-05
ATP8B4	4.54E-05
HLA-DPB1	8.24E-05
CYSLTR2	9.67E-05
IL16	0.000236
BTK	0.000237
LILRA4	0.000253
CD200R1	0.000332
AMICA1	0.000363
ARHGEF6	0.000409
ZEB2	0.000438
ACSM5	0.000454
C13orf18	0.000485
KIAA0748	0.000515
GIMAP8	0.000565
STAP1	0.000592
RASSF2	0.000595
MS4A1	0.000602
TRAF3IP3	0.000608
TLR7	0.000645
LY9	0.000832
PTPRC	0.000894
SLAMF1	0.000926
DPEP2	0.000946
ABI3BP	0.000973
CHRDL1	0.001064
PRKCB	0.001069
ITGA4	0.001118
IRF8	0.001186
P2RY13	0.001212
PSTPIP1	0.001234
RCSD1	0.001234
FCRL2	0.001237
FCRL1	0.001326
IL7R	0.001411
ST8SIA1	0.001659
CD19	0.001668
SCN2B	0.001706
HLA-DMB	0.001723
TPSAB1	0.001763
CD40LG	0.001766
KBTBD8	0.001917

CLEC10A	0.001988
RTN1	0.002047
IKZF3	0.002152
TNFAIP8L2	0.00224
LRMP	0.002358
CD37	0.002459
CCR2	0.002714
RASGRP2	0.00278
TNFSF8	0.00282
CSF2RB	0.002919
GPR34	0.002943
WDFY4	0.003007
CEACAM21	0.003171
LOC100233209	0.003185
P2RY8	0.003189
PLD4	0.003196
ARHGAP15	0.003268
SNX20	0.003355
P2RY12	0.003374
RSPO1	0.003414
CD300LF	0.003428
FLJ40330	0.003468
COL6A6	0.003558
TLR10	0.003685
GIMAP4	0.003805
LY86	0.004089
CLECL1	0.004145
PIK3CG	0.004157
CYTH4	0.004174
P2RY14	0.004264
FOLR2	0.004325
ZNF831	0.004344
CTSG	0.00457
BIN2	0.004713
GGTA1	0.004713
FGL2	0.004768
GAPT	0.004979
ANKRD44	0.004981
CCR6	0.00501
DARC	0.005173
CD33	0.005296
PLEK	0.005387
NCKAP1L	0.005699
CLEC4A	0.005735
HLA-DRA	0.00585

CD2	0.005869
SIT1	0.005911
HLA-DPA1	0.006069
CD226	0.00608
GVIN1	0.006303
KIAA0125	0.00632
HLA-DOA	0.006321
PPP2R2B	0.006823
TRAT1	0.006883
SELPLG	0.006897
SASH3	0.00698
PTGDS	0.006989
CASS4	0.007059
POU2AF1	0.007087
FLI1	0.007131
CH25H	0.0072
SPN	0.007222
HLA-DQB2	0.007505
FCRLA	0.007547
MFAP4	0.007632
ARHGAP30	0.007775
HLA-DQB1	0.007847
MPEG1	0.007995
GPR174	0.008038
DOCK2	0.008125
SLCO2B1	0.00856
DOCK8	0.008735
IKZF1	0.008903
CD180	0.009001
BTLA	0.009111
DOK2	0.009195
FCRL3	0.009231
CD84	0.009317
TNFRSF13B	0.009354
MEI1	0.009522
RGS18	0.009686
C17orf87	0.010009
AMPD1	0.010084
KCNA3	0.010116
CD48	0.010412
HGF	0.010507
C4orf7	0.011502
C10orf128	0.012217
FCRL5	0.012239
TPSB2	0.012517

CX3CR1	0.012554
SLC37A2	0.012575
MS4A2	0.01304
CD5	0.013114
CD22	0.013648
CD1B	0.013685
GIMAP6	0.01376
FGD2	0.013967
FCER1A	0.014281
PYHIN1	0.014331
GPR110	0.01454
CD79A	0.014689
P2RX1	0.014869
KCNMA1	0.015114
NLRP3	0.015373
RASGRP4	0.015565
PPP1R16B	0.015832
PAX5	0.015911
LAX1	0.01633
CD52	0.01647
HLA-DRB5	0.016679
LST1	0.017097
C8orf80	0.017282
KCNJ5	0.017306
FXYD1	0.017315
MEOX1	0.017393
CD1E	0.017641
HLA-DRB1	0.018066
RGS13	0.018438
C1orf186	0.018494
CXCR5	0.018716
SLC2A9	0.01901
FAM49A	0.019192
TLR4	0.019765
ABCA6	0.019987
SIGLECP3	0.020006
BLK	0.020015
FLT3	0.020028
GNG2	0.020271
CCR8	0.021318
CR1	0.021783
CYSLTR1	0.022149
P2RY10	0.022269
TMEM119	0.024306
EVI2B	0.024532

SIGLEC5	0.024596
CYTIP	0.024618
CD27	0.025071
IL13RA2	0.025315
CMKLR1	0.026118
TXLNB	0.026361
PLXNC1	0.026659
C5orf20	0.02761
FAIM3	0.027783
CCL14	0.027929
DNAJC5B	0.028018
COLEC12	0.028122
IL10RA	0.028327
PTPN22	0.028645
CD80	0.02867
INSL4	0.029902
LTA	0.030228
CPA3	0.030369
ABCD2	0.030596
TNFRSF17	0.030676
GLIPR2	0.030918
SIGLEC11	0.031987
NFATC2	0.032444
ACAP1	0.032666
CD79B	0.033135
SCN7A	0.033918
IGJ	0.034287
HK3	0.03519
CHIT1	0.03642
ITGAL	0.038644
TIFAB	0.038751
CCR4	0.038834
CDH23	0.038862
GFRA1	0.039346
CD3G	0.039493
HCK	0.039645
MAP1LC3C	0.039724
SVEP1	0.039814
PRAM1	0.039819
WAS	0.040085
HPGDS	0.041143
PTGDR	0.041739
DOCK11	0.041831
ADORA3	0.042424
SIGLEC8	0.042647

LSP1	0.042917
TAGAP	0.043489
KLHL6	0.043588
CD28	0.043713
NCF1B	0.044185
LOC96610	0.044377
MNDA	0.044532
C7orf58	0.044958
GFRA2	0.045138
NLRC4	0.045181
ICOS	0.045906
IGSF6	0.046131
MMRN1	0.046145
ART4	0.046329
KLRB1	0.046453
SIGLEC1	0.04678
TREM2	0.046856
CD53	0.047666
PI16	0.048225
IRF4	0.048629
S100P	0.048894
SFTPC	0.048954

Table S3. Two hundred and ninety-four genes were significantly predicting prognosis of LUAD patients by univariate Cox regression analysis (p-value < 0.05).

Gene	HR	HR.95L	HR.95H	CoxPvalue
ACSM5	0.809859	0.73325	0.894472	3.19E-05
STAP1	0.833529	0.762031	0.911736	6.91E-05
MS4A1	0.884535	0.831953	0.94044	8.72E-05
CD40LG	0.819842	0.74214	0.905679	9.23E-05
INSL4	1.086	1.041737	1.132143	0.000102
KLRB1	0.811169	0.729415	0.902085	0.000113
CCR2	0.818819	0.739427	0.906736	0.000122
P2RX1	0.811168	0.728378	0.903368	0.000139
CD200R1	0.796714	0.708662	0.895707	0.000143
FCRL1	0.856691	0.790846	0.928019	0.00015
IL16	0.778613	0.683112	0.887466	0.000178
TRAF3IP3	0.791988	0.700601	0.895295	0.000193
TLR10	0.841966	0.76855	0.922396	0.00022
IKZF3	0.832669	0.755077	0.918234	0.000243
CD5	0.822135	0.740221	0.913114	0.000255
LY9	0.813162	0.727833	0.908495	0.000256
CCR6	0.809045	0.72148	0.907237	0.000288
WDFY4	0.836023	0.758777	0.921132	0.000294
CD19	0.885967	0.828716	0.947174	0.000382
PTGDS	0.850353	0.777227	0.930359	0.00041
ACAP1	0.823273	0.737816	0.918627	0.000505
HPGDS	0.866461	0.798998	0.93962	0.000529
SIGLEC6	0.853561	0.779731	0.934382	0.000603
SLAMF1	0.828994	0.744457	0.923131	0.000632
SIGLECP3	0.843844	0.765337	0.930404	0.000655
KIAA0125	0.878271	0.814911	0.946557	0.00068
COL29A1	0.877415	0.812934	0.94701	0.000785
CXorf21	0.814443	0.72248	0.918113	0.000786
ZNF831	0.840638	0.759519	0.930422	0.0008
MEI1	0.833463	0.748705	0.927815	0.000871
TNFRSF13B	0.867418	0.797695	0.943235	0.000878
P2RY8	0.815022	0.722397	0.919522	0.00089
BLK	0.885362	0.823827	0.951492	0.000923
CD79A	0.87771	0.812378	0.948297	0.000949
RASGRP2	0.834688	0.749824	0.929156	0.000956
FAIM3	0.82525	0.735876	0.925479	0.001023
FCRLA	0.885194	0.822916	0.952185	0.001052
SCN7A	0.888808	0.828284	0.953755	0.001054
FCRL3	0.874405	0.806494	0.948034	0.001139
P2RY13	0.842673	0.760032	0.9343	0.001153
IGJ	0.881788	0.817278	0.95139	0.001172
LRMP	0.844571	0.762454	0.935532	0.001208

CD1C	0.885747	0.822524	0.95383	0.001323
AMPD1	0.874334	0.805448	0.949111	0.001339
HLA-DPB1	0.849537	0.768595	0.939003	0.001413
CD79B	0.85634	0.778038	0.942523	0.001525
LILRA4	0.862735	0.787356	0.945329	0.00155
BTK	0.843454	0.758551	0.93786	0.00166
CCR4	0.869501	0.79684	0.948788	0.001686
FCRL2	0.88641	0.821932	0.955947	0.001753
ITGAL	0.830593	0.738648	0.933982	0.001929
CASS4	0.844218	0.758239	0.939946	0.002001
MS4A2	0.887219	0.822315	0.957245	0.00202
PRKCB	0.833827	0.742904	0.935877	0.002036
HLA-DMB	0.844534	0.758347	0.940516	0.002094
CHRDL1	0.901714	0.843932	0.963451	0.002199
KLHL6	0.842115	0.754248	0.940218	0.00224
HLA-DQA1	0.876972	0.806055	0.954129	0.002278
CLECL1	0.850972	0.767092	0.944023	0.002304
CD1E	0.897851	0.837738	0.962278	0.002308
CD1B	0.888851	0.823899	0.958924	0.00234
HLA-DQB2	0.902603	0.844842	0.964313	0.00239
FMO2	0.866038	0.789222	0.950331	0.002405
CLEC10A	0.873455	0.800219	0.953394	0.00246
DPEP2	0.843264	0.755094	0.94173	0.002483
ADH1B	0.927667	0.883618	0.973912	0.002487
C4orf7	0.921289	0.873582	0.971602	0.002512
COL6A6	0.895491	0.833373	0.962238	0.002617
PYHIN1	0.851937	0.767468	0.945703	0.002631
C8orf80	0.866242	0.788498	0.951652	0.002764
SCN2B	0.87426	0.800548	0.954759	0.002788
MFAP4	0.874625	0.801066	0.954939	0.002802
IRF4	0.868031	0.791034	0.952523	0.002824
ADAMTS8	0.898282	0.837207	0.963813	0.002827
SASH3	0.830212	0.734631	0.938228	0.002866
CX3CR1	0.879393	0.807978	0.95712	0.002938
ITK	0.855643	0.772008	0.948337	0.002971
LAX1	0.868001	0.790156	0.953515	0.003149
HDC	0.880189	0.808616	0.958098	0.003186
BTLA	0.849846	0.76271	0.946937	0.0032
PAX5	0.892086	0.826831	0.962491	0.003215
PIK3CG	0.857903	0.774636	0.950119	0.003258
RGS13	0.868853	0.79107	0.954284	0.003305
HLA-DRB5	0.892992	0.827858	0.963251	0.003402
CD226	0.839653	0.746795	0.944057	0.00347
CHIT1	0.915651	0.863018	0.971495	0.00353
FCER2	0.893007	0.827616	0.963564	0.003539

KBTBD8	0.827246	0.728189	0.939778	0.003563
ARHGEF6	0.816659	0.712428	0.93614	0.003647
SAMD3	0.84425	0.753178	0.946335	0.003648
CPA3	0.905145	0.846029	0.968391	0.003828
CH25H	0.889884	0.822224	0.963111	0.003833
SNX20	0.841723	0.748862	0.946099	0.003865
ARHGAP30	0.811388	0.704034	0.935113	0.003896
CCR7	0.862609	0.780157	0.953775	0.003936
SIGLEC11	0.862434	0.77989	0.953716	0.003937
PTPRC	0.850825	0.761914	0.950112	0.004121
CD2	0.845238	0.753308	0.948387	0.00421
CD22	0.881463	0.808456	0.961062	0.004232
PTGDR	0.822424	0.719209	0.940453	0.004273
C13orf18	0.850141	0.760301	0.950598	0.004385
GPR174	0.859405	0.774114	0.954093	0.004494
CD52	0.849432	0.758985	0.950656	0.004499
HLA-DRA	0.864752	0.782232	0.955977	0.004514
SPIB	0.889229	0.820015	0.964284	0.004516
TCF21	0.882277	0.809204	0.961948	0.004519
RSPO1	0.871121	0.791898	0.958269	0.004565
AMICA1	0.8513	0.761652	0.951501	0.004574
SFTPC	0.957001	0.928354	0.986532	0.004591
CTSG	0.900163	0.836954	0.968146	0.004634
P2RY14	0.832393	0.732968	0.945306	0.004704
HLA-DRB1	0.877919	0.802075	0.960934	0.004737
FGD2	0.843662	0.74978	0.9493	0.004737
CXCR5	0.884842	0.812412	0.96373	0.004987
CD27	0.866744	0.784354	0.957788	0.005012
SIT1	0.860949	0.775425	0.955905	0.005035
POU2AF1	0.895348	0.828596	0.967477	0.005168
CYSLTR2	0.846911	0.753586	0.951794	0.005281
FLJ40330	0.902539	0.839673	0.970112	0.005375
C5orf20	0.879261	0.802822	0.962979	0.005556
CD69	0.864566	0.779814	0.958529	0.0057
ANKRD44	0.855953	0.766553	0.95578	0.005718
HLA-DOA	0.874361	0.794917	0.961745	0.005735
SPN	0.854344	0.764011	0.955359	0.005764
RCSD1	0.823896	0.718021	0.945382	0.005775
TAGAP	0.843393	0.746684	0.952627	0.006126
CD37	0.850336	0.757188	0.954942	0.006166
ST8SIA1	0.861006	0.773524	0.958384	0.00619
FAM23A	0.864929	0.779388	0.959858	0.006313
TNFRSF17	0.900518	0.83528	0.970852	0.006316
KIAA0748	0.869785	0.786903	0.961397	0.006324
IRF8	0.852583	0.760116	0.956298	0.006472

ARHGAP15	0.849582	0.755438	0.955459	0.006522
GVIN1	0.859269	0.769801	0.959137	0.006858
GPR114	0.868386	0.783847	0.962042	0.006924
LOC100233209	0.860515	0.771188	0.960189	0.007221
C16orf54	0.858038	0.76725	0.959569	0.007291
TLR7	0.866361	0.78011	0.96215	0.007338
C1orf186	0.880725	0.802563	0.966499	0.007393
ABI3BP	0.886639	0.811844	0.968325	0.007455
GAPT	0.870268	0.786019	0.963548	0.007479
IL5RA	0.889687	0.816043	0.969977	0.008015
FXYD1	0.899312	0.831391	0.972781	0.00808
HLA-DPA1	0.873801	0.79074	0.965586	0.008117
P2RY12	0.891088	0.818	0.970707	0.00827
DNAJC5B	0.879163	0.798921	0.967465	0.008357
PLD4	0.893971	0.822522	0.971625	0.008358
RASSF2	0.837772	0.734499	0.955565	0.008361
LILRA1	0.852043	0.756437	0.959733	0.008369
CLEC7A	0.869316	0.783075	0.965055	0.008608
ITGA4	0.836816	0.732441	0.956065	0.008768
C10orf128	0.869504	0.782852	0.965748	0.009037
TPSAB1	0.903843	0.837404	0.975555	0.009451
CYTIP	0.848743	0.749859	0.960669	0.009463
S100P	1.063192	1.015089	1.113576	0.009489
GFRA1	0.911672	0.850124	0.977676	0.009513
CD28	0.862106	0.770588	0.964494	0.009561
GZMK	0.886484	0.809151	0.971208	0.009674
CEACAM21	0.871733	0.785431	0.967518	0.009858
TIFAB	0.881569	0.801084	0.970141	0.009864
HGF	0.88598	0.807775	0.971756	0.01024
GIMAP8	0.832364	0.723282	0.957897	0.010463
CNR2	0.887028	0.809085	0.972478	0.010628
HLA-DQB1	0.899411	0.82915	0.975625	0.010631
IL7R	0.895933	0.823439	0.97481	0.010693
CD3G	0.877011	0.792872	0.970078	0.010762
C7	0.925889	0.872539	0.982502	0.010991
PSTPIP1	0.857554	0.761613	0.965581	0.011131
CTLA4	0.876278	0.791316	0.970361	0.011143
ABCD2	0.867933	0.777855	0.968441	0.011291
CSF2RB	0.863588	0.770803	0.967542	0.011441
ART4	0.88184	0.799872	0.972209	0.011531
FLT3	0.875987	0.790291	0.970975	0.011712
NLRC4	0.844965	0.741144	0.963331	0.011786
FAM49A	0.853867	0.754805	0.96593	0.012042
CD300LF	0.877641	0.792537	0.971884	0.012143
IL10RA	0.850702	0.749554	0.9655	0.012294

SH2D1A	0.876429	0.790061	0.972239	0.012709
FCER1A	0.923783	0.86779	0.983388	0.012954
P2RY10	0.87672	0.790301	0.972588	0.012958
GIMAP6	0.835875	0.725523	0.963012	0.013076
CCL14	0.907513	0.840528	0.979837	0.013116
CCL17	0.91831	0.858181	0.982653	0.013647
CD48	0.872069	0.782108	0.972379	0.013732
CCL13	0.910042	0.84424	0.980973	0.013831
KCNA3	0.900405	0.828036	0.979099	0.014125
THEMIS	0.878169	0.791504	0.974323	0.01426
C7orf58	0.880509	0.795035	0.975172	0.014585
IKZF1	0.860626	0.762831	0.970958	0.014734
TRAT1	0.888155	0.807341	0.977058	0.014818
RTN1	0.888041	0.80712	0.977075	0.014863
KCNMA1	0.883434	0.799338	0.976378	0.015167
INHA	1.060363	1.011241	1.111872	0.015442
TXLNB	0.876348	0.787481	0.975242	0.015542
FCRL5	0.913447	0.848754	0.983072	0.015713
EVI2B	0.860554	0.761548	0.97243	0.016027
DOCK8	0.847207	0.74015	0.969749	0.016145
MPEG1	0.866373	0.770622	0.97402	0.016373
SLAMF6	0.875082	0.784681	0.975899	0.016463
OGN	0.921195	0.861396	0.985145	0.016529
ABCA6	0.888871	0.806927	0.979136	0.016975
MS4A7	0.870231	0.776171	0.975689	0.017234
PPP1R16B	0.869161	0.774403	0.975515	0.017272
ADAM6	0.911478	0.844277	0.984028	0.017692
CPNE5	0.883916	0.797725	0.979419	0.018411
GIMAP1	0.844436	0.733553	0.972081	0.018562
NCF1B	0.887319	0.802638	0.980935	0.019485
SIGLEC8	0.898051	0.820454	0.982986	0.019693
BIN2	0.859566	0.756178	0.97709	0.020645
DMBT1	0.954409	0.917345	0.992971	0.020942
GPR65	0.864756	0.764408	0.978278	0.020947
DOCK2	0.876567	0.783516	0.98067	0.021399
TNFAIP8L2	0.870597	0.773271	0.980172	0.02196
TREM2	0.884823	0.796759	0.98262	0.022152
TNFSF8	0.884307	0.795824	0.982628	0.022268
CLEC12A	0.904904	0.830058	0.986499	0.023295
CYTH4	0.861345	0.756875	0.980235	0.023662
TPSB2	0.917775	0.851665	0.989018	0.024482
CDH23	0.894196	0.810865	0.986089	0.02505
CYSLTR1	0.886866	0.798446	0.985079	0.025057
FOLR2	0.899121	0.818809	0.987311	0.025917
ATP8B4	0.869106	0.767966	0.983566	0.026251

CD3E	0.878429	0.783412	0.984971	0.026471
CRTAM	0.885882	0.795911	0.986023	0.026585
ITGA8	0.926801	0.866527	0.991267	0.026719
SCGB1A1	0.961768	0.929062	0.995627	0.027228
CADM3	0.915238	0.845664	0.990537	0.028116
WAS	0.866952	0.763198	0.984811	0.028141
C13orf30	0.940035	0.889407	0.993546	0.028583
EOMES	0.894503	0.809506	0.988426	0.028634
SLA	0.871118	0.769724	0.985868	0.02886
CD84	0.901007	0.820576	0.989321	0.028888
LY86	0.887172	0.796762	0.98784	0.029031
CLEC4A	0.882489	0.788739	0.987382	0.029141
PRAM1	0.890716	0.802696	0.988387	0.029257
IL12RB1	0.879336	0.783257	0.9872	0.029393
AMBP	0.943514	0.895391	0.994223	0.02949
CD80	0.889207	0.799592	0.988866	0.030269
FGA	1.039515	1.003613	1.076702	0.030688
MNDA	0.895694	0.810405	0.989958	0.030954
CD53	0.874379	0.773827	0.987996	0.031263
HLA-DQA2	0.919132	0.851013	0.992704	0.031845
CD4	0.871329	0.767839	0.988768	0.032756
DARC	0.930177	0.870358	0.994108	0.032824
MAP1LC3C	0.917956	0.848544	0.993046	0.03285
MRC1	0.917976	0.84853	0.993105	0.032978
PNOC	0.909789	0.833925	0.992554	0.033321
ROS1	0.939786	0.887472	0.995184	0.033573
IGSF6	0.891055	0.80102	0.99121	0.033803
GPR34	0.892274	0.802661	0.991892	0.034796
CD1A	0.941818	0.890788	0.99577	0.034935
GGTA1	0.881143	0.783373	0.991115	0.034972
CR1	0.908788	0.831384	0.993399	0.035223
CCR5	0.878381	0.778474	0.991109	0.035297
LSP1	0.869865	0.763733	0.990746	0.035729
RASGRP4	0.883138	0.786339	0.991852	0.035898
PLEK	0.887363	0.793465	0.992374	0.036249
LST1	0.889877	0.797591	0.992841	0.036745
FCN1	0.913666	0.839336	0.994578	0.037019
ICOS	0.89598	0.808059	0.993467	0.037129
C17orf87	0.902512	0.819018	0.994517	0.038362
CD33	0.893242	0.802663	0.994044	0.038502
C6orf176	1.044831	1.002221	1.089252	0.038978
EGR2	0.884786	0.787624	0.993933	0.039161
LILRB1	0.888041	0.793255	0.994153	0.039228
CD180	0.887353	0.79198	0.994212	0.039396
P2RX7	0.904481	0.822066	0.99516	0.039447

DCN	0.890987	0.798248	0.994501	0.039561
LOC96610	0.922876	0.854902	0.996254	0.039771
KCNJ5	0.912925	0.836972	0.995771	0.039821
ADORA3	0.888536	0.793833	0.994538	0.039858
ALOX5AP	0.899603	0.812971	0.995467	0.040569
LTA	0.892989	0.800904	0.995661	0.041523
ASAM	1.098057	1.003369	1.201681	0.042046
EVI2A	0.884098	0.785036	0.99566	0.042184
KCNE1	0.919948	0.848768	0.997097	0.042285
MARCH1	0.88047	0.778633	0.995627	0.042372
MGC29506	0.92624	0.860162	0.997394	0.042452
IL21R	0.894268	0.802609	0.996395	0.042824
GIMAP4	0.860485	0.743903	0.995338	0.043087
ZBP1	0.905353	0.821967	0.9972	0.043712
LCP2	0.872573	0.764156	0.996371	0.044045
NCKAP1L	0.894506	0.802513	0.997045	0.044071
S100B	0.92529	0.857828	0.998057	0.044397
CCL19	0.933652	0.87315	0.998346	0.044603
LCP1	0.885396	0.786174	0.99714	0.044729
TPSD1	0.938979	0.882955	0.998557	0.044859
TMEM119	0.890065	0.794321	0.997349	0.04489
SLC9A9	0.867901	0.75562	0.996867	0.045031
PTPRO	0.904087	0.818563	0.998547	0.04674
COLEC12	0.909716	0.828696	0.998658	0.046792
PPP2R2B	0.893628	0.799656	0.998643	0.047264
PLA2G2D	0.935271	0.875064	0.999621	0.048708
GPNMB	0.898081	0.806626	0.999904	0.049796

Table S4. Two hundred and fourteen genes were significantly predicting the prognosis of LUAD patients by both Kaplan-Meier and univariate Cox regression analyses (p-value < 0.05).

Gene	Kaplan-Meier analysis (p-value)	Univariate Cox regression analysis			
		HR	HR.95L	HR.95H	CoxPvalue
SPN	0.007222297	0.854344	0.764011	0.955359	0.005764
ITGA4	0.001117926	0.836816	0.732441	0.956065	0.008768
ITGAL	0.038643672	0.830593	0.738648	0.933982	0.001929
ART4	0.046329101	0.88184	0.799872	0.972209	0.011531
IL10RA	0.028327347	0.850702	0.749554	0.9655	0.012294
HLA-DPA1	0.006069497	0.873801	0.79074	0.965586	0.008117
CH25H	0.00720028	0.889884	0.822224	0.963111	0.003833
HLA-DQB1	0.007847485	0.899411	0.82915	0.975625	0.010631
LOC96610	0.044377336	0.922876	0.854902	0.996254	0.039771
SCN2B	0.001706409	0.87426	0.800548	0.954759	0.002788
PRKCB	0.001068569	0.833827	0.742904	0.935877	0.002036
FAM49A	0.019191998	0.853867	0.754805	0.96593	0.012042
C4orf7	0.011501881	0.921289	0.873582	0.971602	0.002512
EVI2B	0.024531893	0.860554	0.761548	0.97243	0.016027
ZNF831	0.004344384	0.840638	0.759519	0.930422	0.0008
CD200R1	0.0003325	0.796714	0.708662	0.895707	0.000143
P2RY8	0.003189305	0.815022	0.722397	0.919522	0.00089
HLA-DRB1	0.018065765	0.877919	0.802075	0.960934	0.004737
HLA-DRB5	0.016678636	0.892992	0.827858	0.963251	0.003402
SASH3	0.006980117	0.830212	0.734631	0.938228	0.002866
HLA-DMB	0.00172258	0.844534	0.758347	0.940516	0.002094
KIAA0748	0.000514956	0.869785	0.786903	0.961397	0.006324
ICOS	0.045905931	0.89598	0.808059	0.993467	0.037129
POU2AF1	0.007087056	0.895348	0.828596	0.967477	0.005168
CYSLTR2	9.67E-05	0.846911	0.753586	0.951794	0.005281
CYSLTR1	0.022148886	0.886866	0.798446	0.985079	0.025057
HPGDS	0.04114348	0.866461	0.798998	0.93962	0.000529
TXLNB	0.026361435	0.876348	0.787481	0.975242	0.015542
KCNA3	0.010115963	0.900405	0.828036	0.979099	0.014125
CX3CR1	0.012553778	0.879393	0.807978	0.95712	0.002938
ATP8B4	4.54E-05	0.869106	0.767966	0.983566	0.026251
TLR10	0.003684876	0.841966	0.76855	0.922396	0.00022
PTPRC	0.000894487	0.850825	0.761914	0.950112	0.004121
P2RY10	0.022269148	0.87672	0.790301	0.972588	0.012958
MAP1LC3C	0.039724122	0.917956	0.848544	0.993046	0.03285
FAIM3	0.027782992	0.82525	0.735876	0.925479	0.001023
KLRB1	0.046453085	0.811169	0.729415	0.902085	0.000113
RTN1	0.002046635	0.888041	0.80712	0.977075	0.014863
IRF4	0.048628766	0.868031	0.791034	0.952523	0.002824
IRF8	0.001186027	0.852583	0.760116	0.956298	0.006472
CSF2RB	0.002918993	0.863588	0.770803	0.967542	0.011441
C7orf58	0.044958337	0.880509	0.795035	0.975172	0.014585
HLA-DQB2	0.00750471	0.902603	0.844842	0.964313	0.00239
CR1	0.021782902	0.908788	0.831384	0.993399	0.035223

INSL4	0.029902004	1.086	1.041737	1.132143	0.000102
C13orf18	0.000485054	0.850141	0.760301	0.950598	0.004385
PIK3CG	0.004156849	0.857903	0.774636	0.950119	0.003258
MNDA	0.044531961	0.895694	0.810405	0.989958	0.030954
CD48	0.01041234	0.872069	0.782108	0.972379	0.013732
CYTH4	0.00417363	0.861345	0.756875	0.980235	0.023662
ACSM5	0.00045445	0.809859	0.73325	0.894472	3.19E-05
CDH23	0.038861867	0.894196	0.810865	0.986089	0.02505
CD79B	0.033135118	0.85634	0.778038	0.942523	0.001525
CD79A	0.01468945	0.87771	0.812378	0.948297	0.000949
CD28	0.043712999	0.862106	0.770588	0.964494	0.009561
CD27	0.025070774	0.866744	0.784354	0.957788	0.005012
RASSF2	0.000595164	0.837772	0.734499	0.955565	0.008361
PTGDR	0.041738517	0.822424	0.719209	0.940453	0.004273
MEI1	0.009522146	0.833463	0.748705	0.927815	0.000871
PRAM1	0.03981899	0.890716	0.802696	0.988387	0.029257
NCKAP1L	0.005698816	0.894506	0.802513	0.997045	0.044071
FCER1A	0.014281154	0.923783	0.86779	0.983388	0.012954
CTSG	0.004570418	0.900163	0.836954	0.968146	0.004634
LY9	0.000832414	0.813162	0.727833	0.908495	0.000256
PSTPIP1	0.001233569	0.857554	0.761613	0.965581	0.011131
PYHIN1	0.014330739	0.851937	0.767468	0.945703	0.002631
C5orf20	0.027610187	0.879261	0.802822	0.962979	0.005556
LST1	0.017096734	0.889877	0.797591	0.992841	0.036745
SIGLEC11	0.031987334	0.862434	0.77989	0.953716	0.003937
C10orf128	0.01221683	0.869504	0.782852	0.965748	0.009037
FLJ40330	0.003467861	0.902539	0.839673	0.970112	0.005375
SLAMF1	0.000925969	0.828994	0.744457	0.923131	0.000632
FCRL1	0.001326315	0.856691	0.790846	0.928019	0.00015
FCRL2	0.001237166	0.88641	0.821932	0.955947	0.001753
FCRL3	0.009231357	0.874405	0.806494	0.948034	0.001139
FCRLA	0.007546822	0.885194	0.822916	0.952185	0.001052
AMICA1	0.000363364	0.8513	0.761652	0.951501	0.004574
DOCK8	0.008734813	0.847207	0.74015	0.969749	0.016145
DOCK2	0.00812459	0.876567	0.783516	0.98067	0.021399
GIMAP8	0.000564837	0.832364	0.723282	0.957897	0.010463
GIMAP4	0.003805457	0.860485	0.743903	0.995338	0.043087
GIMAP6	0.013759758	0.835875	0.725523	0.963012	0.013076
LAX1	0.016330476	0.868001	0.790156	0.953515	0.003149
LOC100233209	0.003184757	0.860515	0.771188	0.960189	0.007221
SNX20	0.003354659	0.841723	0.748862	0.946099	0.003865
TLR7	0.000645291	0.866361	0.78011	0.96215	0.007338
FCRL5	0.012238658	0.913447	0.848754	0.983072	0.015713
KBTBD8	0.001917167	0.827246	0.728189	0.939778	0.003563
GFRA1	0.039345824	0.911672	0.850124	0.977676	0.009513
SCN7A	0.033918051	0.888808	0.828284	0.953755	0.001054
MFAP4	0.007632372	0.874625	0.801066	0.954939	0.002802
LSP1	0.042917279	0.869865	0.763733	0.990746	0.035729
ANKRD44	0.004981455	0.855953	0.766553	0.95578	0.005718

IGSF6	0.046131327	0.891055	0.80102	0.99121	0.033803
NLRC4	0.045181134	0.844965	0.741144	0.963331	0.011786
HLA-DPB1	8.24E-05	0.849537	0.768595	0.939003	0.001413
RSPO1	0.003414086	0.871121	0.791898	0.958269	0.004565
CCR2	0.002713547	0.818819	0.739427	0.906736	0.000122
CCR4	0.038834214	0.869501	0.79684	0.948788	0.001686
CD80	0.028669804	0.889207	0.799592	0.988866	0.030269
CD84	0.009317234	0.901007	0.820576	0.989321	0.028888
HLA-DOA	0.006320593	0.874361	0.794917	0.961745	0.005735
S100P	0.048894336	1.063192	1.015089	1.113576	0.009489
C1orf186	0.018493718	0.880725	0.802563	0.966499	0.007393
CHIT1	0.036420196	0.915651	0.863018	0.971495	0.00353
TNFAIP8L2	0.002239795	0.870597	0.773271	0.980172	0.02196
SIGLEC8	0.042647261	0.898051	0.820454	0.982986	0.019693
FOLR2	0.004325455	0.899121	0.818809	0.987311	0.025917
ABCD2	0.03059623	0.867933	0.777855	0.968441	0.011291
BIN2	0.004712512	0.859566	0.756178	0.97709	0.020645
CD300LF	0.003428224	0.877641	0.792537	0.971884	0.012143
ARHGAP30	0.007775231	0.811388	0.704034	0.935113	0.003896
PAX5	0.015910588	0.892086	0.826831	0.962491	0.003215
PLD4	0.003196339	0.893971	0.822522	0.971625	0.008358
KCNJ5	0.017305858	0.912925	0.836972	0.995771	0.039821
RCSD1	0.001234106	0.823896	0.718021	0.945382	0.005775
KLHL6	0.043587887	0.842115	0.754248	0.940218	0.00224
COL6A6	0.003558368	0.895491	0.833373	0.962238	0.002617
PPP1R16B	0.015832477	0.869161	0.774403	0.975515	0.017272
P2RX1	0.014869037	0.811168	0.728378	0.903368	0.000139
ARHGAP15	0.003268487	0.849582	0.755438	0.955459	0.006522
IL7R	0.001411104	0.895933	0.823439	0.97481	0.010693
LRMP	0.002358286	0.844571	0.762454	0.935532	0.001208
TRAF3IP3	0.000607538	0.791988	0.700601	0.895295	0.000193
IL16	0.000235784	0.778613	0.683112	0.887466	0.000178
MS4A2	0.013040011	0.887219	0.822315	0.957245	0.00202
MS4A1	0.000602099	0.884535	0.831953	0.94044	8.72E-05
TMEM119	0.024306263	0.890065	0.794321	0.997349	0.04489
TPSB2	0.012516766	0.917775	0.851665	0.989018	0.024482
CCL14	0.027929421	0.907513	0.840528	0.979837	0.013116
HLA-DRA	0.005850191	0.864752	0.782232	0.955977	0.004514
BLK	0.020015327	0.885362	0.823827	0.951492	0.000923
P2RY14	0.004264016	0.832393	0.732968	0.945306	0.004704
P2RY12	0.003374387	0.891088	0.818	0.970707	0.00827
P2RY13	0.001212355	0.842673	0.760032	0.9343	0.001153
LTA	0.030227654	0.892989	0.800904	0.995661	0.041523
NCF1B	0.044185448	0.887319	0.802638	0.980935	0.019485
TPSAB1	0.001762669	0.903843	0.837404	0.975555	0.009451
GPR174	0.008038149	0.859405	0.774114	0.954093	0.004494
CEACAM21	0.003170658	0.871733	0.785431	0.967518	0.009858
CD22	0.013648085	0.881463	0.808456	0.961062	0.004232
DPEP2	0.000946299	0.843264	0.755094	0.94173	0.002483

PPP2R2B	0.006823299	0.893628	0.799656	0.998643	0.047264
CHRDL1	0.001064057	0.901714	0.843932	0.963451	0.002199
CASS4	0.007058865	0.844218	0.758239	0.939946	0.002001
HGF	0.010506596	0.88598	0.807775	0.971756	0.01024
CLECL1	0.004145104	0.850972	0.767092	0.944023	0.002304
TAGAP	0.043488552	0.843393	0.746684	0.952627	0.006126
BTLA	0.009110557	0.849846	0.76271	0.946937	0.0032
CLEC4A	0.005735036	0.882489	0.788739	0.987382	0.029141
ABI3BP	0.000972777	0.886639	0.811844	0.968325	0.007455
GAPT	0.004979086	0.870268	0.786019	0.963548	0.007479
CPA3	0.030369326	0.905145	0.846029	0.968391	0.003828
SIGLECP3	0.020005639	0.843844	0.765337	0.930404	0.000655
CD52	0.016470098	0.849432	0.758985	0.950656	0.004499
CD53	0.047665592	0.874379	0.773827	0.987996	0.031263
CD226	0.00608045	0.839653	0.746795	0.944057	0.00347
CD40LG	0.001765836	0.819842	0.74214	0.905679	9.23E-05
KIAA0125	0.006320313	0.878271	0.814911	0.946557	0.00068
BTK	0.000236873	0.843454	0.758551	0.93786	0.00166
AMPD1	0.010083704	0.874334	0.805448	0.949111	0.001339
RASGRP4	0.01556529	0.883138	0.786339	0.991852	0.035898
CD33	0.005296334	0.893242	0.802663	0.994044	0.038502
CD37	0.002459446	0.850336	0.757188	0.954942	0.006166
CD3G	0.039492843	0.877011	0.792872	0.970078	0.010762
GVIN1	0.006303474	0.859269	0.769801	0.959137	0.006858
FXYD1	0.017315366	0.899312	0.831391	0.972781	0.00808
CYTIP	0.024618463	0.848743	0.749859	0.960669	0.009463
CD1E	0.017641358	0.897851	0.837738	0.962278	0.002308
CD19	0.001667911	0.885967	0.828716	0.947174	0.000382
GGTA1	0.004713326	0.881143	0.783373	0.991115	0.034972
FLT3	0.020028071	0.875987	0.790291	0.970975	0.011712
TNFSF8	0.002820356	0.884307	0.795824	0.982628	0.022268
ST8SIA1	0.001659143	0.861006	0.773524	0.958384	0.00619
CD180	0.009001247	0.887353	0.79198	0.994212	0.039396
WAS	0.0400847	0.866952	0.763198	0.984811	0.028141
MPEG1	0.007994682	0.866373	0.770622	0.97402	0.016373
CCR6	0.005009768	0.809045	0.72148	0.907237	0.000288
COLEC12	0.028122105	0.909716	0.828696	0.998658	0.046792
C17orf87	0.010009097	0.902512	0.819018	0.994517	0.038362
RASGRP2	0.002779628	0.834688	0.749824	0.929156	0.000956
CXCR5	0.018716366	0.884842	0.812412	0.96373	0.004987
ARHGEF6	0.000409472	0.816659	0.712428	0.93614	0.003647
TREM2	0.046856338	0.884823	0.796759	0.98262	0.022152
CD1B	0.013684688	0.888851	0.823899	0.958924	0.00234
FGD2	0.013966888	0.843662	0.74978	0.9493	0.004737
PTGDS	0.006989324	0.850353	0.777227	0.930359	0.00041
GPR34	0.00294327	0.892274	0.802661	0.991892	0.034796
STAP1	0.000591569	0.833529	0.762031	0.911736	6.91E-05
ADORA3	0.042424359	0.888536	0.793833	0.994538	0.039858
DNAJC5B	0.028017585	0.879163	0.798921	0.967465	0.008357

TIFAB	0.038751447	0.881569	0.801084	0.970141	0.009864
WDFY4	0.003006661	0.836023	0.758777	0.921132	0.000294
DARC	0.005173413	0.930177	0.870358	0.994108	0.032824
PLEK	0.005387364	0.887363	0.793465	0.992374	0.036249
TNFRSF13B	0.009354048	0.867418	0.797695	0.943235	0.000878
TNFRSF17	0.030676106	0.900518	0.83528	0.970852	0.006316
KCNMA1	0.015114487	0.883434	0.799338	0.976378	0.015167
IGJ	0.034287444	0.881788	0.817278	0.95139	0.001172
SIT1	0.005910964	0.860949	0.775425	0.955905	0.005035
CXorf21	3.67E-05	0.814443	0.72248	0.918113	0.000786
ABCA6	0.019986911	0.888871	0.806927	0.979136	0.016975
LILRA4	0.000253064	0.862735	0.787356	0.945329	0.00155
CLEC10A	0.001988191	0.873455	0.800219	0.953394	0.00246
TRAT1	0.006882906	0.888155	0.807341	0.977058	0.014818
SFTPC	0.048953934	0.957001	0.928354	0.986532	0.004591
LY86	0.004088691	0.887172	0.796762	0.98784	0.029031
CD5	0.013113854	0.822135	0.740221	0.913114	0.000255
CD2	0.005868713	0.845238	0.753308	0.948387	0.00421
IKZF1	0.008902838	0.860626	0.762831	0.970958	0.014734
IKZF3	0.002152375	0.832669	0.755077	0.918234	0.000243
RGS13	0.018438069	0.868853	0.79107	0.954284	0.003305
ACAP1	0.032665745	0.823273	0.737816	0.918627	0.000505
C8orf80	0.0172824	0.866242	0.788498	0.951652	0.002764

Table S5. Enriched gene sets in HALLMARK collection in high risk group (| NES |> 1, NOM p-val <0.05, and FDR q-val <0.25).

NAME	SIZE	ES	NES	NOM p-val	FDR q-val
HALLMARK_MYC_TARGETS_V2	58	0.695173	2.202864	0	0.003354
HALLMARK_MYC_TARGETS_V1	188	0.684782	2.179232	0	0.002525
HALLMARK_UNFOLDED_PROTEIN_RESPONSE	106	0.449782	2.062728	0.007952	0.005446
HALLMARK_E2F_TARGETS	187	0.692561	1.694993	0.014957	0.055008
HALLMARK_OXIDATIVE_PHOSPHORYLATION	182	0.52744	1.870662	0.021654	0.017228
HALLMARK_G2M_CHECKPOINT	184	0.653637	1.64406	0.049041	0.064271

Table S6. Enriched gene sets in HALLMARK collection in low risk group (| NES |> 1, NOM p-val <0.05, and FDR q-val <0.25).

NAME	SIZE	ES	NES	NOM p-val	FDR q-val
HALLMARK_ALLOGRAFT_REJECTION	195	-0.7277	-1.80873	0	0.174512
HALLMARK_BILE_ACID_METABOLISM	112	-0.5102	-1.70854	0.00198	0.103957
HALLMARK_INFLAMMATORY_RESPONSE	197	-0.67736	-1.79792	0.004211	0.096328
HALLMARK_KRAS_SIGNALING_UP	193	-0.565	-1.73162	0.004246	0.10223
HALLMARK_MYOGENESIS	198	-0.52863	-1.67136	0.005906	0.085053
HALLMARK_PI3K_AKT_MTOR_SIGNALING	103	-0.35424	-1.78013	0.006186	0.084976
HALLMARK_COMPLEMENT	195	-0.54752	-1.80038	0.006303	0.125581
HALLMARK_APICAL_SURFACE	43	-0.5864	-1.67111	0.008197	0.078106
HALLMARK_APICAL_JUNCTION	194	-0.47693	-1.66409	0.010309	0.075843
HALLMARK_IL6_JAK_STAT3_SIGNALING	87	-0.63227	-1.69742	0.010395	0.088554
HALLMARK_KRAS_SIGNALING_DN	188	-0.50232	-1.50134	0.011278	0.146652
HALLMARK_APOPTOSIS	158	-0.43601	-1.70849	0.018519	0.090963
HALLMARK_NOTCH_SIGNALING	32	-0.49153	-1.66368	0.023454	0.070927
HALLMARK_PEROXISOME	101	-0.36148	-1.54993	0.02621	0.128459
HALLMARK_INTERFERON_GAMMA_RESPONSE	196	-0.62398	-1.69654	0.028926	0.079956
HALLMARK_HEDGEHOG_SIGNALING	35	-0.56624	-1.51242	0.039583	0.145987

Table S7. Enriched gene sets in C7 collection in high risk group (| NES |> 1, NOM p-val <0.05, and FDR q-val <0.25).

NAME	SIZE	ES	NES	NOM p-val	FDR q-val
GSE15930_NAIVE_VS_48H_IN_VITRO_STIM_CD8_TCELL_DN	187	0.62947	2.116874	0	0.013089
GSE41867_DAY6_VS_DAY8_LCMV_ARMSTRONG_EFFECTOR_CD8_TCELL_DN	178	0.476119	2.152076	0	0.023364
GSE15624_CTRL_VS_6H_HALOFUGINONE_TREATED_CD4_TCELL_DN	147	0.374566	1.912438	0.001923	0.039731
GSE411_100MIN_VS_400MIN_IL6_STIM_MACROPHAGE_DN	180	0.473055	2.142472	0.001961	0.014648
GSE18893_TCONV_VS_TREG_24H_TNF_STIM_UP	175	0.69304	1.882174	0.002008	0.048614
GSE24210_CTRL_VS_IL35_TREATED_TCONV_CD4_TCELL_DN	181	0.470325	2.102339	0.003788	0.011575
GSE31082_DN_VS_DP_THYMOCYTE_UP	170	0.43078	1.976383	0.003953	0.034054
GSE21360_SECONDARY_VS_TERTIARY_MEMORY_CD8_TCELL_UP	183	0.534217	2.03383	0.007707	0.021282
GSE22589_HEALTHY_VS_HIV_AND_SIV_INFECTED_DC_UP	174	0.338889	1.819404	0.01165	0.069396
GSE21927_SPLEEN_C57BL6_VS_EL4_TUMOR_BALBC_MONOCYTES_DN	181	0.52869	1.957053	0.013645	0.035703
GSE7568_IL4_TGFB_DEXAMETHASONE_VS_IL4_TGFB_TREATED_MACROPHAGE_UP	153	0.356687	1.817565	0.017717	0.065788
GSE41867_NAIVE_VS_DAY30_LCMV_ARMSTRONG_MEMORY_CD8_TCELL_DN	176	0.375439	1.935684	0.017857	0.034562
GSE22432_MULTIPOTENT_VS_COMMON_DC_PROGENITOR_UNTREATED_UP	175	0.476198	1.941632	0.021318	0.036841
GSE15930_NAIVE_VS_48H_IN_VITRO_STIM_IFNAB_CD8_TCELL_DN	179	0.48847	1.863556	0.028	0.053464
GSE22919_RESTING_VS_IL2_IL12_IL15_STIM_NK_CELL_UP	179	0.330352	1.755592	0.030651	0.088565
GSE7764_IL15_TREATED_VS_CTRL_NK_CELL_24H_UP	178	0.539806	1.682847	0.031621	0.119774
GSE22432_MULTIPOTENT_VS_COMMON_DC_PROGENITOR_UNTREATED_DN	176	0.367307	1.802186	0.035849	0.071139

GSE2770_TGFB_AND_IL4_VS_IL12_TREATED_ACT_CD4_TCELL_6H_DN	177	0.337729	1.644197	0.039773	0.125027
GSE22886_UNSTIM_VS_STIM_MEMORY_TCELL_DN	185	0.467589	1.828615	0.041916	0.068535
GSE21546_UNSTIM_VS_ANTI_CD3_STIM_DP_THYMOCYTES_DN	172	0.444768	1.646296	0.046154	0.127142
GSE31082_DN_VS_CD4_SP_THYMOCYTE_UP	165	0.341841	1.666892	0.046748	0.12069
GSE14000_TRANSLATED_RNA_VS_MRNA_DC_UP	184	0.384554	1.793747	0.047714	0.072266
GSE20727_CTRL_VS_ROS_INH_AND_DNFB_ALLERGEN_TREATED_DC_DN	174	0.423748	1.65438	0.049407	0.123918

Table S8. Enriched gene sets in C7 collection in low risk group (| NES |> 1, NOM p-val <0.05, and FDR q-val <0.25).

NAME	SIZE	ES	NES	NOM p-val	FDR q-val
GSE37605_C57BL6_VS_NOD_FOXP3_IRES_GFP_TCONV_DN	150	-0.51339	-2.32021	0	0.001103
GSE25123_CTRL_VS_IL4_AND_ROSIGLITAZONE_STIM_MACROPHAGE_UP	184	-0.45581	-2.32008	0	0.001121
GSE14908_RESTING_VS_HDM_STIM_CD4_TCELL_NONATOPIC_PATIENT_DN	173	-0.40511	-2.32169	0	0.001142
GSE13484_UNSTIM_VS_3H_YF17D_VACCINE_STIM_PBMC_UP	180	-0.40161	-2.32192	0	0.001184
GSE9650_EXHAUSTED_VS_MEMORY_CD8_TCELL_DN	184	-0.45343	-2.31157	0	0.001203
GSE32164_ALTERNATIVELY_ACT_M2_VS_CMYC_INHIBITED_MACROPHAGE_UP	186	-0.59089	-2.34627	0	0.001225
GSE16385_IFNG_TNF_VS_UNSTIM_MACROPHAGE_ROSIGLITAZONE_TREATED_DN	184	-0.57054	-2.32254	0	0.00123
GSE17721_PAM3CSK4_VS_GADIQUIMOD_2H_BMDC_DN	178	-0.43218	-2.31359	0	0.001242
GSE15330_GRANULOCYTE_MONOCYTE_PROGENITOR_VS_PRO_BCELL_DN	184	-0.4784	-2.2926	0	0.001272
GSE22886_NAIVE_BCELL_VS_NEUTROPHIL_UP	179	-0.5882	-2.35418	0	0.001278
GSE17721_POLYIC_VS_PAM3CSK4_2H_BMDC_UP	175	-0.43892	-2.32893	0	0.001279

GSE37533_UNTREATED_VS_PIOGLIZATONE_TREATED_CD4_TCELL_PPARG2_AND_FOXP3_TRASDUCED_DN	145	-0.45644	-2.34895	0	0.001302
GSE13411_PLASMA_CELL_VS_MEMORY_BCELL_DN	178	-0.59126	-2.29939	0	0.001306
GSE15330_WT_VS_IKAROS_KO_HSC_UP	188	-0.46133	-2.30533	0	0.001331
GSE17580_UNINFECTED_VS_S_MANSONI_INF_TEFF_UP	190	-0.55316	-2.33303	0	0.001332
GSE360_CTRL_VS_M_TUBERCULOSIS_DC_UP	184	-0.42924	-2.29989	0	0.001343
GSE6269_FLU_VS_STAPH_AUREUS_INF_PBMC_UP	159	-0.56943	-2.23021	0	0.001359
GSE2405_S_AUREUS_VS_UNTREATED_NEUTROPHIL_UP	178	-0.40532	-2.23059	0	0.001362
GSE23925_DARK_ZONE_VS_NAIVE_BCELL_UP	182	-0.47248	-2.35577	0	0.001369
GSE3982_MAST_CELL_VS_BASOPHIL_DN	176	-0.55239	-2.30533	0	0.00137
GSE37533_PPARG1_FOXP3_VS_PPARG2_FOXP3_TRANSDUCED_CD4_TCELL_DN	170	-0.54645	-2.39283	0	0.00137
GSE23568_CTRL_VS_ID3_TRANSDUCED_CD8_TCELL_UP	184	-0.54009	-2.23022	0	0.001378
GSE411_UNSTIM_VS_100MIN_IL6_STIM_MACROPHAGE_DN	185	-0.55721	-2.23129	0	0.001381

GOLDRATH_NAIVE_VS_EFF_CD8_TCELL_UP	191	-0.42783	-2.28538	0	0.001383
GSE12198_CTRL_VS_LOW_IL2_STIM_NK_CELL_UP	178	-0.46172	-2.22789	0	0.001388
GSE411_WT_VS_SOCS3_KO_MACROPHAGE_IL6_STIM_100MIN_DN	176	-0.46573	-2.33467	0	0.00139
GSE46143_CTRL_VS_LMP2A_TRANSDUCED_CD10_POS_GC_BCELL_DN	181	-0.44482	-2.23183	0	0.001402
GSE21670_UNTREATED_VS_TGFB_IL6_TREATED_STAT3_KO_CD4_TCELL_DN	178	-0.48113	-2.22916	0	0.001408
GSE14908_RESTING_VS_HDM_STIM_CD4_TCELL_ATOPIC_PATIENT_DN	178	-0.40676	-2.30608	0	0.001411
GSE23505_UNTREATED_VS_4DAY_IL6_IL1_IL23_TREATED_CD4_TCELL_UP	181	-0.48912	-2.23234	0	0.001423
GSE411_WT_VS_SOCS3_KO_MACROPHAGE_IL6_STIM_400MIN_DN	179	-0.40045	-2.22655	0	0.001426
GSE3720_UNSTIM_VS_LPS_STIM_VD1_GAMMADELTA_TCELL_UP	170	-0.51624	-2.23251	0	0.001428
GSE27786_LIN_NEG_VS_BCELL_DN	171	-0.43782	-2.27761	0	0.001436
GSE17301_CTRL_VS_48H_IFNA2_STIM_CD8_TCELL_UP	179	-0.42953	-2.23553	0	0.00144
GSE12392_WT_VS_IFNAR_KO_CD8A_NEG_SPLEEN_DC_DN	166	-0.48854	-2.39718	0	0.001443

GSE43955_TH0_VS_TGFB_IL6_IL23_TH17_ACT_CD4_TCELL_60H_DN	184	-0.48237	-2.23275	0	0.00145
GSE5099_DAY3_VS_DAY7_MCSF_TREATED_MACROPHAGE_UP	178	-0.56624	-2.33523	0	0.001454
GSE26669_CTRL_VS_COSTIM_BLOCK_MLR_CD4_TCELL_DN	171	-0.53569	-2.23734	0	0.001463
GSE21670_TGFB_VS_TGFB_AND_IL6_TREATED_CD4_TCELL_DN	178	-0.47764	-2.37382	0	0.001467
GSE21670_UNTREATED_VS_IL6_TREATED_CD4_TCELL_DN	185	-0.56602	-2.27959	0	0.001471
GSE22886_NAIVE_TCELL_VS_NKCELL_UP	176	-0.56367	-2.22513	0	0.001472
GSE33425_CD161_HIGH_VS_INT_CD8_TCELL_UP	186	-0.58942	-2.35733	0	0.001474
GSE27786_LIN_NEG_VS_CD8_TCELL_DN	188	-0.44087	-2.24656	0	0.001477
GSE22601_DOUBLE_NEGATIVE_VS_DOUBLE_POSITIVE_THYMOCYTE_DN	189	-0.46561	-2.2153	0	0.001478
GSE16755_CTRL_VS_IFNA_TREATED_MAC_UP	176	-0.44678	-2.25621	0	0.001483
GSE23568_ID3_TRANSDUCED_VS_ID3_KO_CD8_TCELL_DN	187	-0.56401	-2.21442	0	0.001485
GSE27786_LIN_NEG_VS_NKTCELL_DN	179	-0.4249	-2.24778	0	0.001486

GSE2405_0H_VS_1.5H_A_PHAGOCYTOPHILUM_STIM_NEUTROPHIL_DN	184	-0.47126	-2.23878	0	0.001486
GSE13411_IGM_MEMORY_BCELL_VS_PLASMA_CELL_UP	182	-0.51283	-2.23986	0	0.001492
GSE33424_CD161_HIGH_VS_NEG_CD8_TCELL_UP	192	-0.61683	-2.21535	0	0.001495
GSE2770_IL12_VS_IL4_TREATED_ACT_CD4_TCELL_48H_DN	178	-0.50137	-2.21314	0	0.0015
GSE45365_WT_VS_IFNAR_KO_CD8A_DC_MCMV_INFECTION_UP	181	-0.44752	-2.25557	0	0.001505
GSE22601_CD4_SINGLE_POSITIVE_VS_CD8_SINGLE_POSITIVE_THYMOCYTE_DN	178	-0.50557	-2.37692	0	0.001506
GSE13484_12H_UNSTIM_VS_YF17D_VACCINE_STIM_PBMC_UP	178	-0.44268	-2.27963	0	0.001508
GSE360_HIGH_DOSE_B_MALAYI_VS_M_TUBERCULOSIS_DC_UP	185	-0.43549	-2.257	0	0.001511
GSE14908_RESTING_VS_HDM_STIM_CD4_TCELL_NONATOPIC_PATIENT_UP	183	-0.48241	-2.24782	0	0.001511
GSE37416_0H_VS_6H_F_TULARENSIS_LVS_NEUTROPHIL_UP	176	-0.45397	-2.2154	0	0.001513
GSE3982_DC_VS_BASOPHIL_DN	181	-0.49054	-2.21235	0	0.001514
GSE2770_TGFB_AND_IL4_ACT_VS_ACT_CD4_TCELL_48H_DN	176	-0.53726	-2.25434	0	0.001515

GSE16450_CTRL_VS_IFNA_6H_STIM_MATURE_NEURON_CELL_LINE_DN	183	-0.41777	-2.33543	0	0.001523
GSE37416_CTRL_VS_12H_F_TULARENSIS_LVS_NEUTROPHIL_UP	171	-0.47207	-2.21677	0	0.00153
KAECH_DAY15_EFF_VS_MEMORY_CD8_TCELL_DN	189	-0.49946	-2.24846	0	0.001538
GSE34205_HEALTHY_VS_FLU_INF_INFANT_PBMC_UP	168	-0.5366	-2.25871	0	0.001541
GSE15330_WT_VS_IKAROS_KO_LYMPHOID_MULTIPOTENT_PROGENITOR_UP	184	-0.39829	-2.20818	0	0.001544
GSE360_CTRL_VS_L_DONOVANI_MAC_UP	185	-0.4123	-2.21146	0	0.001547
GSE22886_NAIVE_BCELL_VS_BM_PLASMA_CELL_UP	183	-0.53703	-2.21802	0	0.001549
GSE9650_NAIVE_VS_EXHAUSTED_CD8_TCELL_UP	184	-0.33887	-2.20889	0	0.001558
GSE28237_EARLY_VS_LATE_GC_BCELL_DN	180	-0.46787	-2.20835	0	0.001561
GSE2770_TGFB_AND_IL4_VS_IL4_TREATED_ACT_CD4_TCELL_6H_UP	161	-0.41657	-2.24855	0	0.001565
GSE43955_1H_VS_42H_ACT_CD4_TCELL_WITH_TGFB_IL6_UP	185	-0.50203	-2.39406	0	0.001565
GSE3982_MEMORY_CD4_TCELL_VS_BCELL_DN	174	-0.61148	-2.21812	0	0.001567

GSE41867_DAY6_EFFECTOR_VS_DAY30_MEMORY_CD8_TCELL_LCMV_ARMSTRONG_UP	182	-0.52276	-2.25939	0	0.001572
GSE27786_LIN_NEG_VS_CD4_TCELL_DN	177	-0.45496	-2.21975	0	0.00158
GSE24574_BCL6_HIGH_TFH_VS_TFH_CD4_TCELL_DN	184	-0.45952	-2.21823	0	0.001586
GSE360_CTRL_VS_T_GONDII_MAC_UP	186	-0.46985	-2.33687	0	0.001599
GSE17721_0.5H_VS_12H_GARDIQUIMOD_BMDC_DN	186	-0.4412	-2.22056	0	0.0016
GSE2770_UNTREATED_VS_TGFB_AND_IL4_TREATED_ACT_CD4_TCELL_2H_DN	183	-0.47894	-2.262	0	0.001604
GSE46606_IRF4HIGH_VS_IRF4MID_CD40L_IL2_IL5_DAY1_STIMULATED_BCELL_UP	188	-0.3962	-2.20732	0	0.001619
GSE17721_LPS_VS_PAM3CSK4_6H_BMDC_UP	189	-0.50472	-2.27047	0	0.001619
GSE37605_C57BL6_VS_NOD_FOXP3_FUSION_GFP_TCONV_UP	158	-0.53999	-2.22062	0	0.00162
GSE17580_TREG_VS_TEFF_DN	185	-0.50084	-2.27415	0	0.001633
GSE15330_HSC_VS_PRO_BCELL_UP	176	-0.40653	-2.26224	0	0.001637
GSE13484_12H_VS_3H_YF17D_VACCINE_STIM_PBMC_UP	171	-0.44868	-2.2207	0	0.001641

GSE3982_DC_VS_MAC_LPS_STIM_UP	178	-0.44383	-2.26348	0	0.001651
GSE33425_CD8_ALPHAALPHA_VS_ALPHABETA_CD161_HIGH_TCELL_UP	191	-0.63961	-2.37801	0	0.001657
GSE24574_BCL6_HIGH_VS_LOW_TFH_CD4_TCELL_UP	186	-0.52673	-2.16973	0	0.00166
GSE22886_CD8_VS_CD4_NAIVE_TCELL_UP	175	-0.46921	-2.17004	0	0.001663
GSE360_L_DONOVANI_VS_B_MALAYI_LOW_DOSE_DC_DN	187	-0.55065	-2.19585	0	0.001673
GSE27786_LSK_VS_NKCELL_DN	177	-0.46305	-2.1684	0	0.001683
GSE41867_DAY8_EFFECTOR_VS_DAY30_EXHAUSTED_CD8_TCELL_LCMV_CLONE13_DN	184	-0.51568	-2.33832	0	0.001683
GSE41867_DAY6_VS_DAY15_LCMV_ARMSTRONG_EFFECTOR_CD8_TCELL_DN	165	-0.42818	-2.1707	0	0.001687
GSE17721_0.5H_VS_24H_LPS_BMDC_DN	186	-0.48606	-2.2648	0	0.001687
GSE40274_FOXP3_VS_FOXP3_AND_EOS_TRANSDUCED_ACTIVATED_CD4_TCELL_UP	181	-0.52642	-2.1966	0	0.001688
GSE13411_SWITCHED_MEMORY_BCELL_VS_PLASMA_CELL_UP	182	-0.49317	-2.38733	0	0.001689
GSE43863_TH1_VS_LY6C_INT_CXCR5POS_EFFECTOR_CD4_TCELL_DN	188	-0.54582	-2.16843	0	0.001694

GSE17721_LPS_VS_POLYIC_8H_BMDC_DN	181	-0.50099	-2.19782	0	0.001695
GSE2770_UNTREATED_VS_TGFB_AND_IL4_TREATED_ACT_CD4_TCELL_4H_UP	184	-0.48669	-2.17075	0	0.001698
GSE33425_CD161_INT_VS_NEG_CD8_TCELL_DN	192	-0.53485	-2.19806	0	0.001701
GSE17580_UNINFECTED_VS_S_MANSONI_INF_TREG_UP	185	-0.56246	-2.19325	0	0.001702
GSE41867_NAIVE_VS_DAY15_LCMV_ARMSTRONG_EFFECTOR_CD8_TCELL_DN	183	-0.47208	-2.1933	0	0.001703
GSE9988_LPS_VS_CTRL_TREATED_MONOCYTE_DN	182	-0.43591	-2.1708	0	0.00171
GSE45365_NK_CELL_VS_CD8_TCELL_MCMV_INFECTION_DN	179	-0.60604	-2.17127	0	0.001715
GSE2770_TGFB_AND_IL4_ACT_VS_ACT_CD4_TCELL_6H_UP	168	-0.43203	-2.19829	0	0.001718
GSE43955_TH0_VS_TGFB_IL6_TH17_ACT_CD4_TCELL_4H_DN	189	-0.49412	-2.16757	0	0.001719
GSE5589_UNSTIM_VS_45MIN_LPS_AND_IL6_STIM_MACROPHAGE_UP	185	-0.44483	-2.19876	0	0.001724
GSE360_HIGH_DOSE_B_MALAYI_VS_M_TUBERCULOSIS_MAC_UP	189	-0.52069	-2.26643	0	0.001724
GSE10094_LCMV_VS_LISTERIA_IND_EFF_CD4_TCELL_UP	179	-0.43452	-2.17128	0	0.001727

GSE29618_MONOCYTE_VS_MDC_DAY7_FLU_VACCINE_DN	173	-0.57558	-2.16638	0	0.001728
GSE16385_ROSIGLITAZONE_IL4_VS_IL4_ALONE_STIM_MACROPHAGE_12H_UP	181	-0.55742	-2.40258	0	0.001731
GSE25123_CTRL_VS_IL4_STIM_MACROPHAGE_UP	181	-0.48103	-2.20085	0	0.001735
GSE32901_TH1_VS_TH17_ENRICHED_CD4_TCELL_UP	163	-0.51793	-2.17131	0	0.00174
GSE3982_BCELL_VS_NKCELL_UP	177	-0.56837	-2.17834	0	0.00174
GSE22886_NAIVE_CD8_TCELL_VS_DC_UP	177	-0.6538	-2.20238	0	0.00174
GSE11864_UNTREATED_VS_CSF1_IN_MAC_UP	174	-0.51543	-2.1989	0	0.001741
GSE13738_TCR_VS_BYSTANDER_ACTIVATED_CD4_TCELL_UP	173	-0.51473	-2.17839	0	0.001741
GSE3337_CTRL_VS_4H_IFNG_IN_CD8POS_DC_UP	188	-0.50351	-2.17283	0	0.001741
GSE29164_DAY3_VS_DAY7_CD8_TCELL_AND_IL12_TREATED_MELANOMA_UP	180	-0.4648	-2.17958	0	0.001744
GSE36888_STAT5_AB_KNOCKIN_VS_WT_TCELL_IL2_TREATED_17H_UP	184	-0.48506	-2.20456	0	0.001745
GSE11864_UNTREATED_VS_CSF1_PAM3CYS_IN_MAC_UP	167	-0.45132	-2.20333	0	0.001745

GSE23398_WT_VS_IL2_KO_CD4_TCELL_SCURFY_MOUSE_DN	187	-0.46274	-2.1714	0	0.001745
GSE34205_HEALTHY_VS_RSV_INF_INFANT_PBMC_UP	168	-0.6248	-2.16489	0	0.001747
GSE17721_0.5H_VS_4H_PAM3CSK4_BMDC_UP	181	-0.44574	-2.17617	0	0.001751
GSE6269_HEALTHY_VS_STAPH_PNEUMO_INF_PBMC_UP	163	-0.45653	-2.17683	0	0.001751
GSE39110_DAY3_VS_DAY6_POST_IMMUNIZATION_CD8_TCELL_UP	177	-0.53872	-2.20148	0	0.001753
GSE12366_PLASMA_CELL_VS_MEMORY_BCELL_DN	175	-0.49196	-2.16382	0	0.001754
GSE27786_LIN_NEG_VS_NKCELL_DN	178	-0.46988	-2.1734	0	0.001754
GSE5589_IL6_KO_VS_IL10_KO_LPS_AND_IL10_STIM_MACROPHAGE_180MIN_UP	178	-0.47559	-2.18024	0	0.001758
GSE15330_WT_VS_IKAROS_KO_MEGAKARYOCYTE_ERYTHROID_PROGENITOR_DN	182	-0.48036	-2.19897	0	0.001758
GSE360_CTRL_VS_L_DONOVANI_DC_UP	186	-0.47899	-2.16491	0	0.001759
GSE19401_PAM2CSK4_VS_RETINOIC_ACID_AND_PAM2CSK4_STIM_FOLLICULAR_DC_DN	186	-0.51418	-2.175	0	0.001764
GSE29164_UNTREATED_VS_CD8_TCELL_TREATED_MELANOMA_DAY3_DN	187	-0.61691	-2.17714	0	0.001765

GSE7764_IL15_NK_CELL_24H_VS_SPLENOCYTE_DN	175	-0.51683	-2.18027	0	0.001772
GSE7831_CPG_VS_INFLUENZA_STIM_PDC_1H_UP	178	-0.48244	-2.33914	0	0.001777
GSE22611_NOD2_TRANSDUCED_VS_CTRL_HEK293T_STIMULATED_WITH_MDP_2H_UP	170	-0.53039	-2.18085	0	0.001786
GSE18893_TCONV_VS_TREG_2H_CULTURE_UP	169	-0.44511	-2.16236	0	0.001792
GSE17186_MEMORY_VS_CD21HIGH_TRANSITIONAL_BCELL_DN	188	-0.556	-2.1815	0	0.001792
GSE6674_ANTI_IGM_VS_ANTI_IGM_AND_CPG_STIM_BCELL_UP	175	-0.4804	-2.16053	0	0.001804
GSE3982_BCELL_VS_CENT_MEMORY_CD4_TCELL_UP	175	-0.57877	-2.1609	0	0.001805
GSE22886_IGG_IGA_MEMORY_BCELL_VS_BM_PLASMA_CELL_UP	182	-0.4844	-2.18292	0	0.001807
GSE3691_CONVENTIONAL_VS_PLASMACYTOID_DC_SPLEEN_UP	184	-0.51467	-2.13183	0	0.001807
GSE32255_WT_UNSTIM_VS_JMJD2D_KNOCKDOWN_4H_LPS_STIM_DC_UP	165	-0.4773	-2.13042	0	0.00181
GSE2770_IL12_AND_TGFB_ACT_VS_ACT_CD4_TCELL_48H_UP	179	-0.45862	-2.13301	0	0.001812
GSE6566_STRONG_VS_WEAK_DC_STIMULATED_CD4_TCELL_DN	158	-0.53572	-2.18415	0	0.001813

GSE8685_IL2_STARVED_VS_IL2_ACT_IL2_STARVED_CD4_TCELL_UP	177	-0.46698	-2.1319	0	0.001815
GSE28726_ACT_CD4_TCELL_VS_ACT_VA24NEG_NKTCELL_DN	179	-0.46202	-2.13051	0	0.001818
GSE1460_CD4_THYMOCYTE_VS_THYMIC_STROMAL_CELL_UP	179	-0.5464	-2.13209	0	0.001819
GSE36392_MAC_VS_NEUTROPHIL_IL25_TREATED_LUNG_UP	171	-0.47404	-2.13336	0	0.001821
GSE6259_DEC205_POS_DC_VS_CD8_TCELL_UP	167	-0.47103	-2.19247	0	0.001824
GSE20715_0H_VS_48H_OZONE_LUNG_UP	185	-0.59745	-2.15778	0	0.001825
GSE13411_NAIVE_BCELL_VS_PLASMA_CELL_UP	174	-0.54091	-2.13066	0	0.001826
GSE17322_CD103_POS_VS_CD11B_HIGH_LUNG_DC_UP	177	-0.44181	-2.13133	0	0.001826
GSE360_DC_VS_MAC_B_MALAYI_LOW_DOSE_DN	189	-0.47424	-2.18474	0	0.001828
GSE20715_0H_VS_24H_OZONE_LUNG_UP	183	-0.59827	-2.13092	0	0.001829
GSE6259_33D1_POS_DC_VS_CD8_TCELL_DN	118	-0.52939	-2.1335	0	0.001829
GSE26343_UNSTIM_VS_LPS_STIM_MACROPHAGE_DN	180	-0.46953	-2.15876	0	0.001837

GSE24671_CTRL_VS_BAKIMULC_INFECTED_MOUSE_SPLENOCYTES_UP	187	-0.473	-2.13369	0	0.001837
GSE36476_YOUNG_VS_OLD_DONOR_MEMORY_CD4_TCELL_40H_TSST_ACT_DN	181	-0.39625	-2.19044	0	0.001838
GSE22886_TCELL_VS_BCELL_NAIVE_UP	186	-0.56016	-2.12869	0	0.001838
GSE10325_LUPUS_BCELL_VS_LUPUS_MYELOID_UP	171	-0.56326	-2.12797	0	0.001838
GSE411_100MIN_VS_400MIN_IL6_STIM_MACROPHAGE_UP	180	-0.44869	-2.18713	0	0.001839
GSE13229_IMM_VS_INTMATURE_NKCELL_DN	172	-0.4144	-2.18522	0	0.001843
GSE3982_MAC_VS_BASOPHIL_DN	171	-0.54422	-2.19178	0	0.001843
GSE15930_STIM_VS_STIM_AND_IL12_48H_CD8_T_CELL_UP	188	-0.4655	-2.1475	0	0.001844
GSE4984_GALECTIN1_VS_LPS_STIM_DC_UP	151	-0.41569	-2.1337	0	0.001846
GSE45365_NK_CELL_VS_CD11B_DC_MCMV_INFECTION_DN	188	-0.60392	-2.14771	0	0.001848
GSE41867_LCMV_ARMSTRONG_VS_CLONE13_DAY15_EFFECTOR_CD8_TCELL_UP	181	-0.43267	-2.1569	0	0.001854
GSE26030_TH1_VS_TH17_DAY5_POST_POLARIZATION_DN	182	-0.45673	-2.13415	0	0.001854

GSE22886_NAIVE_CD8_TCELL_VS_NKCELL_UP	175	-0.57613	-2.18731	0	0.001855
GSE5589_UNSTIM_VS_45MIN_LPS_STIM_MACROPHAGE_UP	182	-0.42371	-2.18951	0	0.001857
GSE32423_MEMORY_VS_NAIVE_CD8_TCELL_IL7_IL4_DN	175	-0.4508	-2.15531	0	0.001858
GSE10273_HIGH_VS_LOW_IL7_TREATED_IRF4_8_NULL_PRE_BCELL_DN	177	-0.51843	-2.186	0	0.001858
GSE360_T_GONDII_VS_B_MALAYI_LOW_DOSE_MAC_DN	190	-0.42435	-2.13439	0	0.001858
GSE12366_PLASMA_CELL_VS_NAIVE_BCELL_DN	179	-0.5194	-2.14773	0	0.001859
GSE411_UNSTIM_VS_400MIN_IL6_STIM_SOCS3_KO_MACROPHAGE_UP	186	-0.47231	-2.14839	0	0.001859
GSE20727_CTRL_VS_H2O2_TREATED_DC_DN	171	-0.5482	-2.15519	0	0.00186
GSE22886_NAIVE_VS_MEMORY_TCELL_DN	190	-0.48844	-2.15618	0	0.001863
GSE3039_CD4_TCELL_VS_NKT_CELL_UP	180	-0.50567	-2.13504	0	0.001867
GSE11864_CSF1_IFNG_VS_CSF1_IFNG_PAM3CYS_IN_MAC_UP	177	-0.42608	-2.14773	0	0.001869
GSE22886_NAIVE_TCELL_VS_DC_UP	180	-0.58526	-2.1485	0	0.00187

GSE22919_RESTING_VS_IL2_IL12_IL15_STIM_NK_CELL_DN	17<br>5	-<br>0.449<br>13	-<br>2.187<br>39	0	0.0018<br>71
KAECH_DAY8_EFF_VS_DAY15_EFF_CD8_TCELL_DN	18<br>2	-<br>0.524<br>5	-<br>2.135<br>29	0	0.0018<br>71
GOLDRATH_EFF_VS_MEMORY_CD8_TCELL_DN	18<br>9	-<br>0.433<br>7	-<br>2.149<br>28	0	0.0018<br>73
GSE20727_CTRL_VS_H2O2_TREATED_DC_UP	17<br>3	-<br>0.539<br>11	-<br>2.149<br>66	0	0.0018<br>75
GSE12366_GC_VS_MEMORY_BCELL_DN	18<br>3	-<br>0.557<br>85	-<br>2.135<br>38	0	0.0018<br>8
GSE26030_UNSTIM_VS_RESTIM_TH1_DAY5_POST_POLARIZATION_DN	17<br>9	-<br>0.505<br>63	-<br>2.149<br>69	0	0.0018<br>86
GSE38697_LIGHT_ZONE_VS_DARK_ZONE_BCELL_UP	15<br>9	-<br>0.481<br>49	-<br>2.146<br>82	0	0.0018<br>88
GSE17721_PAM3CSK4_VS_CPG_6H_BMDC_DN	19<br>0	-<br>0.501<br>7	-<br>2.123<br>3	0	0.0018<br>9
GSE2770_UNTREATED_VS_TGFB_AND_IL4_TREATED_ACT_CD4_TCELL_48H_DN	19<br>0	-<br>0.511<br>27	-<br>2.125<br>13	0	0.0018<br>92
GSE40274_GATA1_VS_FOXP3_AND_GATA1_TRANSDUCED_ACTIVATED_CD4_TCELL_UP	17<br>9	-<br>0.529<br>32	-<br>2.123<br>63	0	0.0018<br>95
GSE7568_IL4_VS_IL4_AND_TGFB_TREATED_MACROPHAGE_24H_UP	15<br>9	-<br>0.448<br>97	-<br>2.123<br>89	0	0.0018<br>96
GSE25677_MPL_VS_MPL_AND_R848_STIM_BCELL_UP	14<br>8	-<br>0.504<br>77	-<br>2.149<br>72	0	0.0018<br>97

GSE41867_DAY8_EFFECTOR_VS_DAY30_MEMORY_CD8_TCELL_LCMV_ARMSTRONG_DN	181	-0.40426	-2.13729	0	0.001897
GSE16522_ANTI_CD3CD28_STIM_VS_UNSTIM_MEMORY_CD8_TCELL_DN	172	-0.4606	-2.15326	0	0.001897
GSE16385_ROSIGLITAZONE_IL4_VS_IFNG_TNF_STIM_MACROPHAGE_UP	187	-0.56745	-2.125	0	0.001897
GSE33292_DN3_THYMOCYTE_VS_TCF1_KO_TCELL_LYMPHOMA_UP	191	-0.5694	-2.12339	0	0.001898
GSE27859_CD11C_INT_F480_HI_MACROPHAGE_VS_CD11C_ING_F480_INT_DC_UP	161	-0.47049	-2.13559	0	0.001898
GSE339_CD4POS_VS_CD8POS_DC_UP	191	-0.51959	-2.12427	0	0.0019
GSE40274_FOXP3_VS_FOXP3_AND_SATB1_TRANSDUCED_ACTIVATED_CD4_TCELL_DN	164	-0.50394	-2.12521	0	0.0019
GSE10147_IL3_AND_HIVP17_VS_IL3_AND_CPG_STIM_PDC_DN	140	-0.43758	-2.15098	0	0.001901
GSE5099_CLASSICAL_M1_VS_ALTERNATIVE_M2_MACROPHAGE_UP	180	-0.58274	-2.13822	0	0.001902
GSE43955_1H_VS_10H_ACT_CD4_TCELL_UP	187	-0.44809	-2.13584	0	0.001902
GSE12845_IGD_NEG_BLOOD_VS_DARKZONE_GC_TONSIL_BCELL_UP	172	-0.49636	-2.15237	0	0.001905
GSE557_CIITA_KO_VS_I_AB_KO_DC_UP	185	-0.53982	-2.13757	0	0.001906

GSE7852_TREG_VS_TCONV_FAT_DN	186	-0.53905	-2.14991	0	0.001907
GSE22886_NAIVE_CD8_TCELL_VS_MEMORY_TCELL_DN	187	-0.50128	-2.15141	0	0.001908
GSE41867_NAIVE_VS_DAY6_LCMV_EFFECTOR_CD8_TCELL_UP	179	-0.5471	-2.12452	0	0.001908
GSE1112_OT1_VS_HY_CD8AB_THYMOCYTE_RTOC_CULTURE_UP	181	-0.48861	-2.12563	0	0.001908
GSE37416_0H_VS_48H_F_TULARENSIS_LVS_NEUTROPHIL_UP	182	-0.50867	-2.15336	0	0.001908
GSE1460_CORD_VS_ADULT_BLOOD_NAIVE_CD4_TCELL_DN	178	-0.34245	-2.13689	0	0.001911
GSE11864_CSF1_IFNG_VS_CSF1_PAM3CYS_IN_MAC_UP	181	-0.49909	-2.13858	0	0.001911
GSE5503_PLN_DC_VS_SPLEEN_DC_ACTIVATED_ALLOGENIC_TCELL_DN	181	-0.39253	-2.12617	0	0.001911
GSE360_DC_VS_MAC_UP	186	-0.4327	-2.15116	0	0.001913
GSE13522_CTRL_VS_T_CRUZI_Y_STRAIN_INF_SKIN_IFNAR_KO_UP	181	-0.60755	-2.14034	0	0.001913
GSE11864_UNTREATED_VS_CSF1_IFNG_IN_MAC_UP	166	-0.46282	-2.1501	0	0.001913
GSE22886_IGM_MEMORY_BCELL_VS_BM_PLASMA_CELL_UP	176	-0.52056	-2.13973	0	0.001913

GSE3565_CTRL_VS_LPS_INJECTED_SPLENOCYTES_DN	161	-0.52052	-2.13911	0	0.001915
GSE19941_IL10_KO_VS_IL10_KO_AND_NFKBP50_KO_LPS_STIM_MACROPHAGE_DN	185	-0.48242	-2.15258	0	0.001917
GSE43863_TH1_VS_LY6C_LOW_CXCR5NEG_EFFECTOR_CD4_TCELL_DN	189	-0.37457	-2.14081	0	0.001917
GSE22886_CD4_TCELL_VS_BCELL_NAIVE_UP	186	-0.60064	-2.15145	0	0.001919
GSE3691_IFN_PRODUCING_KILLER_DC_VS_PLASMACYTOID_DC_SPLEEN_UP	183	-0.4508	-2.12173	0	0.001924
GSE45739_UNSTIM_VS_ACD3_ACD28_STIM_NRAS_KO_CD4_TCELL_UP	182	-0.54806	-2.12067	0	0.001926
GSE27291_0H_VS_7D_STIM_GAMMADELTA_TCELL_DN	164	-0.50633	-2.12146	0	0.001926
GSE17721_0.5H_VS_12H_POLYIC_BMDC_DN	187	-0.453	-2.14127	0	0.001927
GSE4984_UNTREATED_VS_GALECTIN1_TREATED_DC_DN	157	-0.70512	-2.11796	0	0.001927
GSE2770_TGFB_AND_IL4_VS_TGFB_AND_IL12_TREATED_ACT_CD4_TCELL_6H_DN	186	-0.47811	-2.14084	0	0.001927
GSE22886_NAIVE_CD4_TCELL_VS_DC_UP	175	-0.62589	-2.11858	0	0.001928
GSE8685_IL2_ACT_IL2_STARVED_VS_IL15_ACT_IL2_STARVED_CD4_TCELL_UP	182	-0.4801	-2.12128	0	0.001929

GSE17721_PAM3CSK4_VS_GADIQUIMOD_4H_BMDC_DN	184	-0.53573	-2.12191	0	0.001932
GSE3039_NKT_CELL_VS_B1_BCELL_UP	175	-0.5191	-2.11861	0	0.001935
GSE10239_NAIVE_VS_KLRG1HIGH_EFF_CD8_TCELL_UP	184	-0.46768	-2.14204	0	0.001936
GSE26030_UNSTIM_VS_RESTIM_TH1_DAY15_POST_POLARIZATION_DN	187	-0.48419	-2.14314	0	0.00194
GSE30971_CTRL_VS_LPS_STIM_MACROPHAGE_WBP7_HET_4H_DN	176	-0.48359	-2.11895	0	0.001941
GSE20715_0H_VS_6H_OZONE_LUNG_UP	183	-0.50466	-2.11868	0	0.001943
GSE22432_UNTREATED_VS_TGFB1_TREATED_COMMON_DC_PROGENITOR_DN	173	-0.48637	-2.14483	0	0.001943
GSE2770_IL4_ACT_VS_ACT_CD4_TCELL_6H_UP	167	-0.53846	-2.12001	0	0.001944
GSE5679_PPARG_LIGAND_ROSIGLITAZONE_VS_RARA_AGONIST_AM580_TREATED_DC_DN	184	-0.59146	-2.14436	0	0.001944
GSE6269_HEALTHY_VS_E_COLI_INF_PBMC_UP	159	-0.53222	-2.14209	0	0.001946
GSE27786_NKCELL_VS_MONO_MAC_UP	176	-0.3654	-2.1191	0	0.001949
GSE46606_DAY1_VS_DAY3_CD40L_IL2_IL5_STIMULATED_IRF4MID_BCELL_DN	181	-0.42086	-2.1426	0	0.00195

GSE12003_MIR223_KO_VS_WT_BM_PROGENITOR_4D_CULTURE_UP	180	-0.48493	-2.1451	0	0.001953
GSE17186_NAIVE_VS_CD21LOW_TRANSITIONAL_BCELL_CORD_BLOOD_UP	182	-0.47296	-2.14336	0	0.001955
GSE22886_IGA_VS_IGM_MEMORY_BCELL_DN	179	-0.53105	-2.11925	0	0.001957
GSE40274_FOXP3_VS_FOXP3_AND_PBX1_TRANSDUCED_ACTIVATED_CD4_TCELL_UP	176	-0.62577	-2.11916	0	0.001957
GSE17721_0.5H_VS_8H_LPS_BMDC_DN	184	-0.48367	-2.11153	0	0.001963
GSE369_PRE_VS_POST_IL6_INJECTION_IFNG_WT_LIVER_DN	187	-0.50153	-2.14353	0	0.001965
GSE34392_ST2_KO_VS_WT_DAY8_LCMV_EFFECTOR_CD8_TCELL_UP	185	-0.4295	-2.11508	0	0.001967
GSE15330_HSC_VS_GRANULOCYTE_MONOCYTE_PROGENITOR_DN	173	-0.3959	-2.11215	0	0.00197
GSE3982_DC_VS_CENT_MEMORY_CD4_TCELL_DN	180	-0.57104	-2.1123	0	0.001978
GSE1448_CTRL_VS_ANTI_VBETA5_DP_THYMOCYTE_UP	183	-0.44027	-2.11337	0	0.001982
GSE43863_TH1_VS_TFH_EFFECTOR_CD4_TCELL_DN	176	-0.49735	-2.11559	0	0.001982
GSE17974_IL4_AND_ANTI_IL12_VS_UNTREATED_4H_ACT_CD4_TCELL_UP	160	-0.50588	-2.11519	0	0.001982

GSE3982_BASOPHIL_VS_TH1_UP	179	-0.51942	-2.11269	0	0.001985
GSE26156_DOUBLE_POSITIVE_VS_CD4_SINGLE_POSITIVE_THYMOCYTE_UP	179	-0.49208	-2.11367	0	0.001989
GSE7568_IL4_TGFB_DEXAMETHASONE_VS_IL4_TGFB_TREATED_MACROPHAGE_DN	165	-0.47252	-2.11584	0	0.001989
GSE6269_HEALTHY_VS_FLU_INF_PBMC_UP	149	-0.46716	-2.11073	0	0.001992
GSE25677_R848_VS_MPL_AND_R848_STIM_BCELL_DN	155	-0.43016	-2.11277	0	0.001993
GSE13547_WT_VS_ZFX_KO_BCELL_UP	156	-0.60826	-2.11094	0	0.001998
GSE39820_CTRL_VS_IL1B_IL6_IL23A_CD4_TCELL_DN	189	-0.53622	-2.11079	0	0.001999
GSE45365_NK_CELL_VS_CD8_TCELL_MCMV_INFECTION_UP	180	-0.46833	-2.10885	0	0.002053
GSE26669_CTRL_VS_COSTIM_BLOCK_MLR_CD8_TCELL_DN	183	-0.52781	-2.10925	0	0.002053
GSE17186_CD21LOW_VS_CD21HIGH_TRANSITIONAL_BCELL_CORD_BLOOD_DN	176	-0.42174	-2.10789	0	0.002053
GSE46606_IRF4_KO_VS_WT_CD40L_IL2_IL5_1DAY_STIMULATED_BCELL_UP	173	-0.55952	-2.1089	0	0.00206
KAECH_NAIVE_VS_MEMORY_CD8_TCELL_UP	182	-0.46312	-2.10799	0	0.002061

GSE7852_TREG_VS_TCONV_DN	178	-0.59355	-2.10634	0	0.002062
GSE35435_RESTING_VS_IL4_TREATED_MACROPHAGE_UP	179	-0.4888	-2.10711	0	0.002062
GSE5542_UNTREATED_VS_IFNA_TREATED_EPITHELIAL_CELLS_24H_UP	187	-0.54763	-2.10551	0	0.002083
GSE2770_TGFB_AND_IL4_VS_IL12_TREATED_ACT_CD4_TCELL_6H_UP	189	-0.54132	-2.10506	0	0.00212
GSE41867_MEMORY_VS_EXHAUSTED_CD8_TCELL_DAY30_LCMV_UP	181	-0.63252	-2.10443	0	0.002149
GSE43863_NAIVE_VS_MEMORY_LY6C_INT_CXCR5POS_CD4_TCELL_D150_LCMV_UP	179	-0.5826	-2.10348	0	0.002151
GSE9988_LOW_LPS_VS_CTRL_TREATED_MONOCYTE_DN	182	-0.40083	-2.10364	0	0.002154
GSE26343_WT_VS_NFAT5_KO_MACROPHAGE_LPS_STIM_DN	178	-0.50417	-2.1043	0	0.002156
GSE6092_IFNG_VS_IFNG_AND_B_BURGDORFERI_INF_ENDOTHELIAL_CELL_UP	173	-0.5395	-2.10403	0	0.002157
GSE29618_MONOCYTE_VS_MDC_DN	171	-0.58136	-2.10149	0	0.002161
GSE28726_ACT_CD4_TCELL_VS_ACT_NKTCELL_DN	176	-0.44781	-2.10187	0	0.002163
GSE411_100MIN_VS_400MIN_IL6_STIM_SOCS3_KO_MACROPHAGE_DN	180	-0.54547	-2.40836	0	0.002164

GSE1791_CTRL_VS_NEUROMEDINU_IN_T_CELL_LINE_12H_UP	175	-0.37072	-2.10248	0	0.00217
GSE27241_CTRL_VS_DIGOXIN_TREATED_CD4_TCELL_IN_TH17_POLARIZING_CONDITIONS_DN	179	-0.56161	-2.10204	0	0.00217
GSE6092_UNSTIM_VS_IFNG_STIM_ENDOTHELIAL_CELL_DN	179	-0.52037	-2.10281	0	0.002178
GSE15330_HSC_VS_LYMPHOID_PRIMED_MULTIPOTENT_PROGENITOR_IKAROS_KO_DN	178	-0.41245	-2.10054	0	0.002179
GSE2128_CTRL_VS_MIMETOPE_NEGATIVE_SELECTION_DP_THYMOCYTE_NOD_DN	163	-0.52551	-2.1003	0	0.00218
GSE12392_WT_VS_IFNB_KO_CD8A_POS_SPLEEN_DC_UP	176	-0.38392	-2.10304	0	0.002184
GSE557_WT_VS_CIITA_KO_DC_DN	182	-0.47709	-2.10284	0	0.002185
GSE7831_UNSTIM_VS_CPG_STIM_PDC_1H_DN	187	-0.47252	-2.09948	0	0.002194
GSE5542_UNTREATED_VS_IFNG_TREATED_EPITHELIAL_CELLS_6H_DN	191	-0.54893	-2.09934	0	0.0022
GSE369_PRE_VS_POST_IL6_INJECTION_IFNG_KO_LIVER_UP	179	-0.49891	-2.0981	0	0.002202
GSE360_T_GONDII_VS_B_MALAYI_LOW_DOSE_DC_DN	191	-0.59886	-2.09854	0	0.002204
GSE24210_CTRL_VS_IL35_TREATED_TCONV_CD4_TCELL_UP	182	-0.54438	-2.09909	0	0.002205

GSE360_DC_VS_MAC_L_DONOVANI_DN	191	-0.44568	-2.09868	0	0.002207
GSE13738_TCR_VS_BYSTANDER_ACTIVATED_CD4_TCELL_DN	176	-0.55117	-2.0983	0	0.002209
GSE16697_CD4_TCELL_VS_TFH_CD4_TCELL_UP	181	-0.50912	-2.09704	0	0.002216
GSE22886_NAIVE_CD8_TCELL_VS_NEUTROPHIL_UP	172	-0.47668	-2.44841	0	0.002218
GSE13547_2H_VS_12_H_ANTI_IGM_STIM_ZFX_KO_BCELL_UP	152	-0.60187	-2.09707	0	0.002223
GSE26030_UNSTIM_VS_RESTIM_TH17_DAY5_POST_POLARIZATION_UP	168	-0.50781	-2.09696	0	0.002224
GSE14415_INDUCED_VS_NATURAL_TREG_UP	143	-0.50755	-2.09641	0	0.002244
GSE23568_ID3_KO_VS_WT_CD8_TCELL_DN	190	-0.62531	-2.09635	0	0.002252
GSE28737_FOLLICULAR_VS_MARGINAL_ZONE_BCELL_BCL6_HET_UP	173	-0.50168	-2.09348	0	0.002294
GSE9037_CTRL_VS_LPS_4H_STIM_BMDM_UP	184	-0.43661	-2.09306	0	0.002294
GSE3982_BASOPHIL_VS_TH2_UP	181	-0.53699	-2.09409	0	0.002295
GSE9037_CTRL_VS_LPS_1H_STIM_IRAK4_KO_BMDM_UP	176	-0.41077	-2.09256	0	0.0023

GSE3565_DUSP1_VS_WT_SPLENOCYTES_DN	172	-0.50373	-2.09349	0	0.002302
GSE37416_0H_VS_3H_F_TULARENSIS_LVS_NEUTROPHIL_UP	169	-0.37957	-2.09431	0	0.002302
GSE27786_LSK_VS_CD4_TCELL_DN	173	-0.48972	-2.09449	0	0.002306
GSE27859_MACROPHAGE_VS_CD11C_INT_F480_INT_DC_DN	181	-0.54836	-2.09277	0	0.002307
GSE3039_ALPHABETA_CD8_TCELL_VS_B1_BCELL_UP	179	-0.49484	-2.09221	0	0.002319
GSE27241_CTRL_VS_DIGOXIN_TREATED_CD4_TCELL_IN_TH17_POLARIZING_CONDITIONS_UP	149	-0.599	-2.09057	0	0.00232
GSE7764_NKCELL_VS_SPLENOCYTE_DN	190	-0.53613	-2.09198	0	0.002322
GSE5679_PPARG_LIGAND_ROSIGLITAZONE_VS_ROSIGLITAZONE_AND_RARA_AGONIST_AM580_TREATED_DC_UP	185	-0.51649	-2.09086	0	0.002324
GSE3039_ALPHABETA_CD8_TCELL_VS_B2_BCELL_UP	182	-0.54884	-2.09113	0	0.002328
GSE37301_COMMON_LYMPHOID_PROGENITOR_VS_CD4_TCELL_DN	182	-0.40965	-2.09099	0	0.002331
GSE9601_NFKB_INHIBITOR_VS_PI3K_INHIBITOR_TREATED_HCMV_INF_MONOCYTE_UP	182	-0.3815	-2.09115	0	0.002335
GSE17721_0.5H_VS_24H_POLYIC_BMDC_DN	188	-0.41805	-2.09128	0	0.002342

GSE12845_PRE_GC_VS_DARKZONE_GC_TONSIL_BCELL_UP	177	-0.61318	-2.08871	0	0.002356
GSE17721_LPS_VS_POLYIC_24H_BMDC_DN	182	-0.51541	-2.08888	0	0.002359
GSE5503_LIVER_DC_VS_SPLEEN_DC_ACTIVATED_ALLOGENIC_TCELL_DN	176	-0.39923	-2.08852	0	0.00236
GSE17301_IFNA2_VS_IFNA5_STIM_ACD3_ACD28_ACT_CD8_TCELL_UP	178	-0.48845	-2.0894	0	0.00236
GSE17721_POLYIC_VS_CPG_16H_BMDC_UP	190	-0.46412	-2.08944	0	0.002361
GSE12198_LOW_IL2_STIM_NK_CELL_VS_HIGH_IL2_STIM_NK_CELL_UP	183	-0.35819	-2.08962	0	0.002368
GSE10239_NAIVE_VS_DAY4.5_EFF_CD8_TCELL_UP	181	-0.47442	-2.08449	0	0.002387
GSE11961_FOLLICULAR_BCELL_VS_GERMINAL_CENTER_BCELL_DAY40_UP	184	-0.48567	-2.08449	0	0.002394
GSE23568_CTRL_TRANSDUCED_VS_WT_CD8_TCELL_UP	191	-0.55279	-2.08592	0	0.002397
GSE1448_ANTI_VALPHA2_VS_VBETA5_DP_THYMOCYTE_UP	184	-0.43603	-2.08694	0	0.0024
GSE5589_WT_VS_IL10_KO_LPS_STIM_MACROPHAGE_180MIN_UP	149	-0.44057	-2.086	0	0.002401
GSE2706_LPS_VS_R848_AND_LPS_8H_STIM_DC_UP	170	-0.47862	-2.08506	0	0.002401

GSE25123_ROSIGLITAZONE_VS_IL4_AND_ROSIGLITAZONE_STIM_PPARG_KO_MACROPHAGE_DAY10_DN	176	-0.49577	-2.08532	0	0.002405
KAECH_DAY15_EFF_VS_MEMORY_CD8_TCELL_UP	188	-0.59481	-2.08554	0	0.002406
GSE3982_MAST_CELL_VS_BCELL_DN	171	-0.55614	-2.08695	0	0.002408
GSE20715_WT_VS_TLR4_KO_6H_OZONE_LUNG_UP	183	-0.4982	-2.08608	0	0.002408
GSE21927_SPLEEN_MONOCYTE_VS_GMCSF_GCSF_BONE_MARROW_DN	174	-0.49057	-2.08633	0	0.002411
GSE3039_CD4_TCELL_VS_ALPHAALPHA_CD8_TCELL_UP	178	-0.51454	-2.08292	0	0.002427
GSE17301_IFNA2_VS_IFNA2_AND_ACD3_ACD28_STIM_CD8_TCELL_UP	185	-0.45918	-2.08298	0	0.002431
GSE10325_BCELL_VS_MYELOID_UP	166	-0.65815	-2.08187	0	0.002438
GSE3982_BCELL_VS_TH2_UP	175	-0.57221	-2.08255	0	0.002441
GSE29164_UNTREATED_VS_CD8_TCELL_TREATED_MELANOMA_DAY7_UP	189	-0.56374	-2.08139	0	0.002444
GSE21380_NON_TFH_VS_TFH_CD4_TCELL_DN	187	-0.52684	-2.07794	0	0.002448
GSE41867_NAIVE_VS_DAY8_LCMV_EFFECTOR_CD8_TCELL_DN	181	-0.45578	-2.08271	0	0.002448

GSE17721_0.5H_VS_12H_LPS_BMDC_DN	186	-0.4437	-2.07832	0	0.00245
GSE8921_UNSTIM_VS_TLR1_2_STIM_MONOCYTE_24H_UP	179	-0.59641	-2.07862	0	0.002452
GSE13547_WT_VS_ZFX_KO_BCELL_ANTI_IGM_STIM_2H_DN	153	-0.46603	-2.0781	0	0.002452
GSE24142_EARLY_THYMIC_PROGENITOR_VS_DN3_THYMOCYTE_DN	184	-0.58513	-2.07874	0	0.002456
GSE411_UNSTIM_VS_100MIN_IL6_STIM_SOCS3_KO_MACROPHAGE_UP	180	-0.43516	-2.07888	0	0.002459
GSE22886_NAIVE_CD4_TCELL_VS_48H_ACT_TH1_UP	175	-0.46804	-2.07975	0	0.002459
GSE41176_UNSTIM_VS_ANTI_IGM_STIM_BCELL_3H_DN	171	-0.4278	-2.07177	0	0.002462
GSE36888_UNTREATED_VS_IL2_TREATED_TCELL_2H_UP	173	-0.49425	-2.07635	0	0.002462
GSE3982_CENT_MEMORY_CD4_TCELL_VS_NKCELL_DN	182	-0.54696	-2.07979	0	0.002464
GSE46606_DAY1_VS_DAY3_CD40L_IL2_IL5_STIMULATED_IRF4HIGH_BCELL_DN	180	-0.52213	-2.07897	0	0.002466
GSE24142_EARLY_THYMIC_PROGENITOR_VS_DN3_THYMOCYTE_FETAL_DN	188	-0.53088	-2.07586	0	0.002466
GSE28726_NAIVE_VS_ACTIVATED_CD4_TCELL_UP	183	-0.53272	-2.06956	0	0.002467

GSE40274_FOXP3_VS_FOXP3_AND_IRF4_TRANSDUCED_ACTIVATED_CD4_TCELL_DN	174	-0.59494	-2.07602	0	0.002467
GSE14308_TH1_VS_TH17_DN	180	-0.38256	-2.07613	0	0.002467
GSE4535_BM_DERIVED_DC_VS_FOLLICULAR_DC_UP	180	-0.59016	-2.0791	0	0.002468
GSE40274_FOXP3_VS_FOXP3_AND_LEF1_TRANSDUCED_ACTIVATED_CD4_TCELL_UP	176	-0.51972	-2.07417	0	0.002468
GSE15624_3H_VS_6H_HALOFUGINONE_TREATED_CD4_TCELL_UP	158	-0.51654	-2.07189	0	0.002469
GSE13229_IMM_VS_MATURE_NKCELL_DN	177	-0.48653	-2.07948	0	0.002469
GSE8921_UNSTIM_0H_VS_TLR1_2_STIM_MONOCYTE_24H_DN	185	-0.42785	-2.07687	0	0.002469
GSE44649_NAIVE_VS_ACTIVATED_CD8_TCELL_UP	185	-0.41694	-2.07111	0	0.002472
GSE2706_2H_VS_8H_R848_AND_LPS_STIM_DC_UP	176	-0.47191	-2.07236	0	0.002472
GSE2128_CTRL_VS_MIMETOPE_NEGATIVE_SELECTION_DP_THYMOCYTE_C57BL6_DN	185	-0.43588	-2.0696	0	0.002473
GSE23505_IL6_IL1_IL23_VS_IL6_IL1_TGFB_TREATED_CD4_TCELL_UP	185	-0.52202	-2.06919	0	0.002474
GSE9988_ANTI_TREM1_VS_CTRL_TREATED_MONOCYTES_DN	175	-0.51989	-2.08009	0	0.002475

GSE22886_NAIVE_BCELL_VS_DC_UP	171	-0.64237	-2.07198	0	0.002475
GSE22935_UNSTIM_VS_12H_MBOVIS_BCG_STIM_MACROPHAGE_UP	180	-0.33213	-2.07419	0	0.002475
GSE37532_WT_VS_PPARG_KO_VISCERAL_ADIPOSE_TISSUE_TCONV_UP	176	-0.59231	-2.07135	0	0.002476
GSE2770_TGFB_AND_IL4_VS_TGFB_AND_IL12_TREATED_ACT_CD4_TCELL_48H_DN	171	-0.48397	-2.06826	0	0.002477
GSE20198_IL12_VS_IL12_IL18_TREATED_ACT_CD4_TCELL_DN	189	-0.39194	-2.0743	0	0.002478
GSE17186_NAIVE_VS_CD21HIGH_TRANSITIONAL_BCELL_CORD_BLOOD_UP	177	-0.38665	-2.07245	0	0.002479
GSE21033_CTRL_VS_POLYIC_STIM_DC_12H_UP	149	-0.58108	-2.07158	0	0.002479
GSE37533_UNTREATED_VS_PIOGLIZATONE_TREATED_CD4_TCELL_PPARG1_AND_FOXP3_TRASDUCED_DN	189	-0.48654	-2.08034	0	0.002479
GSE45739_NRAS_KO_VS_WT_UNSTIM_CD4_TCELL_DN	175	-0.48457	-2.06967	0	0.002479
GSE36826_WT_VS_IL1R_KO_SKIN_UP	187	-0.60081	-2.07446	0	0.002481
GSE27786_LSK_VS_CD8_TCELL_DN	180	-0.43788	-2.07202	0	0.002481
GSE36476_YOUNG_VS_OLD_DONOR_MEMORY_CD4_TCELL_72H_TSST_ACT_DN	171	-0.56831	-2.07422	0	0.002482

GSE3982_DC_VS_MAC_UP	175	-0.51588	-2.06833	0	0.002483
GSE41176_UNSTIM_VS_ANTI_IGM_STIM_TAK1_KO_BCELL_24H_UP	185	-0.4683	-2.06816	0	0.002483
GSE17721_POLYIC_VS_GARDIQUIMOD_16H_BMDC_UP	182	-0.44946	-2.07307	0	0.002484
GSE45881_CXCR6HI_VS_CXCR1LO_COLONIC_LAMINA_PROPRIA_DN	186	-0.55536	-2.06778	0	0.002485
GSE14350_TREG_VS_TEFF_UP	177	-0.45281	-2.07459	0	0.002485
GSE43863_DAY6_EFF_VS_DAY150_MEM_TFH_CD4_TCELL_UP	189	-0.52426	-2.0727	0	0.002485
GSE32901_NAIVE_VS_TH17_ENRICHED_CD4_TCELL_DN	163	-0.46242	-2.06974	0	0.002485
GSE8921_UNSTIM_0H_VS_TLR1_2_STIM_MONOCYTE_12H_DN	183	-0.47603	-2.06858	0	0.002486
GSE22886_NAIVE_CD4_TCELL_VS_12H_ACT_TH1_UP	183	-0.50631	-2.07425	0	0.002488
GSE32164_RESTING_DIFFERENTIATED_VS_ALTERNATIVELY_ACT_M2_MACROPHAGE_DN	190	-0.55362	-2.06786	0	0.002489
GSE40225_WT_VS_RIP_B7X_DIABETIC_MOUSE_PANCREATIC_CD8_TCELL_UP	171	-0.52681	-2.07309	0	0.00249
GSE40274_LEF1_VS_FOXP3_AND_LEF1_TRANSDUCED_ACTIVATED_CD4_TCELL_DN	174	-0.53506	-2.07278	0	0.002491

GSE40666_NAIVE_VS_EFFECTOR_CD8_TCELL_WITH_IFNA_STIM_90MIN_UP	184	-0.53025	-2.06982	0	0.002492
GSE4748_CYANOBACTERIUM_LPSLIKE_VS_LPS_AND_CYANOBACTERIUM_LPSLIKE_STIM_DC_3H_UP	162	-0.65974	-2.07357	0	0.002492
GSE27859_CD11C_INT_F480_HI_MACROPHAGE_VS_CD11C_ING_F480_INT_DC_DN	186	-0.44431	-2.07323	0	0.002492
GSE37532_TREG_VS_TCONV_PPARG_KO_CD4_TCELL_FROM_VISCERAL_ADIPOSE_TISSUE_DN	181	-0.51931	-2.06982	0	0.002498
GSE17721_PAM3CSK4_VS_GADIQUIMOD_1H_BMDC_DN	178	-0.37021	-2.06717	0	0.002498
GSE339_CD4POS_VS_CD4CD8DN_DC_UP	186	-0.47885	-2.07327	0	0.002499
GSE40666_WT_VS_STAT4_KO_CD8_TCELL_WITH_IFNA_STIM_90MIN_UP	178	-0.53949	-2.07336	0	0.002499
GSE22432_MULTIPOTENT_PROGENITOR_VS_PDC_UP	184	-0.4831	-2.07367	0	0.002499
GSE22886_NAIVE_VS_IGG_IGA_MEMORY_BCELL_DN	179	-0.523	-2.07051	0	0.002502
GSE10240_IL22_VS_IL22_AND_IL17_STIM_PRIMARY_BRONCHIAL_EPITHELIAL_CELLS_UP	185	-0.49101	-2.06695	0	0.002504
GSE14000_UNSTIM_VS_16H_LPS_DC_TRANSLATED_RNA_UP	176	-0.43072	-2.06989	0	0.002504
GSE5542_UNTREATED_VS_IFNA_TREATED_EPITHELIAL_CELLS_24H_DN	172	-0.47526	-2.0639	0	0.002505

GSE22886_UNSTIM_VS_STIM_MEMORY_TCELL_UP	179	-0.51199	-2.06359	0	0.002507
GSE10239_NAIVE_VS_KLRG1INT_EFF_CD8_TCELL_UP	177	-0.45324	-2.06476	0	0.002507
GSE32034_LY6C_HIGH_VS_LOW_ROSIGLIZATONE_TREATED_MONOCYTE_UP	187	-0.44816	-2.06399	0	0.00251
GSE27786_NEUTROPHIL_VS_MONO_MAC_UP	170	-0.46382	-2.06312	0	0.002512
GSE8685_IL2_STARVED_VS_IL15_ACT_IL2_STARVED_CD4_TCELL_UP	176	-0.39685	-2.06477	0	0.002513
GSE37301_PRO_BCELL_VS_GRANULOCYTE_MONOCYTE_PROGENITOR_DN	166	-0.53187	-2.06279	0	0.002515
GSE32423_CTRL_VS_IL7_IL4_MEMORY_CD8_TCELL_UP	180	-0.48101	-2.06405	0	0.002519
GSE33162_UNTREATED_VS_4H_LPS_STIM_HDAC3_HET_MACROPHAGE_DN	182	-0.4921	-2.06478	0	0.002519
GSE22432_PDC_VS_TGFB1_TREATEDCOMMON_DC_PROGENITOR_DN	182	-0.51573	-2.06492	0	0.002523
GSE360_T_GONDII_VS_B_MALAYI_HIGH_DOSE_MAC_DN	187	-0.54681	-2.06413	0	0.002525
GSE37605_C57BL6_VS_NOD_FOXP3_IRES_GFP_TREG_DN	182	-0.5093	-2.06595	0	0.002526
GSE21927_SPLENIC_VS_TUMOR_MONOCYTES_FROM_C26GM_TUMOROUS_MICE_BALBC_DN	184	-0.45901	-2.06123	0	0.002528

GSE1460_DP_VS_CD4_THYMOCYTE_DN	189	-0.59866	-2.06603	0	0.00253
GSE40274_CTRL_VS_SATB1_TRANSDUCED_ACTIVATED_CD4_TCELL_UP	153	-0.53517	-2.06544	0	0.002533
GSE27786_BCELL_VS_NKCELL_DN	181	-0.4502	-2.06553	0	0.002537
GSE23984_CTRL_VS_HYPOCALEMIC_VITAMIND_ANALOG_TCELL_DN	177	-0.50056	-2.06564	0	0.00254
GSE2770_UNTREATED_VS_ACT_CD4_TCELL_2H_UP	181	-0.52506	-2.0603	0	0.00256
GSE4748_LPS_VS_LPS_AND_CYANOBACTERIUM_LPSLIKE_STIM_DC_3H_UP	168	-0.55037	-2.06035	0	0.002564
GSE15735_CTRL_VS_HDAC_INHIBITOR_TREATED_CD4_TCELL_12H_DN	177	-0.54216	-2.05955	0	0.00259
GSE17721_LPS_VS_PAM3CSK4_24H_BMDC_UP	181	-0.43698	-2.05973	0	0.002591
GSE40274_HELIOS_VS_FOXP3_AND_HELIOS_TRANSDUCED_ACTIVATED_CD4_TCELL_DN	179	-0.48694	-2.05999	0	0.002593
GSE36078_WT_VS_IL1R_KO_LUNG_DC_DN	178	-0.52765	-2.05957	0	0.002596
GSE10325_CD4_TCELL_VS_MYELOID_UP	183	-0.63808	-2.05217	0	0.002597
GSE5679_RARA_AGONIST_AM580_VS_AM580_AND_ROSIGLITAZONE_TREATED_DC_UP	179	-0.49357	-2.05227	0	0.002598

GSE27786_LSK_VS_BCELL_DN	178	-0.39468	-2.05245	0	0.002599
GSE17721_CTRL_VS_PAM3CSK4_24H_BMDC_UP	184	-0.40126	-2.05309	0	0.002603
GSE8868_SPLEEN_VS_INTESTINE_CD11B_POS_CD11C_NEG_DC_UP	173	-0.43091	-2.05258	0	0.002604
GSE29164_DAY3_VS_DAY7_UNTREATED_MELANOMA_UP	168	-0.55308	-2.0534	0	0.002607
GSE40274_CTRL_VS_LEF1_TRANSDUCED_ACTIVATED_CD4_TCELL_UP	164	-0.54931	-2.05329	0	0.002609
GSE6875_WT_VS_FOXP3_KO_TREG_UP	175	-0.45423	-2.05262	0	0.002609
GSE3982_BCELL_VS_BASOPHIL_UP	166	-0.45378	-2.05288	0	0.00261
GSE7219_WT_VS_NIK_NFKB2_KO_DC_DN	161	-0.6043	-2.05164	0	0.002611
GSE21927_SPLEEN_C57BL6_VS_EL4_TUMOR_BALBC_MONOCYTES_UP	179	-0.478	-2.05341	0	0.002613
GSE7831_UNSTIM_VS_CPG_STIM_PDC_4H_DN	186	-0.48647	-2.05348	0	0.002614
GSE13306_RA_VS_UNTREATED_MEM_CD4_TCELL_UP	182	-0.59323	-2.05135	0	0.002614
GSE22886_CD8_TCELL_VS_BCELL_NAIVE_UP	187	-0.59536	-2.0587	0	0.002616

GSE44732_UNSTIM_VS_IL27_STIM_IMATURE_DC_UP	177	-0.48943	-2.05352	0	0.002616
GSE5542_UNTREATED_VS_IFNA_TREATED_EPITHELIAL_CELLS_6H_UP	175	-0.62515	-2.04993	0	0.002617
GSE5542_UNTREATED_VS_IFNG_TREATED_EPITHELIAL_CELLS_6H_UP	174	-0.50315	-2.05011	0	0.00262
GSE16522_MEMORY_VS_NAIVE_ANTI_CD3CD28_STIM_CD8_TCELL_UP	178	-0.43643	-2.05357	0	0.002621
GSE27786_CD8_TCELL_VS_MONO_MAC_UP	179	-0.34899	-2.05375	0	0.002622
GSE23321_CD8_STEM_CELL_MEMORY_VS_EFFECTOR_MEMORY_CD8_TCELL_UP	188	-0.45497	-2.05817	0	0.002626
GSE17721_4H_VS_24H_POLYIC_BMDC_DN	175	-0.38499	-2.05017	0	0.002626
KAECH_NAIVE_VS_DAY15_EFF_CD8_TCELL_UP	184	-0.39666	-2.05384	0	0.002628
GSE8621_UNSTIM_VS_LPS_PRIMED_AND_LPS_STIM_MACROPHAGE_DN	182	-0.5367	-2.05043	0	0.002628
GSE3039_ALPHAALPHA_CD8_TCELL_VS_B2_BCELL_UP	186	-0.5955	-2.05805	0	0.002629
GSE17301_ACD3_ACD28_VS_ACD3_ACD28_AND_IFNA5_STIM_CD8_TCELL_DN	185	-0.57772	-2.05837	0	0.002633
GSE17721_12H_VS_24H_PAM3CSK4_BMDC_DN	189	-0.46158	-2.05404	0	0.002634

GSE26030_UNSTIM_VS_RESTIM_TH17_DAY5_POST_POLARIZATION_DN	179	-0.55048	-2.05755	0	0.002634
GSE26488_WT_VS_VP16_TRANSGENIC_HDAC7_KO_DOUBLE_POSITIVE_THYMOCYTE_DN	157	-0.5538	-2.05708	0	0.002639
GSE41867_LCMV_ARMSTRONG_VS_CLONE13_DAY8_EFFECTOR_CD8_TCELL_UP	180	-0.40114	-2.05405	0	0.002639
GSE14308_TH2_VS_TH17_DN	177	-0.43037	-2.04926	0	0.002641
GSE16385_IFNG_TNF_VS_IL4_STIM_MACROPHAGE_UP	179	-0.4871	-2.04718	0	0.002641
GSE6674_PL2_3_VS_ANTI_IGM_AND_CPG_STIM_BCELL_UP	183	-0.46403	-2.04837	0	0.002641
GSE9650_NAIVE_VS_MEMORY_CD8_TCELL_UP	182	-0.46194	-2.0541	0	0.002645
GSE41867_DAY6_EFFECTOR_VS_DAY30_MEMORY_CD8_TCELL_LCMV_ARMSTRONG_DN	184	-0.45279	-2.04721	0	0.002646
GSE32423_IL7_VS_IL7_IL4_MEMORY_CD8_TCELL_UP	182	-0.42273	-2.0475	0	0.002646
GSE23505_UNTREATED_VS_4DAY_IL6_IL1_IL23_TREATED_CD4_TCELL_DN	180	-0.61593	-2.04885	0	0.002648
GSE17812_WT_VS_THPOK_KO_MEMORY_CD8_TCELL_UP	181	-0.49631	-2.05465	0	0.002649
GSE19198_CTRL_VS_IL21_TREATED_TCELL_24H_DN	185	-0.48881	-2.04795	0	0.002649

GSE40274_CTRL_VS_GATA1_TRANSDUCED_ACTIVATED_CD4_TCELL_UP	123	-0.60396	-2.05412	0	0.002651
GSE46606_IRF4HIGH_VS_IRF4MID_CD40L_IL2_IL5_DAY3_STIMULATED_BCELL_DN	181	-0.50708	-2.04761	0	0.002652
GSE17721_POLYIC_VS_CPG_6H_BMDC_DN	175	-0.4056	-2.0468	0	0.002654
GSE2770_UNTREATED_VS_IL12_TREATED_ACT_CD4_TCELL_48H_UP	186	-0.48671	-2.05465	0	0.002654
GSE6259_FLT3L_INDUCED_VS_WT_SPLENIC_DC_33D1_POS_UP	162	-0.57577	-2.04663	0	0.002655
GSE24142_EARLY_THYMIC_PROGENITOR_VS_DN3_THYMOCYTE_ADULT_DN	185	-0.49587	-2.04764	0	0.002657
GSE17186_BLOOD_VS_CORD_BLOOD_CD21LOW_TRANSITIONAL_BCELL_UP	183	-0.4673	-2.04617	0	0.002658
GSE11057_CD4_CENT_MEM_VS_PBMC_UP	179	-0.52304	-2.04766	0	0.002662
GSE3982_MAC_VS_BCELL_DN	172	-0.45352	-2.05467	0	0.002666
GSE21546_SAP1A_KO_VS_SAP1A_KO_AND_ELK1_KO_ANTI_CD3_STIM_DP_THYMOCYTES_DN	188	-0.56892	-2.04575	0	0.002666
GSE9650_EFFECTOR_VS_EXHAUSTED_CD8_TCELL_UP	180	-0.4043	-2.05476	0	0.002668
GSE45365_HEALTHY_VS_MCMV_INFECTION_CD8A_DC_UP	183	-0.35395	-2.04546	0	0.002671

GSE27786_CD4_VS_CD8_TCELL_UP	18<br>0	-<br>0.498<br>97	-<br>2.054<br>79	0	0.0026<br>74
GSE32255_UNSTIM_VS_4H_LPS_STIM_DC_UP	17<br>5	-<br>0.583<br>51	-<br>2.055<br>96	0	0.0026<br>74
GSE40274_CTRL_VS_FOXP3_AND_SATB1_TRANSDUCED_ACTIVATED_CD4_TCELL_UP	15<br>5	-<br>0.469<br>23	-<br>2.043<br>33	0	0.0026<br>75
GSE39152_CD103_NEG_VS_POS_MEMORY_CD8_TCELL_UP	19<br>0	-<br>0.477<br>64	-<br>2.043<br>4	0	0.0026<br>75
GSE39556_CD8A_DC_VS_NK_CELL_MOUSE_3H_POST_POLYIC_INJ_DN	18<br>1	-<br>0.495<br>39	-<br>2.055<br>81	0	0.0026<br>76
GSE22886_NAIVE_TCELL_VS_MONOCYTE_UP	18<br>1	-<br>0.627<br>11	-<br>2.044<br>91	0	0.0026<br>76
GSE3982_BASOPHIL_VS_CENT_MEMORY_CD4_TCELL_UP	17<br>6	-<br>0.470<br>75	-<br>2.044<br>76	0	0.0026<br>79
GSE6269_STAPH_AUREUS_VS_STREP_PNEUMO_INF_PBMC_DN	16<br>2	-<br>0.556<br>14	-<br>2.043<br>68	0	0.0026<br>79
GSE10856_CTRL_VS_TNFRSF6B_IN_MACROPHAGE_UP	16<br>3	-<br>0.508<br>83	-<br>2.044<br>49	0	0.0026<br>79
GSE22601_DOUBLE_POSITIVE_VS_CD8_SINGLE_POSITIVE_THYMOCYTE_UP	18<br>2	-<br>0.541<br>75	-<br>2.054<br>89	0	0.0026<br>79
GSE5542_UNTREATED_VS_IFNG_TREATED_EPITHELIAL_CELLS_24H_UP	18<br>5	-<br>0.561<br>21	-<br>2.044<br>31	0	0.0026<br>8
GSE3982_EOSINOPHIL_VS_DC_DN	18<br>6	-<br>0.441<br>81	-<br>2.043<br>81	0	0.0026<br>82

GSE360_CTRL_VS_L_MAJOR_DC_UP	189	-0.43122	-2.05546	0	0.002683
GSE40274_FOXP3_VS_FOXP3_AND_IRF4_TRANSDUCED_ACTIVATED_CD4_TCELL_UP	179	-0.5177	-2.04397	0	0.002683
GSE43863_TH1_VS_TFH_MEMORY_CD4_TCELL_DN	182	-0.55833	-2.05511	0	0.002683
GSE27670_BLIMP1_VS_LMP1_TRANSDUCED_GC_BCELL_UP	189	-0.56429	-2.04305	0	0.002684
GSE13946_CTRL_VS_DSS_COLITIS_GD_TCELL_FROM_COLON_DN	159	-0.54553	-2.04266	0	0.002688
GSE37301_MULTIPOTENT_PROGENITOR_VS_CD4_TCELL_UP	157	-0.572	-2.05512	0	0.002689
GSE3565_CTRL_VS_LPS_INJECTED_DUSP1_KO_SPLENOCYTES_DN	164	-0.50208	-2.03805	0	0.002707
GSE3920_UNTREATED_VS_IFNA_TREATED_ENDOTHELIAL_CELL_DN	154	-0.49369	-2.03805	0	0.002712
GSE5455_HEALTHY_VS_TUMOR_BEARING_MOUSE_SPLEEN_MONOCYTE_DN	181	-0.58363	-2.03776	0	0.002712
GSE32423_IL7_VS_IL7_IL4_NAIVE_CD8_TCELL_DN	178	-0.44936	-2.03829	0	0.002712
GSE40443_INDUCED_VS_TOTAL_TREG_UP	189	-0.46303	-2.03934	0	0.002714
GSE3720_UNSTIM_VS_PMA_STIM_VD1_GAMMADELTA_TCELL_UP	165	-0.46203	-2.03786	0	0.002714

GSE360_L_DONOVANI_VS_B_MALAYI_HIGH_DOSE_MAC_DN	191	-0.46715	-2.04149	0	0.002715
GSE42724_B1_BCELL_VS_PLASMABLAST_DN	181	-0.36316	-2.03834	0	0.002717
GSE2197_CPG_DNA_VS_UNTREATED_IN_DC_UP	177	-0.48637	-2.03959	0	0.002717
GSE369_SOCS3_KO_VS_WT_LIVER_DN	177	-0.4473	-2.0383	0	0.002718
GSE40274_FOXP3_VS_FOXP3_AND_XBP1_TRANSDUCED_ACTIVATED_CD4_TCELL_DN	146	-0.53617	-2.03841	0	0.002722
GSE24142_EARLY_THYMIC_PROGENITOR_VS_DN3_THYMOCYTE_FETAL_UP	187	-0.53252	-2.03968	0	0.002722
GSE5589_LPS_AND_IL10_VS_LPS_AND_IL6_STIM_IL10_KO_MACROPHAGE_45MIN_DN	177	-0.50152	-2.03872	0	0.002724
GSE369_SOCS3_KO_VS_WT_LIVER_POST_IL6_INJECTION_DN	183	-0.58092	-2.03848	0	0.002727
GSE40277_EOS_AND_LEF1_TRANSDUCED_VS_CTRL_CD4_TCELL_UP	181	-0.4678	-2.03975	0	0.002727
GSE16451_CTRL_VS_WEST_EQUINE_ENC_VIRUS_IMMATURE_NEURON_CELL_LINE_UP	177	-0.49382	-2.04014	0	0.00273
GSE45382_UNTREATED_VS_TGFB_TREATED_MACROPHAGES_UP	184	-0.5706	-2.04017	0	0.002736
GSE557_WT_VS_I_AB_KO_DC_UP	172	-0.39048	-2.03729	0	0.002747

GSE1460_CD4_THYMOCYTE_VS_NAIVE_CD4_TCELL_ADULT_BLOOD_DN	183	-0.45273	-2.03438	0	0.002755
GSE17721_PAM3CSK4_VS_GADIQUIMOD_0.5H_BMDC_DN	187	-0.4209	-2.03623	0	0.002757
GSE5589_IL6_KO_VS_IL10_KO_LPS_STIM_MACROPHAGE_45MIN_UP	180	-0.41796	-2.03507	0	0.002757
GSE22432_CDC_VS_COMMON_DC_PROGENITOR_UP	180	-0.52493	-2.0347	0	0.002758
GSE12392_WT_VS_IFNB_KO_CD8A_NEG_SPLEEN_DC_DN	188	-0.56691	-2.03608	0	0.002758
GSE22886_IGM_MEMORY_BCELL_VS_BLOOD_PLASMA_CELL_UP	173	-0.58768	-2.03531	0	0.002758
GSE5589_LPS_VS_LPS_AND_IL6_STIM_MACROPHAGE_45MIN_UP	180	-0.44385	-2.03454	0	0.002758
GSE22601_IMMATURE_CD4_SINGLE_POSITIVE_VS_CD8_SINGLE_POSITIVE_THYMOCYTE_UP	185	-0.58822	-2.03491	0	0.002759
GSE4984_GALECTIN1_VS_VEHICLE_CTRL_TREATED_DC_UP	165	-0.5013	-2.03384	0	0.00276
GSE17721_LPS_VS_GARDIQUIMOD_16H_BMDC_UP	189	-0.47014	-2.03499	0	0.00276
GSE17721_POLYIC_VS_CPG_24H_BMDC_UP	186	-0.46926	-2.03584	0	0.002761
GSE5099_UNSTIM_VS_MCSF_TREATED_MONOCYTE_DAY3_DN	181	-0.43641	-2.03472	0	0.002761

GSE15735_CTRL_VS_HDAC_INHIBITOR_TREATED_CD4_TCELL_2H_UP	181	-0.53909	-2.03556	0	0.002761
GSE4984_LPS_VS_VEHICLE_CTRL_TREATED_DC_DN	180	-0.57379	-2.03625	0	0.002762
GSE40184_HEALTHY_VS_HCV_INFECTED_DONOR_PBMC_DN	185	-0.43538	-2.03643	0	0.002764
GSE2770_IL12_AND_TGFB_VS_IL4_TREATED_ACT_CD4_TCELL_48H_UP	184	-0.51058	-2.03578	0	0.002764
GSE17186_NAIVE_VS_CD21LOW_TRANSITIONAL_BCELL_UP	182	-0.46147	-2.03394	0	0.002764
GSE20715_0H_VS_24H_OZONE_TLR4_KO_LUNG_UP	179	-0.5712	-2.034	0	0.002765
GSE40273_EOS_KO_VS_WT_TREG_DN	177	-0.48336	-2.03413	0	0.002766
GSE22886_NAIVE_CD8_TCELL_VS_MEMORY_TCELL_UP	177	-0.57774	-2.03658	0	0.002767
GSE16451_CTRL_VS_WEST_EQUINE_ENC_VIRUS_MATURE_NEURON_CELL_LINE_DN	184	-0.54623	-2.03371	0	0.002769
GSE22886_UNSTIM_VS_IL2_STIM_NKCELL_UP	175	-0.47662	-2.03419	0	0.002769
GSE27786_LIN_NEG_VS_MONO_MAC_DN	178	-0.45697	-2.03342	0	0.002781
GSE12845_IGD_POS_VS_NEG_BLOOD_BCELL_UP	170	-0.55341	-2.03279	0	0.00279

GSE4748_CTRL_VS_CYANOBACTERIUM_LPSLIKE_STIM_DC_3H_UP	187	-0.58631	-2.03056	0	0.002813
GSE40274_CTRL_VS_FOXP3_AND_HELIOS_TRANSDUCED_ACTIVATED_CD4_TCELL_UP	185	-0.47838	-2.03189	0	0.002813
GSE3982_BCELL_VS_EFF_MEMORY_CD4_TCELL_UP	180	-0.49052	-2.03043	0	0.002817
GSE34515_CD16_NEG_MONOCYTE_VS_DC_DN	170	-0.41152	-2.03058	0	0.002817
GSE17721_LPS_VS_PAM3CSK4_8H_BMDC_UP	183	-0.45037	-2.02639	0	0.00282
GSE11864_CSF1_VS_CSF1_PAM3CYS_IN_MAC_UP	168	-0.44149	-2.03013	0	0.002821
GSE17721_PAM3CSK4_VS_CPG_8H_BMDC_DN	182	-0.42437	-2.0306	0	0.002822
GSE19772_CTRL_VS_HCMV_INF_MONOCYTES_UP	191	-0.50855	-2.02533	0	0.002824
GSE22140_HEALTHY_VS_ARTHRITIC_MOUSE_CD4_TCELL_DN	192	-0.41893	-2.02501	0	0.002824
GSE29618_BCELL_VS_MONOCYTE_DAY7_FLU_VACCINE_UP	171	-0.64608	-2.02639	0	0.002825
GSE14415_NATURAL_TREG_VS_TCONV_UP	146	-0.57707	-2.03142	0	0.002826
GSE43863_NAIVE_VS_MEMORY_TFH_CD4_TCELL_D150_LCMV_UP	177	-0.60074	-2.03072	0	0.002827

GSE7852_LN_VS_THYMUS_TREG_UP	179	-0.57259	-2.03063	0	0.002827
GSE18281_SUBCAPSULAR_VS_PERIMEDULLARY_CORTICAL_REGION_OF_THYMUS_UP	182	-0.47352	-2.02582	0	0.002828
GSE17721_4_VS_24H_CPG_BMDC_DN	177	-0.35444	-2.02653	0	0.002828
GSE12392_WT_VS_IFNAR_KO_CD8A_NEG_SPLEEN_DC_UP	178	-0.57197	-2.02508	0	0.002829
GSE6259_33D1_POS_VS_DEC205_POS_FLT3L_INDUCED_SPLENIC_DC_DN	170	-0.43891	-2.02547	0	0.00283
GSE360_CTRL_VS_T_GONDII_DC_UP	191	-0.54659	-2.02666	0	0.002831
GSE22432_UNTREATED_VS_TGFB1_TREATED_COMMON_DC_PROGENITOR_UP	182	-0.43558	-2.02439	0	0.002832
GSE37416_CTRL_VS_6H_F_TULARENSIS_LVS_NEUTROPHIL_UP	168	-0.38823	-2.02511	0	0.002832
GSE39556_UNTREATED_VS_3H_POLYIC_INJ_MOUSE_CD8A_DC_DN	181	-0.53906	-2.02683	0	0.002832
GSE16266_LPS_VS_HEATSHOCK_AND_LPS_STIM_MEF_UP	187	-0.63931	-2.03086	0	0.002832
GSE37301_MULTIPOTENT_PROGENITOR_VS_COMMON_LYMPHOID_PROGENITOR_UP	160	-0.49807	-2.02587	0	0.002833
GSE14308_INDUCED_VS_NATURAL_TREG_DN	188	-0.43528	-2.02989	0	0.002833

GSE23568_CTRL_TRANSDUCED_VS_WT_CD8_TCELL_DN	188	-0.59711	-2.02688	0	0.002835
GSE3337_4H_VS_16H_IFNG_IN_CD8POS_DC_DN	184	-0.55217	-2.03125	0	0.002837
GSE12845_NAIVE_VS_PRE_GC_TONSIL_BCELL_UP	181	-0.45576	-2.02589	0	0.002837
GSE17721_POLYIC_VS_CPG_12H_BMDC_UP	177	-0.46591	-2.03088	0	0.002837
GSE7219_UNSTIM_VS_LPS_AND_ANTI_CD40_STIM_NIK_NFKB2_KO_DC_UP	171	-0.64005	-2.02691	0	0.00284
GSE24142_DN2_VS_DN3_THYMOCYTE_ADULT_DN	177	-0.46	-2.02712	0	0.00284
GSE39110_UNTREATED_VS_IL2_TREATED_CD8_TCELL_DAY6_POST_IMMUNIZATION_DN	187	-0.56388	-2.02834	0	0.002841
GSE14415_FOXP3_KO_NATURAL_TREG_VS_TCONV_DN	163	-0.52382	-2.01928	0	0.002841
GSE13411_NAIVE_VS_SWITCHED_MEMORY_BCELL_DN	182	-0.40509	-2.0206	0	0.002841
GSE17721_POLYIC_VS_CPG_8H_BMDC_UP	178	-0.48347	-2.02384	0	0.002842
GSE3203_HEALTHY_VS_INFLUENZA_INFECTED_LN_BCELL_UP	178	-0.49507	-2.02263	0	0.002842
GSE40277_GATA1_AND_SATB1_TRANSDUCED_VS_CTRL_CD4_TCELL_UP	182	-0.50128	-2.01906	0	0.002842

GSE40068_CXCR5POS_BCL6POS_TFH_VS_CXCR5NEG_BCL6NEG_CD4_TCELL_DN	189	-0.54465	-2.0259	0	0.002842
GSE30962_ACUTE_VS_CHRONIC_LCMV_PRIMARY_INF_CD8_TCELL_UP	178	-0.55672	-2.01948	0	0.002842
GSE27786_LIN_NEG_VS_ERYTHROBLAST_UP	184	-0.34506	-2.02763	0	0.002843
GSE37301_HEMATOPOIETIC_STEM_CELL_VS_RAG2_KO_NK_CELL_UP	164	-0.51777	-2.02246	0	0.002843
GSE40666_UNTREATED_VS_IFNA_STIM_EFFECTOR_CD8_TCELL_90MIN_DN	174	-0.4419	-2.02395	0	0.002843
GSE14308_TH1_VS_INDUCED_TREG_UP	180	-0.42496	-2.02746	0	0.002843
GSE39556_CD8A_DC_VS_NK_CELL_DN	186	-0.59446	-2.02073	0	0.002844
GSE43955_10H_VS_60H_ACT_CD4_TCELL_UP	187	-0.44326	-2.02012	0	0.002844
GSE21670_STAT3_KO_VS_WT_CD4_TCELL_TGFB_IL6_TREATED_UP	167	-0.40002	-2.01928	0	0.002845
GSE5455_EX_VIVO_VS_POST_24H_INCUBATION_MONOCYTES_FROM_TUMOR_BEARING_MOUSE_DN	185	-0.53932	-2.02099	0	0.002845
GSE36392_TYPE_2_MYELOID_VS_EOSINOPHIL_IL25_TREATED_LUNG_DN	176	-0.57276	-2.02776	0	0.002846
GSE17974_IL4_AND_ANTI_IL12_VS_UNTREATED_2H_ACT_CD4_TCELL_UP	154	-0.53697	-2.02903	0	0.002846

GSE32901_TH17_EMRICHED_VS_TH17_NEG_CD4_TCELL_DN	160	-0.4382	-2.02934	0	0.002846
GSE40273_XBP1_KO_VS_WT_TREG_UP	176	-0.45542	-2.02271	0	0.002846
GSE24142_EARLY_THYMIC_PROGENITOR_VS_DN2_THYMOCYTE_FETAL_UP	188	-0.59643	-2.01949	0	0.002847
GSE37532_TREG_VS_TCONV_CD4_TCELL_FROM_LN_DN	183	-0.43209	-2.02752	0	0.002847
GSE39110_DAY3_VS_DAY6_POST_IMMUNIZATION_CD8_TCELL_WITH_IL2_TREATMENT_DN	177	-0.49034	-2.02163	0	0.002847
GSE7460_CD8_TCELL_VS_TREG_ACT_UP	183	-0.56942	-2.02876	0	0.002847
GSE30083_SP3_VS_SP4_THYMOCYTE_DN	177	-0.6287	-2.02179	0	0.002848
GSE40666_WT_VS_STAT1_KO_CD8_TCELL_WITH_IFNA_STIM_90MIN_UP	173	-0.45764	-2.02104	0	0.002848
GSE14308_TH1_VS_NATURAL_TREG_DN	177	-0.42992	-2.02023	0	0.002849
GSE37605_TREG_VS_TCONV_C57BL6_FOXP3_FUSION_GFP_DN	157	-0.53192	-2.01953	0	0.002849
GSE24142_ADULT_VS_FETAL_DN2_THYMOCYTE_UP	189	-0.55562	-2.02852	0	0.00285
GSE29618_PDC_VS_MDC_UP	182	-0.54249	-2.02188	0	0.002851

GSE25088_WT_VS_STAT6_KO_MACROPHAGE_ROSIGLITAZONE_STIM_DN	178	-0.46781	-2.02944	0	0.002851
GSE25088_WT_VS_STAT6_KO_MACROPHAGE_IL4_STIM_UP	175	-0.48122	-2.02118	0	0.002853
GSE24634_TEFF_VS_TCONV_DAY5_IN_CULTURE_DN	178	-0.52516	-2.02145	0	0.002853
GSE37301_LYMPHOID_PRIMED_MPP_VS_CD4_TCELL_DN	177	-0.31881	-2.02195	0	0.002855
GSE5503_MLN_DC_VS_SPLEEN_DC_ACTIVATED_ALLOGENIC_TCELL_UP	184	-0.49201	-2.02279	0	0.002855
GSE28737_WT_VS_BCL6_HET_FOLLICULAR_BCELL_DN	177	-0.56146	-2.02888	0	0.002855
GSE41867_NAIVE_VS_DAY6_LCMV_EFFECTOR_CD8_TCELL_DN	177	-0.5095	-2.01864	0	0.002858
GSE5589_LPS_AND_IL10_VS_LPS_AND_IL6_STIM_IL10_KO_MACROPHAGE_45MIN_UP	186	-0.54018	-2.02311	0	0.002859
GSE5542_UNTREATED_VS_IFNA_AND_IFNG_TREATED_EPITHELIAL_CELLS_6H_DN	187	-0.55314	-2.0289	0	0.00286
GSE7831_UNSTIM_VS_CPG_STIM_PDC_1H_UP	179	-0.50637	-2.02316	0	0.002862
GSE20366_TREG_VS_NAIVE_CD4_TCELL_HOMEOSTATIC_CONVERSION_DN	186	-0.52524	-2.01686	0	0.002872
GSE12845_IGD_POS_BLOOD_VS_PRE_GC_TONSIL_BCELL_UP	180	-0.4117	-2.01711	0	0.002879

GSE21033_CTRL_VS_POLYIC_STIM_DC_24H_UP	146	-0.57073	-2.01755	0	0.00288
GSE12366_GC_VS_NAIVE_BCELL_DN	174	-0.57889	-2.01759	0	0.002884
GSE22886_NAIVE_CD4_TCELL_VS_NEUTROPHIL_UP	182	-0.49055	-2.42552	0	0.002885
GSE15324_NAIVE_VS_ACTIVATED_CD8_TCELL_UP	171	-0.54966	-2.01759	0	0.002889
GSE40274_HELIOS_VS_FOXP3_AND_HELIOS_TRANSDUCED_ACTIVATED_CD4_TCELL_UP	177	-0.56232	-2.01765	0	0.002891
GSE5679_RARA_AGONIST_AM580_VS_AM580_AND_ROSIGLITAZONE_TREATED_DC_DN	181	-0.61961	-2.01379	0	0.002898
GSE5589_LPS_VS_LPS_AND_IL10_STIM_MACROPHAGE_180MIN_UP	180	-0.54464	-2.01423	0	0.002899
GSE22601_DOUBLE_NEGATIVE_VS_CD4_SINGLE_POSITIVE_THYMOCYTE_UP	180	-0.44239	-2.01424	0	0.002903
GSE36527_CD62L_HIGH_CD69_NEG_VS_CD62L_LOW_CD69_POS_TREG_KLRG1_NEG_UP	180	-0.47131	-2.01574	0	0.002904
GSE3982_CENT_MEMORY_CD4_TCELL_VS_TH1_UP	176	-0.52255	-2.01452	0	0.002905
GSE21927_C26GM_VS_4T1_TUMOR_MONOCYTE_BALBC_DN	174	-0.46573	-2.01429	0	0.002905
GSE7460_WT_VS_FOXP3_HET_ACT_TCONV_UP	182	-0.49104	-2.0146	0	0.002907

GSE17721_0.5H_VS_8H_POLYIC_BMDC_DN	189	-0.4252	-2.01518	0	0.002907
GSE32986_UNSTIM_VS_GMCSF_STIM_DC_UP	169	-0.54884	-2.01437	0	0.002909
GSE3565_DUSP1_VS_WT_SPLENOCYTES_POST_LPS_INJECTION_UP	149	-0.42897	-2.01317	0	0.002911
GSE37301_PRO_BCELL_VS_CD4_TCELL_UP	156	-0.49936	-2.01545	0	0.002912
GSE37416_0H_VS_24H_F_TULARENSIS_LVS_NEUTROPHIL_UP	174	-0.42598	-2.01462	0	0.002912
GSE43863_DAY6_EFF_VS_DAY150_MEM_LY6C_INT_CXCR5POS_CD4_TCELL_DN	180	-0.65372	-2.01333	0	0.002912
GSE32986_CURDLAN_LOWDOSE_VS_CURDLAN_HIGHDOSE_STIM_DC_UP	174	-0.46801	-2.01339	0	0.002917
GSE3039_NKT_CELL_VS_B2_BCELL_UP	194	-0.56294	-2.01296	0	0.002918
GSE32255_WT_VS_JMJD2D_KNOCKDOWN_4H_LPS_STIM_DC_UP	144	-0.54271	-2.01166	0	0.002919
GSE14350_TREG_VS_TEFF_IN_IL2RB_KO_UP	181	-0.40534	-2.01205	0	0.002921
GSE6674_ANTI_IGM_VS_CPG_STIM_BCELL_UP	175	-0.45448	-2.01233	0	0.002922
GSE2770_IL12_AND_TGFB_ACT_VS_ACT_CD4_TCELL_2H_DN	168	-0.466	-2.01168	0	0.002924

GSE7568_CTRL_VS_24H_TGFB_TREATED_MACROPHAGES_WITH_IL4_AND_DEXAMETHASONE_UP	177	-0.49371	-2.01211	0	0.002924
GSE27786_CD4_TCELL_VS_NKTCELL_UP	183	-0.40177	-2.01249	0	0.002925
GSE5679_CTRL_VS_PPARG_LIGAND_ROSIGLITAZONE_AND_RARA_AGONIST_AM580_TREATED_DC_UP	189	-0.53491	-2.01171	0	0.002926
GSE16697_CD4_TCELL_VS_TFH_CD4_TCELL_DN	179	-0.53688	-2.01128	0	0.002927
GSE22935_24H_VS_48H_MBOVIS_BCG_STIM_MYD88_KO_MACROPHAGE_UP	183	-0.48021	-2.01077	0	0.002933
GSE2770_UNTREATED_VS_IL12_TREATED_ACT_CD4_TCELL_2H_UP	189	-0.43936	-2.01023	0	0.002942
GSE31082_DP_VS_CD4_SP_THYMOCYTE_DN	187	-0.40508	-2.00999	0	0.00295
GSE5542_UNTREATED_VS_IFNA_AND_IFNG_TREATED_EPITHELIAL_CELLS_24H_DN	184	-0.48823	-2.00967	0	0.002952
GSE14308_NAIVE_CD4_TCELL_VS_INDUCED_TREG_UP	170	-0.40233	-2.00801	0	0.002965
GSE14308_TH2_VS_NAIVE_CD4_TCELL_DN	176	-0.43259	-2.00909	0	0.002965
GSE41087_WT_VS_FOXP3_MUT_ANTI_CD3_CD28_STIM_CD4_TCELL_UP	176	-0.61328	-2.00826	0	0.002965
GSE8921_UNSTIM_0H_VS_TLR1_2_STIM_MONOCYTE_6H_DN	180	-0.52478	-2.00867	0	0.002965

GSE39110_UNTREATED_VS_IL2_TREATED_CD8_TCELL_DAY3_POST_IMMUNIZATION_UP	180	-0.53177	-2.00833	0	0.002968
GSE10325_LUPUS_CD4_TCELL_VS_LUPUS_BCELL_UP	179	-0.65943	-2.00772	0	0.00297
GSE36078_WT_VS_IL1R_KO_LUNG_DC_AFTER_AD5_T425A_HEXON_INF_DN	180	-0.42696	-2.00011	0	0.002981
GSE36078_WT_VS_IL1R_KO_LUNG_DC_AFTER_AD5_INF_DN	185	-0.53209	-2.00055	0	0.002982
GSE6674_PL2_3_VS_ANTI_IGM_AND_CPG_STIM_BCELL_DN	180	-0.469	-2.00076	0	0.002982
GSE13484_UNSTIM_VS_12H_YF17D_VACCINE_STIM_PBMC_UP	172	-0.39365	-2.00091	0	0.002984
GSE15930_NAIVE_VS_72H_IN_VITRO_STIM_TRICHOSTATINA_CD8_TCELL_UP	184	-0.44455	-2.00734	0	0.002984
GSE40273_GATA1_KO_VS_WT_TREG_DN	183	-0.49376	-2.00031	0	0.002985
GSE20715_WT_VS_TLR4_KO_48H_OZONE_LUNG_UP	185	-0.50081	-2.00011	0	0.002985
GSE7568_IL4_VS_IL4_AND_DEXAMETHASONE_TREATED_MACROPHAGE_DN	144	-0.5253	-2.00281	0	0.002986
GSE7460_CTRL_VS_TGFB_TREATED_ACT_FOXP3_MUT_TCONV_UP	181	-0.50383	-2.00093	0	0.002987
GSE25677_MPL_VS_R848_STIM_BCELL_DN	168	-0.57515	-2.00128	0	0.002987

GSE24142_EARLY_THYMIC_PROGENITOR_VS_DN3_THYMOCYTE_ADULT_UP	186	-0.54096	-2.00297	0	0.002987
GSE32533_MIR17_KO_VS_MIR17_OVEREXPRESS_ACT_CD4_TCELL_UP	184	-0.59575	-2.00311	0	0.002988
GSE32901_NAIVE_VS_TH17_ENRICHED_CD4_TCELL_UP	140	-0.5987	-2.00231	0	0.002988
GSE24726_WT_VS_E2_2_KO_PDC_DAY6_POST_DELETION_DN	187	-0.51413	-2.00299	0	0.002989
GSE12505_WT_VS_E2_2_HET_PDC_DN	145	-0.41335	-2.00329	0	0.00299
GSE11961_MARGINAL_ZONE_BCELL_VS_MEMORY_BCELL_DAY7_UP	182	-0.44399	-2.00277	0	0.002991
GSE17721_POLYIC_VS_GARDIQUIMOD_24H_BMDC_UP	179	-0.46053	-2.00132	0	0.002991
GSE17721_0.5H_VS_4H_PAM3CSK4_BMDC_DN	181	-0.4226	-2.00268	0	0.002992
GSE24634_NAIVE_CD4_TCELL_VS_DAY5_IL4_CONV_TREG_UP	176	-0.5011	-2.00321	0	0.002992
GSE26488_CTRL_VS_PEPTIDE_INJECTION_OT2_THYMOCYTE_DN	175	-0.56487	-2.00235	0	0.002992
GSE8921_3H_VS_24H_TLR1_2_STIM_MONOCYTE_UP	180	-0.48995	-2.00272	0	0.002994
GSE3982_CENT_MEMORY_CD4_TCELL_VS_TH2_UP	178	-0.51792	-2.00341	0	0.002994

GSE37416_0H_VS_12H_F_TULARENSIS_LVS_NEUTROPHIL_UP	176	-0.44986	-2.0044	0	0.002994
GSE17721_0.5H_VS_4H_CPG_BMDC_DN	183	-0.5225	-2.00133	0	0.002995
GSE21380_NON_TFH_VS_TFH_CD4_TCELL_UP	181	-0.60498	-2.00363	0	0.002995
GSE37301_HEMATOPOIETIC_STEM_CELL_VS_GRAN_MONO_PROGENITOR_DN	165	-0.48352	-2.0065	0	0.002995
GSE1460_INTRATHYMIC_T_PROGENITOR_VS_DP_THYMOCYTE_DN	178	-0.54416	-2.00148	0	0.002996
GSE7348_UNSTIM_VS_LPS_STIM_MACROPHAGE_UP	143	-0.42901	-1.99938	0	0.002996
GSE17580_UNINFECTED_VS_S_MANSONI_INF_TEFF_DN	185	-0.55505	-2.00667	0	0.002996
GSE41867_DAY15_EFFECTOR_VS_DAY30_EXHAUSTED_CD8_TCELL_LCMV_CLONE13_UP	182	-0.53097	-2.00237	0	0.002997
GSE8621_LPS_STIM_VS_LPS_PRIMED_AND_LPS_STIM_MACROPHAGE_DN	185	-0.47273	-2.00454	0	0.002997
GSE13306_TREG_VS_TCONV_SPLEEN_DN	182	-0.53746	-2.00687	0	0.002999
GSE5542_IFNA_VS_IFNA_AND_IFNG_TREATED_EPITHELIAL_CELLS_24H_UP	183	-0.51511	-2.00209	0	0.002999
GSE17721_POLYIC_VS_GARDIQUIMOD_6H_BMDC_UP	184	-0.43087	-2.00194	0	0.002999

GSE1460_INTRATHYMIC_T_PROGENITOR_VS_CD4_THYMOCYTE_DN	187	-0.64201	-2.00503	0	0.002999
GSE25677_MPL_VS_R848_STIM_BCELL_UP	164	-0.58832	-1.99876	0	0.002999
GSE15930_STIM_VS_STIM_AND_TRICHOSTATINA_48H_CD8_T_CELL_DN	189	-0.42981	-2.00691	0	0.002999
GSE25088_CTRL_VS_IL4_STIM_STAT6_KO_MACROPHAGE_UP	156	-0.58108	-2.00364	0	0.002999
GSE37605_FOXP3_FUSION_GFP_VS_IRES_GFP_TREG_NOD_DN	159	-0.58732	-1.99834	0	0.003
GSE39916_B_CELL_SPLEEN_VS_PLASMA_CELL_BONE_MARROW_DN	184	-0.60198	-2.00153	0	0.003
GSE3982_EOSINOPHIL_VS_NEUTROPHIL_UP	178	-0.38511	-1.99888	0	0.003
GSE28408_LY6G_POS_VS_NEG_DC_UP	179	-0.4715	-2.00476	0	0.003
GSE40666_UNTREATED_VS_IFNA_STIM_EFFECTOR_CD8_TCELL_90MIN_UP	181	-0.50626	-2.00462	0	0.003
GSE24142_ADULT_VS_FETAL_EARLY_THYMIC_PROGENITOR_UP	183	-0.53139	-2.00538	0	0.003001
GSE21774_CD62L_POS_CD56_DIM_VS_CD62L_NEG_CD56_DIM_NK_CELL_UP	183	-0.59261	-2.00551	0	0.003002
GSE27241_WT_CTRL_VS_DIGOXIN_TREATED_RORGT_KO_CD4_TCELL_IN_TH17_POLARIZING_CONDITIONS_UP	166	-0.5876	-2.00384	0	0.003002

GSE6269_E_COLI_VS_STAPH_AUREUS_INF_PBMC_UP	130	-0.52639	-1.99785	0	0.003002
GSE40274_FOXP3_VS_FOXP3_AND_HELIOS_TRANSDUCED_ACTIVATED_CD4_TCELL_DN	123	-0.4714	-1.99745	0	0.003002
GSE360_LOW_DOSE_B_MALAYI_VS_M_TUBERCULOSIS_DC_UP	188	-0.43628	-2.00725	0	0.003004
GSE3982_BCELL_VS_TH1_UP	171	-0.5396	-2.00389	0	0.003004
GSE20484_MCSG_VS_CXCL4_MONOCYTE_DERIVED_MACROPHAGE_DN	178	-0.46682	-2.00153	0	0.003004
GSE40666_UNTREATED_VS_IFNA_STIM_STAT1_KO_CD8_TCELL_90MIN_DN	179	-0.59755	-2.00693	0	0.003004
GSE40274_FOXP3_VS_FOXP3_AND_PBX1_TRANSDUCED_ACTIVATED_CD4_TCELL_DN	186	-0.49915	-2.00565	0	0.003004
GSE4590_SMALL_VS_LARGE_PRE_BCELL_DN	140	-0.54911	-2.00481	0	0.003004
GSE12366_NAIVE_VS_MEMORY_BCELL_DN	175	-0.53274	-1.99758	0	0.003006
GSE2706_2H_VS_8H_R848_STIM_DC_UP	173	-0.53166	-2.00399	0	0.003008
GSE10325_CD4_TCELL_VS_LUPUS_CD4_TCELL_UP	186	-0.4775	-2.0057	0	0.003008
GSE26343_WT_VS_NFAT5_KO_MACROPHAGE_LPS_STIM_UP	185	-0.46977	-1.99808	0	0.003009

GSE11961_GERMINAL_CENTER_BCELL_DAY7_VS_MEMORY_BCELL_DAY40_UP	176	-0.5136	-2.00585	0	0.00301
GSE40274_LEF1_VS_FOXP3_AND_LEF1_TRANSDUCED_ACTIVATED_CD4_TCELL_UP	188	-0.49088	-2.00413	0	0.003012
GSE19825_CD24LOW_VS_IL2RA_HIGH_DAY3_EFF_CD8_TCELL_UP	175	-0.47126	-2.00586	0	0.003014
GSE40274_CTRL_VS_FOXP3_AND_XBP1_TRANSDUCED_ACTIVATED_CD4_TCELL_DN	144	-0.54661	-1.99698	0	0.003018
GSE2770_TGFB_AND_IL4_VS_IL4_TREATED_ACT_CD4_TCELL_48H_DN	171	-0.42577	-1.99665	0	0.003021
GSE6259_DEC205_POS_DC_VS_CD4_TCELL_DN	180	-0.38728	-1.99676	0	0.003022
GSE41867_DAY6_EFFECTOR_VS_DAY30_EXHAUSTED_CD8_TCELL_LCMV_CLONE13_UP	175	-0.55795	-1.99609	0	0.003024
GSE29618_BCELL_VS_MDC_DAY7_FLU_VACCINE_UP	168	-0.65449	-1.99582	0	0.003024
KAECH_DAY8_EFF_VS_MEMORY_CD8_TCELL_DN	189	-0.44258	-1.99609	0	0.003028
GSE17721_LPS_VS_POLYIC_6H_BMDC_DN	187	-0.39743	-1.99629	0	0.003031
GSE17721_LPS_VS_POLYIC_6H_BMDC_UP	174	-0.40509	-1.99478	0	0.003057
GSE24634_TREG_VS_TCONV_POST_DAY5_IL4_CONVERSION_DN	183	-0.61032	-1.99429	0	0.003062

GSE17301_ACD3_ACD28_VS_ACD3_ACD28_AND_IFNA2_STIM_CD8_TCELL_DN	187	-0.46505	-1.99406	0	0.003065
GSE31082_DN_VS_CD8_SP_THYMOCYTE_DN	190	-0.52895	-1.99371	0	0.003079
GSE17721_LPS_VS_GARDIQUIMOD_6H_BMDC_UP	184	-0.43356	-1.99278	0	0.003081
GSE1460_INTRATHYMIC_T_PROGENITOR_VS_NAIVE_CD4_TCELL_ADULT_BLOOD_DN	178	-0.5193	-1.99308	0	0.003081
GSE27859_MACROPHAGE_VS_CD11C_INT_F480_INT_DC_UP	158	-0.51658	-1.99341	0	0.003082
GSE11864_UNTREATED_VS_CSF1_IFNG_PAM3CYS_IN_MAC_UP	162	-0.37376	-1.99279	0	0.003085
GSE35825_UNTREATED_VS_IFNA_STIM_MACROPHAGE_UP	167	-0.37905	-1.99166	0	0.003098
GSE22443_NAIVE_VS_ACT_AND_IL12_TREATED_CD8_TCELL_UP	190	-0.42657	-1.99172	0	0.003102
GSE7831_UNSTIM_VS_INFLUENZA_STIM_PDC_1H_DN	185	-0.50116	-1.99069	0	0.003115
GSE45365_WT_VS_IFNAR_KO_CD8A_DC_UP	177	-0.37386	-1.99076	0	0.003118
GSE27241_CTRL_VS_DIGOXIN_TREATED_RORGT_KO_CD4_TCELL_IN_TH17_POLARIZING_CONDITIONS_DN	166	-0.60644	-1.99096	0	0.003119
GSE10325_LUPUS_CD4_TCELL_VS_LUPUS_BCELL_DN	174	-0.67938	-1.99078	0	0.003121

GSE17974_IL4_AND_ANTI_IL12_VS_UNTREATED_0.5H_ACT_CD4_TCELL_UP	155	-0.43835	-1.99004	0	0.003132
GSE40685_NAIVE_CD4_TCELL_VS_TREG_DN	179	-0.42565	-1.99009	0	0.003136
GSE29618_BCELL_VS_MONOCYTE_UP	163	-0.64497	-1.98973	0	0.003153
GSE25123_WT_VS_PPARG_KO_MACROPHAGE_ROSIGLITAZONE_STIM_UP	180	-0.55971	-1.98906	0	0.003164
GSE2826_WT_VS_BTK_KO_BCELL_UP	180	-0.39648	-1.98893	0	0.003165
GSE32986_UNSTIM_VS_CURDLAN_HIGHDOSE_STIM_DC_DN	178	-0.57436	-1.98913	0	0.003167
GSE22886_NAIVE_BCELL_VS_BLOOD_PLASMA_CELL_UP	187	-0.57123	-1.98893	0	0.003169
GSE21670_UNTREATED_VS_TGFB_TREATED_STAT3_KO_CD4_TCELL_DN	185	-0.59237	-1.98914	0	0.00317
GSE21546_WT_VS_SAP1A_KO_AND_ELK1_KO_DP_THYMOCYTES_UP	186	-0.50643	-1.98869	0	0.003172
GSE30083_SP1_VS_SP4_THYMOCYTE_DN	178	-0.56894	-1.98872	0	0.003176
GSE5455_HEALTHY_VS_TUMOR_BEARING_MOUSE_SPLEEN_MONOCYTE_UP	172	-0.56252	-1.98891	0	0.003178
GSE15271_CXCR4_POS_VS_NEG_GC_BCELL_DN	164	-0.50927	-1.98705	0	0.003194

GSE24102_GRANULOCYSTIC_MDSC_VS_NEUTROPHIL_UP	169	-0.42963	-1.98699	0	0.003195
GSE25123_CTRL_VS_IL4_STIM_MACROPHAGE_DN	181	-0.42747	-1.98758	0	0.003196
GSE26030_UNSTIM_VS_RESTIM_TH17_DAY15_POST_POLARIZATION_DN	187	-0.36525	-1.98724	0	0.003196
GSE16450_IMMATURE_VS_MATURE_NEURON_CELL_LINE_12H_IFNA_STIM_DN	176	-0.43733	-1.98683	0	0.003197
GSE45739_NRAS_KO_VS_WT_ACD3_ACD28_STIM_CD4_TCELL_DN	172	-0.44757	-1.98739	0	0.003197
GSE17186_NAIVE_VS_CD21LOW_TRANSITIONAL_BCELL_DN	177	-0.46472	-1.98795	0	0.003198
GSE19401_UNSTIM_VS_RETINOIC_ACID_STIM_FOLLICULAR_DC_DN	177	-0.51462	-1.98767	0	0.0032
GSE37532_WT_VS_PPARG_KO_VISCERAL_ADIPOSE_TISSUE_TREG_DN	187	-0.42077	-1.98615	0	0.003206
GSE28237_FOLLICULAR_VS_EARLY_GC_BCELL_UP	165	-0.43211	-1.98641	0	0.003209
GSE360_L_MAJOR_VS_T_GONDII_MAC_UP	186	-0.57915	-1.98588	0	0.00321
GSE10325_LUPUS_CD4_TCELL_VS_LUPUS_MYELOID_UP	178	-0.63981	-1.98616	0	0.00321
GSE3039_CD4_TCELL_VS_B2_BCELL_UP	181	-0.55534	-1.98552	0	0.003212

GSE7460_CTRL_VS_TGFB_TREATED_ACT_FOXP3_HET_TCONV_DN	185	-0.48499	-1.98622	0	0.003214
GSE9650_NAIVE_VS_MEMORY_CD8_TCELL_DN	188	-0.5848	-1.98552	0	0.003216
GSE3982_MAST_CELL_VS_DC_UP	175	-0.49361	-1.98552	0	0.00322
GSE27786_LSK_VS_ERYTHROBLAST_UP	181	-0.32252	-1.98561	0	0.003224
GSE25123_CTRL_VS_ROSIGLITAZONE_STIM_PPARG_KO_MACROPHAGE_DN	182	-0.4987	-1.98521	0	0.003225
GSE15767_MED_VS_SCS_MAC_LN_DN	179	-0.43602	-1.98526	0	0.003232
GSE3039_NKT_CELL_VS_ALPHABETA_CD8_TCELL_DN	175	-0.43267	-1.9837	0	0.003251
GSE45739_UNSTIM_VS_ACD3_ACD28_STIM_WT_CD4_TCELL_DN	181	-0.62863	-1.98347	0	0.003251
GSE17721_ALL_VS_24H_PAM3CSK4_BMDC_UP	182	-0.5041	-1.98447	0	0.003252
GSE22935_24H_VS_48H_MBOVIS_BCG_STIM_MACROPHAGE_UP	181	-0.54745	-1.98374	0	0.003253
GSE13306_LAMINA_PROPRIA_VS_SPLEEN_TREG_UP	184	-0.44915	-1.98382	0	0.003254
GSE11961_MEMORY_BCELL_DAY7_VS_GERMINAL_CENTER_BCELL_DAY7_UP	179	-0.4922	-1.98257	0	0.003254

GSE6259_FLT3L_INDUCED_DEC205_POS_DC_VS_CD8_TCELL_UP	139	-0.54338	-1.98304	0	0.003254
GSE28737_WT_VS_BCL6_HET_MARGINAL_ZONE_BCELL_UP	174	-0.48097	-1.9826	0	0.003254
KAECH_NAIVE_VS_MEMORY_CD8_TCELL_DN	188	-0.58989	-1.98307	0	0.003257
GSE23502_WT_VS_HDC_KO_MYELOID_DERIVED_SUPPRESSOR_CELL_BM_UP	182	-0.53079	-1.9826	0	0.003258
GSE11057_PBMC_VS_MEM_CD4_TCELL_DN	171	-0.5611	-1.98276	0	0.00326
GSE36078_UNTREATED_VS_AD5_T425A_HEXON_INF_MOUSE_LUNG_DC_UP	180	-0.48248	-1.98268	0	0.003261
GSE11961_FOLLICULAR_BCELL_VS_MEMORY_BCELL_DAY7_UP	178	-0.44136	-1.98278	0	0.003264
GSE15735_CTRL_VS_HDAC_INHIBITOR_TREATED_CD4_TCELL_2H_DN	180	-0.57966	-1.98231	0	0.003266
GSE30962_ACUTE_VS_CHRONIC_LCMV_SECONDARY_INF_CD8_TCELL_UP	176	-0.50769	-1.98279	0	0.003267
GSE9037_CTRL_VS_LPS_4H_STIM_IRAK4_KO_BMDM_UP	171	-0.41474	-1.98099	0	0.003277
GSE32423_MEMORY_VS_NAIVE_CD8_TCELL_IL7_UP	177	-0.52878	-1.98127	0	0.003278
GSE26495_NAIVE_VS_PD1LOW_CD8_TCELL_DN	185	-0.64367	-1.98138	0	0.003278

GSE17721_PAM3CSK4_VS_CPG_4H_BMDC_DN	181	-0.52019	-1.98105	0	0.00328
GSE40666_WT_VS_STAT4_KO_CD8_TCELL_DN	173	-0.38248	-1.98203	0	0.00328
GSE13522_WT_VS_IFNAR_KO_SKIN_UP	181	-0.50318	-1.9819	0	0.00328
GSE17721_PAM3CSK4_VS_GADIQUIMOD_6H_BMDC_DN	185	-0.45673	-1.98171	0	0.003281
GSE29618_BCELL_VS_MDC_UP	167	-0.64129	-1.98143	0	0.003282
GSE7460_FOXP3_MUT_VS_WT_ACT_WITH_TGFB_TCONV_DN	180	-0.52056	-1.98109	0	0.003284
GSE26290_CTRL_VS_AKT_INHIBITOR_TREATED_ANTI_CD3_AND_IL2_STIM_CD8_TCELL_DN	182	-0.51768	-1.98153	0	0.003284
GSE7460_CTRL_VS_TGFB_TREATED_ACT_FOXP3_MUT_TCONV_DN	182	-0.54529	-1.98013	0	0.003301
GSE20198_IL12_IL18_VS_IFNA_TREATED_ACT_CD4_TCELL_UP	181	-0.37159	-1.98003	0	0.003303
GSE24634_NAIVE_CD4_TCELL_VS_DAY3_IL4_CONV_TREG_UP	175	-0.50403	-1.97878	0	0.003311
GSE29618_BCELL_VS_PDC_DAY7_FLU_VACCINE_UP	167	-0.62553	-1.97833	0	0.003316
GSE11386_NAIVE_VS_MEMORY_BCELL_DN	154	-0.52701	-1.97931	0	0.003317

GSE3920_IFNA_VS_IFNG_TREATED_FIBROBLAST_UP	163	-0.58519	-1.97902	0	0.003317
GSE10325_CD4_TCELL_VS_BCELL_DN	171	-0.68803	-1.97842	0	0.00332
GSE2706_2H_VS_8H_LPS_STIM_DC_UP	184	-0.51157	-1.97937	0	0.003321
GSE7548_NAIVE_VS_DAY28_PCC_IMMUNIZATION_CD4_TCELL_UP	188	-0.43312	-1.97747	0	0.003321
GSE11961_UNSTIM_VS_ANTI_IGM_AND_CD40_STIM_6H_FOLLICULAR_BCELL_DN	179	-0.5452	-1.97667	0	0.003323
GSE11057_NAIVE_CD4_VS_PBMC_CD4_TCELL_UP	166	-0.45599	-1.9766	0	0.003324
GSE11961_MEMORY_BCELL_DAY7_VS_GERMINAL_CENTER_BCELL_DAY7_DN	183	-0.5405	-1.97638	0	0.003324
GSE3039_CD4_TCELL_VS_B1_BCELL_UP	188	-0.50655	-1.97698	0	0.003325
GSE8835_HEALTHY_VS_CLL_CD4_TCELL_UP	178	-0.51542	-1.97754	0	0.003325
GSE37416_CTRL_VS_24H_F_TULARENSIS_LVS_NEUTROPHIL_UP	172	-0.4495	-1.9771	0	0.003325
GSE17974_IL4_AND_ANTI_IL12_VS_UNTREATED_48H_ACT_CD4_TCELL_DN	162	-0.6402	-1.97647	0	0.003326
GSE24972_WT_VS_IRF8_KO_SPLEEN_FOLLICULAR_BCELL_DN	177	-0.47542	-1.97606	0	0.003327

GSE17721_CTRL_VS_GARDIQUIMOD_0.5H_BMDC_UP	182	-0.38452	-1.9751	0	0.00333
GSE3039_ALPHAALPHA_VS_ALPHABETA_CD8_TCELL_DN	181	-0.68698	-1.97761	0	0.003333
GSE24142_EARLY_THYMIC_PROGENITOR_VS_DN2_THYMOCYTE_ADULT_UP	181	-0.59456	-1.97514	0	0.003333
GSE41176_UNSTIM_VS_ANTI_IGM_STIM_BCELL_6H_DN	184	-0.46351	-1.9756	0	0.003333
GSE24142_DN2_VS_DN3_THYMOCYTE_DN	191	-0.53125	-1.97578	0	0.003334
GSE43955_10H_VS_30H_ACT_CD4_TCELL_WITH_TGFB_IL6_UP	183	-0.47583	-1.97473	0	0.003335
GSE22886_CD8_TCELL_VS_BCELL_NAIVE_DN	179	-0.64557	-1.9752	0	0.003337
GSE5589_WT_VS_IL6_KO_LPS_AND_IL10_STIM_MACROPHAGE_45MIN_DN	182	-0.40459	-1.97258	0	0.003337
GSE11961_MARGINAL_ZONE_BCELL_VS_MEMORY_BCELL_DAY40_DN	170	-0.43896	-1.97524	0	0.003337
GSE46606_IRF4MID_VS_WT_CD40L_IL2_IL5_DAY3_STIMULATED_BCELL_DN	180	-0.39614	-1.97272	0	0.003338
GSE20727_H2O2_VS_ROS_INHIBITOR_TREATED_DC_UP	176	-0.50293	-1.97447	0	0.003339
GSE6092_B_BURGDOFERI_VS_B_BURGDORFERI_AND_IFNG_STIM_ENDOTHELIAL_CELL_UP	165	-0.42017	-1.97483	0	0.003339

GSE21360_SECONDARY_VS_TERTIARY_MEMORY_CD8_TCELL_DN	153	-0.52204	-1.97484	0	0.00334
GSE15733_BM_VS_SPLEEN_MEMORY_CD4_TCELL_DN	181	-0.40322	-1.97533	0	0.003341
GSE25088_IL4_VS_IL4_AND_ROSIGLITAZONE_STIM_MACROPHAGE_DAY10_DN	181	-0.50963	-1.97275	0	0.003342
GSE3920_IFNA_VS_IFNB_TREATED_ENDOTHELIAL_CELL_DN	155	-0.65579	-1.97392	0	0.003343
GSE339_CD8POS_VS_CD4CD8DN_DC_UP	186	-0.4278	-1.97372	0	0.003344
GSE24972_MARGINAL_ZONE_BCELL_VS_FOLLICULAR_BCELL_IRF8_KO_DN	174	-0.51457	-1.9731	0	0.003344
GSE3982_EOSINOPHIL_VS_MAST_CELL_UP	187	-0.53774	-1.97406	0	0.003344
GSE4590_LARGE_PRE_BCELL_VS_VPREB_POS_LARGE_PRE_BCELL_UP	163	-0.51647	-1.97313	0	0.003346
GSE19198_1H_VS_24H_IL21_TREATED_TCELL_UP	185	-0.60332	-1.97407	0	0.003347
GSE13522_WT_VS_IFNAR_KO_SKIN_DN	168	-0.55143	-1.9738	0	0.003347
GSE17974_1.5H_VS_72H_IL4_AND_ANTI_IL12_ACT_CD4_TCELL_UP	174	-0.45405	-1.9741	0	0.00335
GSE13229_MATURE_VS_INTMATURE_NKCELL_DN	176	-0.38025	-1.97414	0	0.00335

GSE12845_IGD_POS_BLOOD_VS_DARKZONE_GC_TONSIL_BCELL_UP	171	-0.47567	-1.97077	0	0.003363
GSE37301_CD4_TCELL_VS_GRANULOCYTE_MONOCYTE_PROGENITOR_UP	155	-0.57181	-1.96987	0	0.003364
GSE17721_PAM3CSK4_VS_GADIQUIMOD_16H_BMDC_UP	183	-0.48998	-1.97078	0	0.003367
GSE1740_UNSTIM_VS_IFNA_STIMULATED_MCSF_IFNG_DERIVED_MACROPHAGE_UP	155	-0.58343	-1.9709	0	0.003368
GSE14699_NAIVE_VS_ACT_CD8_TCELL_DN	164	-0.50494	-1.97095	0	0.003368
GSE16385_UNTREATED_VS_12H_ROSIGLITAZONE_IL4_TREATED_MACROPHAGE_UP	185	-0.33827	-1.96812	0	0.003369
GSE3994_WT_VS_PAC1_KO_ACTIVATED_MAST_CELL_DN	190	-0.46234	-1.97003	0	0.00337
GSE7348_LPS_VS_TOLERIZED_AND_LPS_STIM_MACROPHAGE_DN	161	-0.50249	-1.97029	0	0.003371
GSE26928_NAIVE_VS_CXCR5_POS_CD4_TCELL_UP	159	-0.50616	-1.97109	0	0.003371
GSE32986_UNSTIM_VS_GMCSF_AND_CURDLAN_LOWDOSE_STIM_DC_DN	173	-0.54185	-1.97201	0	0.003371
GSE22432_MULTIPOTENT_VS_COMMON_DC_PROGENITOR_DN	184	-0.43774	-1.96793	0	0.003371
GSE1925_CTRL_VS_3H_IFNG_STIM_IFNG_PRIMED_MACROPHAGE_UP	162	-0.46351	-1.96824	0	0.003373

GSE360_CTRL_VS_B_MALAYI_HIGH_DOSE_MAC_DN	193	-0.45239	-1.97098	0	0.003374
GSE7460_CD8_TCELL_VS_CD4_TCELL_ACT_UP	180	-0.6089	-1.9711	0	0.003375
GSE21927_UNTREATED_VS_GMCSF_IL6_TREATED_BONE_MARROW_DN	152	-0.49537	-1.96824	0	0.003376
GSE11924_TH2_VS_TH17_CD4_TCELL_DN	179	-0.41146	-1.97117	0	0.003378
GSE29618_BCELL_VS_PDC_UP	173	-0.62261	-1.96927	0	0.003379
GSE40274_CTRL_VS_EOS_TRANSDUCED_ACTIVATED_CD4_TCELL_DN	137	-0.62393	-1.96857	0	0.003379
GSE2585_AIRE_KO_VS_WT_CD80_LOW_MTEC_DN	187	-0.48744	-1.96834	0	0.00338
GSE17721_PAM3CSK4_VS_CPG_12H_BMDC_DN	183	-0.47107	-1.9715	0	0.003382
GSE7831_UNSTIM_VS_INFLUENZA_STIM_PDC_4H_DN	180	-0.43529	-1.97133	0	0.003382
GSE6259_FLT3L_INDUCED_33D1_POS_DC_VS_CD8_TCELL_DN	136	-0.54451	-1.9693	0	0.003382
GSE5589_LPS_VS_LPS_AND_IL6_STIM_IL6_KO_MACROPHAGE_45MIN_DN	184	-0.44198	-1.96875	0	0.003384
GSE21927_SPLEEN_VS_BONE_MARROW_MONOCYTE_BALBC_DN	169	-0.47435	-1.96758	0	0.003385

GSE7831_1H_VS_4H_INFLUENZA_STIM_PDC_DN	180	-0.55951	-1.96651	0	0.003403
GSE3982_MEMORY_CD4_TCELL_VS_TH1_UP	176	-0.49481	-1.96695	0	0.003404
GSE41867_NAIVE_VS_DAY6_LCMV_ARMSTRONG_EFFECTOR_CD8_TCELL_UP	182	-0.45341	-1.96622	0	0.003406
GSE22886_CD4_TCELL_VS_BCELL_NAIVE_DN	173	-0.632	-1.96629	0	0.003409
GSE25088_CTRL_VS_IL4_AND_ROSIGLITAZONE_STIM_MACROPHAGE_DN	171	-0.3963	-1.965	0	0.003423
GSE36888_STAT5_AB_KNOCKIN_VS_WT_TCELL_IL2_TREATED_2H_DN	186	-0.52213	-1.96509	0	0.003425
GSE24142_ADULT_VS_FETAL_DN3_THYMOCYTE_UP	183	-0.45724	-1.95885	0	0.003426
GSE7852_THYMUS_VS_FAT_TREG_DN	179	-0.51009	-1.95922	0	0.003426
GSE26495_NAIVE_VS_PD1HIGH_CD8_TCELL_DN	180	-0.64829	-1.95876	0	0.003427
GSE1925_CTRL_VS_24H_IFNG_STIM_MACROPHAGE_UP	174	-0.45668	-1.96527	0	0.003428
GSE17186_NAIVE_VS_CD21HIGH_TRANSITIONAL_BCELL_CORD_BLOOD_DN	175	-0.44639	-1.95899	0	0.003428
GSE1460_NAIVE_CD4_TCELL_ADULT_BLOOD_VS_THYMIC_STROMAL_CELL_UP	179	-0.42647	-1.96551	0	0.003428

GSE23568_ID3_TRANSDUCED_VS_ID3_KO_CD8_TCELL_UP	188	-0.56175	-1.95704	0	0.003428
GSE17721_LPS_VS_GARDIQUIMOD_12H_BMDC_UP	181	-0.44204	-1.96511	0	0.003429
GSE23308_WT_VS_MINERALCORTICOID_REC_KO_MACROPHAGE_CORTICOSTERONE_TREATED_UP	176	-0.31276	-1.95924	0	0.003429
GSE34006_UNTREATED_VS_A2AR_AGONIST_TREATED_TREG_DN	185	-0.47567	-1.959	0	0.003431
GSE26890_CXCR1_NEG_VS_POS_EFFECTOR_CD8_TCELL_DN	175	-0.41097	-1.95726	0	0.003432
GSE2770_IL12_AND_TGFB_VS_IL4_TREATED_ACT_CD4_TCELL_48H_DN	159	-0.56351	-1.95704	0	0.003432
GSE36888_UNTREATED_VS_IL2_TREATED_STAT5_AB_KNOCKIN_TCELL_2H_DN	179	-0.51868	-1.96552	0	0.003432
GSE3982_DC_VS_EFF_MEMORY_CD4_TCELL_DN	178	-0.50364	-1.95927	0	0.003433
GSE40655_FOXO1_KO_VS_WT_NTREG_UP	178	-0.38553	-1.96558	0	0.003433
GSE339_CD4POS_VS_CD8POS_DC_IN_CULTURE_UP	183	-0.44853	-1.95734	0	0.003435
GSE11864_UNTREATED_VS_CSF1_IN_MAC_DN	173	-0.41574	-1.95743	0	0.003435
GSE42724_NAIVE_BCELL_VS_PLASMABLAST_DN	174	-0.54242	-1.95929	0	0.003436

GSE17721_LPS_VS_POLYIC_12H_BMDC_DN	181	-0.45061	-1.95745	0	0.003437
GSE32986_CURDLAN_HIGHDOSE_VS_GMCSF_AND_CURDLAN_HIGHDOSE_STIM_DC_DN	189	-0.59738	-1.96066	0	0.003438
GSE26669_CD4_VS_CD8_TCELL_IN_MLR_COSTIM_BLOCK_UP	172	-0.42581	-1.96233	0	0.003438
GSE37532_VISCERAL_ADIPOSE_TISSUE_VS_LN_DERIVED_TREG_CD4_TCELL_UP	159	-0.44474	-1.96089	0	0.003438
GSE5542_UNTREATED_VS_IFNA_TREATED_EPITHELIAL_CELLS_6H_DN	171	-0.46667	-1.96209	0	0.003438
GSE21033_1H_VS_24H_POLYIC_STIM_DC_UP	156	-0.51868	-1.96104	0	0.003438
GSE26495_PD1HIGH_VS_PD1LOW_CD8_TCELL_UP	161	-0.60356	-1.95962	0	0.003438
GSE17721_CPG_VS_GARDIQUIMOD_8H_BMDC_DN	178	-0.36373	-1.95937	0	0.003439
GSE11057_CD4_EFF_MEM_VS_PBMC_UP	182	-0.47437	-1.96094	0	0.003439
GSE36888_STAT5_AB_KNOCKIN_VS_WT_TCELL_IL2_TREATED_6H_UP	174	-0.45381	-1.96113	0	0.00344
GSE17721_LPS_VS_CPG_16H_BMDC_UP	182	-0.46254	-1.95637	0	0.00344
GSE360_DC_VS_MAC_L_MAJOR_DN	189	-0.49456	-1.95749	0	0.003441

GSE30971_WBP7_HET_VS_KO_MACROPHAGE_4H_LPS_STIM_DN	168	-0.60725	-1.96246	0	0.003441
GSE24671_CTRL_VS_SENDAI_VIRUS_INFECTED_MOUSE_SPLENOCYTES_UP	153	-0.59904	-1.95786	0	0.003441
GSE3982_MAST_CELL_VS_NEUTROPHIL_DN	184	-0.50748	-1.96234	0	0.003441
GSE20727_H2O2_VS_ROS_INHIBITOR_TREATED_DC_DN	179	-0.4726	-1.96012	0	0.003441
GSE17721_POLYIC_VS_GARDIQUIMOD_8H_BMDC_UP	188	-0.44041	-1.96256	0	0.003441
GSE22935_UNSTIM_VS_48H_MBOVIS_BCG_STIM_MACROPHAGE_DN	184	-0.45136	-1.95965	0	0.003442
GSE17721_POLYIC_VS_CPG_2H_BMDC_DN	179	-0.50451	-1.95985	0	0.003444
GSE3039_B2_VS_B1_BCELL_UP	186	-0.55504	-1.95638	0	0.003444
GSE9316_IL6_KO_VS_IFNG_KO_INVIVO_EXPANDED_CD4_TCELL_UP	187	-0.49719	-1.96114	0	0.003444
GSE25087_TREG_VS_TCONV_FETUS_UP	176	-0.4656	-1.96018	0	0.003444
GSE40441_NRP1_POS_INDUCED_TREG_VS_NRP1_NEG_NATURAL_TREG_UP	183	-0.53608	-1.96029	0	0.003444
GSE13484_12H_VS_3H_YF17D_VACCINE_STIM_PBMC_DN	181	-0.43546	-1.96014	0	0.003445

GSE40274_CTRL_VS_FOXP3_AND_GATA1_TRANSDUCED_ACTIVATED_CD4_TCELL_UP	134	-0.61487	-1.95646	0	0.003445
GSE15624_CTRL_VS_3H_HALOFUGINONE_TREATED_CD4_TCELL_UP	152	-0.41265	-1.96259	0	0.003445
GSE17721_LPS_VS_CPG_1H_BMDC_DN	194	-0.4546	-1.95966	0	0.003445
GSE40666_NAIVE_VS_EFFECTOR_CD8_TCELL_WITH_IFNA_STIM_90MIN_DN	173	-0.44682	-1.95989	0	0.003446
GSE15330_HSC_VS_MEGAKARYOCYTE_ERYTHROID_PROGENITOR_UP	151	-0.54153	-1.96022	0	0.003446
GSE411_UNSTIM_VS_100MIN_IL6_STIM_SOCS3_KO_MACROPHAGE_DN	188	-0.44999	-1.96119	0	0.003447
GSE40685_NAIVE_CD4_TCELL_VS_TREG_UP	164	-0.42027	-1.95629	0	0.003448
GSE18791_CTRL_VS_NEWCASTLE_VIRUS_DC_2H_UP	160	-0.45668	-1.9626	0	0.003448
GSE43863_DAY6_EFF_VS_DAY150_MEM_TH1_CD4_TCELL_DN	184	-0.54006	-1.96022	0	0.00345
GSE43955_10H_VS_30H_ACT_CD4_TCELL_UP	191	-0.53431	-1.96144	0	0.003451
GSE360_L_MAJOR_VS_B_MALAYI_LOW_DOSE_MAC_DN	177	-0.36543	-1.96156	0	0.003452
GSE26030_TH1_VS_TH17_DAY15_POST_POLARIZATION_UP	174	-0.45466	-1.96268	0	0.003452

GSE17974_IL4_AND_ANTI_IL12_VS_UNTREATED_12H_ACT_CD4_TCELL_UP	172	-0.49466	-1.96166	0	0.003453
GSE40274_CTRL_VS_FOXP3_AND_IRF4_TRANSDUCED_ACTIVATED_CD4_TCELL_UP	146	-0.59504	-1.96381	0	0.003453
GSE15330_WT_VS_IKAROS_KO_GRANULOCYTE_MONOCYTE_PROGENITOR_UP	184	-0.37802	-1.96125	0	0.003454
GSE3982_EOSINOPHIL_VS_NKCELL_UP	183	-0.49443	-1.96146	0	0.003454
GSE34515_CD16_POS_MONOCYTE_VS_DC_DN	178	-0.54413	-1.96288	0	0.003455
GSE22886_IGG_IGA_MEMORY_BCELL_VS_BLOOD_PLASMA_CELL_UP	183	-0.54658	-1.96341	0	0.003455
GSE360_DC_VS_MAC_L_DONOVANI_UP	188	-0.46705	-1.9633	0	0.003456
GSE33425_CD161_HIGH_VS_NEG_CD8_TCELL_UP	191	-0.44836	-1.96301	0	0.003456
GSE43955_1H_VS_10H_ACT_CD4_TCELL_WITH_TGFB_IL6_UP	182	-0.4303	-1.96389	0	0.003457
GSE18893_CTRL_VS_TNF_TREATED_TCONV_24H_DN	182	-0.47871	-1.96306	0	0.003463
GSE25123_CTRL_VS_IL4_AND_ROSIGLITAZONE_STIM_PPARG_KO_MACROPHAGE_DN	179	-0.49987	-1.95537	0	0.003471
GSE22229_RENAL_TRANSPLANT_IMMUNOSUPP_THERAPY_VS_HEALTHY_PBMC_UP	181	-0.49629	-1.95546	0	0.003473

GSE41867_NAIVE_VS_DAY8_LCMV_ARMSTRONG_EFFECTOR_CD8_TCELL_DN	184	-0.43029	-1.95552	0	0.00348
GSE40274_CTRL_VS_FOXP3_AND_LEF1_TRANSDUCED_ACTIVATED_CD4_TCELL_UP	160	-0.66592	-1.95454	0	0.003517
GSE14699_NAIVE_VS_DELETIONAL_TOLERANCE_CD8_TCELL_DN	186	-0.58437	-1.95355	0	0.003529
GSE8835_CD4_VS_CD8_TCELL_CLL_PATIENT_UP	186	-0.64481	-1.95376	0	0.003529
GSE5542_IFNA_VS_IFNA_AND_IFNG_TREATED_EPITHELIAL_CELLS_6H_DN	173	-0.40686	-1.9541	0	0.00353
GSE37301_HEMATOPOIETIC_STEM_CELL_VS_RAG2_KO_NK_CELL_DN	174	-0.55777	-1.95379	0	0.003532
GSE3039_CD4_TCELL_VS_B1_BCELL_DN	185	-0.55942	-1.95307	0	0.003532
GSE2770_IL12_VS_TGFB_AND_IL12_TREATED_ACT_CD4_TCELL_48H_UP	183	-0.52691	-1.95384	0	0.003533
GSE30153_LUPUS_VS_HEALTHY_DONOR_BCELL_DN	188	-0.55552	-1.95401	0	0.003535
GSE27896_HDAC6_KO_VS_WT_TREG_DN	156	-0.44552	-1.95386	0	0.003536
GSE10239_MEMORY_VS_DAY4.5_EFF_CD8_TCELL_UP	181	-0.47172	-1.9526	0	0.003542
GSE25088_IL4_VS_IL4_AND_ROSIGLITAZONE_STIM_STAT6_KO_MACROPHAGE_DAY10_UP	182	-0.47853	-1.95261	0	0.003545

GSE21670_IL6_VS_TGFB_AND_IL6_TREATED_CD4_TCELL_UP	169	-0.49334	-1.9527	0	0.003546
GSE44649_WT_VS_MIR155_KO_ACTIVATED_CD8_TCELL_DN	179	-0.53641	-1.952	0	0.003554
GSE32533_MIR17_KO_VS_MIR17_OVEREXPRESS_ACT_CD4_TCELL_DN	178	-0.54191	-1.95157	0	0.003554
GSE33292_WT_VS_TCF1_KO_DN3_THYMOCYTE_UP	175	-0.4751	-1.95162	0	0.003558
GSE21670_TGFB_VS_IL6_TREATED_STAT3_KO_CD4_TCELL_DN	183	-0.56442	-1.95166	0	0.00356
GSE8621_LPS_PRIMED_UNSTIM_VS_LPS_PRIMED_AND_LPS_STIM_MACROPHAGE_UP	179	-0.39013	-1.95168	0	0.003563
GSE19888_ADENOSINE_A3R_INH_PRETREAT_AND_ACT_BY_A3R_VS_A3R_INH_AND_TCELL_MEMBRANES_ACT_MAST_CELL_DN	179	-0.35536	-1.95205	0	0.003564
GSE25087_TREG_VS_TCONV_ADULT_DN	161	-0.46621	-1.95118	0	0.00357
GSE14415_NATURAL_TREG_VS_FOXP3_KO_NATURAL_TREG_UP	147	-0.56688	-1.951	0	0.003575
GSE37532_VISCERAL_ADIPOSE_TISSUE_VS_LN_DERIVED_TREG_CD4_TCELL_DN	141	-0.46617	-1.95033	0	0.003588
GSE36476_CTRL_VS_TSST_ACT_16H_MEMORY_CD4_TCELL_YOUNG_UP	181	-0.49728	-1.95035	0	0.003591
GSE1460_CORD_VS_ADULT_BLOOD_NAIVE_CD4_TCELL_UP	166	-0.4448	-1.95038	0	0.003595

GSE26669_CD4_VS_CD8_TCELL_IN_MLR_COSTIM_BLOCK_DN	188	-0.50745	-1.94996	0	0.003599
GSE37563_WT_VS_CTLA4_KO_CD4_TCELL_D4_POST_IMMUNIZATION_UP	153	-0.50304	-1.94967	0	0.003604
GSE22229_RENAL_TRANSPLANT_VS_HEALTHY_PBMC_DN	176	-0.46613	-1.94932	0	0.003606
GSE29618_PDC_VS_MDC_DAY7_FLU_VACCINE_UP	180	-0.54199	-1.94939	0	0.003607
GSE16450_IMMATURE_VS_MATURE_NEURON_CELL_LINE_12H_IFNA_STIM_UP	187	-0.57707	-1.94923	0	0.003608
GSE2770_IL4_ACT_VS_ACT_CD4_TCELL_48H_DN	179	-0.48895	-1.94939	0	0.003611
GSE23502_WT_VS_HDC_KO_MYELOID_DERIVED_SUPPRESSOR_CELL_BM_DN	184	-0.5196	-1.94888	0	0.003615
GSE15735_2H_VS_12H_HDAC_INHIBITOR_TREATED_CD4_TCELL_UP	186	-0.5475	-1.94894	0	0.003616
GSE43863_NAIVE_VS_MEMORY_TH1_CD4_TCELL_D150_LCMV_UP	154	-0.6064	-1.94745	0	0.003618
GSE23925_LIGHT_ZONE_VS_DARK_ZONE_BCELL_DN	184	-0.4724	-1.9476	0	0.003619
GSE17721_LPS_VS_CPG_0.5H_BMDC_DN	187	-0.50282	-1.94826	0	0.00362
GSE15930_NAIVE_VS_72H_IN_VITRO_STIM_IL12_CD8_TCELL_UP	185	-0.43486	-1.94746	0	0.003621

GSE37301_PRO_BCELL_VS_CD4_TCELL_DN	151	-0.60579	-1.94768	0	0.003622
GSE22886_NAIVE_CD4_TCELL_VS_MONOCYTE_UP	184	-0.64044	-1.94831	0	0.003623
GSE7460_CTRL_VS_TGFB_TREATED_ACT_TCONV_DN	183	-0.47735	-1.94778	0	0.003623
GSE27859_DC_VS_CD11C_INT_F480_INT_DC_UP	179	-0.41071	-1.94791	0	0.003625
GSE32255_UNSTIM_VS_4H_LPS_STIM_DC_DN	147	-0.47864	-1.94858	0	0.003625
GSE36392_TYPE_2_MYELOID_VS_EOSINOPHIL_IL25_TREATED_LUNG_UP	174	-0.41851	-1.9465	0	0.003632
GSE3982_MAST_CELL_VS_MAC_UP	183	-0.38575	-1.94703	0	0.003634
GSE8921_UNSTIM_VS_TLR1_2_STIM_MONOCYTE_3H_DN	173	-0.3905	-1.94668	0	0.003638
GSE17974_CTRL_VS_ACT_IL4_AND_ANTI_IL12_1H_CD4_TCELL_DN	177	-0.46304	-1.94627	0	0.003639
GSE7460_CTRL_VS_FOXP3_OVEREXPR_TCONV_1_DN	178	-0.48749	-1.94543	0	0.003647
GSE17721_LPS_VS_PAM3CSK4_16H_BMDC_UP	185	-0.44439	-1.94602	0	0.003647
GSE3982_EOSINOPHIL_VS_TH2_UP	180	-0.48157	-1.94554	0	0.003651

GSE42724_NAIVE_VS_MEMORY_BCELL_UP	175	-0.50546	-1.94555	0	0.003654
GSE17721_LPS_VS_GARDIQUIMOD_0.5H_BMDC_UP	186	-0.46016	-1.94557	0	0.003654
GSE42724_NAIVE_VS_B1_BCELL_UP	183	-0.43198	-1.9457	0	0.003655
GSE7460_TREG_VS_TCONV_ACT_UP	177	-0.50221	-1.94515	0	0.003656
GSE7460_FOXP3_MUT_VS_WT_ACT_TCONV_DN	178	-0.50722	-1.94572	0	0.003658
GSE17721_LPS_VS_POLYIC_16H_BMDC_UP	181	-0.38067	-1.94394	0	0.003661
GSE12845_IGD_POS_BLOOD_VS_NAIVE_TONSIL_BCELL_UP	163	-0.44322	-1.94486	0	0.003662
GSE3982_MAST_CELL_VS_DC_DN	183	-0.53666	-1.94395	0	0.003664
GSE9650_EFFECTOR_VS_MEMORY_CD8_TCELL_DN	190	-0.40047	-1.94397	0	0.003668
GSE2770_IL12_AND_TGFB_ACT_VS_ACT_CD4_TCELL_6H_UP	173	-0.55503	-1.94419	0	0.003669
GSE4984_UNTREATED_VS_VEHICLE_CTRL_TREATED_DC_UP	138	-0.45738	-1.94434	0	0.003669
GSE26669_CD4_VS_CD8_TCELL_IN_MLR_DN	174	-0.4516	-1.94368	0	0.00368

GSE7768_OVA_ALONE_VS_OVA_WITH_LPS_IMMUNIZED_MOUSE_WHOLE_SPLEEN_6H_UP	153	-0.42757	-1.9431	0	0.00369
GSE19941_LPS_VS_LPS_AND_IL10_STIM_IL10_KO_MACROPHAGE_DN	147	-0.55448	-1.94333	0	0.003691
GSE37532_WT_VS_PPARG_KO_LN_TCONV_UP	177	-0.52135	-1.94354	0	0.003691
GSE29949_CD8_POS_DC_SPLEEN_VS_MONOCYTE_BONE_MARROW_UP	186	-0.50137	-1.94317	0	0.003692
GSE37301_CD4_TCELL_VS_GRANULOCYTE_MONOCYTE_PROGENITOR_DN	171	-0.59654	-1.94238	0	0.003701
GSE17721_CPG_VS_GARDIQUIMOD_6H_BMDC_UP	183	-0.47564	-1.94186	0	0.003705
GSE10240_CTRL_VS_IL17_STIM_PRIMARY_BRONCHIAL_EPITHELIAL_CELLS_DN	184	-0.44771	-1.94198	0	0.003707
GSE3982_EFF_MEMORY_VS_CENT_MEMORY_CD4_TCELL_UP	168	-0.52452	-1.9416	0	0.003707
GSE22432_CONVENTIONAL_CDC_VS_PLASMACYTOID_PDC_DN	181	-0.41444	-1.93938	0	0.003718
GSE19772_HCMV_INFL_VS_HCMV_INF_MONOCYTES_AND_PI3K_INHIBITION_DN	185	-0.43597	-1.93982	0	0.00372
GSE14415_ACT_TCONV_VS_ACT_NATURAL_TREG_UP	157	-0.53213	-1.93945	0	0.003721
GSE22886_DC_VS_MONOCYTE_UP	186	-0.47085	-1.93961	0	0.003722

GSE26351_UNSTIM_VS_WNT_PATHWAY_STIM_HEMATOPOIETIC_PROGENITORS_UP	173	-0.34756	-1.9395	0	0.003722
GSE14350_IL2RB_KO_VS_WT_TEFF_DN	183	-0.52084	-1.93988	0	0.003726
GSE20366_CD103_KLRG1_DP_VS_DN_TREG_DN	177	-0.48467	-1.94003	0	0.003728
GSE17721_LPS_VS_CPG_6H_BMDC_UP	184	-0.44441	-1.93873	0	0.003728
GSE37301_HEMATOPOIETIC_STEM_CELL_VS_PRO_BCELL_UP	180	-0.47531	-1.94023	0	0.003729
GSE21379_WT_VS_SAP_KO_TFH_CD4_TCELL_DN	179	-0.57985	-1.9403	0	0.003732
GSE4142_NAIVE_BCELL_VS_PLASMA_CELL_DN	186	-0.47623	-1.94033	0	0.003735
GSE27670_CTRL_VS_LMP1_TRANSDUCED_GC_BCELL_DN	186	-0.45939	-1.93763	0	0.003737
GSE27859_MACROPHAGE_VS_CD11C_INT_F480_HI_MACROPHAGE_DN	162	-0.3905	-1.94041	0	0.003738
GSE15330_LYMPHOID_MULTIPOTENT_VS_MEGAKARYOCYTE_ERYTHROID_PROGENITOR_DN	187	-0.4334	-1.93763	0	0.00374
GSE1740_UNSTIM_VS_IFNA_STIMULATED_MCSF_IFNG_DERIVED_MACROPHAGE_DN	169	-0.3538	-1.93764	0	0.003744
GSE23505_UNTREATED_VS_4DAY_IL6_IL1_TGFB_TREATED_CD4_TCELL_UP	183	-0.50349	-1.93839	0	0.003745

GSE3982_MAST_CELL_VS_CENT_MEMORY_CD4_TCELL_DN	174	-0.48389	-1.93795	0	0.003745
GSE5542_UNTREATED_VS_IFNG_TREATED_EPITHELIAL_CELLS_24H_DN	171	-0.47262	-1.93799	0	0.003747
GSE3982_MAST_CELL_VS_TH1_UP	182	-0.44206	-1.93826	0	0.003747
GSE22935_UNSTIM_VS_24H_MBOVIS_BCG_STIM_MACROPHAGE_UP	182	-0.49275	-1.93843	0	0.003748
GSE3691_IFN_PRODUCING_KILLER_DC_VS_CONVENTIONAL_DC_SPLEEN_UP	178	-0.36477	-1.93767	0	0.003749
GSE17580_TREG_VS_TEFF_S_MANSONI_INF_DN	179	-0.50832	-1.93801	0	0.00375
GSE24142_DN2_VS_DN3_THYMOCYTE_FETAL_DN	188	-0.52866	-1.93804	0	0.003751
GSE3920_UNTREATED_VS_IFNG_TREATED_FIBROBLAST_DN	172	-0.54426	-1.93709	0	0.003751
GSE3982_DC_VS_TH1_UP	184	-0.5432	-1.93715	0	0.003752
GSE21670_IL6_VS_TGFB_AND_IL6_TREATED_STAT3_KO_CD4_TCELL_DN	182	-0.47371	-1.937	0	0.003758
GSE17580_TREG_VS_TEFF_UP	177	-0.48429	-1.93586	0	0.003759
GSE3982_CTRL_VS_IGE_STIM_MAST_CELL_UP	185	-0.36027	-1.93563	0	0.003759

GSE33424_CD161_INT_VS_NEG_CD8_TCELL_DN	180	-0.41026	-1.93574	0	0.003759
GSE3982_EOSINOPHIL_VS_BASOPHIL_UP	187	-0.50043	-1.93591	0	0.003761
GSE14308_TH1_VS_NAIVE_CD4_TCELL_DN	169	-0.43344	-1.93661	0	0.003762
GSE1460_INTRATHYMIC_T_PROGENITOR_VS_NAIVE_CD4_TCELL_CORD_BLOOD_DN	183	-0.52763	-1.93623	0	0.003762
GSE40274_CTRL_VS_HELIOS_TRANSDUCED_ACTIVATED_CD4_TCELL_UP	151	-0.50587	-1.93632	0	0.003762
GSE14350_IL2RB_KO_VS_WT_TREG_DN	187	-0.53107	-1.93546	0	0.003762
GSE20366_TREG_VS_TCONV_DN	182	-0.54281	-1.93478	0	0.003763
GSE18804_SPLEEN_MACROPHAGE_VS_COLON_TUMORAL_MACROPHAGE_UP	183	-0.5365	-1.9354	0	0.003763
GSE7460_CTRL_VS_TGFB_TREATED_ACT_TCONV_UP	186	-0.5506	-1.93514	0	0.003763
GSE16450_CTRL_VS_IFNA_6H_STIM_IMMATURE_NEURON_CELL_LINE_DN	168	-0.56077	-1.93495	0	0.003763
GSE22611_UNSTIM_VS_6H_MDP_STIM_NOD2_TRANSDUCED_HEK293T_CELL_DN	179	-0.44618	-1.93439	0	0.003764
GSE37301_COMMON_LYMPHOID_PROGENITOR_VS_GRAN_MONO_PROGENITOR_DN	173	-0.52899	-1.93601	0	0.003765

GSE20366_TREG_VS_NAIVE_CD4_TCELL_UP	182	-0.47451	-1.93517	0	0.003766
GSE21670_STAT3_KO_VS_WT_CD4_TCELL_UP	184	-0.59272	-1.93496	0	0.003767
GSE30083_SP2_VS_SP4_THYMOCYTE_DN	181	-0.61399	-1.93522	0	0.00377
GSE4590_PRE_BCELL_VS_VPREB_POS_LARGE_PRE_BCELL_DN	149	-0.36365	-1.93375	0	0.003771
GSE39556_UNTREATED_VS_3H_POLYIC_INJ_MOUSE_CD8A_DC_UP	190	-0.60512	-1.93394	0	0.003771
GSE16450_IMMATURE_VS_MATURE_NEURON_CELL_LINE_UP	164	-0.59408	-1.9338	0	0.003772
GSE20366_EX_VIVO_VS_HOMEOSTATIC_CONVERSION_NAIVE_CD4_TCELL_UP	181	-0.4547	-1.9331	0	0.003789
GSE7852_LN_VS_THYMUS_TREG_DN	184	-0.42676	-1.93316	0	0.003789
GSE29618_LAIV_VS_TIV_FLU_VACCINE_DAY7_MONOCYTE_DN	180	-0.4527	-1.93297	0	0.003797
GSE7831_CPG_VS_INFLUENZA_STIM_PDC_1H_DN	195	-0.51902	-1.9324	0	0.003801
GSE45739_UNSTIM_VS_ACD3_ACD28_STIM_WT_CD4_TCELL_UP	185	-0.57194	-1.93236	0	0.003802
GSE25123_ROSIGLITAZONE_VS_IL4_AND_ROSIGLITAZONE_STIM_MACROPHAGE_DAY10_UP	176	-0.44002	-1.93252	0	0.003805

GSE2706_R848_VS_R848_AND_LPS_8H_STIM_DC_DN	164	-0.55113	-1.93172	0	0.003816
GSE1925_CTRL_VS_3H_IFNG_STIM_MACROPHAGE_UP	184	-0.40871	-1.93141	0	0.003817
GSE18791_CTRL_VS_NEWCASTLE_VIRUS_DC_16H_DN	172	-0.59516	-1.93125	0	0.003817
GSE30962_PRIMARY_VS_SECONDARY_CHRONIC_LCMV_INF_CD8_TCELL_DN	182	-0.55453	-1.93156	0	0.003818
GSE9239_CTRL_VS_TNF_INHIBITOR_TREATED_DC_UP	173	-0.45452	-1.93033	0	0.003856
GSE11973_MIR223_KOVS_WT_BONE_MARROW_NEUTROPHIL_UP	184	-0.45645	-1.93031	0	0.003857
GSE24634_IL4_VS_CTRL_TREATED_NAIVE_CD4_TCELL_DAY3_UP	189	-0.53196	-1.9304	0	0.003858
GSE360_L_DONOVANI_VS_M_TUBERCULOSIS_MAC_UP	186	-0.43955	-1.92974	0	0.003862
GSE360_L_DONOVANI_VS_B_MALAYI_LOW_DOSE_MAC_UP	188	-0.40093	-1.92999	0	0.003864
GSE17721_PAM3CSK4_VS_GADIQUIMOD_12H_BMDC_DN	190	-0.49471	-1.92953	0	0.003867
GSE3982_EFF_MEMORY_CD4_TCELL_VS_NKCELL_DN	180	-0.54479	-1.92866	0	0.003904
GSE17721_CPG_VS_GARDIQUIMOD_16H_BMDC_UP	183	-0.52008	-1.92848	0	0.003904

GSE30153_LUPUS_VS_HEALTHY_DONOR_BCELL_UP	189	-0.48473	-1.92852	0	0.003906
GSE37533_UNTREATED_VS_PIOGLIZATONE_TREATED_CD4_TCELL_FOXP3_TRASDUCED_CD4_TCELL_DN	150	-0.49532	-1.92824	0	0.003911
GSE14415_ACT_VS_CTRL_NATURAL_TREG_DN	156	-0.41767	-1.92795	0	0.003911
GSE41867_DAY6_VS_DAY8_LCMV_CLONE13_EFFECTOR_CD8_TCELL_UP	178	-0.48803	-1.92778	0	0.003913
GSE28783_CTRL_ANTI_MIR_VS_UNTREATED_ATHEROSCLEROSIS_MACROPHAGE_UP	183	-0.48753	-1.92805	0	0.003914
GSE3039_CD4_TCELL_VS_ALPHABETA_CD8_TCELL_UP	179	-0.58718	-1.92751	0	0.003916
GSE29164_DAY3_VS_DAY7_CD8_TCELL_TREATED_MELANOMA_UP	182	-0.50007	-1.92717	0	0.00392
GSE23308_CTRL_VS_CORTICOSTERONE_TREATED_MACROPHAGE_MINERALCORTICOID_REC_KO_UP	181	-0.32335	-1.92718	0	0.003923
GSE40666_WT_VS_STAT1_KO_CD8_TCELL_UP	182	-0.51586	-1.92691	0	0.00393
GSE9650_EXHAUSTED_VS_MEMORY_CD8_TCELL_UP	183	-0.54913	-1.92683	0	0.003931
GSE5589_LPS_VS_LPS_AND_IL10_STIM_IL10_KO_MACROPHAGE_180MIN_DN	181	-0.4517	-1.92648	0	0.003941
GSE7219_UNSTIM_VS_LPS_AND_ANTI_CD40_STIM_DC_UP	166	-0.58652	-1.92641	0	0.003941

GSE45739_UNSTIM_VS_ACD3_ACD28_STIM_NRAS_KO_CD4_TCELL_DN	178	-0.66744	-1.92575	0	0.003958
GSE11961_FOLLICULAR_BCELL_VS_PLASMA_CELL_DAY7_DN	177	-0.42609	-1.92594	0	0.003958
GSE24210_RESTING_TREG_VS_TCONV_UP	180	-0.46706	-1.92542	0	0.003965
GSE16385_ROSIGLITAZONE_IFNG_TNF_VS_IL4_STIM_MACROPHAGE_UP	190	-0.5407	-1.92548	0	0.003967
GSE3982_BCELL_VS_BASOPHIL_DN	182	-0.52611	-1.9251	0	0.003985
GSE32986_GMCSF_VS_GMCSF_AND_CURDLAN_LOWDOSE_STIM_DC_DN	171	-0.51818	-1.92499	0	0.00399
GSE17301_CTRL_VS_48H_IFNA2_STIM_CD8_TCELL_DN	170	-0.4872	-1.9245	0	0.004
GSE3982_DC_VS_BCELL_DN	175	-0.4853	-1.9244	0	0.004001
GSE35825_IFNA_VS_IFNG_STIM_MACROPHAGE_DN	178	-0.4101	-1.92254	0	0.004004
GSE19941_LPS_VS_LPS_AND_IL10_STIM_IL10_KO_NFKBP50_KO_MACROPHAGE_DN	182	-0.54895	-1.92274	0	0.004005
GSE30962_PRIMARY_VS_SECONDARY_ACUTE_LCMV_INF_CD8_TCELL_DN	179	-0.47999	-1.92299	0	0.004007
GSE4142_NAIVE_VS_GC_BCELL_UP	183	-0.49872	-1.9228	0	0.004009

GSE7764_NKCELL_VS_SPLENOCYTE_UP	186	-0.54274	-1.92233	0	0.004009
GSE360_T_GONDII_VS_M_TUBERCULOSIS_DC_UP	184	-0.46857	-1.92311	0	0.00401
GSE3720_VD1_VS_VD2_GAMMADELTA_TCELL_WITH_LPS_STIM_DN	176	-0.41146	-1.92318	0	0.004011
GSE45365_NK_CELL_VS_CD8A_DC_UP	191	-0.4115	-1.92413	0	0.004013
GSE23502_BM_VS_COLON_TUMOR_MYELOID_DERIVED_SUPPRESSOR_CELL_UP	189	-0.53827	-1.92364	0	0.004013
GSE7348_UNSTIM_VS_LPS_STIM_MACROPHAGE_DN	161	-0.56632	-1.92319	0	0.004014
GSE29164_CD8_TCELL_VS_CD8_TCELL_AND_IL12_TREATED_MELANOMA_DAY3_DN	189	-0.46744	-1.9237	0	0.004015
GSE20366_CD103_POS_VS_NEG_TREG_KLRG1NEG_DN	189	-0.52232	-1.92373	0	0.004019
GSE32423_IL7_VS_IL4_MEMORY_CD8_TCELL_UP	183	-0.41753	-1.9239	0	0.004019
GSE7852_LN_VS_FAT_TCONV_UP	174	-0.41253	-1.92184	0	0.00402
GSE29618_PRE_VS_DAY7_POST_TIV_FLU_VACCINE_MDC_DN	173	-0.44861	-1.92135	0	0.004032
GSE7460_TCONV_VS_TREG_THYMUS_UP	191	-0.49293	-1.92128	0	0.004034

GSE21063_CTRL_VS_ANTI_IGM_STIM_BCELL_NFATC1_KO_16H_UP	179	-0.53216	-1.92143	0	0.004037
GSE22282_HYPOXIA_VS_NORMOXIA_MYELOID_DC_DN	174	-0.52707	-1.92095	0	0.00405
GSE12845_IGD_NEG_BLOOD_VS_PRE_GC_TONSIL_BCELL_UP	175	-0.42532	-1.92067	0	0.004053
GSE3920_UNTREATED_VS_IFNB_TREATED_ENDOTHELIAL_CELL_UP	178	-0.43691	-1.91979	0	0.004057
GSE36009_WT_VS_NLRP10_KO_DC_UP	181	-0.46061	-1.91988	0	0.004057
GSE22025_PROGESTERONE_VS_TGFB1_AND_PROGESTERONE_TREATED_CD4_TCELL_DN	187	-0.51587	-1.92018	0	0.004058
GSE17721_CPG_VS_GARDIQUIMOD_24H_BMDC_DN	180	-0.47104	-1.92023	0	0.004058
GSE17721_POLYIC_VS_PAM3CSK4_6H_BMDC_UP	190	-0.42285	-1.91966	0	0.004058
GSE9946_IMMATURE_VS_MATURE_STIMULATORY_DC_UP	130	-0.4479	-1.92024	0	0.004061
GSE3982_MAC_VS_TH2_UP	188	-0.48226	-1.92024	0	0.004064
GSE19888_ADENOSINE_A3R_INH_VS_ACT_IN_MAST_CELL_DN	181	-0.60421	-1.91874	0	0.004095
GSE7219_WT_VS_NIK_NFKB2_KO_LPS_AND_ANTI_CD40_STIM_DC_DN	183	-0.52675	-1.91723	0	0.00412

GSE34515_CD16_POS_MONOCYTE_VS_DC_UP	183	-0.39083	-1.91739	0	0.004122
GSE21670_UNTREATED_VS_IL6_TREATED_STAT3_KO_CD4_TCELL_UP	177	-0.62512	-1.9178	0	0.004125
GSE18281_CORTEX_VS_MEDULLA_THYMUS_DN	165	-0.47034	-1.9168	0	0.004129
GSE13522_CTRL_VS_T_CRUZI_G_STRAIN_INF_SKIN_UP	179	-0.45467	-1.9168	0	0.004132
GSE18893_CTRL_VS_TNF_TREATED_TCONV_2H_UP	181	-0.36932	-1.91648	0	0.004133
GSE2585_THYMIC_DC_VS_THYMIC_MACROPHAGE_DN	180	-0.4401	-1.91683	0	0.004134
GSE25088_CTRL_VS_IL4_AND_ROSIGLITAZONE_STIM_MACROPHAGE_UP	167	-0.49125	-1.91633	0	0.004136
GSE40068_CXCR5POS_BCL6POS_TFH_VS_CXCR5NEG_BCL6NEG_CD4_TCELL_UP	178	-0.46701	-1.91615	0	0.004148
GSE37533_PPARG2_FOXP3_VS_FOXP3_TRANSDUCED_CD4_TCELL_PIOGLITAZONE_TREATED_DN	174	-0.40507	-1.91494	0	0.004161
GSE10325_CD4_TCELL_VS_BCELL_UP	185	-0.63841	-1.91508	0	0.004162
GSE22589_HEALTHY_VS_SIV_INFECTED_DC_DN	187	-0.50488	-1.91585	0	0.004164
GSE29618_MONOCYTE_VS_PDC_DN	179	-0.49324	-1.91485	0	0.004165

GSE17974_CTRL_VS_ACT_IL4_AND_ANTI_IL12_4H_CD4_TCELL_UP	170	-0.52562	-1.91533	0	0.004165
GSE29618_PDC_VS_MDC_DN	191	-0.65238	-1.91459	0	0.004166
GSE7768_OVA_ALONE_VS_OVA_WITH_LPS_IMMUNIZED_MOUSE_WHOLE_SPLEEN_6H_DN	161	-0.57526	-1.91558	0	0.004167
GSE1925_CTRL_VS_IFNG_PRIMED_MACROPHAGE_24H_IFNG_STIM_UP	183	-0.45717	-1.91566	0	0.004167
GSE21546_WT_VS_SAP1A_KO_AND_ELK1_KO_DP_THYMOCYTES_DN	184	-0.53456	-1.91568	0	0.004169
GSE21379_WT_VS_SAP_KO_CD4_TCELL_DN	177	-0.55611	-1.91435	0	0.004174
GSE41867_NAIVE_VS_DAY6_LCMV_ARMSTRONG_EFFECTOR_CD8_TCELL_DN	178	-0.54268	-1.91404	0	0.004175
GSE17974_0H_VS_4H_IN_VITRO_ACT_CD4_TCELL_UP	170	-0.50072	-1.91418	0	0.004178
GSE13173_UNTREATED_VS_IL12_TREATED_ACT_CD8_TCELL_DN	126	-0.40491	-1.91386	0	0.004185
GSE3691_IFN_PRODUCING_KILLER_DC_VS_CONVENTIONAL_DC_SPLEEN_DN	180	-0.53646	-1.91368	0	0.004192
GSE17721_POLYIC_VS_GARDIQUIMOD_12H_BMDC_UP	178	-0.45193	-1.9127	0	0.004202
GSE3982_MAC_VS_CENT_MEMORY_CD4_TCELL_DN	176	-0.45802	-1.91281	0	0.004204

GSE30083_SP1_VS_SP3_THYMOCYTE_DN	174	-0.54209	-1.91295	0	0.004204
GSE17721_LPS_VS_GARDIQUIMOD_0.5H_BMDC_DN	182	-0.41414	-1.91231	0	0.004207
GSE14308_TH17_VS_INDUCED_TREG_UP	183	-0.38863	-1.91296	0	0.004208
GSE14415_INDUCED_TREG_VS_TCONV_DN	148	-0.52266	-1.91256	0	0.004208
GSE411_WT_VS_SOCS3_KO_MACROPHAGE_IL6_STIM_400MIN_UP	185	-0.4791	-1.91247	0	0.00421
GSE6674_UNSTIM_VS_CPG_STIM_BCELL_DN	152	-0.3954	-1.91257	0	0.004212
GSE3982_MEMORY_CD4_TCELL_VS_BCELL_UP	177	-0.60883	-1.9119	0	0.004224
GSE13493_DP_VS_CD8POS_THYMOCYTE_UP	178	-0.44143	-1.91171	0	0.004229
GSE19941_UNSTIM_VS_LPS_AND_IL10_STIM_IL10_KO_MACROPHAGE_UP	181	-0.55011	-1.9117	0	0.004229
GSE20366_TREG_VS_NAIVE_CD4_TCELL_DEC205_CONVERSION_UP	188	-0.53924	-1.91115	0	0.00424
GSE3982_EOSINOPHIL_VS_BCELL_DN	178	-0.3511	-1.91092	0	0.004245
GSE44955_MCSF_VS_MCSF_AND_IL27_STIM_MACROPHAGE_DN	175	-0.41692	-1.91096	0	0.004246

GSE36392_EOSINOPHIL_VS_NEUTROPHIL_IL25_TREATED_LUNG_UP	187	-0.52852	-1.91064	0	0.004251
GSE28737_WT_VS_BCL6_KO_FOLLICULAR_BCELL_DN	186	-0.49456	-1.91029	0	0.004253
GSE23114_PERITONEAL_CAVITY_B1A_BCELL_VS_SPLEEN_BCELL_IN_SLE2C1_MOUSE_UP	186	-0.52575	-1.91056	0	0.004254
GSE411_WT_VS_SOCS3_KO_MACROPHAGE_DN	185	-0.32263	-1.90973	0	0.004255
GSE21033_CTRL_VS_POLYIC_STIM_DC_6H_DN	145	-0.47498	-1.91007	0	0.004256
GSE11961_FOLLICULAR_BCELL_VS_MARGINAL_ZONE_BCELL_UP	177	-0.54259	-1.9099	0	0.004256
GSE32034_UNTREATED_VS_ROSIGLIZATONE_TREATED_LY6C_LOW_MONOCYTE_DN	176	-0.52683	-1.91014	0	0.004257
GSE11057_NAIVE_VS_EFF_MEMORY_CD4_TCELL_UP	166	-0.53876	-1.91015	0	0.004257
GSE17721_POLYIC_VS_GARDIQUIMOD_0.5H_BMDC_DN	180	-0.35114	-1.90995	0	0.004257
GSE17721_LPS_VS_PAM3CSK4_24H_BMDC_DN	177	-0.39415	-1.90955	0	0.004258
GSE13522_CTRL_VS_T_CRUZI_Y_STRAIN_INF_SKIN_IFNAR_KO_DN	184	-0.52844	-1.90904	0	0.004262
GSE20366_TREG_VS_NAIVE_CD4_TCELL_DN	182	-0.5691	-1.90926	0	0.004263

GSE36476_CTRL_VS_TSST_ACT_40H_MEMORY_CD4_TCELL_YOUNG_UP	179	-0.50232	-1.9093	0	0.004265
GSE17721_LPS_VS_CPG_2H_BMDC_DN	180	-0.48233	-1.90876	0	0.004268
GSE17721_12H_VS_24H_POLYIC_BMDC_DN	177	-0.31562	-1.9093	0	0.004268
GSE13946_CTRL_VS_DSS_COLITIS_GD_TCELL_FROM_COLON_UP	167	-0.53926	-1.90861	0	0.00427
GSE21546_WT_VS_SAP1A_KO_ANTI_CD3_STIM_DP_THYMOCYTES_UP	180	-0.49572	-1.90837	0	0.004272
GSE20715_0H_VS_48H_OZONE_TLR4_KO_LUNG_UP	188	-0.5755	-1.90868	0	0.004273
GSE21927_SPLEEN_VS_4T1_TUMOR_MONOCYTE_BALBC_UP	159	-0.4746	-1.90794	0	0.00428
GSE13547_WT_VS_ZFX_KO_BCELL_ANTI_IGM_STIM_12H_DN	142	-0.52618	-1.90804	0	0.00428
GSE2770_UNTREATED_VS_IL4_TREATED_ACT_CD4_TCELL_48H_UP	187	-0.37727	-1.90787	0	0.004284
GSE16385_ROSIGLITAZONE_IL4_VS_IFNG_TNF_STIM_MACROPHAGE_DN	177	-0.59132	-1.90745	0	0.004291
GSE17186_MEMORY_VS_CD21LOW_TRANSITIONAL_BCELL_DN	178	-0.37952	-1.90746	0	0.004294
GSE17721_POLYIC_VS_GARDIQUIMOD_0.5H_BMDC_UP	185	-0.53133	-1.90749	0	0.004295

GSE29164_DAY3_VS_DAY7_CD8_TCELL_TREATED_MELANOMA_DN	172	-0.44655	-1.90649	0	0.004301
GSE20366_CD103_POS_VS_CD103_KLRG1_DP_TREG_DN	186	-0.52909	-1.90638	0	0.004303
GSE10240_CTRL_VS_IL22_STIM_PRIMARY_BRONCHIAL_EPITHELIAL_CELLS_UP	182	-0.50781	-1.90649	0	0.004304
GSE27786_CD8_TCELL_VS_ERYTHROBLAST_UP	182	-0.29093	-1.90606	0	0.004307
GSE27786_CD4_TCELL_VS_NKTCELL_DN	184	-0.40774	-1.9068	0	0.004307
GSE17721_PAM3CSK4_VS_GADIQUIMOD_8H_BMDC_UP	169	-0.35154	-1.90682	0	0.004311
GSE26928_NAIVE_VS_EFF_MEMORY_CD4_TCELL_DN	176	-0.44433	-1.90684	0	0.004314
GSE11961_PLASMA_CELL_DAY7_VS_MEMORY_BCELL_DAY40_UP	184	-0.46473	-1.90702	0	0.004316
GSE29949_MICROGLIA_BRAIN_VS_CD8_POS_DC_SPLEEN_UP	187	-0.45663	-1.90685	0	0.004316
GSE4142_NAIVE_BCELL_VS_PLASMA_CELL_UP	182	-0.41617	-1.90564	0	0.00432
GSE15330_MEGAKARYOCYTE_ERYTHROID_VS_GRANULOCYTE_MONOCYTE_PROGENITOR_DN	188	-0.3885	-1.90535	0	0.004333
GSE29618_PRE_VS_DAY7_FLU_VACCINE_MONOCYTE_DN	176	-0.38469	-1.90523	0	0.004335

GSE13738_RESTING_VS_BYSTANDER_ACTIVATED_CD4_TCELL_UP	152	-0.54175	-1.90355	0	0.004336
GSE10240_IL22_VS_IL22_AND_IL17_STIM_PRIMARY_BRONCHIAL_EPITHELIAL_CELLS_DN	178	-0.48802	-1.90357	0	0.004339
GSE17721_LPS_VS_CPG_4H_BMDC_UP	188	-0.42104	-1.90334	0	0.004339
GSE37301_COMMON_LYMPHOID_PROGENITOR_VS_RAG2_KO_NK_CELL_DN	181	-0.54374	-1.9036	0	0.00434
GSE21670_UNTREATED_VS_IL6_TREATED_CD4_TCELL_UP	171	-0.39068	-1.90422	0	0.00434
GSE5589_LPS_VS_LPS_AND_IL10_STIM_MACROPHAGE_45MIN_UP	188	-0.49676	-1.90436	0	0.004342
GSE17186_BLOOD_VS_CORD_BLOOD_CD21HIGH_TRANSITIONAL_BCELL_DN	180	-0.45014	-1.90427	0	0.004342
GSE32901_NAIVE_VS_TH1_CD4_TCELL_DN	161	-0.56455	-1.90198	0	0.004342
GSE36009_UNSTIM_VS_LPS_STIM_DC_UP	177	-0.44802	-1.90193	0	0.004342
GSE43863_DAY6_EFF_VS_DAY150_MEM_TH1_CD4_TCELL_UP	185	-0.56034	-1.90452	0	0.004343
GSE2585_THYMIC_DC_VS_MTEC_DN	183	-0.46204	-1.90309	0	0.004343
GSE18791_UNSTIM_VS_NEWCATSLE_VIRUS_DC_18H_DN	159	-0.61159	-1.90198	0	0.004345

GSE45365_HEALTHY_VS_MCMV_INFECTION_CD8A_DC_DN	185	-0.4798	-1.90315	0	0.004346
GSE1460_DP_VS_CD4_THYMOCYTE_UP	178	-0.40213	-1.90462	0	0.004346
GSE28726_NAIVE_CD4_TCELL_VS_NAIVE_NKTCELL_DN	190	-0.55411	-1.90503	0	0.004346
GSE3982_EOSINOPHIL_VS_EFF_MEMORY_CD4_TCELL_DN	180	-0.37227	-1.90219	0	0.004347
GSE15930_NAIVE_VS_24H_IN_VITRO_STIM_CD8_TCELL_UP	189	-0.47238	-1.90201	0	0.004347
GSE11057_EFF_MEM_VS_CENT_MEM_CD4_TCELL_UP	174	-0.52006	-1.90225	0	0.004348
GSE31082_DN_VS_CD4_SP_THYMOCYTE_DN	184	-0.41396	-1.90474	0	0.004348
GSE3039_NKT_CELL_VS_ALPHAALPHA_CD8_TCELL_DN	185	-0.7088	-1.90374	0	0.004348
GSE37301_HEMATOPOIETIC_STEM_CELL_VS_GRAN_MONO_PROGENITOR_UP	149	-0.4921	-1.90395	0	0.004348
GSE23114_WT_VS_SLE2C1_MOUSE_PERITONEAL_CAVITY_B1A_BCELL_UP	170	-0.44018	-1.90376	0	0.00435
GSE22886_NAIVE_CD4_TCELL_VS_NKCELL_UP	175	-0.51065	-1.9048	0	0.00435
GSE3982_MEMORY_CD4_TCELL_VS_TH2_UP	171	-0.50178	-1.90274	0	0.004351

GSE14769_UNSTIM_VS_80MIN_LPS_BMDM_UP	174	-0.31963	-1.90285	0	0.004351
GSE19888_CTRL_VS_TCELL_MEMBRANES_ACT_MAST_CELL_PRETREAT_A3R_INH_DN	189	-0.61548	-1.90286	0	0.004352
GSE46468_LUNG_INNATE_LYMPHOID_CELL_VS_SPLEEN_CD4_TCELL_UP	161	-0.36351	-1.90162	0	0.004353
GSE14415_FOXP3_KO_NATURAL_TREG_VS_TCONV_UP	159	-0.46008	-1.90254	0	0.004354
GSE27786_BCELL_VS_CD4_TCELL_DN	171	-0.46331	-1.90261	0	0.004354
GSE41867_NAIVE_VS_DAY8_LCMV_ARMSTRONG_EFFECTOR_CD8_TCELL_UP	176	-0.40354	-1.90238	0	0.004355
GSE3982_MAC_VS_NKCELL_DN	178	-0.48992	-1.90122	0	0.004357
GSE6875_TCONV_VS_FOXP3_KO_TREG_UP	179	-0.51526	-1.90123	0	0.00436
KAECH_NAIVE_VS_DAY8_EFF_CD8_TCELL_UP	188	-0.36008	-1.90097	0	0.004361
GSE11057_EFF_MEM_VS_CENT_MEM_CD4_TCELL_DN	159	-0.56362	-1.90071	0	0.004374
GSE2585_CTEC_VS_THYMIC_MACROPHAGE_UP	179	-0.49935	-1.90045	0	0.004375
GSE17721_CTRL_VS_CPG_12H_BMDC_DN	179	-0.45809	-1.9002	0	0.004376

GSE17721_0.5H_VS_12H_PAM3CSK4_BMDC_UP	185	-0.39529	-1.90014	0	0.004376
GSE15930_STIM_VS_STIM_AND_TRICHOSTATINA_72H_CD8_T_CELL_UP	177	-0.3727	-1.90046	0	0.004377
GSE7852_LN_VS_THYMUS_TCONV_UP	183	-0.47534	-1.90055	0	0.004378
GSE25123_WT_VS_PPARG_KO_MACROPHAGE_IL4_STIM_DN	182	-0.56986	-1.89979	0	0.004384
GSE22886_NAIVE_CD8_TCELL_VS_MONOCYTE_UP	190	-0.68965	-1.89965	0	0.004386
GSE2770_UNTREATED_VS_ACT_CD4_TCELL_48H_DN	164	-0.48976	-1.89981	0	0.004387
GSE7852_TREG_VS_TCONV_LN_UP	183	-0.55167	-1.89915	0	0.004396
GSE11864_CSF1_VS_CSF1_IFNG_IN_MAC_UP	165	-0.43381	-1.89886	0	0.004408
GSE21360_NAIVE_VS_PRIMARY_MEMORY_CD8_TCELL_UP	163	-0.55675	-1.89822	0	0.004429
GSE339_CD8POS_VS_CD4CD8DN_DC_DN	192	-0.45364	-1.89826	0	0.00443
GSE2405_HEAT_KILLED_LYSATE_VS_LIVE_A_PHAGOCYTOPHILUM_STIM_NEUTROPHIL_9H_DN	185	-0.66324	-2.47095	0	0.004435
GSE7460_TCONV_VS_TREG_LN_DN	188	-0.54466	-1.89773	0	0.004436

GSE7460_TREG_VS_TCONV_ACT_DN	173	-0.52452	-1.89782	0	0.004437
GSE17721_LPS_VS_GARDIQUIMOD_24H_BMDC_UP	182	-0.48269	-1.89773	0	0.004439
GSE15330_WT_VS_IKAROS_KO_GRANULOCYTE_MONOCYTE_PROGENITOR_DN	187	-0.43399	-1.89792	0	0.00444
GSE17721_CPG_VS_GARDIQUIMOD_0.5H_BMDC_UP	184	-0.44969	-1.89799	0	0.004442
GSE25088_ROSIGLITAZONE_VS_IL4_AND_ROSIGLITAZONE_STIM_MACROPHAGE_DAY10_UP	177	-0.3448	-1.89702	0	0.004452
GSE24081_CONTROLLER_VS_PROGRESSOR_HIV_SPECIFIC_CD8_TCELL_UP	182	-0.50812	-1.89709	0	0.004455
GSE25123_WT_VS_PPARG_KO_MACROPHAGE_IL4_AND_ROSIGLITAZONE_STIM_DN	175	-0.44631	-1.89649	0	0.00448
GSE24634_IL4_VS_CTRL_TREATED_NAIVE_CD4_TCELL_DAY10_UP	181	-0.57583	-1.89637	0	0.004481
GSE27670_BLIMP1_VS_LMP1_TRANSDUCED_GC_BCELL_DN	185	-0.3315	-1.89591	0	0.004493
GSE37301_HEMATOPOIETIC_STEM_CELL_VS_COMMON_LYMPHOID_PROGENITOR_DN	188	-0.44246	-1.89479	0	0.004506
GSE29164_UNTREATED_VS_CD8_TCELL_AND_IL12_TREATED_MELANOMA_DAY3_UP	173	-0.42177	-1.89523	0	0.004508
GSE17721_CTRL_VS_POLYIC_1H_BMDC_DN	187	-0.50196	-1.89485	0	0.004508

GSE3982_EOSINOPHIL_VS_BASOPHIL_DN	173	-0.33603	-1.89525	0	0.004511
GSE19888_ADENOSINE_A3R_INH_VS_INH_PRETREAT_AND_ACT_WITH_TCELL_MEMBRANES_MAST_CELL_UP	173	-0.50131	-1.89506	0	0.004511
GSE11924_TFH_VS_TH1_CD4_TCELL_UP	187	-0.35929	-1.89444	0	0.004518
GSE7831_UNSTIM_VS_INFLUENZA_STIM_PDC_4H_UP	188	-0.48354	-1.89451	0	0.004518
GSE21063_CTRL_VS_ANTI_IGM_STIM_BCELL_3H_UP	162	-0.57757	-1.8939	0	0.004533
GSE13306_TREG_VS_TCONV_LAMINA_PROPRIA_UP	187	-0.4746	-1.89337	0	0.004549
GSE17974_IL4_AND_ANTI_IL12_VS_UNTREATED_12H_ACT_CD4_TCELL_DN	169	-0.61534	-1.89295	0	0.004551
GSE17721_POLYIC_VS_CPG_1H_BMDC_DN	191	-0.4588	-1.89338	0	0.004552
GSE3920_IFNA_VS_IFNB_TREATED_ENDOTHELIAL_CELL_UP	160	-0.51512	-1.89299	0	0.004553
GSE32986_CURDLAN_HIGHDOSE_VS_GMCSF_AND_CURDLAN_HIGHDOSE_STIM_DC_UP	178	-0.38735	-1.8926	0	0.004557
GSE3982_MAST_CELL_VS_NKCELL_DN	188	-0.5541	-1.89266	0	0.004558
GSE557_WT_VS_CIITA_KO_DC_UP	176	-0.37605	-1.892	0	0.004577

GSE2405_S_AUREUS_VS_A_PHAGOCYTOPHILUM_NEUTROPHIL_UP	185	-0.58246	-1.89186	0	0.004578
GSE40666_UNTREATED_VS_IFNA_STIM_STAT4_KO_EFFECTOR_CD8_TCELL_90MIN_DN	178	-0.50824	-1.89159	0	0.004585
GSE18893_CTRL_VS_TNF_TREATED_TREG_24H_DN	168	-0.42543	-1.89093	0	0.004599
GSE22886_NAIVE_BCELL_VS_MONOCYTE_UP	171	-0.63341	-1.8903	0	0.004614
GSE17721_LPS_VS_GARDIQUIMOD_2H_BMDC_UP	187	-0.50257	-1.8904	0	0.004616
GSE29618_MONOCYTE_VS_PDC_DAY7_FLU_VACCINE_DN	179	-0.49298	-1.89048	0	0.004616
GSE7852_TREG_VS_TCONV_FAT_UP	188	-0.47028	-1.8902	0	0.004617
GSE27291_6H_VS_7D_STIM_GAMMADELTA_TCELL_DN	151	-0.49048	-1.88962	0	0.004618
GSE8921_UNSTIM_VS_TLR1_2_STIM_MONOCYTE_12H_UP	179	-0.41073	-1.8905	0	0.004619
GSE2935_UV_INACTIVATED_VS_LIVE_SENDAI_VIRUS_INF_MACROPHAGE_UP	168	-0.53155	-1.88963	0	0.00462
GSE29617_CTRL_VS_DAY3_TIV_FLU_VACCINE_PBMC_2008_UP	158	-0.40728	-1.88973	0	0.004622
GSE2770_TGFB_AND_IL4_VS_IL12_TREATED_ACT_CD4_TCELL_48H_UP	176	-0.38293	-1.88976	0	0.004623

GSE14699_NAIVE_VS_DELETIONAL_TOLERANCE_CD8_TCELL_UP	139	-0.54455	-1.88984	0	0.004626
GSE17721_POLYIC_VS_PAM3CSK4_24H_BMDC_UP	182	-0.39117	-1.88883	0	0.00464
GSE40274_CTRL_VS_FOXP3_AND_PBX1_TRANSDUCED_ACTIVATED_CD4_TCELL_UP	152	-0.54393	-1.88871	0	0.004642
GSE24210_TCONV_VS_TREG_UP	175	-0.47113	-1.88884	0	0.004643
GSE30971_2H_VS_4H_LPS_STIM_MACROPHAGE_WBP7_KO_DN	174	-0.50331	-1.8889	0	0.004645
GSE12845_IGD_NEG_BLOOD_VS_NAIVE_TONSIL_BCELL_DN	181	-0.41127	-1.88835	0	0.004654
GSE19941_UNSTIM_VS_LPS_STIM_IL10_KO_MACROPHAGE_DN	179	-0.44995	-1.88759	0	0.004667
GSE27786_LSK_VS_LIN_NEG_CELL_UP	179	-0.38083	-1.88799	0	0.004668
GSE10211_UV_INACT_SENDAI_VS_LIVE_SENDAI_VIRUS_TRACHEAL_EPITHELIAL_CELLS_UP	141	-0.4741	-1.88706	0	0.004687
GSE17974_IL4_AND_ANTI_IL12_VS_UNTREATED_48H_ACT_CD4_TCELL_UP	162	-0.5432	-1.88662	0	0.004702
GSE17721_CTRL_VS_POLYIC_12H_BMDC_DN	177	-0.45402	-1.88623	0	0.004712
GSE22033_UNTREATED_VS_ROSIGLITAZONE_TREATED_MEF_DN	179	-0.43872	-1.88605	0	0.004715

GSE17721_LPS_VS_PAM3CSK4_2H_BMDC_UP	185	-0.48834	-1.88597	0	0.004717
GSE15735_CTRL_VS_HDAC_INHIBITOR_TREATED_CD4_TCELL_12H_UP	174	-0.38313	-1.88563	0	0.004721
GSE40666_WT_VS_STAT4_KO_CD8_TCELL_WITH_IFNA_STIM_90MIN_DN	182	-0.42444	-1.8854	0	0.004721
GSE360_HIGH_VS_LOW_DOSE_B_MALAYI_MAC_DN	190	-0.43459	-1.88572	0	0.004722
GSE21033_1H_VS_12H_POLYIC_STIM_DC_UP	171	-0.48009	-1.88541	0	0.004724
GSE3920_IFNA_VS_IFNG_TREATED_FIBROBLAST_DN	177	-0.4862	-1.88549	0	0.004725
GSE17186_CD21LOW_VS_CD21HIGH_TRANSITIONAL_BCELL_CORD_BLOOD_UP	183	-0.35214	-1.88572	0	0.004725
GSE4590_PRE_BCELL_VS_VPREB_POS_LARGE_PRE_BCELL_UP	151	-0.53623	-1.88519	0	0.004728
GSE3203_INFLUENZA_INF_VS_IFNB_TREATED_LN_BCELL_DN	173	-0.41644	-1.88477	0	0.004749
GSE11961_MEMORY_BCELL_DAY7_VS_PLASMA_CELL_DAY7_UP	181	-0.46652	-1.88469	0	0.004749
GSE14415_NATURAL_TREG_VS_FOXP3_KO_NATURAL_TREG_DN	145	-0.47976	-1.88464	0	0.004749
GSE46242_TH1_VS_ANERGIC_TH1_CD4_TCELL_WITH_EGR2_DELETED_UP	173	-0.50456	-1.88448	0	0.004761

GSE27786_NKCELL_VS_NKTCELL_DN	178	-0.43267	-1.88436	0	0.004765
GSE22045_TREG_VS_TCONV_DN	159	-0.45433	-1.88421	0	0.004773
GSE25085_FETAL_LIVER_VS_FETAL_BM_SP4_THYMIC_IMPLANT_DN	186	-0.53212	-1.88399	0	0.004773
GSE21927_SPLEEN_MONOCYTE_VS_GMCSF_GCSF_BONE_MARROW_UP	151	-0.49515	-1.88413	0	0.004774
GSE43863_LY6C_INT_CXCR5POS_VS_LY6C_LOW_CXCR5NEG_EFFECTOR_CD4_TCELL_UP	183	-0.50551	-1.88371	0	0.004776
GSE4984_LPS_VS_VEHICLE_CTRL_TREATED_DC_UP	172	-0.51871	-1.88339	0	0.004779
GSE25123_CTRL_VS_IL4_STIM_PPARG_KO_MACROPHAGE_DN	178	-0.52203	-1.88319	0	0.004782
GSE21063_WT_VS_NFATC1_KO_3H_ANTI_IGM_STIM_BCELL_UP	182	-0.53145	-1.8834	0	0.004783
GSE12366_NAIVE_VS_MEMORY_BCELL_UP	161	-0.55255	-1.88324	0	0.004783
GSE21063_CTRL_VS_ANTI_IGM_STIM_BCELL_8H_UP	147	-0.5922	-1.88256	0	0.004818
GSE22886_UNSTIM_VS_IL15_STIM_NKCELL_UP	171	-0.51224	-1.88259	0	0.00482
GSE15930_NAIVE_VS_24H_IN_VITRO_STIM_IL12_CD8_TCELL_UP	189	-0.46217	-1.88244	0	0.00482

GSE22611_NOD2_VS_CTRL_TRANSDUCED_HEK293T_CELL_DN	185	-0.43302	-1.8826	0	0.004823
GSE19941_IL10_KO_VS_IL10_KO_AND_NFKBP50_KO_LPS_STIM_MACROPHAGE_UP	186	-0.48604	-1.88209	0	0.00483
GSE40277_EOS_AND_LEF1_TRANSDUCED_VS_GATA1_AND_SATB1_TRANSDUCED_CD4_TCELL_UP	185	-0.45614	-1.88195	0	0.004833
GSE22501_PERIPHERAL_BLOOD_VS_CORD_BLOOD_TREG_DN	184	-0.51351	-1.88168	0	0.004848
GSE17721_CTRL_VS_POLYIC_6H_BMDC_DN	186	-0.45943	-1.88134	0	0.004862
GSE32423_MEMORY_VS_NAIVE_CD8_TCELL_UP	182	-0.52875	-1.88138	0	0.004864
GSE43955_TH0_VS_TGFB_IL6_TH17_ACT_CD4_TCELL_30H_DN	184	-0.42564	-1.88047	0	0.004885
GSE36527_CD62L_HIGH_CD69_NEG_VS_CD62L_LOW_CD69_POS_TREG_KLRG1_NEG_DN	186	-0.49909	-1.8807	0	0.004885
GSE5142_CTRL_VS_HTERT_TRANSDUCED_CD8_TCELL_EARLY_PASSAGE_CLONE_DN	174	-0.45689	-1.88023	0	0.004886
GSE7764_IL15_TREATED_VS_CTRL_NK_CELL_24H_DN	184	-0.56427	-1.87968	0	0.004905
GSE28737_WT_VS_BCL6_KO_FOLLICULAR_BCELL_UP	184	-0.54758	-1.8795	0	0.004909
GSE17721_POLYIC_VS_PAM3CSK4_1H_BMDC_DN	189	-0.51095	-1.87888	0	0.004928

GSE17721_CTRL_VS_GARDIQUIMOD_24H_BMDC_UP	183	-0.36731	-1.8787	0	0.004933
GSE2770_UNTREATED_VS_ACT_CD4_TCELL_6H_UP	176	-0.43094	-1.8786	0	0.004934
GSE19401_PAM2CSK4_VS_RETINOIC_ACID_AND_PAM2CSK4_STIM_FOLLICULAR_DC_UP	180	-0.47068	-1.87851	0	0.004937
GSE25087_FETAL_VS_ADULT_TREG_DN	162	-0.52553	-1.87844	0	0.00494
GSE32164_RESTING_DIFFERENTIATED_VS_CMYC_INHIBITED_MACROPHAGE_UP	191	-0.50883	-1.87681	0	0.005008
GSE21774_CD62L_POS_CD56_BRIGHT_VS_CD62L_NEG_CD56_DIM_NK_CELL_UP	181	-0.59994	-1.87716	0	0.005009
GSE40274_CTRL_VS_FOXP3_AND_EOS_TRANSDUCED_ACTIVATED_CD4_TCELL_UP	154	-0.55249	-1.87687	0	0.00501
GSE27786_CD8_TCELL_VS_NKTCELL_UP	176	-0.40273	-1.8764	0	0.005011
GSE5142_CTRL_VS_HTERT_TRANSDUCED_CD8_TCELL_EARLY_PASSAGE_CLONE_UP	187	-0.43453	-1.87693	0	0.005013
GSE20715_WT_VS_TLR4_KO_6H_OZONE_LUNG_DN	171	-0.41204	-1.87641	0	0.005014
GSE19941_UNSTIM_VS_LPS_AND_IL10_STIM_IL10_KO_NFKBP50_KO_MACROPHAGE_UP	159	-0.54446	-1.87642	0	0.005017
GSE6259_BCELL_VS_CD8_TCELL_DN	173	-0.45622	-1.87649	0	0.005017

GSE40666_NAIVE_VS_EFFECTOR_CD8_TCELL_DN	181	-0.47604	-1.87612	0	0.00502
GSE15330_HSC_VS_MEGAKARYOCYTE_ERYTHROID_PROGENITOR_DN	153	-0.49306	-1.87586	0	0.005025
GSE3982_CTRL_VS_IGE_STIM_MAST_CELL_DN	179	-0.34702	-1.8755	0	0.005027
GSE360_T_GONDII_VS_B_MALAYI_HIGH_DOSE_DC_UP	181	-0.52636	-1.87463	0	0.005067
GSE37605_NOD_VS_C57BL6_IRES_GFP_TREG_DN	149	-0.45786	-1.87467	0	0.005068
GSE7831_1H_VS_4H_CPG_STIM_PDC_UP	181	-0.3971	-1.87426	0	0.005082
GSE8835_CD4_VS_CD8_TCELL_CLL_PATIENT_DN	173	-0.54076	-1.87331	0	0.00512
GSE14769_UNSTIM_VS_120MIN_LPS_BMDM_UP	163	-0.30388	-1.87319	0	0.00512
GSE10239_KLRG1INT_VS_KLRG1HIGH_EFF_CD8_TCELL_DN	175	-0.5275	-1.87276	0	0.005136
GOLDRATH_NAIVE_VS_MEMORY_CD8_TCELL_DN	191	-0.55543	-1.87257	0	0.005143
GSE36078_WT_VS_IL1R_KO_LUNG_DC_UP	187	-0.52938	-1.87218	0	0.005154
GSE4590_LARGE_PRE_BCELL_VS_VPREB_POS_LARGE_PRE_BCELL_DN	174	-0.53526	-1.87112	0	0.005166

GSE22886_TH1_VS_TH2_12H_ACT_UP	180	-0.44499	-1.87121	0	0.005166
GSE7852_TREG_VS_TCONV_LN_DN	180	-0.54434	-1.87151	0	0.005168
GSE22935_UNSTIM_VS_24H_MBOVIS_BCG_STIM_MYD88_KO_MACROPHAGE_DN	178	-0.54392	-1.87197	0	0.005168
GSE11961_FOLLICULAR_BCELL_VS_MEMORY_BCELL_DAY40_UP	177	-0.43955	-1.87124	0	0.005169
GSE14308_NAIVE_CD4_TCELL_VS_NATURAL_TREG_UP	176	-0.43911	-1.87103	0	0.00517
GSE44649_WT_VS_MIR155_KO_NAIVE_CD8_TCELL_DN	184	-0.49245	-1.87127	0	0.005171
GSE24210_IL35_TREATED_VS_UNTREATED_TCONV_CD4_TCELL_UP	176	-0.43603	-1.87129	0	0.005174
GSE2826_WT_VS_XID_BCELL_DN	188	-0.48993	-1.87075	0	0.005177
GSE2585_THYMIC_MACROPHAGE_VS_MTEC_UP	173	-0.45416	-1.87082	0	0.005178
GSE5589_LPS_VS_LPS_AND_IL10_STIM_MACROPHAGE_45MIN_DN	178	-0.43162	-1.87173	0	0.005178
GSE26727_WT_VS_KLF2_KO_LPS_STIM_MACROPHAGE_UP	187	-0.46197	-1.87053	0	0.005181
GSE7460_CTRL_VS_TGFB_TREATED_ACT_CD8_TCELL_DN	180	-0.48641	-1.87001	0	0.005193

GSE29618_PRE_VS_DAY7_POST_TIV_FLU_VACCINE_MONOCYTE_DN	177	-0.3983	-1.86957	0	0.005202
GSE30971_2H_VS_4H_LPS_STIM_MACROPHAGE_WBP7_KO_UP	179	-0.49648	-1.86938	0	0.005205
GSE24142_ADULT_VS_FETAL_DN3_THYMOCYTE_DN	185	-0.48528	-1.86962	0	0.005208
GSE16450_CTRL_VS_IFNA_6H_STIM_IMMATURE_NEURON_CELL_LINE_UP	184	-0.50618	-1.86848	0	0.005219
GSE9650_NAIVE_VS_EXHAUSTED_CD8_TCELL_DN	181	-0.5048	-1.86862	0	0.00522
GSE23321_CENTRAL_VS_EFFECTOR_MEMORY_CD8_TCELL_UP	185	-0.42318	-1.86836	0	0.005223
GSE17721_PAM3CSK4_VS_CPG_0.5H_BMDC_DN	185	-0.44471	-1.86806	0	0.005236
GSE3982_NEUTROPHIL_VS_TH2_UP	189	-0.47859	-1.86774	0	0.005246
GSE29618_PRE_VS_DAY7_FLU_VACCINE_MDC_DN	174	-0.38872	-1.86762	0	0.005249
GSE22886_NAIVE_CD4_TCELL_VS_12H_ACT_TH2_UP	176	-0.45443	-1.86721	0	0.005249
GSE17186_BLOOD_VS_CORD_BLOOD_NAIVE_BCELL_DN	173	-0.43873	-1.86703	0	0.00525
GSE21063_WT_VS_NFATC1_KO_BCELL_UP	183	-0.55349	-1.86735	0	0.005253

GSE3982_CENT_MEMORY_CD4_TCELL_VS_NKCELL_UP	177	-0.46936	-1.86708	0	0.005253
GSE15930_STIM_VS_STIM_AND_IL12_48H_CD8_T_CELL_DN	193	-0.45917	-1.86648	0	0.005272
GSE5455_HEALTHY_VS_TUMOR_BEARING_MOUSE_SPLEEN_MONOCYTE_24H_INCUBATION_DN	177	-0.53225	-1.86639	0	0.005275
GSE2770_UNTREATED_VS_IL4_TREATED_ACT_CD4_TCELL_6H_UP	184	-0.41394	-1.86605	0	0.005287
GSE360_L_MAJOR_VS_B_MALAYI_LOW_DOSE_DC_DN	187	-0.39404	-1.86611	0	0.005288
GSE22935_WT_VS_MYD88_KO_MACROPHAGE_24H_MBOVIS_BCG_STIM_DN	182	-0.40117	-1.86561	0	0.005303
GSE32986_GMCSF_VS_GMCSF_AND_CURDLAN_HIGHDOSE_STIM_DC_DN	175	-0.49055	-1.86526	0	0.00531
GSE3720_LPS_VS_PMA_STIM_VD2_GAMMADELTA_TCELL_UP	129	-0.54095	-1.86506	0	0.005315
GSE3982_MAST_CELL_VS_TH2_UP	185	-0.45285	-1.86471	0	0.005322
GSE14415_INDUCED_TREG_VS_FOXP3_KO_INDUCED_TREG_DN	167	-0.52627	-1.86474	0	0.005325
GSE4142_GC_BCELL_VS_MEMORY_BCELL_UP	179	-0.44459	-1.86456	0	0.005327
GSE9509_LPS_VS_LPS_AND_IL10_STIM_IL10_KO_MACROPHAGE_30MIN_UP	167	-0.51091	-1.86436	0	0.005327

GSE32255_WT_UNSTIM_VS_JMJD2D_KNOCKDOWN_4H_LPS_STIM_DC_DN	166	-0.43316	-1.86417	0	0.005327
GSE16385_MONOCYTE_VS_12H_IL4_TREATED_MACROPHAGE_UP	172	-0.48944	-1.86426	0	0.005329
GSE5503_MLN_DC_VS_PLN_DC_ACTIVATED_ALLOGENIC_TCELL_DN	184	-0.49189	-1.86459	0	0.005329
GSE1448_CTRL_VS_ANTI_VALPHA2_DP_THYMOCYTE_DN	188	-0.45861	-1.86461	0	0.005329
GSE7218_UNSTIM_VS_ANTIGEN_STIM_THROUGH_IGG_BCELL_DN	160	-0.72764	-1.86355	0	0.005337
GSE25088_ROSIGLITAZONE_VS_IL4_AND_ROSIGLITAZONE_STIM_MACROPHAGE_DAY10_DN	181	-0.41501	-1.86361	0	0.00534
GSE34156_UNTREATED_VS_24H_NOD2_AND_TLR1_TLR2_LIGAND_TREATED_MONOCYTE_UP	169	-0.53234	-1.86334	0	0.005353
GSE5542_IFNG_VS_IFNA_TREATED_EPITHELIAL_CELLS_6H_DN	174	-0.51225	-1.86304	0	0.005359
GSE37605_C57BL6_VS_NOD_FOXP3_IRES_GFP_TCONV_UP	132	-0.52456	-1.86309	0	0.005361
GSE26290_WT_VS_PDK1_KO_ANTI_CD3_AND_IL2_STIM_CD8_TCELL_DN	180	-0.3256	-1.86254	0	0.005376
GSE3982_EOSINOPHIL_VS_BCELL_UP	185	-0.55407	-1.8622	0	0.005382
GSE22886_TCELL_VS_BCELL_NAIVE_DN	177	-0.63512	-1.86195	0	0.005397

GSE17721_CTRL_VS_LPS_8H_BMDC_DN	185	-0.45195	-1.86164	0	0.005411
GSE5589_IL6_KO_VS_IL10_KO_LPS_AND_IL10_STIM_MACROPHAGE_45MIN_DN	179	-0.4254	-1.8617	0	0.005412
GSE7852_TREG_VS_TCONV_THYMUS_UP	185	-0.54619	-1.86148	0	0.005413
GSE16385_ROSIGLITAZONE_IFNG_TNF_VS_IL4_STIM_MACROPHAGE_DN	182	-0.4012	-1.86092	0	0.005429
GSE26488_WT_VS_HDAC7_DELTAP_TG_OT2_THYMOCYTE_WITH_PEPTIDE_INJECTION_UP	190	-0.53717	-1.86071	0	0.005436
GSE7548_NAIVE_VS_DAY7_PCC_IMMUNIZATION_CD4_TCELL_UP	188	-0.34259	-1.86021	0	0.005447
GSE22025_UNTREATED_VS_PROGESTERONE_TREATED_CD4_TCELL_UP	188	-0.52835	-1.86042	0	0.005449
GSE7459_UNTREATED_VS_IL6_TREATED_ACT_CD4_TCELL_UP	131	-0.51668	-1.86024	0	0.00545
GSE12198_CTRL_VS_HIGH_IL2_STIM_NK_CELL_UP	183	-0.50867	-1.85996	0	0.005452
GSE3982_EOSINOPHIL_VS_DC_UP	186	-0.48909	-1.85962	0	0.005457
GSE1432_CTRL_VS_IFNG_1H_MICROGLIA_DN	182	-0.45262	-1.85969	0	0.005459
GSE38304_MYC_NEG_VS_POS_GC_BCELL_UP	189	-0.48665	-1.85949	0	0.005463

GSE360_DC_VS_MAC_T_GONDII_UP	182	-0.5703	-1.85899	0	0.005468
GSE19941_UNSTIM_VS_LPS_STIM_IL10_KO_MACROPHAGE_UP	181	-0.5312	-1.85921	0	0.005469
GSE23505_UNTREATED_VS_4DAY_IL6_IL1_TGFB_TREATED_CD4_TCELL_DN	183	-0.32003	-1.85903	0	0.005469
GSE28237_FOLLICULAR_VS_LATE_GC_BCELL_UP	180	-0.49856	-1.85889	0	0.005471
GSE17974_0H_VS_24H_IN_VITRO_ACT_CD4_TCELL_UP	166	-0.56328	-1.85921	0	0.005472
GSE21670_TGFB_VS_IL6_TREATED_STAT3_KO_CD4_TCELL_UP	177	-0.43643	-1.85909	0	0.005474
GSE14308_TH2_VS_NATURAL_TREG_DN	180	-0.40354	-1.85914	0	0.005476
GSE22229_UNTREATED_VS_IMMUNOSUPP_THERAPY_RENAL_TRANSPLANT_PATIENT_PBMC_UP	179	-0.51711	-1.85828	0	0.005493
GSE1460_INTRATHYMIC_T_PROGENITOR_VS_THYMIC_STROMAL_CELL_UP	178	-0.39974	-1.85804	0	0.005498
GSE30083_SP1_VS_SP2_THYMOCYTE_UP	182	-0.44606	-1.85746	0	0.005536
GSE15930_NAIVE_VS_24H_IN_VITRO_STIM_INFAB_CD8_TCELL_UP	188	-0.45614	-1.85731	0	0.005538
GSE3920_IFNA_VS_IFNG_TREATED_ENDOTHELIAL_CELL_DN	157	-0.49195	-1.85736	0	0.005538

GSE21063_3H_VS_16H_ANTI_IGM_STIM_BCELL_UP	172	-0.44766	-1.85729	0	0.005539
GSE20198_UNTREATED_VS_IL12_TREATED_ACT_CD4_TCELL_DN	183	-0.41968	-1.85681	0	0.005556
GSE6259_DEC205_POS_DC_VS_CD4_TCELL_UP	179	-0.42133	-1.85636	0	0.00557
GSE3982_NKCELL_VS_TH2_UP	183	-0.55005	-1.85641	0	0.005571
GSE37301_PRO_BCELL_VS_RAG2_KO_NK_CELL_DN	173	-0.51992	-1.85568	0	0.005599
GSE9037_CTRL_VS_LPS_4H_STIM_BMDM_DN	174	-0.36874	-1.85569	0	0.005602
GSE37416_CTRL_VS_48H_F_TULARENSIS_LVS_NEUTROPHIL_UP	165	-0.52847	-1.85579	0	0.005606
GSE30083_SP2_VS_SP3_THYMOCYTE_DN	187	-0.56567	-1.85526	0	0.005609
GSE17721_CTRL_VS_LPS_6H_BMDC_DN	185	-0.438	-1.8551	0	0.005611
GSE21774_CD62L_POS_CD56_BRIGHT_VS_CD62L_NEG_CD56_DIM_NK_CELL_DN	183	-0.49714	-1.85532	0	0.005613
GSE11961_GERMINAL_CENTER_BCELL_DAY7_VS_PLASMA_CELL_DAY7_DN	179	-0.42941	-1.85493	0	0.005617
GSE17721_0.5H_VS_24H_CPG_BMDC_DN	191	-0.49509	-1.8547	0	0.005624

GSE22886_NAIVE_VS_IGM_MEMORY_BCELL_UP	176	-0.50262	-1.8546	0	0.005624
GSE25123_WT_VS_PPARG_KO_MACROPHAGE_IL4_STIM_UP	183	-0.44087	-1.85452	0	0.005626
GSE22886_CD8_VS_CD4_NAIVE_TCELL_DN	174	-0.53428	-1.8544	0	0.005628
GSE37301_MULTIPOTENT_PROGENITOR_VS_LYMPHOID_PRIMED_MPP_DN	173	-0.5246	-1.85429	0	0.005628
GSE24142_EARLY_THYMIC_PROGENITOR_VS_DN2_THYMOCYTE_DN	187	-0.57302	-1.85454	0	0.005628
GSE12845_NAIVE_VS_DARKZONE_GC_TONSIL_BCELL_UP	178	-0.44758	-1.85388	0	0.005651
GSE14699_NAIVE_VS_ACT_CD8_TCELL_UP	182	-0.46516	-1.8536	0	0.005665
GSE24574_BCL6_HIGH_TFH_VS_TFH_CD4_TCELL_UP	182	-0.53839	-1.853	0	0.00569
GSE10211_UV_INACT_SENDAI_VS_LIVE_SENDAI_VIRUS_TRACHEAL_EPITHELIAL_CELLS_DN	147	-0.46373	-1.85292	0	0.005691
GSE13522_WT_VS_IFNAR_KO_SKING_T_CRUZI_Y_STRAIN_INF_UP	182	-0.5853	-1.85279	0	0.005691
GSE18893_TCONV_VS_TREG_24H_CULTURE_UP	186	-0.46293	-1.85264	0	0.005696
GSE7460_TCONV_VS_TREG_LN_UP	180	-0.53466	-1.85261	0	0.005698

GSE22229_RENAL_TRANSPLANT_IMMUNOSUPP_THERAPY_VS_HEALTHY_PBMC_DN	177	-0.47504	-1.85245	0	0.005701
GSE5589_IL6_KO_VS_IL10_KO_LPS_STIM_MACROPHAGE_180MIN_DN	176	-0.46665	-1.85207	0	0.005711
GSE26928_NAIVE_VS_EFF_MEMORY_CD4_TCELL_UP	168	-0.42834	-1.85183	0	0.005725
GSE39916_B_CELL_SPLEEN_VS_PLASMA_CELL_BONE_MARROW_UP	184	-0.50862	-1.85174	0	0.005727
GSE27291_0H_VS_7D_STIM_GAMMADELTA_TCELL_UP	162	-0.42127	-1.85142	0	0.005737
GSE13306_RA_VS_UNTREATED_TREG_UP	173	-0.42749	-1.85142	0	0.005741
GSE21774_CD62L_POS_CD56_DIM_VS_CD62L_NEG_CD56_DIM_NK_CELL_DN	185	-0.57341	-1.85124	0	0.005744
GSE21546_UNSTIM_VS_ANTI_CD3_STIM_ELK1_KO_DP_THYMOCYTES_UP	164	-0.57952	-1.85113	0	0.005746
GSE13306_TREG_VS_TCONV_DN	175	-0.39389	-1.85091	0	0.005748
GSE9601_UNTREATED_VS_PI3K_INHIBITOR_TREATED_HCMV_INF_MONOCYTE_DN	142	-0.43652	-1.85043	0	0.005761
GSE19923_E2A_KO_VS_HEB_AND_E2A_KO_DP_THYMOCYTE_UP	186	-0.49025	-1.84959	0	0.005791
GSE17974_0H_VS_6H_IN_VITRO_ACT_CD4_TCELL_UP	161	-0.50389	-1.84843	0	0.005813

GSE37605_NOD_VS_C57BL6_IRES_GFP_TREG_UP	146	-0.51319	-1.84845	0	0.005816
GSE30083_SP1_VS_SP2_THYMOCYTE_DN	187	-0.55542	-1.84848	0	0.005819
GSE16266_CTRL_VS_LPS_STIM_MEF_DN	183	-0.55168	-1.84872	0	0.005819
GSE22886_NAIVE_VS_IGG_IGA_MEMORY_BCELL_UP	178	-0.52155	-1.84859	0	0.00582
GSE11961_MARGINAL_ZONE_BCELL_VS_GERMINAL_CENTER_BCELL_DAY40_UP	184	-0.39627	-1.84889	0	0.005821
GSE37301_CD4_TCELL_VS_RAG2_KO_NK_CELL_UP	144	-0.56578	-1.84865	0	0.005822
GSE3720_UNSTIM_VS_LPS_STIM_VD1_GAMMADELTA_TCELL_DN	140	-0.54513	-1.8489	0	0.005822
GSE6269_E_COLI_VS_STREP_PNEUMO_INF_PBMC_UP	146	-0.48344	-1.84872	0	0.005823
GSE46606_IRF4_KO_VS_WT_UNSTIM_BCELL_DN	183	-0.57504	-1.84762	0	0.005844
GSE17721_CTRL_VS_LPS_4H_BMDC_DN	183	-0.47362	-1.84781	0	0.005846
GSE3720_VD1_VS_VD2_GAMMADELTA_TCELL_WITH_LPS_STIM_UP	181	-0.38227	-1.84723	0	0.005859
GSE17721_CTRL_VS_POLYIC_4H_BMDC_DN	183	-0.45252	-1.84703	0	0.005869

GSE13229_IMM_VS_MATURE_NKCELL_UP	182	-0.47214	-1.84694	0	0.005871
GSE37301_PRO_BCELL_VS_RAG2_KO_NK_CELL_UP	166	-0.38939	-1.84636	0	0.005872
GSE27786_NKCELL_VS_ERYTHROBLAST_UP	173	-0.28299	-1.8464	0	0.005876
GSE17721_0.5H_VS_4H_LPS_BMDC_DN	184	-0.46354	-1.84656	0	0.005877
GSE20366_EX_VIVO_VS_DEC205_CONVERSION_DN	181	-0.43104	-1.84616	0	0.005879
GSE17974_CTRL_VS_ACT_IL4_AND_ANTI_IL12_6H_CD4_TCELL_UP	162	-0.51876	-1.84657	0	0.00588
GSE24574_BCL6_HIGH_TFH_VS_NAIVE_CD4_TCELL_UP	187	-0.48003	-1.84598	0	0.00588
GSE17721_POLYIC_VS_GARDIQUIMOD_2H_BMDC_UP	180	-0.36996	-1.84659	0	0.005883
GSE5589_LPS_VS_LPS_AND_IL10_STIM_IL6_KO_MACROPHAGE_45MIN_DN	188	-0.47583	-1.84574	0	0.005888
GSE7852_TREG_VS_TCONV_THYMUS_DN	179	-0.48735	-1.84552	0	0.005896
GSE1448_CTRL_VS_ANTI_VBETA5_DP_THYMOCYTE_DN	189	-0.445	-1.84527	0	0.005907
GSE5679_PPARG_LIGAND_ROSIGLITAZONE_VS_ROSIGLITAZONE_AND_RARA_AGONIST_AM580_TREATED_DC_DN	175	-0.49779	-1.84509	0	0.005911

GSE5589_LPS_VS_LPS_AND_IL6_STIM_IL6_KO_MACROPHAGE_45MIN_UP	188	-0.46576	-1.84514	0	0.005914
GSE29949_CD8_POS_DC_SPLEEN_VS_MONOCYTE_BONE_MARROW_DN	190	-0.41825	-1.84472	0	0.005922
GSE21670_STAT3_KO_VS_WT_CD4_TCELL_TGFB_IL6_TREATED_DN	182	-0.62848	-1.84475	0	0.005924
GSE17974_CTRL_VS_ACT_IL4_AND_ANTI_IL12_12H_CD4_TCELL_UP	162	-0.51614	-1.84416	0	0.005933
GSE360_T_GONDII_VS_M_TUBERCULOSIS_DC_DN	190	-0.51405	-1.84366	0	0.005959
GSE26928_EFF_MEM_VS_CENTR_MEM_CD4_TCELL_DN	146	-0.50661	-1.84278	0	0.005959
GSE16266_CTRL_VS_HEATSHOCK_AND_LPS_STIM_MEF_DN	186	-0.35262	-1.84331	0	0.005962
GSE9006_HEALTHY_VS_TYPE_1_DIABETES_PBMC_1MONTH_POST_DX_DN	175	-0.45912	-1.84338	0	0.005962
GSE11961_MEMORY_BCELL_DAY7_VS_PLASMA_CELL_DAY7_DN	186	-0.45897	-1.8431	0	0.005963
GSE3982_MAST_CELL_VS_EFF_MEMORY_CD4_TCELL_DN	180	-0.54034	-1.84295	0	0.005964
GSE17301_CTRL_VS_48H_ACD3_ACD28_STIM_CD8_TCELL_UP	183	-0.43148	-1.84344	0	0.005964
GSE29164_UNTREATED_VS_CD8_TCELL_AND_IL12_TREATED_MELANOMA_DAY7_DN	183	-0.47003	-1.84281	0	0.005964

GSE11961_MARGINAL_ZONE_BCELL_VS_PLASMA_CELL_DAY7_UP	179	-0.42987	-1.84314	0	0.005965
GSE11961_GERMINAL_CENTER_BCELL_DAY7_VS_GERMINAL_CENTER_BCELL_DAY40_DN	184	-0.43761	-1.84298	0	0.005966
GSE39820_CTRL_VS_TGFBETA3_IL6_CD4_TCELL_DN	182	-0.37224	-1.84319	0	0.005966
GSE40274_FOXP3_VS_FOXP3_AND_LEF1_TRANSDUCED_ACTIVATED_CD4_TCELL_DN	186	-0.47646	-1.84244	0	0.005968
GSE22611_MUTANT_NOD2_VS_CTRL_TRANSDUCED_HEK293T_CELL_UP	180	-0.4565	-1.84257	0	0.005969
GSE19374_UNINF_VS_LISTERIA_INFECTED_MACROPHAGE_UP	183	-0.52451	-1.84218	0	0.005977
GSE39820_CTRL_VS_IL1B_IL6_CD4_TCELL_UP	186	-0.45553	-1.84106	0	0.006026
GSE15624_CTRL_VS_6H_HALOFUGINONE_TREATED_CD4_TCELL_UP	129	-0.51688	-1.84086	0	0.006026
GSE11961_GERMINAL_CENTER_BCELL_DAY7_VS_MEMORY_BCELL_DAY40_DN	192	-0.5377	-1.84075	0	0.006031
GSE39820_TGFBETA3_IL6_VS_TGFBETA3_IL6_IL23A_TREATED_CD4_TCELL_UP	180	-0.41953	-1.84058	0	0.006037
GSE40277_GATA1_AND_SATB1_TRANSDUCED_VS_CTRL_CD4_TCELL_DN	188	-0.46334	-1.84011	0	0.006051
GSE21927_GMCSF_IL6_VS_GMCSF_GCSF_TREATED_BONE_MARROW_UP	153	-0.49435	-1.83984	0	0.006065

GSE2706_UNSTIM_VS_8H_LPS_DC_UP	176	-0.35653	-1.83917	0	0.006066
GSE37301_HEMATOPOIETIC_STEM_CELL_VS_MULTIPOTENT_PROGENITOR_DN	153	-0.37147	-1.83922	0	0.006067
GSE17721_CPG_VS_GARDIQUIMOD_4H_BMDC_UP	181	-0.43961	-1.83968	0	0.006069
GSE41176_UNSTIM_VS_ANTI_IGM_STIM_TAK1_KO_BCELL_6H_UP	175	-0.42681	-1.8396	0	0.006069
GSE19888_ADENOSINE_A3R_INH_VS_ACT_WITH_INHIBITOR_PRETREATMENT_IN_MAST_CELL_UP	183	-0.61263	-1.83905	0	0.00607
GSE40666_UNTREATED_VS_IFNA_STIM_STAT1_KO_CD8_TCELL_90MIN_UP	157	-0.52661	-1.83973	0	0.006071
GSE5589_WT_VS_IL10_KO_LPS_AND_IL10_STIM_MACROPHAGE_45MIN_UP	178	-0.42096	-1.83852	0	0.00608
GSE26030_UNSTIM_VS_RESTIM_TH17_DAY15_POST_POLARIZATION_UP	167	-0.42808	-1.8382	0	0.00609
GSE11961_FOLLICULAR_BCELL_VS_GERMINAL_CENTER_BCELL_DAY7_UP	177	-0.37384	-1.83804	0	0.006096
GSE23114_PERITONEAL_CAVITY_B1A_BCELL_VS_SPLEEN_BCELL_IN_SLE2C1_MOUSE_DN	172	-0.46566	-1.83775	0	0.006099
GSE36392_TYPE_2_MYELOID_VS_NEUTROPHIL_IL25_TREATED_LUNG_UP	186	-0.513	-1.83785	0	0.0061
GSE8835_HEALTHY_VS_CLL_CD8_TCELL_DN	176	-0.51773	-1.83777	0	0.006102

GSE37416_12H_VS_48H_F_TULARENSIS_LVS_NEUTROPHIL_DN	155	-0.41633	-1.83705	0	0.00614
GSE32034_UNTREATED_VS_ROSIGLIZATONE_TREATED_LY6C_LOW_MONOCYTE_UP	162	-0.53166	-1.83683	0	0.006154
GSE37534_PIOGLITAZONE_VS_ROSIGLITAZONE_TREATED_CD4_TCELL_PPARG1_FOXP3_TRANSDUCED_DN	154	-0.45652	-1.83671	0	0.006163
GSE5589_WT_VS_IL10_KO_LPS_STIM_MACROPHAGE_45MIN_DN	184	-0.34506	-1.83604	0	0.006203
GSE16450_CTRL_VS_IFNA_6H_STIM_MATURE_NEURON_CELL_LINE_UP	187	-0.53295	-1.83554	0	0.006211
GSE15330_MEGAKARYOCYTE_ERYTHROID_PROGENITOR_VS_PRO_BCELL_UP	182	-0.3566	-1.83534	0	0.006217
GSE17721_CPG_VS_GARDIQUIMOD_8H_BMDC_UP	186	-0.39198	-1.83516	0	0.006225
GSE42021_CD24LO_TREG_VS_CD24LO_TCONV_THYMUS_UP	189	-0.35927	-1.83403	0	0.006247
GSE21670_UNTREATED_VS_TGFB_TREATED_CD4_TCELL_DN	178	-0.49381	-1.83471	0	0.006247
GSE1460_CD4_THYMOCYTE_VS_NAIVE_CD4_TCELL_CORD_BLOOD_DN	175	-0.43404	-1.83415	0	0.006247
GSE35685_CD34POS_CD38NEG_VS_CD34POS_CD10POS_BONE_MARROW_UP	187	-0.40172	-1.83404	0	0.00625
GSE19923_WT_VS_E2A_KO_DP_THYMOCYTE_DN	180	-0.41982	-1.83453	0	0.00625

GSE5589_LPS_VS_LPS_AND_IL10_STIM_IL10_KO_MACROPHAGE_180MIN_UP	184	-0.52786	-1.83416	0	0.006253
GSE34156_NOD2_LIGAND_VS_TLR1_TLR2_LIGAND_24H_TREATED_MONOCYTE_UP	154	-0.48919	-1.83416	0	0.006256
GSE5542_UNTREATED_VS_IFNA_AND_IFNG_TREATED_EPITHELIAL_CELLS_6H_UP	172	-0.45838	-1.8334	0	0.006277
GSE11961_MARGINAL_ZONE_BCELL_VS_MEMORY_BCELL_DAY40_UP	185	-0.49077	-1.83308	0	0.006293
GSE15324_NAIVE_VS_ACTIVATED_ELF4_KO_CD8_TCELL_UP	178	-0.44362	-1.83278	0	0.0063
GSE12003_MIR223_KO_VS_WT_BM_PROGENITOR_8D_CULTURE_UP	116	-0.50154	-1.83255	0	0.006312
GSE15750_DAY6_VS_DAY10_EFF_CD8_TCELL_DN	178	-0.41913	-1.83242	0	0.006316
GSE24972_MARGINAL_ZONE_BCELL_VS_FOLLICULAR_BCELL_DN	180	-0.53644	-1.83236	0	0.006319
GSE3982_BASOPHIL_VS_CENT_MEMORY_CD4_TCELL_DN	175	-0.41886	-1.83186	0	0.00634
GSE17186_NAIVE_VS_CD21HIGH_TRANSITIONAL_BCELL_UP	177	-0.46403	-1.83135	0	0.006369
GSE36392_TYPE_2_MYELOID_VS_NEUTROPHIL_IL25_TREATED_LUNG_DN	184	-0.35779	-1.8305	0	0.006406
GSE37301_MULTIPOTENT_PROGENITOR_VS_LYMPHOID_PRIMED_MPP_UP	148	-0.38155	-1.8299	0	0.006439

GSE21670_STAT3_KO_VS_WT_CD4_TCELL_IL6_TREATED_DN	185	-0.5134	-1.82968	0	0.006447
GSE18893_CTRL_VS_TNF_TREATED_TREG_2H_DN	184	-0.4553	-1.82949	0	0.006455
GSE35543_IN_VIVO_NTREG_VS_IN_VITRO_ITREG_DN	151	-0.50398	-1.82925	0	0.006456
GSE5589_WT_VS_IL10_KO_LPS_AND_IL10_STIM_MACROPHAGE_180MIN_UP	147	-0.41422	-1.82933	0	0.00646
GSE22033_UNTREATED_VS_MRL24_TREATED_MEF_DN	188	-0.39991	-1.82878	0	0.006469
GSE18791_CTRL_VS_NEWCASTLE_VIRUS_DC_14H_DN	162	-0.57747	-1.82825	0	0.006502
GSE17186_MEMORY_VS_NAIVE_BCELL_UP	173	-0.41645	-1.82795	0	0.00651
GSE11961_MARGINAL_ZONE_BCELL_VS_GERMINAL_CENTER_BCELL_DAY7_UP	175	-0.43905	-1.82805	0	0.006515
GSE17974_CTRL_VS_ACT_IL4_AND_ANTI_IL12_72H_CD4_TCELL_UP	168	-0.4606	-1.82744	0	0.006528
GSE13306_RA_VS_UNTREATED_TCONV_DN	179	-0.50944	-1.82744	0	0.006531
GSE17974_0H_VS_12H_IN_VITRO_ACT_CD4_TCELL_UP	164	-0.49619	-1.82687	0	0.006559
GSE2585_THYMIC_MACROPHAGE_VS_MTEC_DN	172	-0.4745	-1.82563	0	0.006566

GSE10239_NAIVE_VS_MEMORY_CD8_TCELL_DN	179	-0.49694	-1.82645	0	0.00657
GSE41867_NAIVE_VS_DAY30_LCMV_CLONE13_EXHAUSTED_CD8_TCELL_UP	177	-0.56492	-1.82615	0	0.006572
GSE32034_LY6C_HIGH_VS_LOW_MONOCYTE_UP	189	-0.53507	-1.82649	0	0.006572
GSE34179_THPOK_KO_VS_WT_VA14I_NKTCELL_UP	182	-0.45985	-1.82585	0	0.006574
GSE6259_33D1_POS_VS_DEC205_POS_SPLENIC_DC_DN	179	-0.4516	-1.82616	0	0.006575
GSE360_L_DONOVANI_VS_T_GONDII_MAC_UP	182	-0.46803	-1.82616	0	0.006578
GSE17301_IFNA2_VS_IFNA2_AND_ACD3_ACD28_STIM_CD8_TCELL_DN	183	-0.48897	-1.82525	0	0.006598
GSE3982_EOSINOPHIL_VS_TH1_UP	186	-0.46475	-1.82501	0	0.006608
GSE369_IFNG_KO_VS_WT_LIVER_UP	173	-0.39928	-1.82489	0	0.006616
GSE24671_CTRL_VS_BAKIMULC_INFECTED_MOUSE_SPLENOCYTES_DN	161	-0.56366	-1.82449	0	0.006633
GSE15659_NAIVE_CD4_TCELL_VS_RESTING_TREG_UP	157	-0.44928	-1.8239	0	0.006649
GSE13493_CD4INTCD8POS_VS_CD8POS_THYMOCYTE_UP	181	-0.41714	-1.82397	0	0.00665

GSE15659_NONSUPPRESSIVE_TCELL_VS_ACTIVATED_TREG_DN	181	-0.39083	-1.82351	0	0.006652
GSE1566_WT_VS_EZH2_KO_LN_TCELL_UP	161	-0.53194	-1.82289	0	0.006684
GSE28130_ACTIVATED_VS_INDUCEED_TREG_UP	180	-0.50948	-1.82285	0	0.006684
GSE37534_GW1929_VS_PIOGLITAZONE_TREATED_CD4_TCELL_PPARG1_FOXP3_TRANSDUCED_UP	183	-0.4973	-1.82275	0	0.006686
GSE8685_IL2_STARVED_VS_IL21_ACT_IL2_STARVED_CD4_TCELL_DN	183	-0.37891	-1.82289	0	0.006687
GSE3982_CTRL_VS_LPS_1H_NEUTROPHIL_UP	175	-0.3841	-1.82236	0	0.006698
GSE13411_NAIVE_VS_IGM_MEMORY_BCELL_UP	171	-0.49623	-1.82206	0	0.006715
GSE14769_UNSTIM_VS_360MIN_LPS_BMDM_DN	187	-0.43716	-1.82122	0	0.006763
GSE13522_WT_VS_IFNG_KO_SKING_T_CRUZI_Y_STRAIN_INF_DN	124	-0.496	-1.82114	0	0.006764
GSE41176_WT_VS_TAK1_KO_ANTI_IGM_STIM_BCELL_1H_DN	178	-0.57704	-1.81991	0	0.006817
GSE4984_UNTREATED_VS_LPS_TREATED_DC_UP	144	-0.45515	-1.81956	0	0.006826
GSE11884_WT_VS_FURIN_KO_NAIVE_CD4_TCELL_UP	122	-0.53356	-1.81876	0	0.006876

GSE21670_TGFB_VS_TGFB_AND_IL6_TREATED_STAT3_KO_CD4_TCELL_UP	179	-0.37401	-1.81855	0	0.00688
GSE3982_EOSINOPHIL_VS_NKCELL_DN	175	-0.39068	-1.81835	0	0.006893
GSE22025_UNTREATED_VS_PROGESTERONE_TREATED_CD4_TCELL_DN	177	-0.41695	-1.81827	0	0.006894
GSE3203_UNTREATED_VS_IFNB_TREATED_LN_BCELL_UP	179	-0.43321	-1.81723	0	0.006926
GSE12366_GC_BCELL_VS_PLASMA_CELL_DN	167	-0.43663	-1.81726	0	0.006928
GSE32901_NAIVE_VS_TH1_CD4_TCELL_UP	118	-0.5536	-1.81743	0	0.006928
GSE2770_UNTREATED_VS_IL4_TREATED_ACT_CD4_TCELL_6H_DN	189	-0.35568	-1.8173	0	0.006934
GSE46606_IRF4MID_VS_WT_CD40L_IL2_IL5_DAY1_STIMULATED_BCELL_DN	180	-0.47674	-1.81682	0	0.00694
GSE13493_CD4INTCD8POS_VS_CD8POS_THYMOCYTE_DN	178	-0.46271	-1.81635	0	0.006944
GSE360_CTRL_VS_T_GONDII_DC_DN	186	-0.49895	-1.8167	0	0.006944
GSE10240_IL17_VS_IL17_AND_IL22_STIM_PRIMARY_BRONCHIAL_EPITHELIAL_CELLS_UP	173	-0.37902	-1.81623	0	0.006947
GSE3982_DC_VS_NEUTROPHIL_DN	181	-0.46137	-1.81687	0	0.006947

GSE8685_IL15_ACT_IL2_STARVED_VS_IL21_ACT_IL2_STARVED_CD4_TCELL_DN	149	-0.4085	-1.81617	0	0.00695
GSE1460_DP_THYMOCYTE_VS_THYMIC_STROMAL_CELL_UP	170	-0.41803	-1.81599	0	0.00696
GSE17721_CTRL_VS_LPS_24H_BMDC_UP	186	-0.40112	-1.81561	0	0.00697
GSE22886_NAIVE_VS_MEMORY_TCELL_UP	179	-0.4686	-1.81469	0	0.007028
GSE40441_NRP1_POS_INDUCED_TREG_VS_NRP1_NEG_NATURAL_TREG_DN	187	-0.45181	-1.81362	0	0.007082
GSE13738_RESTING_VS_TCR_ACTIVATED_CD4_TCELL_UP	163	-0.49095	-1.81334	0	0.007086
GSE2826_XID_VS_BTK_KO_BCELL_DN	191	-0.47681	-1.81337	0	0.00709
GSE16385_UNTREATED_VS_12H_IFNG_TNF_TREATED_MACROPHAGE_DN	167	-0.42952	-1.81339	0	0.007092
GSE28726_NAIVE_CD4_TCELL_VS_NAIVE_VA24NEG_NKTCELL_DN	180	-0.52237	-1.81298	0	0.007106
GSE22601_DOUBLE_NEGATIVE_VS_IMMATURE_CD4_SP_THYMOCYTE_DN	162	-0.47369	-1.81274	0	0.007112
GSE26495_NAIVE_VS_PD1LOW_CD8_TCELL_UP	158	-0.49138	-1.81279	0	0.007113
GSE16385_ROSIGLITAZONE_IL4_VS_IL4_ALONE_STIM_MACROPHAGE_12H_DN	192	-0.42254	-1.81247	0	0.007122

GSE21063_CTRL_VS_ANTI_IGM_STIM_BCELL_NFATC1_KO_3H_UP	187	-0.45608	-1.81248	0	0.007123
GSE34156_UNTREATED_VS_24H_TLR1_TLR2_LIGAND_TREATED_MONOCYTE_UP	162	-0.52839	-1.81259	0	0.007123
GSE14415_INDUCED_TREG_VS_FOXP3_KO_INDUCED_TREG_IL2_CULTURE_DN	124	-0.53114	-1.81249	0	0.007125
GSE17721_CPG_VS_GARDIQUIMOD_1H_BMDC_UP	188	-0.4472	-1.81235	0	0.007128
GSE2770_IL12_AND_TGFB_VS_IL4_TREATED_ACT_CD4_TCELL_6H_DN	178	-0.55871	-1.811	0	0.007183
GSE1460_DP_THYMOCYTE_VS_NAIVE_CD4_TCELL_CORD_BLOOD_DN	182	-0.49019	-1.81088	0	0.007191
GSE36476_CTRL_VS_TSST_ACT_16H_MEMORY_CD4_TCELL_OLD_UP	180	-0.50626	-1.81077	0	0.007194
GSE360_CTRL_VS_T_GONDII_MAC_DN	182	-0.41957	-1.81019	0	0.007233
GSE21927_SPLENIC_C26GM_TUMOROUS_VS_BONE_MARROW_MONOCYTES_UP	139	-0.54201	-1.81019	0	0.007236
GSE29949_DC_BRAIN_VS_MONOCYTE_BONE_MARROW_DN	185	-0.49628	-1.80971	0	0.007261
GSE9037_CTRL_VS_LPS_1H_STIM_BMDM_DN	178	-0.45761	-1.80945	0	0.007274
GSE3565_DUSP1_VS_WT_SPLENOCYTES_POST_LPS_INJECTION_DN	150	-0.5151	-1.80936	0	0.007277

GSE14308_TH2_VS_INDUCED_TREG_UP	179	-0.35036	-1.80892	0	0.007306
GSE46606_IRF4MID_VS_WT_CD40L_IL2_IL5_DAY1_STIMULATED_BCELL_UP	175	-0.38939	-1.80842	0	0.007319
GSE15659_CD45RA_NEG_CD4_TCELL_VS_ACTIVATED_TREG_DN	178	-0.39478	-1.80845	0	0.007321
GSE5589_WT_VS_IL10_KO_LPS_AND_IL6_STIM_MACROPHAGE_45MIN_DN	184	-0.4677	-1.80861	0	0.007322
GSE22935_UNSTIM_VS_48H_MBOVIS_BCG_STIM_MYD88_KO_MACROPHAGE_UP	175	-0.4967	-1.80846	0	0.007324
GSE25087_TREG_VS_TCONV_FETUS_DN	164	-0.37444	-1.80794	0	0.007356
GSE10239_MEMORY_VS_KLRG1INT_EFF_CD8_TCELL_UP	178	-0.4746	-1.80748	0	0.007361
GSE36476_YOUNG_VS_OLD_DONOR_MEMORY_CD4_TCELL_16H_TSST_ACT_DN	173	-0.46258	-1.80751	0	0.007366
GSE43955_1H_VS_10H_ACT_CD4_TCELL_WITH_TGFB_IL6_DN	191	-0.46736	-1.80757	0	0.007367
GSE19923_WT_VS_HEB_KO_DP_THYMOCYTE_UP	190	-0.49137	-1.80766	0	0.007369
GSE10239_MEMORY_VS_KLRG1HIGH_EFF_CD8_TCELL_UP	180	-0.50574	-1.80762	0	0.00737
GSE3982_DC_VS_NKCELL_DN	179	-0.51075	-1.80712	0	0.007378

GSE34515_CD16_NEG_VS_POS_MONOCYTE_UP	179	-0.46042	-1.80699	0	0.007383
GSE25088_WT_VS_STAT6_KO_MACROPHAGE_ROSIGLITAZONE_AND_IL4_STIM_DN	176	-0.50581	-1.80686	0	0.007384
GSE1925_CTRL_VS_24H_IFNG_STIM_IFNG_PRIMED_MACROPHAGE_UP	182	-0.39982	-1.80688	0	0.007385
GSE28737_BCL6_HET_VS_BCL6_KO_MARGINAL_ZONE_BCELL_UP	178	-0.57904	-1.80607	0	0.007427
GSE2770_IL12_VS_TGFB_AND_IL12_TREATED_ACT_CD4_TCELL_48H_DN	180	-0.42339	-1.80472	0	0.007505
GSE8921_UNSTIM_VS_TLR1_2_STIM_MONOCYTE_6H_UP	181	-0.43596	-1.8037	0	0.007543
GSE17974_IL4_AND_ANTI_IL12_VS_UNTREATED_6H_ACT_CD4_TCELL_DN	169	-0.5039	-1.80375	0	0.007546
GSE20727_DNFB_ALLERGEN_VS_ROS_INH_AND_DNFB_ALLERGEN_TREATED_DC_UP	172	-0.49332	-1.80391	0	0.007548
GSE27786_BCELL_VS_NKTCELL_UP	184	-0.35751	-1.80351	0	0.007548
GSE43863_TFH_VS_LY6C_LOW_CXCR5NEG_EFFECTOR_CD4_TCELL_DN	189	-0.56068	-1.80354	0	0.007552
GSE19941_IL10_KO_VS_IL10_KO_AND_NFKBP50_KO_LPS_AND_IL10_STIM_MACROPHAGE_UP	186	-0.4375	-1.80309	0	0.007568
GSE26928_NAIVE_VS_CENT_MEMORY_CD4_TCELL_UP	166	-0.41489	-1.80271	0	0.007587

GSE2826_WT_VS_BTK_KO_BCELL_DN	187	-0.49439	-1.8022	0	0.007596
GSE7460_FOXP3_MUT_VS_HET_ACT_TCONV_UP	184	-0.51641	-1.80222	0	0.007599
GSE1460_INTRATHYMIC_T_PROGENITOR_VS_DP_THYMOCYTE_UP	175	-0.43763	-1.80224	0	0.007604
GSE11057_NAIVE_VS_CENT_MEMORY_CD4_TCELL_UP	153	-0.49883	-1.80166	0	0.00761
GSE2585_THYMIC_DC_VS_MTEC_UP	178	-0.44364	-1.80175	0	0.007611
GSE26669_CD4_VS_CD8_TCELL_IN_MLR_UP	190	-0.3662	-1.80152	0	0.007616
GSE7460_CD8_TCELL_VS_CD4_TCELL_ACT_DN	179	-0.38155	-1.80148	0	0.007617
GSE33513_TCF7_KO_VS_HET_EARLY_THYMIC_PROGENITOR_UP	181	-0.52196	-1.80155	0	0.007618
GSE22611_UNSTIM_VS_2H_MDP_STIM_MUTANT_NOD2_TRANSDUCED_HEK293T_CELL_UP	176	-0.36341	-1.80065	0	0.007638
GSE18203_CTRL_VS_INTRATUMORAL_CPG_INJ_MC38_TUMOR_UP	179	-0.43913	-1.80067	0	0.007642
GSE11961_PLASMA_CELL_DAY7_VS_GERMINAL_CENTER_BCELL_DAY40_UP	180	-0.44368	-1.80037	0	0.007644
GSE25123_CTRL_VS_IL4_AND_ROSIGLITAZONE_STIM_PPARG_KO_MACROPHAGE_UP	173	-0.45949	-1.80038	0	0.007647

GSE43955_TH0_VS_TGFB_IL6_IL23_TH17_ACT_CD4_TCELL_52H_UP	192	-0.38885	-1.80018	0	0.007653
GSE7852_LN_VS_FAT_TREG_UP	182	-0.32806	-1.79991	0	0.007663
GSE21774_CD56_BRIGHT_VS_DIM_CD62L_POSITIVE_NK_CELL_UP	182	-0.48614	-1.79951	0	0.007685
GSE3982_EFF_MEMORY_CD4_TCELL_VS_NKCELL_UP	186	-0.47064	-1.79888	0	0.007715
GSE8685_IL15_ACT_IL2_STARVED_VS_IL21_ACT_IL2_STARVED_CD4_TCELL_UP	157	-0.45921	-1.79871	0	0.00772
GSE369_PRE_VS_POST_IL6_INJECTION_IFNG_KO_LIVER_DN	183	-0.43282	-1.79824	0	0.007734
GSE28783_ANTI_MIR33_VS_CTRL_ATHEROSCLEROSIS_MACROPHAGE_UP	189	-0.53541	-1.79843	0	0.007735
GSE22589_SIV_VS_HIV_AND_SIV_INFECTED_DC_UP	181	-0.52542	-1.79833	0	0.007737
GSE40277_EOS_AND_LEF1_TRANSDUCED_VS_GATA1_AND_SATB1_TRANSDUCED_CD4_TCELL_DN	183	-0.44588	-1.79827	0	0.00774
GSE6674_UNSTIM_VS_CPG_STIM_BCELL_UP	135	-0.47366	-1.79807	0	0.00774
GSE5679_CTRL_VS_PPARG_LIGAND_ROSIGLITAZONE_TREATED_DC_DN	179	-0.41792	-1.79768	0	0.007759
GSE32423_CTRL_VS_IL4_MEMORY_CD8_TCELL_UP	176	-0.4488	-1.79739	0	0.007764

GSE9316_CD4_TCELL_BALBC_VS_TH17_ENRI_CD4_TCELL_SKG_PMA_IONO_STIM_FR4NEG_DN	182	-0.42097	-1.79678	0	0.007777
GSE43955_10H_VS_30H_ACT_CD4_TCELL_WITH_TGFB_IL6_DN	182	-0.46474	-1.79697	0	0.007777
GSE17974_CTRL_VS_ACT_IL4_AND_ANTI_IL12_24H_CD4_TCELL_UP	163	-0.53272	-1.79658	0	0.007789
GSE27786_LSK_VS_NEUTROPHIL_DN	177	-0.43895	-1.79619	0	0.007813
GSE25123_IL4_VS_IL4_AND_ROSIGLITAZONE_STIM_MACROPHAGE_DAY10_UP	191	-0.50859	-1.79605	0	0.007815
GSE6259_BCELL_VS_CD8_TCELL_UP	173	-0.47132	-1.7956	0	0.007848
GSE17721_PAM3CSK4_VS_CPG_2H_BMDC_UP	186	-0.46681	-1.79518	0	0.007861
GSE26495_PD1HIGH_VS_PD1LOW_CD8_TCELL_DN	154	-0.52702	-1.79535	0	0.007862
GSE5679_CTRL_VS_RARA_AGONIST_AM580_TREATED_DC_DN	181	-0.4097	-1.79468	0	0.007891
GSE17974_0H_VS_0.5H_IN_VITRO_ACT_CD4_TCELL_DN	160	-0.48205	-1.79446	0	0.007903
GSE37301_COMMON_LYMPHOID_PROGENITOR_VS_RAG2_KO_NK_CELL_UP	180	-0.49329	-1.79365	0	0.007945
GSE13306_TREG_VS_TCONV_LAMINA_PROPRIA_DN	174	-0.48682	-1.79369	0	0.007947

GSE26559_TCF1_KO_VS_WT_LIN_NEG_CELL_DN	179	-0.54911	-1.79352	0	0.007953
GSE2405_0H_VS_1.5H_A_PHAGOCYTOPHILUM_STIM_NEUTROPHIL_UP	188	-0.49598	-1.7934	0	0.007954
GSE26488_WT_VS_VP16_TRANSGENIC_HDAC7_KO_DOUBLE_POSITIVE_THYMOCYTE_UP	182	-0.46401	-1.79342	0	0.007958
GSE11961_MEMORY_BCELL_DAY7_VS_GERMINAL_CENTER_BCELL_DAY40_DN	176	-0.41466	-1.79323	0	0.00796
GSE10147_IL3_VS_IL3_AND_CPG_STIM_PDC_UP	137	-0.5728	-1.79311	0	0.007964
GSE37301_LYMPHOID_PRIMED_MPP_VS_COMMON_LYMPHOID_PROGENITOR_UP	194	-0.44977	-1.79327	0	0.007964
GSE24574_BCL6_HIGH_TFH_VS_TCONV_CD4_TCELL_DN	179	-0.52031	-1.79278	0	0.007972
GSE17721_CTRL_VS_POLYIC_8H_BMDC_DN	188	-0.43917	-1.79266	0	0.007972
GSE360_DC_VS_MAC_DN	182	-0.38563	-1.79287	0	0.007972
GSE28726_NAIVE_VS_ACTIVATED_NKTCELL_UP	184	-0.48429	-1.7928	0	0.007973
GSE9650_EFFECTOR_VS_EXHAUSTED_CD8_TCELL_DN	182	-0.4732	-1.79256	0	0.007973
GSE360_L_MAJOR_VS_B_MALAYI_LOW_DOSE_DC_UP	194	-0.44199	-1.7926	0	0.007976

GSE7460_TREG_VS_TCONV_ACT_WITH_TGFB_UP	177	-0.5062	-1.79221	0	0.007988
GSE17974_IL4_AND_ANTI_IL12_VS_UNTREATED_6H_ACT_CD4_TCELL_UP	153	-0.55548	-1.79226	0	0.007989
GSE7460_CTRL_VS_TGFB_TREATED_ACT_TREG_DN	184	-0.47423	-1.79196	0	0.007994
GSE17974_CTRL_VS_ACT_IL4_AND_ANTI_IL12_48H_CD4_TCELL_UP	161	-0.51122	-1.79169	0	0.007999
GSE12963_ENV_NEF_VS_ENV_NEF_AND_VPR_DEFICIENT_HIV1_INF_CD4_TCELL_UP	120	-0.4159	-1.79042	0	0.008068
GSE24574_NAIVE_VS_TCONV_CD4_TCELL_UP	186	-0.49755	-1.79001	0	0.008088
GSE9509_10MIN_VS_30MIN_LPS_STIM_IL10_KO_MACROPHAGE_DN	175	-0.51695	-1.79004	0	0.00809
GSE9946_IMMATURE_VS_PROSTAGLANDINE2_TREATED_MATURE_DC_UP	134	-0.49561	-1.78981	0	0.008095
GSE18893_CTRL_VS_TNF_TREATED_TREG_24H_UP	184	-0.42439	-1.78946	0	0.008113
GSE3982_BCELL_VS_CENT_MEMORY_CD4_TCELL_DN	174	-0.52562	-1.78889	0	0.008136
GSE360_T_GONDII_VS_M_TUBERCULOSIS_MAC_UP	182	-0.38111	-1.78869	0	0.008146
GSE13887_RESTING_VS_ACT_CD4_TCELL_DN	127	-0.43501	-1.78864	0	0.008148

GSE2770_UNTREATED_VS_TGFB_AND_IL4_TREATED_ACT_CD4_TCELL_4H_DN	132	-0.43776	-1.78835	0	0.008163
GSE5589_WT_VS_IL6_KO_LPS_AND_IL6_STIM_MACROPHAGE_45MIN_UP	184	-0.56045	-1.78821	0	0.008175
GSE43863_TFH_VS_LY6C_INT_CXCR5POS_EFFECTOR_CD4_TCELL_DN	183	-0.48343	-1.78802	0	0.008175
GSE23321_CENTRAL_VS_EFFECTOR_MEMORY_CD8_TCELL_DN	170	-0.33692	-1.78812	0	0.008175
GSE3203_INFLUENZA_INF_VS_IFNB_TREATED_LN_BCELL_UP	177	-0.46991	-1.7879	0	0.008184
GSE360_T_GONDII_VS_B_MALAYI_LOW_DOSE_DC_UP	183	-0.4793	-1.78713	0	0.008218
GSE3982_BASOPHIL_VS_NKCELL_DN	185	-0.46352	-1.78709	0	0.008218
GSE22935_UNSTIM_VS_12H_MBOVIS_BCG_STIM_MYD88_KO_MACROPHAGE_UP	184	-0.49844	-1.78643	0	0.00826
GSE3982_EFF_MEMORY_CD4_TCELL_VS_TH1_UP	178	-0.4818	-1.78585	0	0.008303
GSE20152_HTNFA_OVERXPRESS_ANKLE_VS_CTRL_SPHK1_KO_ANKLE_UP	183	-0.46342	-1.78541	0	0.008327
GSE13411_NAIVE_VS_IGM_MEMORY_BCELL_DN	179	-0.36351	-1.78439	0	0.008376
GSE39820_CTRL_VS_IL1B_IL6_CD4_TCELL_DN	180	-0.48538	-1.78357	0	0.008409

GSE6259_BCELL_VS_CD4_TCELL_DN	175	-0.3869	-1.78359	0	0.008412
GSE31622_WT_VS_KLF3_KO_BCELL_DN	185	-0.44283	-1.78327	0	0.008419
GSE17721_CTRL_VS_LPS_6H_BMDC_UP	182	-0.29503	-1.7833	0	0.008421
GSE17721_CTRL_VS_GARDIQUIMOD_8H_BMDC_DN	186	-0.4043	-1.78226	0	0.008476
GSE27786_BCELL_VS_NKTCELL_DN	178	-0.40298	-1.78227	0	0.00848
GSE17721_CTRL_VS_GARDIQUIMOD_2H_BMDC_DN	183	-0.40691	-1.78246	0	0.008483
GSE17301_CTRL_VS_48H_ACD3_ACD28_IFNA5_STIM_CD8_TCELL_UP	182	-0.36854	-1.78244	0	0.008484
GSE25088_CTRL_VS_ROSIGLITAZONE_STIM_MACROPHAGE_UP	182	-0.4465	-1.78232	0	0.008487
GSE45365_NK_CELL_VS_BCELL_DN	130	-0.55014	-1.78173	0	0.008519
GSE360_CTRL_VS_B_MALAYI_HIGH_DOSE_DC_UP	182	-0.40961	-1.78146	0	0.008537
GSE43863_DAY6_EFF_VS_DAY150_MEM_TFH_CD4_TCELL_DN	186	-0.5532	-1.78092	0	0.008573
GSE17721_CTRL_VS_LPS_12H_BMDC_UP	174	-0.34973	-1.78093	0	0.008576

GSE39152_SPLEEN_CD103_NEG_VS_BRAIN_CD103_POS_MEMORY_CD8_TCELL_UP	187	-0.40811	-1.78076	0	0.008579
GSE29618_PRE_VS_DAY7_POST_LAIV_FLU_VACCINE_MONOCYTE_UP	181	-0.44812	-1.77998	0	0.008627
GSE4590_SMALL_VS_VPREB_POS_LARGE_PRE_BCELL_DN	147	-0.5117	-1.77958	0	0.008655
GSE360_L_DONOVANI_VS_B_MALAYI_HIGH_DOSE_DC_UP	185	-0.50227	-1.77926	0	0.008656
GSE360_L_MAJOR_VS_B_MALAYI_HIGH_DOSE_DC_UP	193	-0.45622	-1.77931	0	0.008658
GSE17974_0H_VS_48H_IN_VITRO_ACT_CD4_TCELL_UP	162	-0.52006	-1.77917	0	0.008659
GSE22611_MUTANT_NOD2_TRANSDUCED_VS_CTRL_HEK293T_STIMULATED_WITH_MDP_6H_UP	177	-0.49772	-1.77852	0	0.008659
GSE28737_FOLLICULAR_VS_MARGINAL_ZONE_BCELL_BCL6_HET_DN	181	-0.49199	-1.77858	0	0.008662
GSE37301_MULTIPOTENT_PROGENITOR_VS_PRO_BCELL_UP	184	-0.43668	-1.77905	0	0.008666
GSE17721_PAM3CSK4_VS_CPG_16H_BMDC_DN	178	-0.43124	-1.77882	0	0.00867
GSE43863_TH1_VS_LY6C_INT_CXCR5POS_MEMORY_CD4_TCELL_DN	179	-0.53609	-1.77831	0	0.008671
GSE13306_TREG_VS_TCONV_UP	183	-0.50917	-1.77885	0	0.008673

GSE34156_TLR1_TLR2_LIGAND_VS_NOD2_AND_TLR1_TLR2_LIGAND_6H_TREATED_MONOCYTE_UP	185	-0.485	-1.77894	0	0.008673
GSE45365_HEALTHY_VS_MCMV_INFECTION_CD8_TCELL_IFNAR_KO_DN	153	-0.53331	-1.77811	0	0.008684
GSE19888_CTRL_VS_A3R_ACT_TREATED_MAST_CELL_PRETREATED_WITH_A3R_INH_UP	184	-0.50891	-1.77802	0	0.008694
GSE3400_UNTREATED_VS_IFNB_TREATED_MEF_DN	155	-0.50151	-1.7778	0	0.008705
GSE36078_WT_VS_IL1R_KO_LUNG_DC_AFTER_AD5_T425A_HEXON_INF_UP	180	-0.42825	-1.77739	0	0.00873
GSE5542_IFNG_VS_IFNA_TREATED_EPITHELIAL_CELLS_24H_DN	177	-0.48649	-1.7771	0	0.008739
GSE23308_WT_VS_MINERALCORTICOID_REC_KO_MACROPHAGE_DN	185	-0.4765	-1.77717	0	0.008742
GSE20715_WT_VS_TLR4_KO_24H_OZONE_LUNG_UP	182	-0.49	-1.77672	0	0.00876
GSE13484_12H_UNSTIM_VS_YF17D_VACCINE_STIM_PBMC_DN	189	-0.48448	-1.77659	0	0.008766
GSE42021_TREG_PLN_VS_CD24LO_TREG_THYMUS_UP	169	-0.36929	-1.77647	0	0.00877
GSE19772_CTRL_VS_HCMV_INF_MONOCYTES_DN	156	-0.46284	-1.77602	0	0.008791
GSE40184_HEALTHY_VS_HCV_INFECTED_DONOR_PBMC_UP	182	-0.45979	-1.77595	0	0.008792

GSE17721_CTRL_VS_LPS_0.5H_BMDC_UP	177	-0.4204	-1.77497	0	0.008852
GSE20727_ROS_INH_VS_ROS_INH_AND_DNFB_ALLERGEN_TREATED_DC_DN	166	-0.56179	-1.77498	0	0.008855
GSE9509_LPS_VS_LPS_AND_IL10_STIM_IL10_KO_MACROPHAGE_20MIN_UP	164	-0.53186	-1.77428	0	0.008889
GSE32128_INOS_DEPENDENT_VS_INOS_INDEPENDENT_ACTIVATED_TCELL_DN	182	-0.39112	-1.77366	0	0.008909
GSE42021_CD24INT_VS_CD24LOW_TCONV_THYMUS_UP	178	-0.45142	-1.7734	0	0.008911
GSE22229_UNTREATED_VS_IMMUNOSUPP_THERAPY_RENAL_TRANSPLANT_PATIENT_PBMC_DN	183	-0.46437	-1.77353	0	0.008914
GSE14308_TH17_VS_NATURAL_TREG_DN	177	-0.37687	-1.77322	0	0.008925
GSE21546_UNSTIM_VS_ANTI_CD3_STIM_SAP1A_KO_AND_ELK1_KO_DP_THYMOCYTES_UP	152	-0.51231	-1.77286	0	0.008937
GSE25502_WT_VS_KLF13_KO_THYMIC_MEMORY_LIKE_CD8_TCELL_UP	166	-0.43178	-1.77293	0	0.008937
GSE13522_CTRL_VS_T_CRUZI_Y_STRAIN_INF_SKIN_BALBC_MOUSE_DN	125	-0.52762	-1.77241	0	0.008964
GSE11057_NAIVE_VS_MEMORY_CD4_TCELL_UP	160	-0.48766	-1.7715	0	0.009029
GSE43955_1H_VS_60H_ACT_CD4_TCELL_WITH_TGFB_IL6_DN	184	-0.42759	-1.77151	0	0.009033

GSE19888_ADENOSINE_A3R_ACT_VS_A3R_ACT_WITH_A3R_INH_PRETREATMENT_IN_MAST_CELL_DN	183	-0.46845	-1.77114	0	0.009048
GSE5589_UNSTIM_VS_45MIN_LPS_AND_IL10_STIM_MACROPHAGE_UP	179	-0.34658	-1.77097	0	0.009053
GSE17721_12H_VS_24H_CPG_BMDC_UP	182	-0.46058	-1.77088	0	0.009054
GSE16385_IFNG_TNF_VS_ROSIGLITAZONE_STIM_MACROPHAGE_UP	154	-0.38049	-1.77079	0	0.009062
GSE13522_CTRL_VS_T_CRUZI_G_STRAIN_INF_SKIN_DN	127	-0.48714	-1.77058	0	0.009075
GSE16385_ROSIGLITAZONE_VS_UNTREATED_IFNG_TNF_STIM_MACROPHAGE_UP	180	-0.46556	-1.77005	0	0.009105
GSE24574_BCL6_LOW_TFH_VS_NAIVE_CD4_TCELL_DN	179	-0.47006	-1.76992	0	0.009108
GSE40666_NAIVE_VS_EFFECTOR_CD8_TCELL_UP	182	-0.45239	-1.76927	0	0.009142
GSE13306_TREG_VS_TCONV_SPLEEN_UP	179	-0.5076	-1.76902	0	0.009155
GSE29615_CTRL_VS_DAY3_LAIV_IFLU_VACCINE_PBMC_DN	160	-0.4236	-1.76897	0	0.009156
GSE1791_CTRL_VS_NEUROMEDINU_IN_T_CELL_LINE_0.8H_DN	161	-0.5255	-1.76891	0	0.009157
GSE24492_LYVE_NEG_VS_POS_MACROPHAGE_UP	176	-0.43414	-1.76861	0	0.009177

GSE42021_CD24INT_VS_CD24LOW_TREG_THYMUS_UP	178	-0.44776	-1.76853	0	0.009177
GSE11864_CSF1_VS_CSF1_IFNG_IN_MAC_DN	170	-0.39033	-1.76779	0	0.00922
GSE15733_BM_VS_SPLEEN_MEMORY_CD4_TCELL_UP	187	-0.54763	-1.76766	0	0.009227
GSE360_L_DONOVANI_VS_L_MAJOR_MAC_UP	182	-0.33893	-1.76753	0	0.009234
GSE23505_UNTREATED_VS_4DAY_IL6_IL1_TREATED_CD4_TCELL_DN	183	-0.49095	-1.76665	0	0.009288
GSE21546_UNSTIM_VS_ANTI_CD3_STIM_SAP1A_KO_DP_THYMOCYTES_UP	172	-0.46102	-1.76655	0	0.009291
GSE27786_BCELL_VS_CD8_TCELL_DN	174	-0.40418	-1.76645	0	0.009291
GSE26928_EFF_MEMORY_VS_CXCR5_POS_CD4_TCELL_DN	155	-0.44845	-1.76614	0	0.0093
GSE25890_CTRL_VS_IL33_IL7_TREATED_NUOCYTES_UP	183	-0.42331	-1.76544	0	0.009332
GSE3203_WT_VS_IFNAR1_KO_INFLUENZA_INFECTED_LN_BCELL_UP	183	-0.49857	-1.76505	0	0.009352
GSE9601_UNTREATED_VS_NFKB_INHIBITOR_TREATED_HCMV_INF_MONOCYTE_UP	155	-0.52333	-1.76488	0	0.009358
GSE21546_UNSTIM_VS_ANTI_CD3_STIM_SAP1A_KO_AND_ELK1_KO_DP_THYMOCYTES_DN	136	-0.56306	-1.76463	0	0.009368

GSE13522_CTRL_VS_T_CRUZI_BRAZIL_STRAIN_INF_SKIN_DN	184	-0.51978	-1.76439	0	0.009379
GSE21063_WT_VS_NFATC1_KO_16H_ANTI_IGM_STIM_BCELL_DN	164	-0.55024	-1.76414	0	0.009395
GSE15930_NAIVE_VS_72H_IN_VITRO_STIM_IFNAB_CD8_TCELL_UP	189	-0.40817	-1.76389	0	0.009402
GSE8685_IL2_ACT_IL2_STARVED_VS_IL21_ACT_IL2_STARVED_CD4_TCELL_DN	136	-0.49614	-1.76337	0	0.009431
GSE17721_LPS_VS_CPG_24H_BMDC_DN	188	-0.42124	-1.76311	0	0.009461
GSE17721_PAM3CSK4_VS_CPG_24H_BMDC_UP	181	-0.38309	-1.76212	0	0.009487
GSE17721_LPS_VS_CPG_12H_BMDC_DN	182	-0.4361	-1.76205	0	0.009488
GSE22611_UNSTIM_VS_6H_MDP_STIM_MUTANT_NOD2_TRANSDUCED_HEK293T_CELL_DN	175	-0.44791	-1.76236	0	0.009495
GSE11961_FOLLICULAR_BCELL_VS_MEMORY_BCELL_DAY40_DN	184	-0.45745	-1.76245	0	0.009496
GSE360_DC_VS_MAC_L_MAJOR_UP	184	-0.40331	-1.7619	0	0.009499
GSE3982_NEUTROPHIL_VS_BCELL_UP	188	-0.49899	-1.76052	0	0.00959
GSE37301_LYMPHOID_PRIMED_MPP_VS_CD4_TCELL_UP	171	-0.51189	-1.76021	0	0.009607

GSE4535_BM_DERIVED_DC_VS_FOLLICULAR_DC_DN	178	-0.42992	-1.75993	0	0.009609
GSE2585_CD80_HIGH_VS_LOW_MTEC_DN	178	-0.46722	-1.76007	0	0.009609
GSE23925_LIGHT_ZONE_VS_NAIVE_BCELL_DN	174	-0.3157	-1.75983	0	0.009611
GSE17721_POLYIC_VS_CPG_0.5H_BMDC_UP	185	-0.48038	-1.75939	0	0.00963
GSE17721_LPS_VS_POLYIC_2H_BMDC_UP	192	-0.43535	-1.75949	0	0.009631
GSE17721_POLYIC_VS_GARDIQUIMOD_4H_BMDC_UP	181	-0.3396	-1.75941	0	0.009632
GSE2585_THYMIC_DC_VS_THYMIC_MACROPHAGE_UP	172	-0.46107	-1.75853	0	0.009684
GSE17721_CTRL_VS_LPS_2H_BMDC_DN	183	-0.44665	-1.75836	0	0.00969
GSE17721_CTRL_VS_CPG_2H_BMDC_UP	179	-0.28611	-1.75734	0	0.009759
GSE41978_WT_VS_BIM_KO_KLRG1_LOW_EFFECTOR_CD8_TCELL_DN	179	-0.41843	-1.75688	0	0.009787
GSE21927_BALBC_VS_C57BL6_MONOCYTE_SPLEEN_UP	156	-0.43492	-1.75625	0	0.009818
GSE25088_CTRL_VS_IL4_STIM_MACROPHAGE_DN	176	-0.47129	-1.75612	0	0.009824

GSE20152_SPHK1_KO_VS_HTNFA_OVEREXPRESS_ANKLE_UP	176	-0.40962	-1.75601	0	0.009828
GSE17721_CTRL_VS_POLYIC_2H_BMDC_DN	187	-0.44037	-1.75564	0	0.009841
GSE3720_LPS_VS_PMA_STIM_VD2_GAMMADELTA_TCELL_DN	143	-0.53213	-1.75484	0	0.009891
GSE5589_WT_VS_IL6_KO_LPS_STIM_MACROPHAGE_180MIN_DN	123	-0.51598	-1.75493	0	0.009893
GSE15659_CD45RA_NEG_CD4_TCELL_VS_RESTING_TREG_UP	161	-0.47602	-1.75421	0	0.009923
GSE46606_IRF4HIGH_VS_IRF4MID_CD40L_IL2_IL5_DAY1_STIMULATED_BCELL_DN	181	-0.522	-1.7543	0	0.009926
GSE13229_MATURE_VS_INTMATURE_NKCELL_UP	177	-0.54267	-1.75316	0	0.009985
GSE9037_WT_VS_IRAK4_KO_LPS_4H_STIM_BMDM_DN	187	-0.42064	-1.75288	0	0.009994
GSE11924_TH1_VS_TH2_CD4_TCELL_DN	178	-0.42407	-1.75236	0	0.010018
GSE19941_LPS_VS_LPS_AND_IL10_STIM_IL10_KO_NFKBP50_KO_MACROPHAGE_UP	183	-0.45127	-1.75249	0	0.01002
GSE17721_LPS_VS_POLYIC_1H_BMDC_DN	179	-0.33451	-1.7522	0	0.010026
GSE15930_STIM_VS_STIM_AND_IFNAB_24H_CD8_T_CELL_DN	186	-0.41611	-1.75104	0	0.010116

GSE2128_C57BL6_VS_NOD_THYMOCYTE_DN	170	-0.43635	-1.75088	0	0.010123
GSE43863_NAIVE_VS_TFH_CD4_EFF_TCELL_D6_LCMV_DN	149	-0.55279	-1.75083	0	0.010126
GSE17974_IL4_AND_ANTI_IL12_VS_UNTREATED_1H_ACT_CD4_TCELL_UP	146	-0.54261	-1.75051	0	0.010129
GSE22342_CD11C_HIGH_VS_LOW_DECIDUAL_MACROPHAGES_DN	162	-0.52493	-1.75052	0	0.010133
GSE27786_NKTCELL_VS_NEUTROPHIL_DN	185	-0.40699	-1.75063	0	0.010138
GSE34156_TLR1_TLR2_LIGAND_VS_NOD2_AND_TLR1_TLR2_LIGAND_24H_TREATED_MONOCYTE_DN	177	-0.43787	-1.75023	0	0.010146
GSE19401_PLN_VS_PEYERS_PATCH_FOLLICULAR_DC_UP	185	-0.41617	-1.74988	0	0.010163
GSE2405_0H_VS_3H_A_PHAGOCYTOPHILUM_STIM_NEUTROPHIL_DN	159	-0.56961	-1.74972	0	0.010165
GSE20366_TREG_VS_NAIVE_CD4_TCELL_DEC205_CONVERSION_DN	182	-0.498	-1.74979	0	0.010166
GSE22611_NOD2_VS_CTRL_TRANSDUCED_HEK293T_CELL_UP	175	-0.32165	-1.74973	0	0.010167
GSE11924_TH1_VS_TH17_CD4_TCELL_DN	176	-0.38208	-1.7493	0	0.010196
GSE26495_NAIVE_VS_PD1HIGH_CD8_TCELL_UP	154	-0.52498	-1.74935	0	0.010199

GSE21063_CTRL_VS_ANTI_IGM_STIM_BCELL_16H_DN	183	-0.50057	-1.74878	0	0.010237
GSE15930_STIM_VS_STIM_AND_IFNAB_72H_CD8_T_CELL_UP	178	-0.48002	-1.74843	0	0.010255
GSE360_CTRL_VS_B_MALAYI_LOW_DOSE_DC_DN	188	-0.47751	-1.74778	0	0.010301
GSE20198_UNTREATED_VS_IFNA_TREATED_ACT_CD4_TCELL_UP	172	-0.40599	-1.7478	0	0.010302
GSE3982_EOSINOPHIL_VS_CENT_MEMORY_CD4_TCELL_DN	182	-0.31549	-1.74782	0	0.010304
GSE26928_CENTR_MEMORY_VS_CXCR5_POS_CD4_TCELL_UP	161	-0.50058	-1.747	0	0.010373
GSE11924_TH2_VS_TH17_CD4_TCELL_UP	175	-0.46176	-1.74672	0	0.010394
GSE14308_TH17_VS_NAIVE_CD4_TCELL_DN	180	-0.38111	-1.74664	0	0.010397
GSE27291_0H_VS_6H_STIM_GAMMADELTA_TCELL_UP	160	-0.41941	-1.74613	0	0.010437
GSE5589_WT_VS_IL10_KO_LPS_AND_IL10_STIM_MACROPHAGE_45MIN_DN	177	-0.35069	-1.74552	0	0.010467
GSE30971_CTRL_VS_LPS_STIM_MACROPHAGE_WBP7_HET_4H_UP	181	-0.41053	-1.74529	0	0.010475
GSE17974_IL4_AND_ANTI_IL12_VS_UNTREATED_24H_ACT_CD4_TCELL_UP	152	-0.54785	-1.74455	0	0.010536

GSE36009_UNSTIM_VS_LPS_STIM_NLRP10_KO_DC_DN	172	-0.31874	-1.7443	0	0.010554
GSE17721_POLYIC_VS_GARDIQUIMOD_1H_BMDC_UP	184	-0.3875	-1.74408	0	0.010559
GSE12198_LOW_IL2_STIM_NK_CELL_VS_HIGH_IL2_STIM_NK_CELL_DN	181	-0.47558	-1.74395	0	0.010562
GSE42021_CD24INT_TREG_VS_CD24INT_TCONV_THYMUS_DN	183	-0.46409	-1.74401	0	0.010564
GSE17721_PAM3CSK4_VS_CPG_1H_BMDC_DN	185	-0.38634	-1.74367	0	0.010577
GSE24492_LYVE_NEG_VS_POS_MACROPHAGE_DN	180	-0.50085	-1.7429	0	0.010616
GSE17721_4_VS_24H_CPG_BMDC_UP	187	-0.44808	-1.7413	0	0.010704
GSE46606_IRF4_KO_VS_WT_CD40L_IL2_IL5_1DAY_STIMULATED_BCELL_DN	171	-0.47615	-1.74105	0	0.010722
GSE40685_NAIVE_CD4_TCELL_VS_FOXP3_KO_TREG_PRECURSOR_UP	181	-0.41898	-1.74075	0	0.010736
GSE22886_IGA_VS_IGM_MEMORY_BCELL_UP	177	-0.42936	-1.74038	0	0.010763
GSE360_L_MAJOR_VS_M_TUBERCULOSIS_DC_DN	187	-0.43232	-1.74041	0	0.010764
GSE20366_EX_VIVO_VS_HOMEOSTATIC_CONVERSION_NAIVE_CD4_TCELL_DN	184	-0.47841	-1.73979	0	0.010801

GSE30083_SP2_VS_SP3_THYMOCYTE_UP	190	-0.51214	-1.7396	0	0.010815
GSE13522_CTRL_VS_T_CRUZI_BRAZIL_STRAIN_INF_SKIN_UP	129	-0.49092	-1.73614	0	0.011094
GSE11961_MEMORY_BCELL_DAY7_VS_MEMORY_BCELL_DAY40_DN	184	-0.38585	-1.73611	0	0.011099
GSE21546_WT_VS_SAP1A_KO_AND_ELK1_KO_ANTI_CD3_STIM_DP_THYMOCYTES_DN	178	-0.45308	-1.73625	0	0.0111
GSE6259_33D1_POS_DC_VS_BCELL_UP	151	-0.46465	-1.73567	0	0.011127
GSE16385_UNTREATED_VS_12H_IL4_TREATED_MACROPHAGE_DN	174	-0.42592	-1.73543	0	0.011141
GSE43955_TH0_VS_TGFB_IL6_TH17_ACT_CD4_TCELL_42H_DN	189	-0.46407	-1.7348	0	0.011177
GSE15659_NAIVE_VS_PTPRC_NEG_CD4_TCELL_UP	151	-0.45595	-1.73474	0	0.01118
GSE43955_1H_VS_60H_ACT_CD4_TCELL_DN	193	-0.43979	-1.73434	0	0.011193
GSE7460_CTRL_VS_FOXP3_OVEREXPR_TCONV_DN	174	-0.41083	-1.73443	0	0.011197
GSE16385_MONOCYTE_VS_MACROPHAGE_DN	170	-0.50675	-1.73195	0	0.011387
GSE32034_LY6C_HIGH_VS_LOW_ROSIGLIZATONE_TREATED_MONOCYTE_DN	191	-0.43037	-1.7312	0	0.011438

GSE15659_RESTING_VS_ACTIVATED_TREG_DN	179	-0.36781	-1.73106	0	0.011444
GSE36095_WT_VS_HDAC9_KO_TREG_DN	115	-0.57003	-1.73096	0	0.01145
GSE21379_TFH_VS_NON_TFH_CD4_TCELL_UP	178	-0.40821	-1.73054	0	0.011487
GSE26351_WNT_VS_BMP_PATHWAY_STIM_HEMATOPOIETIC_PROGENITORS_DN	175	-0.28682	-1.73056	0	0.01149
GSE29164_DAY3_VS_DAY7_CD8_TCELL_AND_IL12_TREATED_MELANOMA_DN	187	-0.50155	-1.73012	0	0.011507
GSE3982_MAC_VS_EFF_MEMORY_CD4_TCELL_DN	181	-0.44914	-1.73014	0	0.011509
GSE22935_UNSTIM_VS_48H_MBOVIS_BCG_STIM_MACROPHAGE_UP	179	-0.52017	-1.73003	0	0.011512
GSE16385_ROSIGLITAZONE_IL4_VS_ROSIGLITAZONE_ALONE_STIM_MACROPHAGE_UP	177	-0.51358	-1.72945	0	0.011526
GSE19888_CTRL_VS_TCELL_MEMBRANES_ACT_MAST_CELL_PRETREAT_A3R_INH_UP	179	-0.48593	-1.72947	0	0.011528
GSE17721_PAM3CSK4_VS_GADIQUIMOD_24H_BMDC_UP	185	-0.38172	-1.72953	0	0.011533
GSE5589_WT_VS_IL10_KO_LPS_STIM_MACROPHAGE_180MIN_DN	141	-0.50637	-1.72837	0	0.011625
GSE20715_0H_VS_6H_OZONE_TLR4_KO_LUNG_UP	185	-0.48613	-1.72824	0	0.01163

GSE25088_CTRL_VS_IL4_STIM_MACROPHAGE_UP	170	-0.44203	-1.72552	0	0.01185
GSE39820_IL1B_IL6_VS_IL1B_IL6_IL23A_TREATED_CD4_TCELL_UP	174	-0.49779	-1.7252	0	0.011871
GSE360_L_DONOVANI_VS_L_MAJOR_DC_DN	185	-0.42096	-1.72474	0	0.01191
GSE22611_NOD2_TRANSD_VS_CTRL_TRANSD_HEK293_MDP_STIM_2H_UP	172	-0.50218	-1.72435	0	0.011943
GSE22432_MULTIPOTENT_PROGENITOR_VS_CDC_UP	182	-0.45898	-1.72445	0	0.011943
GSE29949_MICROGLIA_BRAIN_VS_CD8_NEG_DC_SPLEEN_DN	180	-0.50376	-1.72407	0	0.011965
GSE21927_BALBC_VS_C57BL6_MONOCYTE_TUMOR_DN	161	-0.38901	-1.72378	0	0.011992
GSE29949_MICROGLIA_VS_DC_BRAIN_DN	183	-0.50282	-1.72369	0	0.011997
GSE9006_TYPE_1_DIABETES_AT_DX_VS_1MONTH_POST_DX_PBMC_DN	167	-0.38869	-1.72352	0	0.012006
GSE19401_RETINOIC_ACID_VS_RETINOIC_ACID_AND_PAM2CSK4_STIM_FOLLICULAR_DC_UP	179	-0.46896	-1.72315	0	0.012034
GSE17721_0.5H_VS_12H_GARDIQUIMOD_BMDC_UP	180	-0.32047	-1.72273	0	0.012079
GSE8621_UNSTIM_VS_LPS_PRIMED_UNSTIM_MACROPHAGE_DN	176	-0.34709	-1.72245	0	0.012091

GSE2770_IL12_VS_IL4_TREATED_ACT_CD4_TCELL_6H_UP	181	-0.41362	-1.72223	0	0.012101
GSE2585_CTEC_VS_THYMIC_DC_DN	192	-0.46227	-1.72107	0	0.012197
GSE1448_ANTI_VALPHA2_VS_VBETA5_DP_THYMOCYTE_DN	182	-0.41384	-1.72065	0	0.012222
GSE2770_TGFB_AND_IL4_VS_IL12_TREATED_ACT_CD4_TCELL_2H_UP	167	-0.37723	-1.71944	0	0.012297
GSE17721_LPS_VS_PAM3CSK4_0.5H_BMDC_DN	183	-0.41369	-1.71908	0	0.012319
GSE15659_TREG_VS_TCONV_UP	156	-0.46813	-1.71872	0	0.012344
GSE20366_CD103_POS_VS_CD103_KLRG1_DP_TREG_UP	178	-0.44266	-1.71861	0	0.012354
GSE17721_CPG_VS_GARDIQUIMOD_16H_BMDC_DN	186	-0.35937	-1.71846	0	0.012365
GSE13547_CTRL_VS_ANTI_IGM_STIM_ZFX_KO_BCELL_12H_UP	134	-0.44429	-1.71794	0	0.012396
GSE32423_MEMORY_VS_NAIVE_CD8_TCELL_IL7_IL4_UP	177	-0.52464	-1.71771	0	0.012411
GSE17721_CTRL_VS_GARDIQUIMOD_4H_BMDC_DN	181	-0.43721	-1.71748	0	0.01243
GSE13229_IMM_VS_INTMATURE_NKCELL_UP	173	-0.51173	-1.71732	0	0.012433

GSE30083_SP1_VS_SP3_THYMOCYTE_UP	181	-0.36796	-1.71716	0	0.012441
GSE29949_CD8_NEG_DC_SPLEEN_VS_MONOCYTE_BONE_MARROW_DN	183	-0.4425	-1.71707	0	0.012444
GSE46606_DAY1_VS_DAY3_CD40L_IL2_IL5_STIMULATED_BCELL_UP	155	-0.5178	-1.71695	0	0.012452
GSE32901_TH1_VS_TH17_NEG_CD4_TCELL_DN	114	-0.50831	-1.71672	0	0.012464
GSE15659_NAIVE_CD4_TCELL_VS_NONSUPPRESSIVE_TCELL_UP	153	-0.46176	-1.71599	0	0.012512
GSE2770_UNTREATED_VS_TGFB_AND_IL4_TREATED_ACT_CD4_TCELL_48H_UP	144	-0.4851	-1.71602	0	0.012516
GSE25123_WT_VS_PPARG_KO_MACROPHAGE_ROSIGLITAZONE_STIM_DN	149	-0.51403	-1.71576	0	0.012529
GSE43863_NAIVE_VS_LY6C_INT_CXCR5POS_CD4_EFF_TCELL_D6_LCMV_DN	150	-0.54271	-1.71549	0	0.012554
GSE17974_0H_VS_0.5H_IN_VITRO_ACT_CD4_TCELL_UP	165	-0.49258	-1.71524	0	0.012584
GSE7218_UNSTIM_VS_ANTIGEN_STIM_THROUGH_IGM_BCELL_DN	112	-0.50057	-1.7151	0	0.012589
GSE37319_WT_VS_RC3H1_KO_CD44LOW_CD8_TCELL_DN	132	-0.47664	-1.71427	0	0.012626
GSE24210_IL35_TREATED_VS_RESTING_TREG_UP	175	-0.50959	-1.71413	0	0.012633

GSE27786_BCELL_VS_NEUTROPHIL_DN	190	-0.44516	-1.71415	0	0.012634
GSE17721_CTRL_VS_LPS_1H_BMDC_DN	183	-0.45501	-1.71353	0	0.012661
GSE29618_PRE_VS_DAY7_FLU_VACCINE_MDC_UP	176	-0.50991	-1.71344	0	0.01267
GSE19198_1H_VS_6H_IL21_TREATED_TCELL_UP	149	-0.58324	-1.71249	0	0.012746
GSE19825_NAIVE_VS_IL2RAHIGH_DAY3_EFF_CD8_TCELL_UP	175	-0.57096	-1.71194	0	0.012783
GSE19888_ADENOSINE_A3R_ACT_VS_TCELL_MEMBRANES_ACT_AND_A3R_INH_PRETREAT_IN_MAST_CELL_UP	170	-0.41899	-1.71179	0	0.012785
GSE16450_IMMATURE_VS_MATURE_NEURON_CELL_LINE_6H_IFNA_STIM_UP	186	-0.50264	-1.71179	0	0.012789
GSE29949_CD8_POS_DC_SPLEEN_VS_DC_BRAIN_DN	184	-0.50493	-1.71119	0	0.012828
GSE43955_TH0_VS_TGFB_IL6_TH17_ACT_CD4_TCELL_52H_DN	188	-0.44371	-1.71006	0	0.012938
GSE28737_WT_VS_BCL6_KO_MARGINAL_ZONE_BCELL_UP	178	-0.5011	-1.70978	0	0.012952
GSE15659_CD45RA_NEG_CD4_TCELL_VS_NONSUPPRESSIVE_TCELL_UP	147	-0.47158	-1.70985	0	0.012952
GSE17721_LPS_VS_CPG_0.5H_BMDC_UP	181	-0.33412	-1.7095	0	0.012964

GSE39820_IL1B_IL6_VS_IL1B_IL6_IL23A_TREATED_CD4_TCELL_DN	181	-0.47107	-1.70952	0	0.012966
GSE3982_NEUTROPHIL_VS_BASOPHIL_UP	179	-0.42022	-1.7092	0	0.012992
GSE22443_NAIVE_VS_ACT_AND_IL2_TREATED_CD8_TCELL_UP	189	-0.52067	-1.7086	0	0.013032
GSE22886_NEUTROPHIL_VS_DC_UP	175	-0.55473	-1.70829	0	0.013049
GSE3982_EFF_MEMORY_CD4_TCELL_VS_TH2_UP	181	-0.45347	-1.70812	0	0.013057
GSE37336_LY6C_POS_VS_NEG_NAIVE_CD4_TCELL_DN	143	-0.58668	-1.70656	0	0.013186
GSE32986_GMCSF_AND_CURDLAN_LOWDOSE_VS_GMCSF_AND_CURDLAN_HIGHDOSE_STIM_DC_DN	170	-0.49679	-1.70657	0	0.013191
GSE37605_TREG_VS_TCONV_C57BL6_FOXP3_FUSION_GFP_UP	129	-0.53312	-1.70605	0	0.013236
GSE21927_UNTREATED_VS_GMCSF_GCSF_TREATED_BONE_MARROW_DN	158	-0.40351	-1.70411	0	0.013439
GSE12484_HEALTHY_VS_PERIDONTITIS_NEUTROPHILS_UP	127	-0.57287	-1.70398	0	0.013449
GSE17721_POLYIC_VS_PAM3CSK4_0.5H_BMDC_DN	185	-0.31895	-1.70373	0	0.013469
GSE17974_IL4_AND_ANTI_IL12_VS_UNTREATED_2H_ACT_CD4_TCELL_DN	157	-0.51178	-1.70321	0	0.013497

GSE22601_DOUBLE_NEGATIVE_VS_CD8_SINGLE_POSITIVE_THYMOCYTE_DN	181	-0.46848	-1.70291	0	0.013511
GSE22886_TH1_VS_TH2_12H_ACT_DN	179	-0.45058	-1.70247	0	0.013531
GSE30971_2H_VS_4H_LPS_STIM_MACROPHAGE_WBP7_HET_DN	149	-0.52113	-1.70252	0	0.013542
GSE36476_CTRL_VS_TSST_ACT_40H_MEMORY_CD4_TCELL_OLD_UP	176	-0.48735	-1.70255	0	0.013544
GSE14026_TH1_VS_TH17_UP	182	-0.48361	-1.70206	0	0.013559
GSE32533_WT_VS_MIR17_OVEREXPRESS_ACT_CD4_TCELL_DN	185	-0.53275	-1.70199	0	0.013561
GSE41176_UNSTIM_VS_ANTI_IGM_STIM_TAK1_KO_BCELL_6H_DN	175	-0.5846	-1.70175	0	0.013579
GSE23502_BM_VS_COLON_TUMOR_HDC_KO_MYELOID_DERIVED_SUPPRESSOR_CELL_UP	180	-0.55767	-1.70086	0	0.013672
GSE17721_PAM3CSK4_VS_GADIQUIMOD_4H_BMDC_UP	184	-0.3825	-1.7007	0	0.013679
GSE3982_NKCELL_VS_TH1_UP	175	-0.48812	-1.7006	0	0.013688
GSE16385_UNTREATED_VS_12H_IL4_TREATED_MACROPHAGE_UP	186	-0.4406	-1.7003	0	0.013714
GSE23321_CENTRAL_MEMORY_VS_NAIVE_CD8_TCELL_DN	188	-0.51841	-1.69943	0	0.013811

GSE5589_WT_VS_IL10_KO_LPS_AND_IL6_STIM_MACROPHAGE_45MIN_UP	179	-0.47799	-1.69888	0	0.013866
GSE4142_PLASMA_CELL_VS_MEMORY_BCELL_UP	179	-0.5375	-1.69876	0	0.013873
GSE6090_UNSTIM_VS_DC_SIGN_STIM_DC_DN	135	-0.57899	-1.69866	0	0.01388
GSE4984_GALECTIN1_VS_LPS_STIM_DC_DN	156	-0.37738	-1.69845	0	0.013893
GSE28783_ANTI_MIR33_VS_UNTREATED_ATHEROSCLEROSIS_MACROPHAGE_UP	184	-0.42627	-1.69766	0	0.013947
GSE14386_UNTREATED_VS_IFNA_TREATED_ACT_PBMC_MS_PATIENT_UP	135	-0.54771	-1.69743	0	0.013951
GSE3203_HEALTHY_VS_INFLUENZA_INFECTED_LN_BCELL_DN	173	-0.50551	-1.69686	0	0.014013
GSE3920_UNTREATED_VS_IFNG_TREATED_FIBROBLAST_UP	162	-0.39322	-1.69616	0	0.014075
GSE2770_IL12_VS_IL4_TREATED_ACT_CD4_TCELL_2H_DN	155	-0.45307	-1.69582	0	0.014091
GSE32423_IL7_VS_IL7_IL4_NAIVE_CD8_TCELL_UP	174	-0.49226	-1.69568	0	0.014097
GSE1791_CTRL_VS_NEUROMEDINU_IN_T_CELL_LINE_12H_DN	147	-0.50343	-1.69551	0	0.014109
GSE43863_NAIVE_VS_LY6C_LOW_CXCR5NEG_CD4_EFF_TCELL_D6_LCMV_UP	144	-0.49904	-1.69511	0	0.014146

GSE17721_CTRL_VS_CPG_24H_BMDC_UP	183	-0.44174	-1.69492	0	0.014162
GSE22045_TREG_VS_TCONV_UP	160	-0.45433	-1.69448	0	0.014191
GSE23114_PERITONEAL_CAVITY_B1A_BCELL_VS_SPLEEN_BCELL_UP	162	-0.49825	-1.69349	0	0.014258
GSE46606_DAY1_VS_DAY3_CD40L_IL2_IL5_STIMULATED_IRF4MID_BCELL_UP	184	-0.50635	-1.69295	0	0.014311
GSE21379_TFH_VS_NON_TFH_SAP_KO_CD4_TCELL_DN	178	-0.38628	-1.69267	0	0.01432
GSE9960_HEALTHY_VS_SEPSIS_PBMC_DN	146	-0.46121	-1.69273	0	0.014322
GSE1925_CTRL_VS_3H_IFNG_STIM_MACROPHAGE_DN	187	-0.46918	-1.69247	0	0.014334
GSE16385_UNTREATED_VS_12H_ROSIGLITAZONE_IFNG_TNF_TREATED_MACROPHAGE_UP	183	-0.484	-1.69238	0	0.014334
GSE21927_SPLENIC_VS_TUMOR_MONOCYTES_FROM_C26GM_TUMOROUS_MICE_BALBC_UP	164	-0.44675	-1.69153	0	0.014383
GSE46606_UNSTIM_VS_CD40L_IL2_IL5_1DAY_STIMULATED_IRF4MID_SORTED_BCELL_DN	167	-0.47704	-1.69144	0	0.014387
GSE17721_CPG_VS_GARDIQUIMOD_12H_BMDC_UP	186	-0.458	-1.69131	0	0.014394
GSE22025_UNTREATED_VS_TGFB1_TREATED_CD4_TCELL_UP	186	-0.42391	-1.69081	0	0.014439

GSE17721_CTRL_VS_CPG_0.5H_BMDC_DN	186	-0.44764	-1.69003	0	0.014504
GSE19198_CTRL_VS_IL21_TREATED_TCELL_1H_UP	158	-0.50571	-1.69004	0	0.014508
GSE360_L_DONOVANI_VS_T_GONDII_DC_UP	191	-0.4794	-1.68943	0	0.01455
GSE22501_PERIPHERAL_BLOOD_VS_CORD_BLOOD_TREG_UP	187	-0.49632	-1.68863	0	0.014623
GSE29949_MICROGLIA_BRAIN_VS_CD8_NEG_DC_SPLEEN_UP	187	-0.44729	-1.6881	0	0.014675
GSE17721_CTRL_VS_GARDIQUIMOD_1H_BMDC_DN	187	-0.39655	-1.68768	0	0.014704
GSE8921_UNSTIM_0H_VS_TLR1_2_STIM_MONOCYTE_3H_UP	178	-0.42138	-1.68763	0	0.014706
GSE26928_CENTR_MEMORY_VS_CXCR5_POS_CD4_TCELL_DN	159	-0.41622	-1.68741	0	0.014723
GSE4142_NAIVE_VS_MEMORY_BCELL_UP	183	-0.48574	-1.68721	0	0.014734
GSE8835_CD4_VS_CD8_TCELL_DN	177	-0.50973	-1.68676	0	0.014784
GSE360_L_MAJOR_VS_M_TUBERCULOSIS_MAC_DN	180	-0.38427	-1.68663	0	0.014785
GSE26023_PHD3_KO_VS_WT_NEUTROPHIL_HYPOXIA_DN	186	-0.42946	-1.68666	0	0.014787

GSE15659_NAIVE_CD4_TCELL_VS_RESTING_TREG_DN	181	-0.42258	-1.68678	0	0.014788
GSE41978_KLRG1_HIGH_VS_LOW_EFFECTOR_CD8_TCELL_UP	188	-0.47471	-1.68514	0	0.014941
GSE12484_HEALTHY_VS_PERIDONTITIS_NEUTROPHILS_DN	141	-0.38175	-1.68426	0	0.015033
GSE31622_WT_VS_KLF3_KO_BCELL_UP	183	-0.50023	-1.684	0	0.015045
GSE22611_NOD2_VS_MUTANT_NOD2_TRANSDUCED_HEK293T_CELL_UP	178	-0.33908	-1.68398	0	0.015047
GSE17721_CTRL_VS_GARDIQUIMOD_12H_BMDC_DN	184	-0.37157	-1.68322	0	0.015122
GSE5099_DAY3_VS_DAY7_MCSF_TREATED_MACROPHAGE_DN	160	-0.45446	-1.68249	0	0.015188
GSE17974_IL4_AND_ANTI_IL12_VS_UNTREATED_4H_ACT_CD4_TCELL_DN	155	-0.53086	-1.68169	0	0.015255
GSE35435_RESTING_VS_IL4_TREATED_MACROPHAGE_DN	178	-0.42325	-1.68137	0	0.015278
GSE21546_WT_VS_ELK1_KO_DP_THYMOCYTES_UP	183	-0.38109	-1.68113	0	0.01529
GSE40274_FOXP3_VS_FOXP3_AND_HELIOS_TRANSDUCED_ACTIVATED_CD4_TCELL_UP	138	-0.51613	-1.68035	0	0.015385
GSE21063_3H_VS_16H_ANTI_IGM_STIM_NFATC1_KOBCELL_UP	178	-0.43496	-1.67924	0	0.015503

GSE24574_BCL6_LOW_TFH_VS_NAIVE_CD4_TCELL_UP	185	-0.39521	-1.67855	0	0.015557
GSE23321_EFFECTOR_MEMORY_VS_NAIVE_CD8_TCELL_UP	184	-0.47371	-1.67846	0	0.015567
GSE10240_IL17_VS_IL17_AND_IL22_STIM_PRIMARY_BRONCHIAL_EPITHELIAL_CELLS_DN	183	-0.44996	-1.67806	0	0.015593
GSE3982_CTRL_VS_LPS_48H_DC_UP	184	-0.40633	-1.67791	0	0.015608
GSE27786_CD4_TCELL_VS_NEUTROPHIL_DN	174	-0.39941	-1.67765	0	0.015619
GSE4142_PLASMA_CELL_VS_GC_BCELL_DN	172	-0.34901	-1.67771	0	0.01562
GSE27786_NKCELL_VS_ERYTHROBLAST_DN	178	-0.47633	-1.67726	0	0.015654
GSE19825_NAIVE_VS_IL2RALOW_DAY3_EFF_CD8_TCELL_UP	181	-0.55698	-1.67733	0	0.015655
GSE5542_IFNG_VS_IFNA_AND_IFNG_TREATED_EPITHELIAL_CELLS_24H_DN	182	-0.3987	-1.67716	0	0.015658
GSE15659_CD45RA_NEG_CD4_TCELL_VS_NONSUPPRESSIVE_TCELL_DN	182	-0.37145	-1.67687	0	0.015691
GSE15930_NAIVE_VS_48H_IN_VITRO_STIM_IFNAB_CD8_TCELL_UP	189	-0.41377	-1.67446	0	0.015922
GSE36476_YOUNG_VS_OLD_DONOR_MEMORY_CD4_TCELL_16H_TSST_ACT_UP	176	-0.47719	-1.6738	0	0.015999

GSE43863_NAIVE_VS_TH1_EFF_CD4_TCELL_D6_LCMV_DN	147	-0.51463	-1.67348	0	0.016038
GSE17721_PAM3CSK4_VS_CPG_16H_BMDC_UP	186	-0.36662	-1.67336	0	0.016046
GSE16385_MONOCYTE_VS_12H_ROSIGLITAZONE_TREATED_MACROPHAGE_UP	179	-0.40582	-1.67299	0	0.016077
GSE6875_TCONV_VS_FOXP3_KO_TREG_DN	182	-0.34817	-1.67245	0	0.016122
GSE41978_WT_VS_BIM_KO_KLRG1_LOW_EFFECTOR_CD8_TCELL_UP	182	-0.44359	-1.67111	0	0.016243
GSE45365_BCELL_VS_CD8_TCELL_UP	154	-0.52337	-1.67073	0	0.016274
GSE28737_FOLLICULAR_VS_MARGINAL_ZONE_BCELL_DN	187	-0.49061	-1.67041	0	0.016317
GSE17721_CTRL_VS_POLYIC_0.5H_BMDC_UP	176	-0.3679	-1.67011	0	0.016337
GSE20366_TREG_VS_TCONV_UP	178	-0.47167	-1.66996	0	0.016343
GSE19941_UNSTIM_VS_LPS_STIM_IL10_KO_NFKBP50_KO_MACROPHAGE_DN	181	-0.45666	-1.66968	0	0.016375
GSE15930_STIM_VS_STIM_AND_IFNAB_24H_CD8_T_CELL_UP	180	-0.37828	-1.66953	0	0.01638
GSE29949_CD8_NEG_DC_SPLEEN_VS_CD8_POS_DC_SPLEEN_UP	183	-0.49978	-1.66903	0	0.016436

GSE360_CTRL_VS_B_MALAYI_LOW_DOSE_DC_UP	189	-0.39726	-1.66858	0	0.01647
GSE16385_UNTREATED_VS_12H_ROSIGLITAZONE_TREATED_MACROPHAGE_UP	150	-0.54646	-1.66762	0	0.016551
GSE15750_WT_VS_TRAF6KO_DAY6_EFF_CD8_TCELL_UP	171	-0.37717	-1.66695	0	0.0166
GSE17721_CPG_VS_GARDIQUIMOD_2H_BMDC_UP	182	-0.42808	-1.66644	0	0.016652
GSE18791_CTRL_VS_NEWCASTLE_VIRUS_DC_1H_UP	145	-0.53533	-1.66617	0	0.016677
GSE21927_SPLENIC_C26GM_TUMOROUS_VS_4T1_TUMOR_MONOCYTES_UP	152	-0.4367	-1.66583	0	0.01669
GSE14413_UNSTIM_VS_IFNB_STIM_RAW264_CELLS_UP	121	-0.4932	-1.66597	0	0.016691
GSE17721_CTRL_VS_CPG_2H_BMDC_DN	184	-0.45438	-1.66493	0	0.016773
GSE32901_TH1_VS_TH17_NEG_CD4_TCELL_UP	110	-0.46954	-1.66441	0	0.016828
GSE22601_DOUBLE_POSITIVE_VS_CD4_SINGLE_POSITIVE_THYMOCYTE_UP	180	-0.44463	-1.66268	0	0.016991
GSE15659_RESTING_VS_ACTIVATED_TREG_UP	144	-0.48212	-1.66195	0	0.017071
GSE27786_LSK_VS_MONO_MAC_DN	177	-0.4076	-1.66155	0	0.017097

GSE29618_LAIV_VS_TIV_FLU_VACCINE_DAY7_MDC_DN	182	-0.44669	-1.66133	0	0.017108
GSE17721_CTRL_VS_PAM3CSK4_1H_BMDC_DN	188	-0.43552	-1.66136	0	0.017109
GSE29949_MICROGLIA_BRAIN_VS_CD8_POS_DC_SPLEEN_DN	189	-0.49932	-1.66108	0	0.017123
GSE3982_BCELL_VS_NKCELL_DN	185	-0.4984	-1.65913	0	0.017353
GSE22611_NOD2_TRANSD_VS_CTRL_TRANSD_HEK293_MDP_STIM_6H_DN	175	-0.41662	-1.65862	0	0.017394
GSE22935_UNSTIM_VS_48H_MBOVIS_BCG_STIM_MYD88_KO_MACROPHAGE_DN	177	-0.47461	-1.65851	0	0.0174
GSE3994_WT_VS_PAC1_KO_ACTIVATED_MAST_CELL_UP	182	-0.47422	-1.65801	0	0.017449
GSE3982_BCELL_VS_EFF_MEMORY_CD4_TCELL_DN	180	-0.48542	-1.65789	0	0.017467
GSE21063_CTRL_VS_ANTI_IGM_STIM_BCELL_8H_DN	181	-0.47855	-1.65587	0	0.017688
GSE14413_UNSTIM_VS_IFNB_STIM_L929_CELLS_UP	105	-0.48166	-1.65535	0	0.017736
GSE1925_CTRL_VS_IFNG_PRIMED_MACROPHAGE_3H_IFNG_STIM_DN	117	-0.51255	-1.65496	0	0.017794
GSE2128_CTRL_VS_MIMETOPE_NEGATIVE_SELECTION_DP_THYMOCYTE_C57BL6_UP	169	-0.47197	-1.65447	0	0.017839

GSE45365_CD8A_DC_VS_CD11B_DC_IFNAR_KO_MCMV_INFECTION_DN	146	-0.52287	-1.65395	0	0.017882
GSE2585_AIRE_KO_VS_WT_CD80_LOW_MTEC_UP	173	-0.46623	-1.65355	0	0.017912
GSE2585_AIRE_KO_VS_WT_CD80_HIGH_MTEC_DN	179	-0.34525	-1.65359	0	0.017913
GSE45365_NK_CELL_VS_CD8A_DC_MCMV_INFECTION_UP	162	-0.49125	-1.65317	0	0.017946
GSE21360_PRIMARY_VS_SECONDARY_MEMORY_CD8_TCELL_DN	183	-0.39441	-1.65131	0	0.018123
GSE36476_YOUNG_VS_OLD_DONOR_MEMORY_CD4_TCELL_40H_TSST_ACT_UP	184	-0.53151	-1.65122	0	0.018133
GSE19512_NAUTRAL_VS_INDUCED_TREG_UP	188	-0.50883	-1.65116	0	0.018135
GSE360_L_MAJOR_VS_B_MALAYI_LOW_DOSE_MAC_UP	178	-0.41652	-1.64649	0	0.018663
GSE2706_LPS_VS_R848_AND_LPS_2H_STIM_DC_UP	153	-0.4833	-1.64553	0	0.018787
GSE39152_SPLEEN_CD103_NEG_VS_BRAIN_CD103_POS_MEMORY_CD8_TCELL_DN	189	-0.44701	-1.6437	0	0.018969
GSE34156_UNTREATED_VS_24H_NOD2_LIGAND_TREATED_MONOCYTE_UP	155	-0.45765	-1.64284	0	0.019022
GSE339_CD4POS_VS_CD4CD8DN_DC_IN_CULTURE_UP	186	-0.42364	-1.64271	0	0.019036

GSE13485_CTRL_VS_DAY3_YF17D_VACCINE_PBMC_UP	149	-0.50002	-1.64267	0	0.01904
GSE27786_NKCELL_VS_NEUTROPHIL_DN	177	-0.42423	-1.64225	0	0.019082
GSE20366_EX_VIVO_VS_DEC205_CONVERSION_NAIVE_CD4_TCELL_DN	172	-0.44549	-1.64168	0	0.019144
GSE40685_NAIVE_CD4_TCELL_VS_FOXP3_KO_TREG_PRECURSOR_DN	176	-0.46232	-1.64157	0	0.019145
GSE37301_HEMATOPOIETIC_STEM_CELL_VS_COMMON_LYMPHOID_PROGENITOR_UP	172	-0.43238	-1.64162	0	0.019147
GSE360_HIGH_VS_LOW_DOSE_B_MALAYI_DC_DN	183	-0.43454	-1.64146	0	0.019154
GSE360_L_MAJOR_VS_T_GONDII_DC_UP	182	-0.49807	-1.64116	0	0.019175
GSE29615_CTRL_VS_DAY7_LAIV_FLU_VACCINE_PBMC_UP	155	-0.51179	-1.64017	0	0.019287
GSE8921_UNSTIM_0H_VS_TLR1_2_STIM_MONOCYTE_3H_DN	178	-0.51576	-1.63834	0	0.019523
GSE40274_FOXP3_VS_FOXP3_AND_GATA1_TRANSDUCED_ACTIVATED_CD4_TCELL_UP	189	-0.49017	-1.63511	0	0.01995
GSE7460_CTRL_VS_FOXP3_OVEREXPR_TCONV_1_UP	178	-0.43084	-1.63421	0	0.020044
GSE2706_LPS_VS_R848_AND_LPS_2H_STIM_DC_DN	143	-0.52518	-1.63305	0	0.020191

GSE15659_TREG_VS_TCONV_DN	157	-0.44934	-1.63308	0	0.020193
GSE23114_WT_VS_SLE2C1_MOUSE_PERITONEAL_CAVITY_B1A_BCELL_DN	158	-0.50798	-1.63006	0	0.02053
GSE11961_PLASMA_CELL_DAY7_VS_MEMORY_BCELL_DAY40_DN	177	-0.48064	-1.62885	0	0.020652
GSE14769_UNSTIM_VS_20MIN_LPS_BMDM_UP	176	-0.5132	-1.62705	0	0.020909
GSE21546_UNSTIM_VS_ANTI_CD3_STIM_SAP1A_KO_DP_THYMOCYTES_DN	130	-0.53454	-1.62587	0	0.02105
GSE46242_TH1_VS_ANERGIC_TH1_CD4_TCELL_DN	164	-0.44591	-1.62533	0	0.021116
GSE18804_BRAIN_VS_COLON_TUMORAL_MACROPHAGE_UP	166	-0.4404	-1.62446	0	0.021242
GSE28737_WT_VS_BCL6_HET_FOLLICULAR_BCELL_UP	181	-0.41029	-1.62343	0	0.021368
GSE15930_NAIVE_VS_72H_IN_VITRO_STIM_CD8_TCELL_UP	189	-0.3647	-1.62177	0	0.02158
GSE20152_HTNFA_OVERXPRESS_ANKLE_VS_CTRL_SPHK1_KO_ANKLE_DN	180	-0.40603	-1.62119	0	0.02164
GSE17721_POLYIC_VS_PAM3CSK4_0.5H_BMDC_UP	186	-0.42418	-1.61975	0	0.021778
GSE27670_CTRL_VS_BLIMP1_TRANSDUCED_GC_BCELL_UP	190	-0.48328	-1.61903	0	0.021851

GSE12003_4D_VS_8D_CULTURE_MIR223_KO_BM_PROGENITOR_UP	144	-0.48897	-1.61879	0	0.021868
GSE21033_CTRL_VS_POLYIC_STIM_DC_1H_UP	174	-0.45006	-1.61688	0	0.022115
GSE5455_HEALTHY_VS_TUMOR_BEARING_MOUSE_SPLEEN_MONOCYTE_24H_INCUBATION_UP	180	-0.3948	-1.61635	0	0.02219
GSE2770_IL12_VS_TGFB_AND_IL12_TREATED_ACT_CD4_TCELL_6H_UP	175	-0.46099	-1.61573	0	0.022265
GSE17721_CTRL_VS_PAM3CSK4_2H_BMDC_DN	186	-0.43001	-1.61466	0	0.022385
GSE26928_EFF_MEMORY_VS_CXCR5_POS_CD4_TCELL_UP	172	-0.43224	-1.61306	0	0.022578
GSE17974_IL4_AND_ANTI_IL12_VS_UNTREATED_1H_ACT_CD4_TCELL_DN	149	-0.49147	-1.61125	0	0.022831
GSE22611_UNSTIM_VS_6H_MDP_STIM_NOD2_TRANSDUCED_HEK293T_CELL_UP	178	-0.40489	-1.61111	0	0.022837
GSE10094_LCMV_VS_LISTERIA_IND_EFF_CD4_TCELL_DN	183	-0.48415	-1.61076	0	0.022872
GSE21927_HEALTHY_VS_TUMOROUS_BALBC_MOUSE_MONOCYTE_DN	146	-0.48519	-1.61023	0	0.022931
GSE10147_IL3_AND_HIVP17_VS_IL3_AND_CPG_STIM_PDC_UP	141	-0.48497	-1.60977	0	0.022965
GSE13411_NAIVE_VS_MEMORY_BCELL_UP	173	-0.50448	-1.606	0	0.023457

GSE25123_CTRL_VS_IL4_AND_ROSIGLITAZONE_STIM_MACROPHAGE_DN	166	-0.40974	-1.60535	0	0.023564
GSE22601_DOUBLE_NEGATIVE_VS_DOUBLE_POSITIVE_THYMOCYTE_UP	163	-0.40529	-1.60505	0	0.023606
GSE2405_0H_VS_6H_A_PHAGOCYTOPHILUM_STIM_NEUTROPHIL_DN	159	-0.44909	-1.60227	0	0.024051
GSE15324_ELF4_KO_VS_WT_ACTIVATED_CD8_TCELL_DN	184	-0.39936	-1.60186	0	0.024111
GSE17721_CTRL_VS_POLYIC_0.5H_BMDC_DN	187	-0.41381	-1.60128	0	0.024166
GSE13762_CTRL_VS_125_VITAMIND_DAY5_DC_UP	128	-0.50794	-1.60136	0	0.024175
GSE37532_VISCERAL_ADIPOSE_TISSUE_VS_LN_DERIVED_TCONV_CD4_TCELL_DN	96	-0.55783	-1.60141	0	0.024177
GSE15324_ELF4_KO_VS_WT_NAIVE_CD8_TCELL_UP	180	-0.42476	-1.60087	0	0.024206
GSE41176_WT_VS_TAK1_KO_ANTI_IGM_STIM_BCELL_1H_UP	183	-0.42937	-1.60037	0	0.024257
GSE45365_HEALTHY_VS_MCMV_INFECTION_CD8_TCELL_IFNAR_KO_UP	154	-0.46982	-1.59986	0	0.024288
GSE21927_EL4_VS_MCA203_TUMOR_MONOCYTES_DN	156	-0.39648	-1.59971	0	0.024313
GSE29949_CD8_NEG_DC_SPLEEN_VS_DC_BRAIN_UP	174	-0.48078	-1.59886	0	0.024444

GSE33162_HDAC3_KO_VS_HDAC3_KO_4H_LPS_STIM_MACROPHAGE_DN	193	-0.47856	-1.59763	0	0.024593
GSE6681_DELETED_FOXP3_VS_WT_TREG_UP	174	-0.48091	-1.59463	0	0.024974
GSE13887_HEALTHY_VS_LUPUS_RESTING_CD4_TCELL_UP	109	-0.45628	-1.59393	0	0.025072
GSE37532_VISCERAL_ADIPOSE_TISSUE_VS_LN_DERIVED_PPARG_KO_TCONV_CD4_TCELL_DN	91	-0.49769	-1.59275	0	0.025194
GSE7568_CTRL_VS_24H_TGFB_TREATED_MACROPHAGES_WITH_IL4_AND_DEXAMETHASONE_DN	184	-0.49656	-1.59265	0	0.025201
GSE17721_PAM3CSK4_VS_GADIQUIMOD_1H_BMDC_UP	188	-0.43768	-1.59137	0	0.025371
GSE360_L_MAJOR_VS_M_TUBERCULOSIS_MAC_UP	182	-0.44407	-1.58339	0	0.026613
GSE41176_UNSTIM_VS_ANTI_IGM_STIM_BCELL_24H_UP	180	-0.48694	-1.58281	0	0.026699
GSE40274_EOS_VS_FOXP3_AND_EOS_TRANSDUCED_ACTIVATED_CD4_TCELL_DN	112	-0.51144	-1.57916	0	0.027274
GSE22886_TH1_VS_TH2_48H_ACT_UP	186	-0.44853	-1.57832	0	0.027405
GSE28783_ANTI_MIR33_VS_CTRL_ATHEROSCLEROSIS_MACROPHAGE_DN	184	-0.44001	-1.5743	0	0.027949
GSE36078_UNTREATED_VS_AD5_INF_MOUSE_LUNG_DC_UP	187	-0.48186	-1.57096	0	0.028504

GSE36476_YOUNG_VS_OLD_DONOR_MEMORY_CD4_TCELL_DN	177	-0.49279	-1.56981	0	0.02867
GSE29618_PRE_VS_DAY7_POST_LAIV_FLU_VACCINE_BCELL_DN	177	-0.39709	-1.56869	0	0.02884
GSE37532_WT_VS_PPARG_KO_LN_TREG_DN	112	-0.53808	-1.56546	0	0.029387
GSE29615_CTRL_VS_LAIV_FLU_VACCINE_PBMC_DN	137	-0.44846	-1.55506	0	0.03108
GSE2706_R848_VS_LPS_8H_STIM_DC_UP	142	-0.46638	-1.54837	0	0.032279
GSE17721_PAM3CSK4_VS_GADIQUIMOD_2H_BMDC_UP	188	-0.42619	-1.548	0	0.032353
GSE22886_IL2_VS_IL15_STIM_NKCELL_UP	184	-0.48897	-1.54729	0	0.032482
GSE4748_LPS_VS_LPS_AND_CYANOBACTERIUM_LPSLIKE_STIM_DC_3H_DN	149	-0.44997	-1.53876	0	0.034095
GSE13306_TREG_RA_VS_TCONV_RA_UP	179	-0.36418	-1.53628	0	0.034578
GSE18791_UNSTIM_VS_NEWCATSLE_VIRUS_DC_2H_UP	144	-0.49775	-1.52097	0	0.037599
GSE11961_FOLLICULAR_BCELL_VS_MARGINAL_ZONE_BCELL_DN	183	-0.40196	-1.50964	0	0.039976
GSE16385_IFNG_TNF_VS_ROSIGLITAZONE_STIM_MACROPHAGE_DN	146	-0.46735	-1.50193	0	0.041647

GSE5542_IFNA_VS_IFNA_AND_IFNG_TREATED_EPITHELIAL_CELLS_6H_UP	175	-0.38214	-1.69234	0.001845	0.014334
GSE17974_0H_VS_2H_IN_VITRO_ACT_CD4_TCELL_UP	169	-0.45468	-1.87314	0.001866	0.005121
GSE27859_MACROPHAGE_VS_DC_DN	178	-0.43314	-1.64589	0.001876	0.018746
GSE3982_EOSINOPHIL_VS_EFF_MEMORY_CD4_TCELL_UP	181	-0.41757	-1.86851	0.00189	0.005221
GSE25846_IL10_POS_VS_NEG_CD8_TCELL_DAY7_POST_CORONAVIRUS_BRAIN_DN	177	-0.3335	-1.79075	0.00189	0.008049
GSE360_CTRL_VS_L_MAJOR_MAC_DN	187	-0.36916	-1.58562	0.001894	0.026281
GSE11961_MEMORY_BCELL_DAY40_VS_GERMINAL_CENTER_BCELL_DAY40_DN	176	-0.37166	-1.64423	0.001898	0.018929
GSE8621_UNSTIM_VS_LPS_STIM_MACROPHAGE_DN	173	-0.43456	-2.12816	0.001901	0.001846
GSE46242_TH1_VS_ANERGIC_TH1_CD4_TCELL_WITH_EGR2_DELETED_DN	171	-0.42664	-1.80407	0.001901	0.007544
GSE9960_GRAM_POS_VS_GRAM_NEG_AND_POS_SEPSIS_PBMC_DN	188	-0.41259	-1.62159	0.001901	0.021601
GSE7509_UNSTIM_VS_TNFA_IL1B_IL6_PGE_STIM_DC_DN	86	-0.49761	-1.47222	0.001901	0.049028
GSE15330_LYMPHOID_MULTIPOTENT_VS_MEGAKARYOCYTE_ERYTHROID_PROGENITOR_IKAROS_KO_UP	154	-0.52669	-1.90803	0.001908	0.004277

GSE44732_UNSTIM_VS_IL27_STIM_IMATURE_DC_DN	181	-0.49144	-1.87229	0.001908	0.005151
GSE37534_GW1929_VS_ROSIGLITAZONE_TREATED_CD4_TCELL_PPARG1_FOXP3_TRANSDUCED_DN	153	-0.30907	-1.79518	0.001908	0.007858
GSE40274_CTRL_VS_XBP1_TRANSDUCED_ACTIVATED_CD4_TCELL_UP	159	-0.56496	-1.97622	0.001912	0.003329
GSE37416_CTRL_VS_3H_F_TULARENSIS_LVS_NEUTROPHIL_UP	165	-0.42617	-1.93903	0.001912	0.003725
GSE2405_0H_VS_9H_A_PHAGOCYTOPHILUM_STIM_NEUTROPHIL_UP	185	-0.39869	-1.70296	0.001912	0.01351
GSE11961_FOLLICULAR_BCELL_VS_GERMINAL_CENTER_BCELL_DAY40_DN	179	-0.46872	-1.99802	0.001916	0.003005
GSE25087_FETAL_VS_ADULT_TCONV_DN	163	-0.48339	-1.8471	0.001916	0.005865
GSE3982_BASOPHIL_VS_NKCELL_UP	175	-0.38863	-1.73037	0.001916	0.011496
GSE45365_CTRL_VS_MCMV_INFECTION_NK_CELL_UP	187	-0.46538	-1.64134	0.001916	0.019157
GSE43955_10H_VS_60H_ACT_CD4_TCELL_DN	181	-0.42824	-1.73236	0.001919	0.011352
GSE46606_IRF4_KO_VS_WT_CD40L_IL2_IL5_3DAY_STIMULATED_BCELL_DN	161	-0.46789	-2.02278	0.001923	0.002851
GSE17974_CTRL_VS_ACT_IL4_AND_ANTI_IL12_2H_CD4_TCELL_UP	169	-0.44409	-1.86232	0.001923	0.005383

GSE25146_UNSTIM_VS_HELIOBACTER_PYLORI_LPS_STIM_AGS_CELL_DN	147	-0.47714	-2.01549	0.001927	0.002916
GSE24292_WT_VS_PPARG_KO_MACROPHAGE_UP	184	-0.39041	-1.82525	0.001927	0.006595
GSE45365_WT_VS_IFNAR_KO_CD11B_DC_UP	163	-0.45709	-1.82431	0.001927	0.006631
GSE24972_WT_VS_IRF8_KO_SPLEEN_FOLLICULAR_BCELL_UP	179	-0.45598	-1.73389	0.001927	0.011233
GSE20198_UNTREATED_VS_IL12_IL18_TREATED_ACT_CD4_TCELL_UP	185	-0.40749	-1.72024	0.001927	0.012241
GSE24142_EARLY_THYMIC_PROGENITOR_VS_DN2_THYMOCYTE_UP	185	-0.58183	-1.92528	0.001931	0.003978
GSE23114_WT_VS_SLE2C1_MOUSE_SPLEEN_B1A_BCELL_DN	185	-0.46149	-1.7368	0.001931	0.011049
GSE45365_WT_VS_IFNAR_KO_BCELL_UP	166	-0.39875	-1.64358	0.001931	0.01896
GSE360_L_DONOVANI_VS_B_MALAYI_HIGH_DOSE_DC_DN	185	-0.45565	-2.22131	0.001934	0.001649
GSE24210_IL35_TREATED_VS_UNTREATED_TCONV_CD4_TCELL_DN	187	-0.46687	-1.72039	0.001934	0.012237
GSE42088_UNINF_VS_LEISHMANIA_INF_DC_24H_UP	183	-0.49857	-2.01527	0.001938	0.00291
GSE22886_DAY0_VS_DAY1_MONOCYTE_IN_CULTURE_UP	187	-0.60409	-1.94804	0.001938	0.00362

GSE34515_CD16_NEG_MONOCYTE_VS_DC_UP	181	-0.53119	-2.08207	0.001942	0.002442
GSE43955_TGFB_IL6_VS_TGFB_IL6_IL23_TH17_ACT_CD4_TCELL_60H_UP	188	-0.45453	-2.04393	0.001942	0.00268
GSE1460_DP_THYMOCYTE_VS_NAIVE_CD4_TCELL_ADULT_BLOOD_DN	183	-0.45415	-2.00902	0.001942	0.002961
GSE21360_NAIVE_VS_SECONDARY_MEMORY_CD8_TCELL_DN	160	-0.55806	-1.99137	0.001942	0.003101
GSE40274_CTRL_VS_FOXP3_TRANSDUCED_ACTIVATED_CD4_TCELL_DN	185	-0.5257	-1.97303	0.001942	0.00334
GSE18281_CORTICAL_THYMOCYTE_VS_WHOLE_CORTEX_THYMUS_DN	179	-0.45472	-1.93616	0.001942	0.003762
GSE20366_EX_VIVO_VS_DEC205_CONVERSION_NAIVE_CD4_TCELL_UP	183	-0.5174	-1.82797	0.001942	0.006513
GSE12003_4D_VS_8D_CULTURE_BM_PROGENITOR_DN	128	-0.45803	-1.76644	0.001942	0.009287
GSE2405_HEAT_KILLED_VS_LIVE_A_PHAGOCYTOPHILUM_STIM_NEUTROPHIL_24H_DN	186	-0.42296	-1.60422	0.001942	0.023716
GSE40274_XBP1_VS_FOXP3_AND_XBP1_TRANSDUCED_ACTIVATED_CD4_TCELL_UP	184	-0.50611	-1.97671	0.001946	0.003327
GSE40068_BCL6_POS_VS_NEG_CXCR5_POS_TFH_DN	172	-0.4367	-1.93673	0.001946	0.003762
GSE24634_TEFF_VS_TCONV_DAY10_IN_CULTURE_DN	190	-0.55715	-1.90659	0.001946	0.004304

GSE19198_6H_VS_24H_IL21_TREATED_TCELL_UP	177	-0.54077	-1.87246	0.001946	0.005146
GSE13493_DP_VS_CD4INTCD8POS_THYMOCYTE_UP	177	-0.35557	-1.74765	0.001946	0.010312
GSE360_CTRL_VS_L_DONOVANI_MAC_DN	189	-0.3405	-1.71617	0.001946	0.012508
GSE21927_SPLEEN_VS_C26GM_TUMOR_MONOCYTE_BALBC_UP	149	-0.50209	-1.63729	0.001946	0.019667
GSE38304_MYC_NEG_VS_POS_GC_BCELL_DN	183	-0.4795	-1.99946	0.001949	0.003
GSE11961_MEMORY_BCELL_DAY7_VS_GERMINAL_CENTER_BCELL_DAY40_UP	185	-0.4192	-1.82612	0.001949	0.006569
GSE43863_NAIVE_VS_LY6C_INT_CXCR5POS_CD4_EFF_TCELL_D6_LCMV_UP	165	-0.41761	-1.78708	0.001949	0.008215
GSE30083_SP2_VS_SP4_THYMOCYTE_UP	182	-0.4882	-1.73605	0.001949	0.011099
GSE9037_WT_VS_IRAK4_KO_BMDM_DN	179	-0.42522	-1.56959	0.001949	0.02869
GSE2706_R848_VS_LPS_2H_STIM_DC_UP	152	-0.44415	-1.523	0.001949	0.037193
GSE3720_LPS_VS_PMA_STIM_VD1_GAMMADELTA_TCELL_DN	161	-0.48967	-2.04218	0.001953	0.002696
GSE17721_CTRL_VS_PAM3CSK4_0.5H_BMDC_UP	187	-0.37628	-1.95731	0.001953	0.003431

GSE36888_STAT5_AB_KNOCKIN_VS_WT_TCELL_IL2_TREATED_2H_UP	175	-0.58486	-1.96397	0.001953	0.00346
GSE41867_NAIVE_VS_DAY8_LCMV_CLONE13_EFFECTOR_CD8_TCELL_UP	183	-0.4164	-1.91767	0.001953	0.004122
GSE17974_0H_VS_1H_IN_VITRO_ACT_CD4_TCELL_DN	173	-0.45696	-1.9068	0.001953	0.004304
GSE9509_LPS_VS_LPS_AND_IL10_STIM_IL10_KO_MACROPHAGE_10MIN_DN	183	-0.27074	-1.89981	0.001953	0.004384
GSE17974_0H_VS_1H_IN_VITRO_ACT_CD4_TCELL_UP	156	-0.48274	-1.88847	0.001953	0.004646
GSE11924_TH1_VS_TH2_CD4_TCELL_UP	179	-0.40085	-1.76223	0.001953	0.00949
GSE12392_IFNAR_KO_VS_IFNB_KO_CD8_NEG_SPLEEN_DC_DN	190	-0.44429	-1.76214	0.001953	0.009491
GSE29618_LAIV_VS_TIV_FLU_VACCINE_DAY7_BCELL_UP	177	-0.4443	-1.49931	0.001953	0.042266
GSE5503_LIVER_DC_VS_MLN_DC_ACTIVATED_ALLOGENIC_TCELL_DN	178	-0.37878	-2.11518	0.001957	0.001974
GSE45881_CXCR6HI_VS_CXCR1LO_COLONIC_LAMINA_PROPRIA_UP	176	-0.46344	-1.93518	0.001957	0.003769
GSE13547_CTRL_VS_ANTI_IGM_STIM_BCELL_12H_DN	161	-0.42142	-1.90447	0.001957	0.004343
GSE17580_TREG_VS_TEFF_S_MANSONI_INF_UP	184	-0.50147	-1.8896	0.001957	0.004617

GSE20366_EX_VIVO_VS_DEC205_CONVERSION_UP	182	-0.41226	-1.79464	0.001957	0.007891
GSE17721_LPS_VS_CPG_2H_BMDC_UP	193	-0.4236	-1.76388	0.001957	0.009398
GSE44649_WT_VS_MIR155_KO_NAIVE_CD8_TCELL_UP	182	-0.44455	-1.67669	0.001957	0.015702
GSE13522_WT_VS_IFNG_KO_SKIN_UP	124	-0.5304	-1.56088	0.001957	0.030083
GSE13173_UNTREATED_VS_IL12_TREATED_ACT_CD8_TCELL_UP	117	-0.50406	-1.54135	0.001957	0.033579
GSE46606_UNSTIM_VS_CD40L_IL2_IL5_3DAY_STIMULATED_IRF4MID_SORTED_BCELL_UP	181	-0.32228	-2.0477	0.001961	0.002649
GSE19198_1H_VS_24H_IL21_TREATED_TCELL_DN	178	-0.60679	-1.95546	0.001961	0.003477
GSE9960_GRAM_NEG_VS_GRAM_POS_SEPSIS_PBMC_DN	182	-0.40084	-1.92225	0.001961	0.004013
GSE12392_CD8A_POS_VS_NEG_SPLEEN_DC_UP	189	-0.38236	-1.80497	0.001961	0.007495
GSE3982_MAST_CELL_VS_BCELL_UP	179	-0.48752	-1.79912	0.001961	0.0077
GSE22589_HIV_VS_HIV_AND_SIV_INFECTED_DC_UP	183	-0.45376	-1.7544	0.001961	0.009924
GSE42088_UNINF_VS_LEISHMANIA_INF_DC_4H_DN	186	-0.50521	-1.97455	0.001965	0.003337

GSE22886_NAIVE_CD8_TCELL_VS_NKCELL_DN	184	-0.46556	-1.95583	0.001965	0.003475
GSE17721_CTRL_VS_CPG_6H_BMDC_UP	185	-0.34704	-1.93519	0.001965	0.003772
GSE39820_CTRL_VS_TGFBETA3_IL6_IL23A_CD4_TCELL_DN	174	-0.38509	-1.91778	0.001965	0.004121
GSE43863_LY6C_INT_CXCR5POS_VS_LY6C_LOW_CXCR5NEG_EFFECTOR_CD4_TCELL_DN	188	-0.48897	-1.82237	0.001965	0.006701
GSE21063_WT_VS_NFATC1_KO_16H_ANTI_IGM_STIM_BCELL_UP	177	-0.34782	-1.81951	0.001965	0.006823
GSE35685_CD34POS_CD38NEG_VS_CD34POS_CD10NEG_CD62LPOS_BONE_MARROW_DN	192	-0.40708	-1.69845	0.001965	0.013888
GSE9988_ANTI_TREM1_AND_LPS_VS_CTRL_TREATED_MONOCYTES_DN	179	-0.45342	-2.13552	0.001969	0.001889
GSE3982_BASOPHIL_VS_EFF_MEMORY_CD4_TCELL_UP	186	-0.46649	-2.08666	0.001969	0.0024
GSE21360_PRIMARY_VS_QUATERNARY_MEMORY_CD8_TCELL_DN	184	-0.44361	-2.01955	0.001969	0.002854
GSE46606_UNSTIM_VS_CD40L_IL2_IL5_1DAY_STIMULATED_IRF4_KO_BCELL_UP	184	-0.49619	-1.97158	0.001969	0.003386
GSE6259_FLT3L_INDUCED_33D1_POS_DC_VS_CD4_TCELL_UP	162	-0.63391	-1.9628	0.001969	0.003453
GSE17301_CTRL_VS_48H_ACD3_ACD28_STIM_CD8_TCELL_DN	187	-0.52897	-1.90977	0.001969	0.004256

GSE11057_CD4_EFF_MEM_VS_PBMC_DN	183	-0.61321	-1.90362	0.001969	0.004343
GSE360_L_DONOVANI_VS_M_TUBERCULOSIS_DC_DN	187	-0.4915	-1.82853	0.001969	0.006484
GSE26488_CTRL_VS_PEPTIDE_INJECTION_OT2_THYMOCYTE_UP	152	-0.51165	-1.77969	0.001969	0.008643
GSE7460_TREG_VS_TCONV_ACT_WITH_TGFB_DN	180	-0.43591	-1.76067	0.001969	0.009585
GSE29618_PRE_VS_DAY7_POST_LAIV_FLU_VACCINE_MONOCYTE_DN	176	-0.36028	-1.75598	0.001969	0.009825
GSE43700_UNTREATED_VS_IL10_TREATED_PBMC_UP	181	-0.41014	-1.65484	0.001969	0.017799
GSE4142_PLASMA_CELL_VS_GC_BCELL_UP	182	-0.38966	-1.65406	0.001969	0.017873
GSE29615_CTRL_VS_DAY7_LAIV_FLU_VACCINE_PBMC_DN	132	-0.50568	-1.65334	0.001969	0.017932
GSE24574_BCL6_HIGH_TFH_VS_TCONV_CD4_TCELL_UP	188	-0.4162	-1.6009	0.001969	0.02421
GSE19401_NAIVE_VS_IMMUNIZED_MOUSE_PLN_FOLLICULAR_DC_DN	186	-0.47023	-2.16942	0.001972	0.001648
GSE40274_IRF4_VS_FOXP3_AND_IRF4_TRANSDUCED_ACTIVATED_CD4_TCELL_UP	167	-0.33914	-2.15444	0.001972	0.001849
GSE12392_CD8A_POS_VS_NEG_SPLEEN_IFNB_KO_DC_DN	183	-0.39851	-1.91159	0.001972	0.004229

GSE23502_BM_VS_COLON_TUMOR_MYELOID_DERIVED_SUPPRESSOR_CELL_DN	179	-0.54861	-1.88764	0.001972	0.004665
GSE17721_CTRL_VS_CPG_8H_BMDC_UP	177	-0.33553	-1.86871	0.001972	0.005219
GSE7852_LN_VS_FAT_TCONV_DN	187	-0.51042	-1.86028	0.001972	0.005451
GSE46606_IRF4HIGH_VS_WT_CD40L_IL2_IL5_DAY3_STIMULATED_BCELL_DN	185	-0.54124	-1.8561	0.001972	0.00559
GSE5589_LPS_VS_LPS_AND_IL10_STIM_MACROPHAGE_180MIN_DN	182	-0.45017	-1.83925	0.001972	0.006069
GSE40273_XBP1_KO_VS_WT_TREG_DN	185	-0.50816	-1.83883	0.001972	0.006077
GSE13547_CTRL_VS_ANTI_IGM_STIM_ZFX_KO_BCELL_12H_DN	147	-0.54593	-1.8258	0.001972	0.006566
GSE24972_MARGINAL_ZONE_BCELL_VS_FOLLICULAR_BCELL_UP	175	-0.42768	-1.65632	0.001972	0.017648
GSE21033_CTRL_VS_POLYIC_STIM_DC_1H_DN	171	-0.40946	-1.62983	0.001972	0.020553
GSE369_SOCS3_KO_VS_IFNG_KO_LIVER_DN	173	-0.38856	-1.58555	0.001972	0.026274
GSE15659_RESTING_TREG_VS_NONSUPPRESSIVE_TCELL_UP	151	-0.43729	-1.57753	0.001972	0.027476
GSE17721_LPS_VS_PAM3CSK4_0.5H_BMDC_UP	184	-0.33707	-1.52305	0.001972	0.03721

GSE18148_CBFB_KO_VS_WT_TREG_UP	170	-0.42813	-1.52141	0.001972	0.037524
GSE43955_1H_VS_60H_ACT_CD4_TCELL_WITH_TGFB_IL6_UP	187	-0.43694	-2.06335	0.001976	0.002502
GSE9988_LOW_LPS_VS_ANTI_TREM1_AND_LPS_MONOCYTE_UP	174	-0.59464	-1.9637	0.001976	0.003452
GSE2405_0H_VS_12H_A_PHAGOCYTOPHILUM_STIM_NEUTROPHIL_UP	183	-0.60084	-1.96298	0.001976	0.003453
GSE26030_TH1_VS_TH17_DAY5_POST_POLARIZATION_UP	183	-0.56529	-1.94958	0.001976	0.003603
GSE25088_ROSIGLITAZONE_VS_IL4_AND_ROSIGLITAZONE_STIM_STAT6_KO_MACROPHAGE_DAY10_UP	170	-0.49226	-1.94544	0.001976	0.00365
GSE31082_DP_VS_CD8_SP_THYMOCYTE_DN	182	-0.40973	-1.90021	0.001976	0.004378
GSE32986_CURDLAN_LOWDOSE_VS_GMCSF_AND_CURDLAN_LOWDOSE_STIM_DC_UP	172	-0.40514	-1.86083	0.001976	0.005434
GSE360_CTRL_VS_L_MAJOR_DC_DN	189	-0.5113	-1.86051	0.001976	0.005444
GSE37301_MULTIPOTENT_PROGENITOR_VS_PRO_BCELL_DN	176	-0.43998	-1.85569	0.001976	0.005604
GSE14350_IL2RB_KO_VS_WT_TREG_UP	182	-0.42946	-1.8522	0.001976	0.005709
GSE7460_TCONV_VS_TREG_THYMUS_DN	189	-0.53115	-1.82305	0.001976	0.006676

GSE2706_UNSTIM_VS_8H_R848_DC_UP	174	-0.48895	-1.80344	0.001976	0.007545
GSE12392_WT_VS_IFNB_KO_CD8A_POS_SPLEEN_DC_DN	184	-0.49072	-1.71181	0.001976	0.012794
GSE21927_SPLEEN_VS_TUMOR_MONOCYTE_BALBC_UP	145	-0.45522	-1.6961	0.001976	0.014074
GSE34217_MIR17_92_OVEREXPRESS_VS_WT_ACT_CD8_TCELL_DN	125	-0.40775	-1.6667	0.001976	0.016624
GSE7460_CTRL_VS_FOXP3_OVEREXPR_TCONV_UP	180	-0.44522	-1.56923	0.001976	0.028756
GSE39382_IL3_VS_IL3_IL33_TREATED_MAST_CELL_UP	183	-0.42742	-2.17389	0.00198	0.001759
GSE5589_UNSTIM_VS_180MIN_LPS_AND_IL10_STIM_MACROPHAGE_DN	180	-0.49257	-1.98098	0.00198	0.003273
GSE24671_CTRL_VS_SENDAI_VIRUS_INFECTED_MOUSE_SPLENOCYTES_DN	177	-0.42217	-1.97867	0.00198	0.003317
GSE9988_LPS_VS_LPS_AND_ANTI_TREM1_MONOCYTE_UP	182	-0.54122	-1.97578	0.00198	0.00333
GSE1925_3H_VS_24H_IFNG_STIM_IFNG_PRIMED_MACROPHAGE_UP	187	-0.396	-1.94291	0.00198	0.00369
GOLDRATH_NAIVE_VS_MEMORY_CD8_TCELL_UP	189	-0.41662	-1.91745	0.00198	0.004124
GSE4590_SMALL_VS_LARGE_PRE_BCELL_UP	172	-0.60147	-1.88796	0.00198	0.004666

GSE7460_WT_VS_FOXP3_HET_ACT_WITH_TGFB_TCONV_DN	181	-0.42915	-1.86131	0.00198	0.005415
GSE33424_CD161_HIGH_VS_INT_CD8_TCELL_UP	186	-0.44132	-1.84219	0.00198	0.005979
GSE17721_LPS_VS_GARDIQUIMOD_4H_BMDC_UP	188	-0.41051	-1.78423	0.00198	0.008392
GSE29618_LAIV_VS_TIV_FLU_VACCINE_DAY7_MONOCYTE_UP	179	-0.39097	-1.77302	0.00198	0.008932
GSE2405_HEAT_KILLED_VS_LIVE_A_PHAGOCYTOPHILUM_STIM_NEUTROPHIL_24H_UP	162	-0.4591	-1.74281	0.00198	0.010616
GSE41978_ID2_KO_VS_BIM_KO_KLRG1_LOW_EFFECTOR_CD8_TCELL_DN	187	-0.42357	-1.73988	0.00198	0.010795
GSE45365_NK_CELL_VS_CD8_TCELL_UP	154	-0.36674	-1.72245	0.00198	0.012096
GSE6259_DEC205_POS_DC_VS_CD8_TCELL_DN	148	-0.48053	-1.68793	0.00198	0.014697
GSE36392_EOSINOPHIL_VS_MAC_IL25_TREATED_LUNG_UP	177	-0.50101	-1.57987	0.00198	0.027147
GSE17721_POLYIC_VS_CPG_4H_BMDC_DN	180	-0.47971	-2.07559	0.001984	0.002459
GSE42724_MEMORY_BCELL_VS_PLASMABLAST_UP	186	-0.45066	-2.01693	0.001984	0.002876
GSE2128_CTRL_VS_MIMETOPE_NEGATIVE_SELECTION_DP_THYMOCYTE_NOD_UP	180	-0.54727	-1.92883	0.001984	0.003905

GSE14000_4H_VS_16H_LPS_DC_UP	170	-0.42995	-1.92119	0.001984	0.004035
GSE3982_NEUTROPHIL_VS_CENT_MEMORY_CD4_TCELL_DN	177	-0.3449	-1.91218	0.001984	0.004213
GSE39820_CTRL_VS_IL1B_IL6_IL23A_CD4_TCELL_UP	187	-0.49934	-1.91116	0.001984	0.004243
GSE6259_33D1_POS_VS_DEC205_POS_SPLENIC_DC_UP	167	-0.48097	-1.84202	0.001984	0.005984
GSE17721_POLYIC_VS_GARDIQUIMOD_2H_BMDC_DN	178	-0.39723	-1.84048	0.001984	0.006038
GSE7548_DAY7_VS_DAY28_PCC_IMMUNIZATION_CD4_TCELL_DN	179	-0.45784	-1.83018	0.001984	0.006419
GSE17721_0.5H_VS_4H_GARDIQUIMOD_BMDC_DN	179	-0.36341	-1.80937	0.001984	0.00728
GSE411_UNSTIM_VS_100MIN_IL6_STIM_MACROPHAGE_UP	183	-0.43125	-1.80074	0.001984	0.007639
GSE2826_XID_VS_BTK_KO_BCELL_UP	185	-0.40804	-1.76413	0.001984	0.009392
GSE18281_SUBCAPSULAR_CORTICAL_REGION_VS_WHOLE_CORTEX_THYMUS_DN	184	-0.46932	-1.73554	0.001984	0.011136
GSE13306_RA_VS_UNTREATED_TREG_DN	181	-0.48217	-1.71063	0.001984	0.012877
GSE33292_DN3_THYMOCYTE_VS_TCF1_KO_TCELL_LYMPHOMA_DN	139	-0.5026	-1.69409	0.001984	0.014224

GSE43955_TH0_VS_TGFB_IL6_TH17_ACT_CD4_TCELL_20H_DN	184	-0.36306	-1.68775	0.001984	0.014707
GSE17974_0H_VS_72H_IN_VITRO_ACT_CD4_TCELL_UP	162	-0.43084	-1.68311	0.001984	0.015128
GSE2585_CD80_HIGH_VS_LOW_AIRE_KO_MTEC_UP	179	-0.51034	-1.68148	0.001984	0.015271
GSE25502_WT_VS_KLF13_KO_THYMIC_MEMORY_LIKE_CD8_TCELL_DN	146	-0.51753	-1.59445	0.001984	0.025007
GSE19198_6H_VS_24H_IL21_TREATED_TCELL_DN	171	-0.55316	-2.06704	0.001988	0.002501
GSE18893_TCONV_VS_TREG_2H_TNF_STIM_DN	168	-0.5352	-2.06517	0.001988	0.002529
GSE3720_UNSTIM_VS_LPS_STIM_VD2_GAMMADELTA_TCELL_UP	154	-0.47017	-1.96082	0.001988	0.003436
GSE39022_LN_VS_SPLEEN_DC_DN	171	-0.34604	-1.93023	0.001988	0.003858
GSE21360_TERTIARY_VS_QUATERNARY_MEMORY_CD8_TCELL_DN	166	-0.56681	-1.91522	0.001988	0.004165
GSE29618_PDC_VS_MDC_DAY7_FLU_VACCINE_DN	189	-0.64037	-1.9115	0.001988	0.004232
GSE6674_UNSTIM_VS_PL2_3_STIM_BCELL_DN	158	-0.4274	-1.88905	0.001988	0.004639
GSE7852_TREG_VS_TCONV_UP	184	-0.4759	-1.86356	0.001988	0.005339

GSE8921_UNSTIM_VS_TLR1_2_STIM_MONOCYTE_12H_DN	182	-0.39968	-1.83715	0.001988	0.006141
GSE14308_TH2_VS_TH1_DN	182	-0.41481	-1.81787	0.001988	0.006906
GSE23308_WT_VS_MINERALCORTICOID_REC_KO_MACROPHAGE_UP	180	-0.28351	-1.81144	0.001988	0.007162
GSE22527_ANTI_CD3_INVIVO_VS_UNTREATED_MOUSE_TREG_UP	176	-0.48379	-1.79207	0.001988	0.007991
GSE27241_WT_VS_RORGT_KO_TH17_POLARIZED_CD4_TCELL_TREATED_WITH_DIGOXIN_DN	171	-0.44685	-1.74731	0.001988	0.01034
GSE3982_DC_VS_NEUTROPHIL_LPS_STIM_DN	186	-0.48945	-1.73523	0.001988	0.011154
GSE25123_IL4_VS_IL4_AND_ROSIGLITAZONE_STIM_PPARG_KO_MACROPHAGE_DAY10_UP	140	-0.40687	-1.7147	0.001988	0.012612
GSE6259_FLT3L_INDUCED_DEC205_POS_DC_VS_BCELL_UP	145	-0.45625	-1.71253	0.001988	0.012751
GSE369_SOCS3_KO_VS_IFNG_KO_LIVER_UP	169	-0.45869	-1.67957	0.001988	0.01547
GSE5589_IL6_KO_VS_IL10_KO_LPS_AND_IL6_STIM_MACROPHAGE_45MIN_DN	176	-0.34404	-1.66172	0.001988	0.017083
GSE2128_C57BL6_VS_NOD_THYMOCYTE_MIMETOPE_NEGATIVE_SELECTION_UP	183	-0.45785	-1.59255	0.001988	0.025208
GSE360_CTRL_VS_M_TUBERCULOSIS_MAC_UP	183	-0.38772	-2.01398	0.001992	0.002897

GSE41176_UNSTIM_VS_ANTI_IGM_STIM_TAK1_KO_BCELL_1H_UP	171	-0.40925	-2.01183	0.001992	0.002916
GSE11818_WT_VS_DICER_KO_TREG_DN	146	-0.38392	-1.93542	0.001992	0.003764
GSE1791_CTRL_VS_NEUROMEDINU_IN_T_CELL_LINE_3H_UP	152	-0.54979	-1.92275	0.001992	0.004008
GSE5589_WT_VS_IL6_KO_LPS_STIM_MACROPHAGE_45MIN_DN	182	-0.45338	-1.86512	0.001992	0.00532
GSE11057_CD4_CENT_MEM_VS_PBMC_DN	180	-0.60097	-1.86315	0.001992	0.005362
GSE43955_TH0_VS_TGFB_IL6_IL23_TH17_ACT_CD4_TCELL_60H_UP	190	-0.41198	-1.7975	0.001992	0.007757
GSE23925_DARK_ZONE_VS_NAIVE_BCELL_DN	186	-0.43043	-1.7787	0.001992	0.008664
GSE6875_TCONV_VS_TREG_UP	181	-0.37667	-1.63459	0.001992	0.020003
GSE17721_CTRL_VS_POLYIC_2H_BMDC_UP	179	-0.33722	-1.61367	0.001992	0.022512
GSE21670_TGFB_VS_IL6_TREATED_CD4_TCELL_UP	177	-0.518	-1.60868	0.001992	0.023119
GSE43955_1H_VS_42H_ACT_CD4_TCELL_DN	189	-0.43725	-1.60728	0.001992	0.023291
GSE8835_HEALTHY_VS_CLL_CD4_TCELL_DN	172	-0.4662	-1.58585	0.001992	0.026253

GSE21670_TGFB_VS_IL6_TREATED_CD4_TCELL_DN	183	-0.348	-1.53711	0.001992	0.034446
GSE24292_WT_VS_PPARG_KO_MACROPHAGE_DN	184	-0.47693	-2.027	0.001996	0.002842
GSE17721_0.5H_VS_8H_CPG_BMDC_DN	181	-0.45161	-2.00511	0.001996	0.003
GSE36888_UNTREATED_VS_IL2_TREATED_TCELL_17H_UP	184	-0.5699	-1.96302	0.001996	0.00346
GSE33424_CD161_INT_VS_NEG_CD8_TCELL_UP	182	-0.58386	-1.95172	0.001996	0.003565
GSE19198_CTRL_VS_IL21_TREATED_TCELL_6H_DN	177	-0.53828	-1.94856	0.001996	0.003621
GSE40068_CXCR5NEG_BCL6NEG_CD4_TCELL_VS_CXCR5POS_BCL6NEG_TFH_DN	185	-0.53863	-1.94655	0.001996	0.003635
GSE10273_LOW_IL7_VS_HIGH_IL7_AND_IRF4_IN_IRF4_8_NULL_PRE_BCELL_DN	177	-0.38192	-1.91654	0.001996	0.004133
GSE3982_DC_VS_TH2_UP	187	-0.54905	-1.85417	0.001996	0.005633
GSE360_CTRL_VS_L_MAJOR_MAC_UP	182	-0.34584	-1.8346	0.001996	0.006248
GSE8678_IL7R_LOW_VS_HIGH_EFF_CD8_TCELL_UP	181	-0.42709	-1.82712	0.001996	0.00655
GSE24142_DN2_VS_DN3_THYMOCYTE_UP	186	-0.48768	-1.81857	0.001996	0.006882

GSE20727_CTRL_VS_ROS_INHIBITOR_TREATED_DC_DN	178	-0.42735	-1.7971	0.001996	0.007771
GSE28737_BCL6_HET_VS_BCL6_KO_FOLLICULAR_BCELL_UP	177	-0.35399	-1.71355	0.001996	0.012663
GSE26488_HDAC7_KO_VS_VP16_TRANSGENIC_HDAC7_KO_DOUBLE_POSITIVE_THYMOCYTE_DN	189	-0.47135	-1.71287	0.001996	0.012718
GSE17974_IL4_AND_ANTI_IL12_VS_UNTREATED_72H_ACT_CD4_TCELL_UP	153	-0.48514	-1.70972	0.001996	0.012952
GSE6269_HEALTHY_VS_E_COLI_INF_PBMC_DN	150	-0.48302	-1.7035	0.001996	0.013487
GSE2770_UNTREATED_VS_TGFB_AND_IL12_TREATED_ACT_CD4_TCELL_6H_DN	182	-0.45842	-1.64962	0.001996	0.018289
GSE360_L_MAJOR_VS_T_GONDII_DC_DN	181	-0.4732	-1.64493	0.001996	0.018842
GSE41867_DAY6_VS_DAY15_LCMV_CLONE13_EFFECTOR_CD8_TCELL_UP	151	-0.54202	-1.59516	0.001996	0.024928
GSE14769_UNSTIM_VS_40MIN_LPS_BMDM_UP	179	-0.41148	-1.55958	0.001996	0.030316
GSE43955_TGFB_IL6_VS_TGFB_IL6_IL23_TH17_ACT_CD4_TCELL_52H_DN	192	-0.46598	-1.95939	0.002	0.003441
GSE6259_33D1_POS_DC_VS_CD4_TCELL_DN	166	-0.60765	-1.93958	0.002	0.003721
GSE1740_UNSTIM_VS_IFNA_STIMULATED_MCSF_DERIVED_MACROPHAGE_DN	161	-0.53497	-1.93599	0.002	0.003763

GSE46242_CTRL_VS_EGR2_DELETED_ANERGIC_TH1_CD4_TCELL_UP	176	-0.45245	-1.85058	0.002	0.005758
GSE36476_CTRL_VS_TSST_ACT_72H_MEMORY_CD4_TCELL_YOUNG_UP	179	-0.48732	-1.84436	0.002	0.005929
GSE5099_UNSTIM_VS_MCSF_TREATED_MONOCYTE_DAY3_UP	181	-0.38259	-1.8175	0.002	0.006924
GSE25088_CTRL_VS_ROSIGLITAZONE_STIM_STAT6_KO_MACROPHAGE_DN	170	-0.32951	-1.81404	0.002	0.007062
GSE12392_WT_VS_IFNB_KO_CD8A_NEG_SPLEEN_DC_UP	184	-0.31946	-1.80406	0.002	0.007541
GSE2585_CTEC_VS_MTEC_THYMUS_UP	174	-0.44668	-1.80346	0.002	0.007547
GSE46606_IRF4HIGH_VS_WT_CD40L_IL2_IL5_DAY1_STIMULATED_BCELL_DN	189	-0.4055	-1.8025	0.002	0.007592
GSE29618_PRE_VS_DAY7_POST_LAIV_FLU_VACCINE_MDC_DN	168	-0.38593	-1.79403	0.002	0.007923
GSE36095_WT_VS_HDAC9_KO_TREG_UP	175	-0.42441	-1.75217	0.002	0.010023
GSE28783_ANTI_MIR33_VS_UNTREATED_ATHEROSCLEROSIS_MACROPHAGE_DN	186	-0.47994	-1.73463	0.002	0.011183
GSE3982_CTRL_VS_PMA_STIM_EOSINOPHIL_UP	173	-0.33578	-1.67836	0.002	0.015576
GSE2770_IL4_ACT_VS_ACT_CD4_TCELL_48H_UP	179	-0.30716	-1.67614	0.002	0.015765

GSE18281_PERIMEDULLARY_CORTICAL_REGION_VS_WHOLE_CORTEX_THYMUS_DN	176	-0.4832	-1.6437	0.002	0.018964
GSE41176_UNSTIM_VS_ANTI_IGM_STIM_TAK1_KO_BCELL_3H_UP	170	-0.45839	-1.61821	0.002	0.021939
GSE11961_MEMORY_BCELL_DAY40_VS_GERMINAL_CENTER_BCELL_DAY40_UP	182	-0.36677	-1.55694	0.002	0.030771
GSE45365_NK_CELL_VS_BCELL_MCMV_INFECTION_DN	182	-0.5094	-2.04581	0.002004	0.002672
GSE11864_CSF1_PAM3CYS_VS_CSF1_IFNG_PAM3CYS_IN_MAC_DN	173	-0.58107	-1.96992	0.002004	0.003367
GSE31082_CD4_VS_CD8_SP_THYMOCYTE_UP	179	-0.47189	-1.94203	0.002004	0.003711
KAECH_NAIVE_VS_DAY15_EFF_CD8_TCELL_DN	187	-0.56936	-1.93276	0.002004	0.003801
GSE22886_DAY0_VS_DAY7_MONOCYTE_IN_CULTURE_UP	178	-0.57627	-1.92947	0.002004	0.003878
GSE9037_CTRL_VS_LPS_4H_STIM_IRAK4_KO_BMDM_DN	180	-0.45811	-1.91038	0.002004	0.004253
GSE17721_PAM3CSK4_VS_CPG_2H_BMDC_DN	186	-0.46291	-1.85093	0.002004	0.00575
GSE7460_WT_VS_FOXP3_HET_ACT_WITH_TGFB_TCONV_UP	175	-0.44859	-1.8073	0.002004	0.007365
GSE27896_HDAC6_KO_VS_WT_TREG_UP	161	-0.42029	-1.78985	0.002004	0.008092

GSE2770_IL12_VS_TGFB_AND_IL12_TREATED_ACT_CD4_TCELL_2H_UP	129	-0.47824	-1.78489	0.002004	0.008343
GSE18281_MEDULLARY_THYMOCYTE_VS_WHOLE_MEDULLA_THYMUS_UP	167	-0.41852	-1.77861	0.002004	0.008666
GSE2770_UNTREATED_VS_IL12_TREATED_ACT_CD4_TCELL_6H_UP	180	-0.38853	-1.76841	0.002004	0.009182
GSE16385_IL4_VS_ROSIGLITAZONE_STIM_MACROPHAGE_DN	161	-0.44559	-1.76625	0.002004	0.009293
GSE46606_UNSTIM_VS_CD40L_IL2_IL5_DAY1_STIMULATED_BCELL_UP	171	-0.41181	-1.73436	0.002004	0.011197
GSE41867_DAY6_VS_DAY15_LCMV_ARMSTRONG_EFFECTOR_CD8_TCELL_UP	169	-0.54368	-1.72217	0.002004	0.012104
GSE5099_MONOCYTE_VS_CLASSICAL_M1_MACROPHAGE_DN	172	-0.31223	-1.71176	0.002004	0.012785
GSE37301_PRO_BCELL_VS_GRANULOCYTE_MONOCYTE_PROGENITOR_UP	138	-0.48346	-1.69632	0.002004	0.014062
GSE41867_DAY15_EFFECTOR_VS_DAY30_MEMORY_CD8_TCELL_LCMV_ARMSTRONG_DN	160	-0.43613	-1.67681	0.002004	0.015693
GSE13411_IGM_VS_SWITCHED_MEMORY_BCELL_DN	175	-0.39235	-2.01088	0.002008	0.002935
GSE7852_THYMUS_VS_FAT_TCONV_DN	184	-0.48826	-1.89883	0.002008	0.004407
GSE15330_HSC_VS_LYMPHOID_PRIMED_MULTIPOTENT_PROGENITOR_UP	190	-0.45395	-1.88241	0.002008	0.00482

GSE339_CD8POS_VS_CD4CD8DN_DC_IN_CULTURE_DN	186	-0.3808	-1.86491	0.002008	0.005318
GSE1432_CTRL_VS_IFNG_24H_MICROGLIA_UP	191	-0.42227	-1.85543	0.002008	0.005612
GSE3982_DC_VS_BCELL_UP	189	-0.51899	-1.83074	0.002008	0.006397
GSE5503_LIVER_DC_VS_PLN_DC_ACTIVATED_ALLOGENIC_TCELL_UP	176	-0.41188	-1.81896	0.002008	0.006868
GSE9946_MATURE_STIMULATORY_VS_PROSTAGLANDINE2_TREATED_MATURE_DC_UP	120	-0.45291	-1.77978	0.002008	0.008639
GSE46606_UNSTIM_VS_CD40L_IL2_IL5_1DAY_STIMULATED_IRF4HIGH_SORTED_BCELL_UP	173	-0.4558	-1.66814	0.002008	0.016505
GSE37301_HEMATOPOIETIC_STEM_CELL_VS_MULTIPOTENT_PROGENITOR_UP	190	-0.41798	-1.66266	0.002008	0.016985
GSE23925_LIGHT_ZONE_VS_DARK_ZONE_BCELL_UP	180	-0.4283	-1.63528	0.002008	0.019936
GSE15215_CD2_POS_VS_NEG_PDC_UP	185	-0.41907	-1.61963	0.002008	0.021784
GSE3982_CTRL_VS_LPS_1H_NEUTROPHIL_DN	184	-0.398	-1.60126	0.002008	0.024162
GSE6092_IFNG_VS_IFNG_AND_B_BURGDORFERI_INF_ENDOTHELIAL_CELL_DN	162	-0.54131	-2.07989	0.002012	0.002468
GSE34156_UNTREATED_VS_6H_NOD2_AND_TLR1_TLR2_LIGAND_TREATED_MONOCYTE_DN	143	-0.61716	-2.07484	0.002012	0.002469

GSE43863_TFH_VS_LY6C_LOW_CXCR5NEG_EFFECTOR_CD4_TCELL_UP	183	-0.50654	-1.97151	0.002012	0.003385
GSE21033_1H_VS_24H_POLYIC_STIM_DC_DN	156	-0.43912	-1.91338	0.002012	0.004199
GSE17721_CTRL_VS_LPS_12H_BMDC_DN	182	-0.43131	-1.91234	0.002012	0.004209
GSE4590_PRE_BCELL_VS_LARGE_PRE_BCELL_UP	152	-0.52497	-1.8809	0.002012	0.004878
GSE14415_INDUCED_TREG_VS_FOXP3_KO_INDUCED_TREG_UP	165	-0.44945	-1.87959	0.002012	0.004909
GSE15330_HSC_VS_GRANULOCYTE_MONOCYTE_PROGENITOR_IKAROS_KO_UP	185	-0.37365	-1.79881	0.002012	0.007716
GSE26928_NAIVE_VS_CXCR5_POS_CD4_TCELL_DN	173	-0.37959	-1.73426	0.002012	0.011195
GSE29949_MICROGLIA_BRAIN_VS_MONOCYTE_BONE_MARROW_UP	187	-0.48561	-1.67729	0.002012	0.015654
GSE6566_STRONG_VS_WEAK_DC_STIMULATED_CD4_TCELL_UP	158	-0.44926	-1.55138	0.002012	0.031749
GSE41867_DAY8_VS_DAY15_LCMV_ARMSTRONG_EFFECTOR_CD8_TCELL_DN	143	-0.48781	-1.51602	0.002012	0.038657
GSE22886_NAIVE_VS_IGM_MEMORY_BCELL_DN	185	-0.51022	-2.05959	0.002016	0.002602
GSE9960_HEALTHY_VS_GRAM_POS_SEPSIS_PBMC_DN	172	-0.57115	-1.92309	0.002016	0.004007

GSE46242_CTRL_VS_EGR2_DELETED_ANERGIC_TH1_CD4_TCELL_DN	174	-0.55073	-1.91732	0.002016	0.004122
GSE21033_3H_VS_24H_POLYIC_STIM_DC_UP	153	-0.50777	-1.82977	0.002016	0.006442
GSE3982_NEUTROPHIL_VS_TH1_UP	174	-0.45099	-1.82664	0.002016	0.006563
GSE36078_WT_VS_IL1R_KO_LUNG_DC_AFTER_AD5_INF_UP	185	-0.5663	-1.82248	0.002016	0.006697
GSE40277_EOS_AND_LEF1_TRANSDUCED_VS_CTRL_CD4_TCELL_DN	185	-0.38028	-1.72991	0.002016	0.011515
GSE42021_CD24HI_VS_CD24INT_TCONV_THYMUS_UP	187	-0.35016	-1.67513	0.002016	0.015851
GSE22601_IMMATURE_CD4_SINGLE_POSITIVE_VS_DOUBLE_POSITIVE_THYMOCYTE_UP	156	-0.55304	-1.6571	0.002016	0.017551
GSE22611_NOD2_TRANSD_VS_CTRL_TRANSD_HEK293_MDP_STIM_6H_UP	182	-0.41585	-1.6069	0.002016	0.023346
GSE46606_UNSTIM_VS_CD40L_IL2_IL5_3DAY_STIMULATED_IRF4_KO_BCELL_UP	150	-0.52432	-1.59431	0.002016	0.025026
GSE8678_IL7R_LOW_VS_HIGH_EFF_CD8_TCELL_DN	176	-0.46942	-2.17031	0.00202	0.001675
GSE360_T_GONDII_VS_B_MALAYI_HIGH_DOSE_DC_DN	189	-0.54974	-2.11729	0.00202	0.001943
GSE27786_CD4_TCELL_VS_NKCELL_UP	174	-0.47324	-2.02319	0.00202	0.002867

GSE39110_UNTREATED_VS_IL2_TREATED_CD8_TCELL_DAY6_POST_IMMUNIZATION_UP	183	-0.60741	-1.89933	0.00202	0.004393
GSE3982_DC_VS_BASOPHIL_UP	178	-0.45749	-1.86438	0.00202	0.005328
GSE360_DC_VS_MAC_M_TUBERCULOSIS_UP	186	-0.52416	-1.8575	0.00202	0.005538
GSE3565_DUSP1_VS_WT_SPLENOCYTES_UP	155	-0.60096	-1.84779	0.00202	0.005843
GSE17721_LPS_VS_POLYIC_4H_BMDC_UP	192	-0.4168	-1.83287	0.00202	0.006299
GSE37301_HEMATOPOIETIC_STEM_CELL_VS_PRO_BCELL_DN	151	-0.3663	-1.82284	0.00202	0.006682
GSE26912_TUMORICIDAL_VS_CTRL_MACROPHAGE_DN	183	-0.48806	-1.7823	0.00202	0.008483
GSE14415_INDUCED_TREG_VS_FAILED_INDUCED_TREG_DN	173	-0.5279	-1.77304	0.00202	0.008934
GSE29617_DAY3_VS_DAY7_TIV_FLU_VACCINE_PBMC_2008_UP	169	-0.48994	-1.76233	0.00202	0.009492
GSE6269_STAPH_AUREUS_VS_STREP_PNEUMO_INF_PBMC_UP	156	-0.38108	-1.68584	0.00202	0.014856
GSE13762_CTRL_VS_125_VITAMIND_DAY12_DC_UP	142	-0.45291	-1.66334	0.00202	0.016928
GSE19772_CTRL_VS_HCMV_INF_MONOCYTES_AND_PI3K_INHIBITION_DN	188	-0.41286	-1.64944	0.00202	0.018302

GSE20366_CD103_KLRG1_DP_VS_DN_TREG_UP	183	-0.38989	-1.60004	0.00202	0.024291
GSE29618_PRE_VS_DAY7_POST_LAIV_FLU_VACCINE_PDC_UP	187	-0.46245	-1.56112	0.00202	0.030061
GSE16450_CTRL_VS_IFNA_12H_STIM_MATURE_NEURON_CELL_LINE_UP	178	-0.44663	-1.53247	0.00202	0.035295
GSE9946_MATURE_STIMULATORY_VS_LISTERIA_INF_MATURE_DC_DN	124	-0.48513	-1.53023	0.00202	0.03575
GSE17721_PAM3CSK4_VS_CPG_24H_BMDC_DN	179	-0.41495	-2.02878	0.002024	0.002852
GSE11057_NAIVE_VS_MEMORY_CD4_TCELL_DN	182	-0.53696	-1.98523	0.002024	0.003229
GSE17186_MEMORY_VS_CD21LOW_TRANSITIONAL_BCELL_UP	180	-0.40426	-1.9816	0.002024	0.003282
GSE40685_TREG_VS_FOXP3_KO_TREG_PRECURSOR_DN	187	-0.63509	-1.97873	0.002024	0.003309
GSE16522_MEMORY_VS_NAIVE_ANTI_CD3CD28_STIM_CD8_TCELL_DN	189	-0.54998	-1.96862	0.002024	0.00338
GSE14350_IL2RB_KO_VS_WT_TEFF_UP	179	-0.48268	-1.88216	0.002024	0.004827
GSE42021_CD24HI_VS_CD24INT_TCONV_THYMUS_DN	185	-0.51528	-1.8555	0.002024	0.005609
GSE43955_10H_VS_60H_ACT_CD4_TCELL_WITH_TGFB_IL6_UP	188	-0.35244	-1.83376	0.002024	0.006267

GSE360_DC_VS_MAC_B_MALAYI_LOW_DOSE_UP	182	-0.34626	-1.82925	0.002024	0.006452
GSE3982_DC_VS_NEUTROPHIL_UP	181	-0.33269	-1.82624	0.002024	0.006577
GSE36009_UNSTIM_VS_LPS_STIM_NLRP10_KO_DC_UP	178	-0.36355	-1.81518	0.002024	0.006995
GSE24081_CONTROLLER_VS_PROGRESSOR_HIV_SPECIFIC_CD8_TCELL_DN	179	-0.46539	-1.74291	0.002024	0.01062
GSE17721_CTRL_VS_CPG_6H_BMDC_DN	187	-0.39031	-1.72891	0.002024	0.011575
GSE18148_CBFB_KO_VS_WT_TREG_DN	181	-0.45452	-1.60971	0.002024	0.022968
GSE13522_CTRL_VS_T_CRUZI_Y_STRAIN_INF_SKIN_129_MOUSE_DN	180	-0.4046	-1.5957	0.002024	0.024851
GSE3982_EFF_MEMORY_VS_CENT_MEMORY_CD4_TCELL_DN	169	-0.39895	-1.59058	0.002024	0.02548
GSE7596_AKT_TRANSD_VS_CTRL_CD4_TCONV_WITH_TGFB_DN	111	-0.48689	-1.57235	0.002024	0.028277
GSE6674_UNSTIM_VS_ANTI_IGM_STIM_BCELL_DN	120	-0.51877	-1.49919	0.002024	0.042286
GSE16385_IFNG_TNF_VS_IL4_STIM_MACROPHAGE_DN	153	-0.42411	-1.49117	0.002024	0.044139
GSE17974_IL4_AND_ANTI_IL12_VS_UNTREATED_0.5H_ACT_CD4_TCELL_DN	141	-0.4605	-1.46405	0.002024	0.051015

GSE22886_CTRL_VS_LPS_24H_DC_UP	188	-0.46876	-2.14335	0.002028	0.001945
GSE19888_ADENOSINE_A3R_ACT_VS_TCELL_MEMBRANES_ACT_IN_MAST_CELL_UP	181	-0.59021	-1.93892	0.002028	0.003727
GSE40685_TREG_VS_FOXP3_KO_TREG_PRECURSOR_UP	179	-0.58035	-1.89367	0.002028	0.004542
GSE6674_CPG_VS_PL2_3_STIM_BCELL_UP	168	-0.43057	-1.86924	0.002028	0.005204
GSE13887_RESTING_VS_NO_TREATED_CD4_TCELL_DN	103	-0.50943	-1.82374	0.002028	0.006651
GSE339_EX_VIVO_VS_IN_CULTURE_CD8POS_DC_UP	178	-0.33234	-1.81252	0.002028	0.007125
GSE17721_POLYIC_VS_GARDIQUIMOD_4H_BMDC_DN	187	-0.33466	-1.77572	0.002028	0.0088
GSE41867_DAY6_VS_DAY8_LCMV_ARMSTRONG_EFFECTOR_CD8_TCELL_UP	184	-0.33717	-1.76916	0.002028	0.009149
GSE36888_UNTREATED_VS_IL2_TREATED_STAT5_AB_KNOCKIN_TCELL_17H_DN	187	-0.40261	-1.7506	0.002028	0.010135
GSE33374_CD8_ALPHAALPHA_VS_ALPHABETA_CD161_HIGH_TCELL_UP	189	-0.47591	-1.71362	0.002028	0.012671
GSE17721_LPS_VS_POLYIC_0.5H_BMDC_UP	180	-0.3666	-1.7087	0.002028	0.013028
GSE43957_UNTREATED_VS_NACL_TREATED_ANTI_CD3_CD28_STIM_CD4_TCELL_UP	191	-0.41615	-1.60023	0.002028	0.024271

GSE24634_NAIVE_CD4_TCELL_VS_DAY10_IL4_CONV_TREG_UP	177	-0.3936	-1.50663	0.002028	0.040629
GSE9946_IMMATURE_VS_MATURE_STIMULATORY_DC_DN	123	-0.48278	-1.50447	0.002028	0.041074
GSE3982_MAC_VS_TH1_UP	180	-0.50048	-1.90409	0.002033	0.004344
GSE17721_POLYIC_VS_PAM3CSK4_12H_BMDC_UP	183	-0.4172	-1.90145	0.002033	0.004357
GSE11057_NAIVE_VS_EFF_MEMORY_CD4_TCELL_DN	177	-0.55712	-1.90042	0.002033	0.004372
GSE17974_IL4_AND_ANTI_IL12_VS_UNTREATED_24H_ACT_CD4_TCELL_DN	159	-0.62559	-1.87687	0.002033	0.005013
GSE17721_0.5H_VS_24H_PAM3CSK4_BMDC_DN	186	-0.341	-1.86649	0.002033	0.005273
GSE17721_ALL_VS_24H_PAM3CSK4_BMDC_DN	184	-0.43374	-1.86502	0.002033	0.005315
GSE22589_HEALTHY_VS_HIV_INFECTED_DC_DN	181	-0.53547	-1.85238	0.002033	0.005703
GSE7218_IGM_VS_IGG_SIGNAL_THGOUGH_ANTIGEN_BCELL_DN	162	-0.65306	-1.81679	0.002033	0.006943
GSE2128_C57BL6_VS_NOD_CD4CD8_DP_THYMOCYTE_DN	178	-0.34783	-1.80305	0.002033	0.007566
GSE46606_IRF4_KO_VS_WT_CD40L_IL2_IL5_3DAY_STIMULATED_BCELL_UP	175	-0.48037	-1.78569	0.002033	0.008316

GSE40274_FOXP3_VS_FOXP3_AND_GATA1_TRANSDUCED_ACTIVATED_CD4_TCELL_DN	181	-0.42604	-1.78113	0.002033	0.008556
GSE17721_0.5H_VS_8H_GARDIQUIMOD_BMDC_DN	184	-0.34814	-1.76228	0.002033	0.009492
GSE17974_CTRL_VS_ACT_IL4_AND_ANTI_IL12_6H_CD4_TCELL_DN	175	-0.42728	-1.74247	0.002033	0.010631
GSE19888_ADENOSINE_A3R_INH_VS_ACT_WITH_INHIBITOR_PRETREATMENT_IN_MAST_CELL_DN	178	-0.50657	-1.67202	0.002033	0.016162
GSE19941_IL10_KO_VS_IL10_KO_AND_NFKBP50_KO_UNSTIM_MACROPHAGE_DN	185	-0.43888	-1.65608	0.002033	0.01767
GSE13547_CTRL_VS_ANTI_IGM_STIM_BCELL_2H_DN	151	-0.45961	-1.65125	0.002033	0.018129
GSE37301_HEMATOPOIETIC_STEM_CELL_VS_CD4_TCELL_DN	121	-0.50374	-1.62986	0.002033	0.020557
GSE13522_WT_VS_IFNG_KO_SKIN_DN	116	-0.47975	-1.55121	0.002033	0.03175
GSE37416_12H_VS_48H_F_TULARENSIS_LVS_NEUTROPHIL_UP	183	-0.48087	-2.06404	0.002037	0.002513
GSE3337_CTRL_VS_16H_IFNG_IN_CD8POS_DC_DN	179	-0.4644	-2.03413	0.002037	0.002761
GSE34515_CD16_NEG_VS_POS_MONOCYTE_DN	169	-0.43023	-1.96986	0.002037	0.00336
GSE15330_HSC_VS_GRANULOCYTE_MONOCYTE_PROGENITOR_IKAROS_KO_DN	182	-0.40251	-1.94819	0.002037	0.003619

GSE15930_STIM_VS_STIM_AND_IFNAB_48H_CD8_T_CELL_DN	19<br>4	-<br>0.484<br>1	-<br>1.906<br>2	0.0020<br>37	0.0043<br>05
GSE24634_NAIVE_CD4_TCELL_VS_DAY7_IL4_CONV_TREG_UP	17<br>7	-<br>0.413<br>56	-<br>1.896<br>24	0.0020<br>37	0.0044<br>81
GSE17721_POLYIC_VS_CPG_4H_BMDC_UP	17<br>7	-<br>0.357<br>82	-<br>1.839<br>57	0.0020<br>37	0.0060<br>66
GSE17721_4_VS_24H_GARDIQUIMOD_BMDC_UP	17<br>8	-<br>0.431<br>75	-<br>1.808<br>57	0.0020<br>37	0.0073<br>22
GSE21546_WT_VS_SAP1A_KO_AND_ELK1_KO_ANTI_CD3_STIM_DP_THYMOCYTES_UP	17<br>5	-<br>0.475<br>65	-<br>1.786<br>34	0.0020<br>37	0.0082<br>61
GSE26488_WT_VS_HDAC7_KO_DOUBLE_POSITIVE_THYMOCYTE_DN	16<br>0	-<br>0.503<br>81	-<br>1.745<br>86	0.0020<br>37	0.0104<br>5
GSE37532_TREG_VS_TCONV_PPARG_KO_CD4_TCELL_FROM_VISCERAL_ADIPOSE_TISSUE<br>_UP	17<br>9	-<br>0.383<br>28	-<br>1.739<br>41	0.0020<br>37	0.0108<br>32
GSE20715_WT_VS_TLR4_KO_LUNG_DN	17<br>7	-<br>0.429<br>31	-<br>1.736<br>59	0.0020<br>37	0.0110<br>68
GSE17721_CTRL_VS_CPG_4H_BMDC_DN	18<br>8	-<br>0.419<br>92	-<br>1.718<br>06	0.0020<br>37	0.0123<br>96
GSE22935_WT_VS_MYD88_KO_MACROPHAGE_DN	18<br>2	-<br>0.544<br>58	-<br>1.690<br>51	0.0020<br>37	0.0144<br>65
GSE6875_WT_VS_FOXP3_KO_TREG_DN	17<br>8	-<br>0.406<br>37	-<br>1.668<br>94	0.0020<br>37	0.0164<br>43
GSE11961_MARGINAL_ZONE_BCELL_VS_GERMINAL_CENTER_BCELL_DAY40_DN	18<br>2	-<br>0.409<br>37	-<br>1.658<br>46	0.0020<br>37	0.0174<br>03

GSE23321_CD8_STEM_CELL_MEMORY_VS_CENTRAL_MEMORY_CD8_TCELL_UP	178	-0.49105	-1.64412	0.002037	0.018935
GSE35543_IN_VIVO_NTREG_VS_IN_VITRO_ITREG_UP	178	-0.51064	-1.63503	0.002037	0.019954
GSE10147_IL3_VS_IL3_AND_HIVP17_STIM_PDC_DN	138	-0.4842	-1.62817	0.002037	0.020739
GSE8621_UNSTIM_VS_LPS_STIM_MACROPHAGE_UP	176	-0.3659	-1.56457	0.002037	0.029514
GSE36078_UNTREATED_VS_AD5_INF_IL1R_KO_MOUSE_LUNG_DC_DN	176	-0.38632	-1.53676	0.002037	0.034508
GSE43955_1H_VS_42H_ACT_CD4_TCELL_UP	189	-0.45223	-2.0284	0.002041	0.002845
GSE29618_PRE_VS_DAY7_FLU_VACCINE_PDC_DN	180	-0.33415	-2.00216	0.002041	0.002999
GSE1112_OT1_VS_HY_CD8AB_THYMOCYTE_RTOC_CULTURE_DN	175	-0.33438	-1.94409	0.002041	0.003668
GSE1791_CTRL_VS_NEUROMEDINU_IN_T_CELL_LINE_0.8H_UP	176	-0.42331	-1.92485	0.002041	0.003991
GSE14308_NAIVE_CD4_TCELL_VS_NATURAL_TREG_DN	177	-0.4038	-1.89786	0.002041	0.004439
GSE17974_IL4_AND_ANTI_IL12_VS_UNTREATED_72H_ACT_CD4_TCELL_DN	172	-0.58059	-1.83568	0.002041	0.00621
GSE27786_CD8_TCELL_VS_NKTCELL_DN	180	-0.38706	-1.82376	0.002041	0.006654

GSE339_EX_VIVO_VS_IN_CULTURE_CD4CD8DN_DC_UP	173	-0.36227	-1.80781	0.002041	0.007362
GSE24142_EARLY_THYMIC_PROGENITOR_VS_DN2_THYMOCYTE_FETAL_DN	183	-0.5121	-1.75349	0.002041	0.009968
GSE34156_NOD2_LIGAND_VS_NOD2_AND_TLR1_TLR2_LIGAND_6H_TREATED_MONOCYTE_DN	189	-0.40431	-1.66808	0.002041	0.016505
GSE15659_NONSUPPRESSIVE_TCELL_VS_ACTIVATED_TREG_UP	153	-0.48302	-1.62969	0.002041	0.020557
GSE3337_CTRL_VS_4H_IFNG_IN_CD8POS_DC_DN	188	-0.52531	-2.05115	0.002045	0.002609
GSE32164_RESTING_DIFFERENTIATED_VS_CMYC_INHIBITED_MACROPHAGE_DN	185	-0.54349	-1.99449	0.002045	0.003061
GSE34205_RSV_VS_FLU_INF_INFANT_PBMC_DN	177	-0.5766	-1.98993	0.002045	0.003132
GSE13411_NAIVE_VS_MEMORY_BCELL_DN	176	-0.33781	-1.87994	0.002045	0.004894
GSE5589_IL6_KO_VS_IL10_KO_LPS_STIM_MACROPHAGE_180MIN_UP	177	-0.44453	-1.8532	0.002045	0.005684
GSE37534_UNTREATED_VS_GW1929_TREATED_CD4_TCELL_PPARG1_AND_FOXP3_TRASDUCED_UP	190	-0.42828	-1.84162	0.002045	0.005995
GSE42021_CD24HI_VS_CD24LOW_TREG_THYMUS_DN	183	-0.51817	-1.78959	0.002045	0.008108
GSE17721_LPS_VS_CPG_8H_BMDC_UP	183	-0.40548	-1.76555	0.002045	0.009326

GSE17721_CPG_VS_GARDIQUIMOD_12H_BMDC_DN	176	-0.36701	-1.73505	0.002045	0.011174
GSE17721_LPS_VS_PAM3CSK4_16H_BMDC_DN	183	-0.33891	-1.72118	0.002045	0.012191
GSE15930_STIM_VS_STIM_AND_TRICHOSTATINA_24H_CD8_T_CELL_UP	188	-0.45408	-1.67255	0.002045	0.016115
GSE16385_MONOCYTE_VS_MACROPHAGE_UP	177	-0.41032	-1.64668	0.002045	0.018659
GSE2770_UNTREATED_VS_TGFB_AND_IL12_TREATED_ACT_CD4_TCELL_48H_UP	177	-0.36854	-1.62919	0.002045	0.020614
GSE15330_LYMPHOID_MULTIPOTENT_VS_PRO_BCELL_UP	186	-0.41027	-1.59613	0.002045	0.024788
GSE37532_VISCERAL_ADIPOSE_TISSUE_VS_LN_DERIVED_TCONV_CD4_TCELL_UP	115	-0.47167	-1.53943	0.002045	0.033968
GSE9988_ANTI_TREM1_VS_ANTI_TREM1_AND_LPS_MONOCYTE_UP	186	-0.38849	-2.01332	0.002049	0.00291
GSE3337_CTRL_VS_16H_IFNG_IN_CD8POS_DC_UP	192	-0.50995	-1.89569	0.002049	0.004486
GSE27786_CD8_TCELL_VS_NKCELL_UP	179	-0.38369	-1.80978	0.002049	0.007259
GSE17721_CTRL_VS_CPG_8H_BMDC_DN	186	-0.44745	-1.78756	0.002049	0.008213
GSE36078_UNTREATED_VS_AD5_INF_IL1R_KO_MOUSE_LUNG_DC_UP	185	-0.41201	-1.77371	0.002049	0.008909

GSE5589_IL6_KO_VS_IL10_KO_LPS_AND_IL10_STIM_MACROPHAGE_45MIN_UP	185	-0.45155	-1.67978	0.002049	0.015451
GSE6259_33D1_POS_DC_VS_BCELL_DN	137	-0.4239	-1.65411	0.002049	0.017874
GSE1740_MCSF_VS_MCSF_AND_IFNG_DAY2_DERIVED_MACROPHAGE_WITH_IFNA_STIM_DN	152	-0.44656	-1.52008	0.002049	0.037803
GSE17721_POLYIC_VS_PAM3CSK4_4H_BMDC_DN	177	-0.34122	-2.02611	0.002053	0.002843
GSE7768_OVA_ALONE_VS_OVA_WITH_MPL_IMMUNIZED_MOUSE_WHOLE_SPLEEN_6H_DN	154	-0.50789	-2.01401	0.002053	0.002901
GSE2770_IL4_ACT_VS_ACT_CD4_TCELL_2H_DN	177	-0.44007	-1.97335	0.002053	0.003346
GSE18281_MEDULLARY_THYMOCYTE_VS_WHOLE_MEDULLA_THYMUS_DN	172	-0.40389	-1.81651	0.002053	0.006941
GSE360_DC_VS_MAC_T_GONDII_DN	193	-0.48165	-1.78191	0.002053	0.008505
GSE21033_3H_VS_12H_POLYIC_STIM_DC_UP	152	-0.41868	-1.75586	0.002053	0.009828
GSE2706_R848_VS_R848_AND_LPS_8H_STIM_DC_UP	156	-0.44911	-1.72957	0.002053	0.011535
GSE16385_IFNG_TNF_VS_UNSTIM_MACROPHAGE_ROSIGLITAZONE_TREATED_UP	157	-0.39562	-1.71691	0.002053	0.012452
GSE360_L_DONOVANI_VS_L_MAJOR_DC_UP	188	-0.44664	-1.68614	0.002053	0.014823

GSE24142_DN2_VS_DN3_THYMOCYTE_ADULT_UP	179	-0.44751	-1.6727	0.002053	0.016102
GSE22342_CD11C_HIGH_VS_LOW_DECIDUAL_MACROPHAGES_UP	168	-0.39145	-1.65145	0.002053	0.018114
GSE26928_EFF_MEM_VS_CENTR_MEM_CD4_TCELL_UP	163	-0.44286	-1.63486	0.002053	0.019974
GSE43955_10H_VS_60H_ACT_CD4_TCELL_WITH_TGFB_IL6_DN	188	-0.40033	-1.62053	0.002053	0.021718
GSE20366_TREG_VS_NAIVE_CD4_TCELL_HOMEOSTATIC_CONVERSION_UP	181	-0.40948	-1.59719	0.002053	0.024637
GSE1432_6H_VS_24H_IFNG_MICROGLIA_DN	184	-0.35124	-1.89971	0.002058	0.004384
GSE22611_UNSTIM_VS_6H_MDP_STIM_MUTANT_NOD2_TRANSDUCED_HEK293T_CELL_UP	172	-0.39569	-1.88767	0.002058	0.004667
GSE1740_MCSF_VS_MCSF_AND_IFNG_DAY2_DERIVED_MACROPHAGE_UP	162	-0.55419	-1.84175	0.002058	0.005996
GSE7460_WT_VS_FOXP3_HET_ACT_TCONV_DN	184	-0.3824	-1.83635	0.002058	0.006189
GSE7509_UNSTIM_VS_IFNA_STIM_IMMATURE_DC_DN	167	-0.67765	-1.81907	0.002058	0.006865
GSE22025_TGFB1_VS_TGFB1_AND_PROGESTERONE_TREATED_CD4_TCELL_UP	186	-0.48437	-1.75989	0.002058	0.00961
GSE2585_CTEC_VS_THYMIC_DC_UP	174	-0.45467	-1.71434	0.002058	0.012624

GSE8515_CTRL_VS_IL1_4H_STIM_MAC_UP	183	-0.4174	-1.67872	0.002058	0.015552
GSE21927_SPLEEN_VS_TUMOR_MONOCYTE_C57BL6_UP	158	-0.51269	-1.65422	0.002058	0.017858
GSE25088_CTRL_VS_IL4_AND_ROSIGLITAZONE_STIM_STAT6_KO_MACROPHAGE_UP	172	-0.30554	-1.64325	0.002058	0.018995
GSE43955_1H_VS_42H_ACT_CD4_TCELL_WITH_TGFB_IL6_DN	191	-0.43513	-1.63852	0.002058	0.019496
GSE42724_B1_BCELL_VS_PLASMABLAST_UP	185	-0.35706	-1.62011	0.002058	0.02175
GSE26488_HDAC7_KO_VS_VP16_TRANSGENIC_HDAC7_KO_DOUBLE_POSITIVE_THYMOCYTE_UP	159	-0.4085	-1.60071	0.002058	0.024225
GSE8868_SPLEEN_VS_INTESTINE_CD11B_POS_CD11C_NEG_DC_DN	182	-0.44473	-1.83444	0.002062	0.00625
GSE41978_WT_VS_ID2_KO_AND_BIM_KO_KLRG1_LOW_EFFECTOR_CD8_TCELL_UP	186	-0.42622	-1.79129	0.002062	0.008024
GSE46606_UNSTIM_VS_CD40L_IL2_IL5_DAY3_STIMULATED_BCELL_UP	170	-0.42777	-1.71988	0.002062	0.012267
GSE8515_CTRL_VS_IL1_4H_STIM_MAC_DN	180	-0.42663	-1.69384	0.002062	0.014251
GSE16385_UNTREATED_VS_12H_ROSIGLITAZONE_IL4_TREATED_MACROPHAGE_DN	174	-0.40136	-1.63356	0.002062	0.020117
GSE5542_IFNG_VS_IFNA_AND_IFNG_TREATED_EPITHELIAL_CELLS_6H_UP	174	-0.33244	-1.63114	0.002062	0.020414

GSE32901_NAIVE_VS_TH17_NEG_CD4_TCELL_DN	117	-0.51387	-1.5118	0.002062	0.039499
GSE46606_DAY1_VS_DAY3_CD40L_IL2_IL5_STIMULATED_IRF4HIGH_BCELL_UP	184	-0.3196	-1.84849	0.002066	0.005821
GSE27786_CD4_TCELL_VS_MONO_MAC_UP	182	-0.31033	-1.8428	0.002066	0.005962
GSE7219_UNSTIM_VS_LPS_AND_ANTI_CD40_STIM_NIK_NFKB2_KO_DC_DN	182	-0.51641	-1.83688	0.002066	0.006154
GSE17721_0.5H_VS_24H_PAM3CSK4_BMDC_UP	185	-0.33656	-1.82582	0.002066	0.006571
GSE15930_NAIVE_VS_48H_IN_VITRO_STIM_IL12_CD8_TCELL_UP	183	-0.46618	-1.80057	0.002066	0.007637
GSE21033_CTRL_VS_POLYIC_STIM_DC_6H_UP	182	-0.40697	-1.79679	0.002066	0.007781
GSE22282_HYPOXIA_VS_NORMOXIA_MYELOID_DC_UP	182	-0.43777	-1.78892	0.002066	0.008137
GSE2706_2H_VS_8H_R848_STIM_DC_DN	168	-0.53466	-1.76674	0.002066	0.009283
GSE11961_FOLLICULAR_BCELL_VS_GERMINAL_CENTER_BCELL_DAY7_DN	181	-0.42783	-1.69752	0.002066	0.013951
GSE11961_MEMORY_BCELL_DAY7_VS_MEMORY_BCELL_DAY40_UP	181	-0.41386	-1.67183	0.002066	0.016153
GSE46242_CTRL_VS_EGR2_DELETED_TH1_CD4_TCELL_DN	167	-0.44847	-1.65901	0.002066	0.017354

GSE17721_LPS_VS_CPG_12H_BMDC_UP	176	-0.49033	-2.02328	0.00207	0.002856
GSE3982_MAC_VS_NEUTROPHIL_DN	182	-0.49644	-1.87034	0.00207	0.005186
GSE32423_IL7_VS_IL7_IL4_MEMORY_CD8_TCELL_DN	182	-0.41267	-1.79937	0.00207	0.007691
GSE11864_CSF1_IFNG_VS_CSF1_IFNG_PAM3CYS_IN_MAC_DN	173	-0.41179	-1.74392	0.00207	0.010559
GSE13411_NAIVE_VS_SWITCHED_MEMORY_BCELL_UP	183	-0.49553	-1.57166	0.00207	0.028387
GSE2770_IL12_VS_TGFB_AND_IL12_TREATED_ACT_CD4_TCELL_6H_DN	183	-0.36862	-2.05832	0.002075	0.002628
GSE5589_IL6_KO_VS_IL10_KO_LPS_AND_IL10_STIM_MACROPHAGE_180MIN_DN	183	-0.41367	-1.84058	0.002075	0.006035
GSE39556_UNTREATED_VS_3H_POLYIC_INJ_MOUSE_NK_CELL_DN	181	-0.47442	-1.82764	0.002075	0.006522
GSE17721_LPS_VS_PAM3CSK4_1H_BMDC_DN	188	-0.4258	-1.72236	0.002075	0.012095
GSE32423_CTRL_VS_IL4_MEMORY_CD8_TCELL_DN	180	-0.40123	-1.69847	0.002075	0.013897
GSE17721_POLYIC_VS_PAM3CSK4_2H_BMDC_DN	190	-0.44283	-1.67243	0.002075	0.016118
GSE29618_PRE_VS_DAY7_FLU_VACCINE_MONOCYTE_UP	169	-0.46477	-1.66503	0.002075	0.016767

GSE15659_NAIVE_CD4_TCELL_VS_ACTIVATED_TREG_UP	150	-0.45805	-1.64986	0.002075	0.01827
GSE2770_IL12_VS_IL4_TREATED_ACT_CD4_TCELL_6H_DN	182	-0.35132	-1.95175	0.002079	0.003568
GSE7219_UNSTIM_VS_LPS_AND_ANTI_CD40_STIM_DC_DN	165	-0.55508	-1.93597	0.002079	0.003762
GSE8621_LPS_STIM_VS_LPS_PRIMED_AND_LPS_STIM_MACROPHAGE_UP	178	-0.44889	-1.58337	0.002079	0.026609
GSE33374_CD8_ALPHAALPHA_VS_ALPHABETA_CD161_HIGH_TCELL_DN	185	-0.54656	-2.10513	0.002083	0.002108
GSE2706_R848_VS_LPS_2H_STIM_DC_DN	147	-0.51934	-1.67777	0.002083	0.015618
GSE17721_PAM3CSK4_VS_GADIQUIMOD_24H_BMDC_DN	181	-0.42276	-2.03549	0.002088	0.002758
GSE9006_HEALTHY_VS_TYPE_1_DIABETES_PBMC_AT_DX_UP	179	-0.41773	-1.90857	0.002088	0.004268
GSE17721_LPS_VS_PAM3CSK4_4H_BMDC_UP	187	-0.42646	-1.84143	0.002088	0.00601
GSE21360_NAIVE_VS_TERTIARY_MEMORY_CD8_TCELL_UP	180	-0.43481	-1.72658	0.002088	0.011756
GSE4590_PRE_BCELL_VS_SMALL_PRE_BCELL_UP	151	-0.40187	-1.71507	0.002088	0.012587
GSE360_L_MAJOR_VS_B_MALAYI_HIGH_DOSE_MAC_UP	184	-0.4905	-1.65496	0.002088	0.017788

GSE21927_SPLEEN_VS_BONE_MARROW_MONOCYTE_BALBC_UP	184	-0.39589	-1.60358	0.002088	0.023829
GSE37532_WT_VS_PPARG_KO_LN_TREG_UP	164	-0.35643	-1.79573	0.002092	0.007846
GSE7460_FOXP3_MUT_VS_WT_ACT_TCONV_UP	183	-0.50404	-1.88107	0.002096	0.004877
GSE18791_CTRL_VS_NEWCASTLE_VIRUS_DC_18H_DN	162	-0.58407	-1.85024	0.002096	0.005773
GSE13484_3H_UNSTIM_VS_YF17D_VACCINE_STIM_PBMC_UP	189	-0.33066	-2.0663	0.002105	0.002533
GSE14000_UNSTIM_VS_16H_LPS_DC_UP	164	-0.33574	-1.89947	0.002105	0.004392
GSE360_L_MAJOR_VS_B_MALAYI_HIGH_DOSE_DC_DN	185	-0.36893	-1.74218	0.002105	0.010639
GSE339_CD8POS_VS_CD4CD8DN_DC_IN_CULTURE_UP	186	-0.36832	-1.73938	0.002105	0.010831
GSE40666_UNTREATED_VS_IFNA_STIM_CD8_TCELL_90MIN_DN	169	-0.30593	-1.73914	0.002105	0.010844
GSE21380_TFH_VS_GERMINAL_CENTER_TFH_CD4_TCELL_UP	187	-0.44725	-1.68207	0.002105	0.01523
GSE13522_CTRL_VS_T_CRUZI_Y_STRAIN_INF_SKIN_IFNG_KO_DN	133	-0.46171	-1.67825	0.002105	0.015575
GSE2770_UNTREATED_VS_TGFB_AND_IL12_TREATED_ACT_CD4_TCELL_2H_UP	183	-0.44586	-1.63044	0.002105	0.020495

GSE25677_R848_VS_MPL_AND_R848_STIM_BCELL_UP	135	-0.43287	-1.59356	0.002105	0.025098
GSE37301_COMMON_LYMPHOID_PROGENITOR_VS_PRO_BCELL_UP	181	-0.40488	-1.57198	0.002105	0.028335
GSE15330_WT_VS_IKAROS_KO_LYMPHOID_MULTIPOTENT_PROGENITOR_DN	176	-0.31949	-1.84913	0.00211	0.005811
GSE37301_HEMATOPOIETIC_STEM_CELL_VS_LYMPHOID_PRIMED_MPP_DN	156	-0.37384	-1.78498	0.00211	0.00834
GSE43863_TFH_VS_LY6C_INT_CXCR5POS_MEMORY_CD4_TCELL_DN	174	-0.41961	-1.62962	0.00211	0.020558
GSE29164_DAY3_VS_DAY7_UNTREATED_MELANOMA_DN	179	-0.34528	-1.9151	0.002114	0.004165
GSE2770_TGFB_AND_IL4_VS_IL12_TREATED_ACT_CD4_TCELL_48H_DN	178	-0.41044	-1.89576	0.002119	0.004489
GSE5463_CTRL_VS_DEXAMETHASONE_TREATED_THYMOCYTE_UP	187	-0.38509	-1.99172	0.002123	0.003106
GSE12198_CTRL_VS_HIGH_IL2_STIM_NK_CELL_DN	185	-0.41108	-1.90456	0.002123	0.004345
GSE360_CTRL_VS_B_MALAYI_LOW_DOSE_MAC_DN	187	-0.36441	-1.69837	0.002137	0.013892
GSE15930_STIM_VS_STIM_AND_IL12_72H_CD8_T_CELL_UP	173	-0.36545	-1.82603	0.002141	0.006568
GSE17721_LPS_VS_PAM3CSK4_12H_BMDC_UP	183	-0.46901	-1.93155	0.002146	0.003815

GSE17721_0.5H_VS_12H_LPS_BMDC_UP	174	-0.3192	-1.63257	0.002155	0.020244
GSE22886_NAIVE_CD4_TCELL_VS_48H_ACT_TH2_UP	178	-0.41373	-1.80223	0.003795	0.007602
GSE2770_UNTREATED_VS_TGFB_AND_IL12_TREATED_ACT_CD4_TCELL_6H_UP	174	-0.31172	-1.96172	0.003802	0.003455
GSE11961_PLASMA_CELL_DAY7_VS_GERMINAL_CENTER_BCELL_DAY40_DN	175	-0.34696	-1.57573	0.003802	0.027759
GSE3982_EOSINOPHIL_VS_MAC_UP	178	-0.41668	-1.68928	0.003817	0.014564
GSE3982_NEUTROPHIL_VS_NKCELL_UP	187	-0.47059	-1.92386	0.003824	0.004017
GSE37533_UNTREATED_VS_PIOGLIZATONE_TREATED_CD4_TCELL_PPARG2_AND_FOXP3_TRASDUCED_UP	138	-0.45733	-1.65079	0.003831	0.018155
GSE23321_CENTRAL_MEMORY_VS_NAIVE_CD8_TCELL_UP	184	-0.45955	-1.709	0.003846	0.013009
GSE34156_NOD2_LIGAND_VS_NOD2_AND_TLR1_TLR2_LIGAND_24H_TREATED_MONOCYTE_DN	152	-0.38259	-1.85908	0.003854	0.005471
GSE21063_CTRL_VS_ANTI_IGM_STIM_BCELL_NFATC1_KO_3H_DN	167	-0.49876	-1.91329	0.003861	0.004202
GSE26290_WT_VS_PDK1_KO_ANTI_CD3_AND_IL2_STIM_CD8_TCELL_UP	178	-0.43825	-1.81512	0.003861	0.006996
GSE13887_RESTING_VS_NO_TREATED_CD4_TCELL_UP	123	-0.49218	-1.51287	0.003861	0.039298

GSE24142_EARLY_THYMIC_PROGENITOR_VS_DN3_THYMOCYTE_UP	190	-0.52716	-1.8778	0.003868	0.00497
GSE27786_CD4_TCELL_VS_ERYTHTROBLAST_UP	178	-0.29209	-1.87163	0.003868	0.005179
GSE19401_UNSTIM_VS_RETINOIC_ACID_AND_PAM2CSK4_STIM_FOLLICULAR_DC_UP	192	-0.43474	-1.81051	0.003868	0.00721
GSE7768_OVA_WITH_LPS_VS_OVA_WITH_MPL_IMMUNIZED_MOUSE_WHOLE_SPLEEN_6H_DN	161	-0.4497	-1.95724	0.003876	0.003428
GSE45365_HEALTHY_VS_MCMV_INFECTION_CD8A_DC_IFNAR_KO_UP	177	-0.32439	-1.80592	0.003876	0.007436
GSE360_DC_VS_MAC_B_MALAYI_HIGH_DOSE_DN	189	-0.51831	-1.97695	0.003883	0.003321
GSE32986_UNSTIM_VS_GMCSF_AND_CURDLAN_HIGHDOSE_STIM_DC_DN	162	-0.5121	-1.86256	0.003883	0.005379
GSE411_UNSTIM_VS_400MIN_IL6_STIM_MACROPHAGE_UP	173	-0.31566	-1.68881	0.003883	0.014606
GSE17974_CTRL_VS_ACT_IL4_AND_ANTI_IL12_0.5H_CD4_TCELL_UP	137	-0.51239	-1.59864	0.003883	0.024462
GSE27786_NKTCELL_VS_ERYTHROBLAST_DN	187	-0.43851	-1.5364	0.003883	0.034564
GSE40274_CTRL_VS_IRF4_TRANSDUCED_ACTIVATED_CD4_TCELL_UP	154	-0.47118	-1.99615	0.003891	0.003032
GSE46242_TH1_VS_ANERGIC_TH1_CD4_TCELL_UP	173	-0.4658	-1.90208	0.003891	0.00435

GSE3720_VD1_VS_VD2_GAMMADELTA_TCELL_WITH_PMA_STIM_DN	185	-0.31346	-1.68948	0.003891	0.014556
GSE40666_WT_VS_STAT1_KO_CD8_TCELL_DN	180	-0.4022	-1.80006	0.003899	0.007658
GSE17721_0.5H_VS_4H_POLYIC_BMDC_UP	183	-0.36439	-1.71937	0.003899	0.0123
GSE23695_CD57_POS_VS_NEG_NK_CELL_DN	181	-0.27259	-1.58405	0.003899	0.026507
GSE9988_LOW_LPS_VS_VEHICLE_TREATED_MONOCYTE_DN	178	-0.4561	-2.04086	0.003906	0.002719
GSE11057_PBMC_VS_MEM_CD4_TCELL_UP	182	-0.6827	-1.81147	0.003906	0.007165
GSE29618_PRE_VS_DAY7_POST_LAIV_FLU_VACCINE_BCELL_UP	180	-0.32836	-1.77172	0.003906	0.009022
GSE22443_NAIVE_VS_ACT_AND_IL12_TREATED_CD8_TCELL_DN	172	-0.4031	-1.70632	0.003914	0.013214
GSE2770_TGFB_AND_IL4_VS_TGFB_AND_IL12_TREATED_ACT_CD4_TCELL_2H_UP	147	-0.49709	-1.67076	0.003914	0.016277
GSE3565_CTRL_VS_LPS_INJECTED_SPLENOCYTES_UP	167	-0.60487	-1.86973	0.003922	0.005202
GSE21927_SPLEEN_C57BL6_VS_4T1_TUMOR_BALBC_MONOCYTES_UP	188	-0.34662	-1.86424	0.003922	0.005327
GSE2770_TGFB_AND_IL4_VS_IL12_TREATED_ACT_CD4_TCELL_2H_DN	174	-0.47524	-1.8152	0.003922	0.006994

GSE17974_CTRL_VS_ACT_IL4_AND_ANTI_IL12_0.5H_CD4_TCELL_DN	156	-0.46044	-1.77404	0.003922	0.008891
GSE11864_CSF1_IFNG_VS_CSF1_PAM3CYS_IN_MAC_DN	164	-0.38234	-1.75875	0.003922	0.009672
GSE17721_POLYIC_VS_PAM3CSK4_16H_BMDC_UP	186	-0.39002	-1.75826	0.003922	0.009692
GSE26030_UNSTIM_VS_RESTIM_TH1_DAY15_POST_POLARIZATION_UP	190	-0.42351	-1.98441	0.003929	0.003248
GSE36891_UNSTIM_VS_PAM_TLR2_STIM_PERITONEAL_MACROPHAGE_DN	152	-0.38593	-1.90206	0.003929	0.004345
GSE3982_EOSINOPHIL_VS_CENT_MEMORY_CD4_TCELL_UP	181	-0.45958	-1.87651	0.003929	0.005018
GSE22935_UNSTIM_VS_12H_MBOVIS_BCG_STIM_MYD88_KO_MACROPHAGE_DN	168	-0.42356	-1.87569	0.003929	0.005023
GSE18791_CTRL_VS_NEWCASTLE_VIRUS_DC_12H_DN	174	-0.55729	-1.83587	0.003929	0.006204
GSE21927_BALBC_VS_C57BL6_MONOCYTE_SPLEEN_DN	150	-0.39541	-1.71921	0.003929	0.012305
GSE2770_UNTREATED_VS_TGFB_AND_IL12_TREATED_ACT_CD4_TCELL_48H_DN	181	-0.26583	-1.6559	0.003929	0.017688
GSE29618_BCELL_VS_PDC_DN	182	-0.46956	-1.96488	0.003937	0.00342
GSE22140_HEALTHY_VS_ARTHRITIC_MOUSE_CD4_TCELL_UP	186	-0.4931	-1.96121	0.003937	0.003451

GSE45365_NK_CELL_VS_BCELL_MCMV_INFECTION_UP	180	-0.45539	-1.93765	0.003937	0.003747
GSE13493_DP_VS_CD4INTCD8POS_THYMOCYTE_DN	181	-0.3542	-1.62387	0.003937	0.02131
GSE40666_WT_VS_STAT4_KO_CD8_TCELL_UP	153	-0.51779	-1.56772	0.003937	0.029023
GSE3982_NEUTROPHIL_VS_EFF_MEMORY_CD4_TCELL_UP	185	-0.46249	-1.98571	0.003945	0.003209
GSE3982_DC_VS_CENT_MEMORY_CD4_TCELL_UP	189	-0.55821	-1.95305	0.003945	0.003537
GSE29618_BCELL_VS_PDC_DAY7_FLU_VACCINE_DN	183	-0.49233	-1.91698	0.003945	0.004134
GSE21670_STAT3_KO_VS_WT_CD4_TCELL_IL6_TREATED_UP	184	-0.40974	-1.74503	0.003945	0.010499
GSE360_CTRL_VS_B_MALAYI_HIGH_DOSE_DC_DN	190	-0.40814	-1.643	0.003945	0.019007
GSE26030_TH1_VS_TH17_DAY15_POST_POLARIZATION_DN	178	-0.51285	-1.92058	0.003953	0.004055
GSE24634_TREG_VS_TCONV_POST_DAY7_IL4_CONVERSION_DN	189	-0.58068	-1.87152	0.003953	0.005172
GSE26343_UNSTIM_VS_LPS_STIM_NFAT5_KO_MACROPHAGE_DN	195	-0.583	-1.86099	0.003953	0.005428
GSE41867_NAIVE_VS_DAY30_LCMV_CLONE13_EXHAUSTED_CD8_TCELL_DN	181	-0.31216	-1.83315	0.003953	0.006294

GSE22229_RENAL_TRANSPLANT_VS_HEALTHY_PBMC_UP	177	-0.46445	-1.74355	0.003953	0.010582
GSE2585_CTEC_VS_MTEC_THYMUS_DN	177	-0.50904	-1.72437	0.003953	0.011946
GSE33162_UNTREATED_VS_4H_LPS_STIM_HDAC3_HET_MACROPHAGE_UP	177	-0.36342	-1.67095	0.003953	0.016257
GSE21360_PRIMARY_VS_SECONDARY_MEMORY_CD8_TCELL_UP	176	-0.41246	-1.62011	0.003953	0.021737
GSE1925_CTRL_VS_3H_IFNG_STIM_IFNG_PRIMED_MACROPHAGE_DN	184	-0.39942	-1.59429	0.003953	0.025019
GSE46143_CTRL_VS_LMP2A_TRANSDUCED_CD10_POS_GC_BCELL_UP	189	-0.3943	-1.56633	0.003953	0.029244
GSE41867_NAIVE_VS_DAY15_LCMV_CONE13_EFFECTOR_CD8_TCELL_UP	148	-0.48738	-1.52747	0.003953	0.036312
GSE14908_ATOPIC_VS_NONATOPIC_PATIENT_RESTING_CD4_TCELL_UP	174	-0.32286	-1.76422	0.00396	0.009392
GSE25677_MPL_VS_MPL_AND_R848_STIM_BCELL_DN	119	-0.48882	-1.75236	0.00396	0.010022
GSE29615_DAY3_VS_DAY7_LAIV_FLU_VACCINE_PBMC_DN	167	-0.45032	-1.66526	0.00396	0.016748
GSE17721_0.5H_VS_24H_CPG_BMDC_UP	184	-0.50329	-1.93804	0.003968	0.003754
GSE24634_IL4_VS_CTRL_TREATED_NAIVE_CD4_TCELL_DAY5_UP	190	-0.5842	-1.9236	0.003968	0.00401

GSE15767_MED_VS_SCS_MAC_LN_UP	185	-0.56535	-1.8731	0.003968	0.00512
GSE26030_UNSTIM_VS_RESTIM_TH1_DAY5_POST_POLARIZATION_UP	185	-0.43898	-1.85968	0.003968	0.005455
GSE7348_UNSTIM_VS_TOLERIZED_AND_LPS_STIM_MACROPHAGE_DN	149	-0.46621	-1.84291	0.003968	0.005963
GSE3982_NEUTROPHIL_VS_EFF_MEMORY_CD4_TCELL_DN	178	-0.34371	-1.81799	0.003968	0.006906
GSE3920_UNTREATED_VS_IFNA_TREATED_FIBROBLAST_UP	162	-0.44698	-1.78736	0.003968	0.008217
GSE2706_UNSTIM_VS_2H_LPS_DC_DN	173	-0.57627	-1.76072	0.003968	0.009586
GSE23505_IL6_IL1_VS_IL6_IL1_TGFB_TREATED_CD4_TCELL_UP	179	-0.3604	-1.73413	0.003968	0.01121
GSE42021_TCONV_PLN_VS_CD24INT_TCONV_THYMUS_DN	187	-0.41723	-1.72736	0.003968	0.011692
GSE27092_WT_VS_HDAC7_PHOSPHO_DEFICIENT_CD8_TCELL_UP	189	-0.47707	-1.68174	0.003968	0.01526
GSE18281_PERIMEDULLARY_CORTICAL_REGION_VS_WHOLE_MEDULLA_THYMUS_UP	186	-0.41433	-1.67644	0.003968	0.015739
GSE3982_DC_VS_MAC_LPS_STIM_DN	189	-0.47312	-1.65262	0.003968	0.017994
GSE3982_MAC_VS_NEUTROPHIL_LPS_STIM_DN	178	-0.42173	-1.61585	0.003968	0.022253

GSE13887_ACT_CD4_VS_NO_TREATED_CD4_TCELL_DN	124	-0.48728	-1.55126	0.003968	0.031753
GSE34217_MIR17_92_OVEREXPRESS_VS_WT_ACT_CD8_TCELL_UP	124	-0.43628	-1.49502	0.003968	0.043197
GSE21546_UNSTIM_VS_ANTI_CD3_STIM_ELK1_KO_DP_THYMOCYTES_DN	152	-0.40415	-1.47848	0.003968	0.047395
GSE30971_CTRL_VS_LPS_STIM_MACROPHAGE_WBP7_HET_2H_DN	175	-0.60728	-2.02302	0.003976	0.002854
GSE22886_DC_VS_MONOCYTE_DN	182	-0.58893	-1.93987	0.003976	0.003723
GSE35825_UNTREATED_VS_IFNG_STIM_MACROPHAGE_DN	172	-0.40304	-1.88666	0.003976	0.004703
GSE19888_CTRL_VS_A3R_ACTIVATION_MAST_CELL_DN	170	-0.46714	-1.51598	0.003976	0.038654
GSE30971_CTRL_VS_LPS_STIM_MACROPHAGE_WBP7_KO_2H_DN	179	-0.56433	-1.99748	0.003984	0.003006
GSE21360_NAIVE_VS_PRIMARY_MEMORY_CD8_TCELL_DN	174	-0.54882	-1.91761	0.003984	0.00412
GSE23321_CD8_STEM_CELL_MEMORY_VS_CENTRAL_MEMORY_CD8_TCELL_DN	157	-0.28558	-1.86057	0.003984	0.00544
GSE14386_UNTREATED_VS_IFNA_TREATED_ACT_PBMC_MS_PATIENT_DN	153	-0.55761	-1.84014	0.003984	0.006055
GSE22886_NAIVE_CD8_TCELL_VS_NEUTROPHIL_DN	179	-0.54236	-1.68728	0.003984	0.014734

GSE2770_TGFB_AND_IL4_VS_IL4_TREATED_ACT_CD4_TCELL_2H_UP	175	-0.4559	-1.68421	0.003984	0.015034
GSE26023_PHD3_KO_VS_WT_NEUTROPHIL_HYPOXIA_UP	180	-0.40843	-1.60978	0.003984	0.022971
GSE19923_E2A_KO_VS_HEB_AND_E2A_KO_DP_THYMOCYTE_DN	168	-0.51647	-1.60821	0.003984	0.023166
GSE10463_CD40L_AND_VA347_VS_CD40L_IN_DC_DN	144	-0.46501	-1.5989	0.003984	0.024446
GSE46025_WT_VS_FOXO1_KO_KLRG1_LOW_CD8_EFFECTOR_TCELL_UP	170	-0.41702	-1.597	0.003984	0.02466
GSE19401_UNSTIM_VS_RETINOIC_ACID_AND_PAM2CSK4_STIM_FOLLICULAR_DC_DN	193	-0.55677	-2.00853	0.003992	0.002963
GSE46606_UNSTIM_VS_CD40L_IL2_IL5_3DAY_STIMULATED_IRF4MID_SORTED_BCELL_DN	187	-0.43714	-1.84357	0.003992	0.005962
GSE2197_IMMUNOSUPPRESSIVE_DNA_VS_UNTREATED_IN_DC_UP	177	-0.43142	-1.80187	0.003992	0.00761
GSE43863_TFH_VS_LY6C_INT_CXCR5POS_MEMORY_CD4_TCELL_UP	182	-0.46963	-1.79266	0.003992	0.007976
GSE43863_DAY6_EFF_VS_DAY150_MEM_LY6C_INT_CXCR5POS_CD4_TCELL_UP	179	-0.44191	-1.78456	0.003992	0.008365
GSE2706_UNSTIM_VS_8H_R848_DC_DN	180	-0.6102	-1.77763	0.003992	0.008715
GSE17721_CTRL_VS_CPG_4H_BMDC_UP	186	-0.2962	-1.76883	0.003992	0.009166

GSE29615_CTRL_VS_LAIV_FLU_VACCINE_PBMC_UP	171	-0.4546	-1.60805	0.003992	0.023166
GSE1566_WT_VS_EZH2_KO_LN_TCELL_DN	184	-0.39574	-1.58092	0.003992	0.027009
GSE13522_WT_VS_IFNG_KO_SKING_T_CRUZI_Y_STRAIN_INF_UP	114	-0.52238	-1.55616	0.003992	0.030891
GSE40274_CTRL_VS_GATA1_TRANSDUCED_ACTIVATED_CD4_TCELL_DN	156	-0.33721	-1.54182	0.003992	0.033507
GSE43955_1H_VS_20H_ACT_CD4_TCELL_UP	191	-0.44048	-1.99338	0.004	0.003078
GSE30971_CTRL_VS_LPS_STIM_MACROPHAGE_WBP7_KO_4H_DN	172	-0.57644	-1.90228	0.004	0.00435
GSE360_CTRL_VS_B_MALAYI_LOW_DOSE_MAC_UP	183	-0.34701	-1.76456	0.004	0.009369
GSE18281_CORTICAL_VS_MEDULLARY_THYMOCYTE_DN	184	-0.49397	-1.64655	0.004	0.018668
GSE26928_NAIVE_VS_CENT_MEMORY_CD4_TCELL_DN	166	-0.38083	-1.64171	0.004	0.019147
GSE15330_LYMPHOID_MULTIPOTENT_VS_GRANULOCYTE_MONOCYTE_PROGENITOR_IKAROS_KO_UP	180	-0.28523	-1.62197	0.004	0.021565
GSE41176_UNSTIM_VS_ANTI_IGM_STIM_TAK1_KO_BCELL_3H_DN	180	-0.50486	-1.49418	0.004	0.043374
GSE36826_NORMAL_VS_STAPH_AUREUS_INF_IL1R_KO_SKIN_DN	181	-0.43571	-2.06527	0.004008	0.002535

GSE15930_STIM_VS_STIM_AND_IFNAB_48H_CD8_T_CELL_UP	183	-0.42223	-2.00084	0.004008	0.002984
GSE11057_NAIVE_VS_CENT_MEMORY_CD4_TCELL_DN	185	-0.52981	-1.97284	0.004008	0.003343
GSE32533_WT_VS_MIR17_KO_ACT_CD4_TCELL_DN	174	-0.36802	-1.96884	0.004008	0.003387
GSE360_DC_VS_MAC_M_TUBERCULOSIS_DN	188	-0.38163	-1.77952	0.004008	0.008656
GSE17721_0.5H_VS_8H_PAM3CSK4_BMDC_UP	181	-0.32614	-1.71306	0.004008	0.012698
GSE36078_UNTREATED_VS_AD5_T425A_HEXON_INF_IL1R_KO_MOUSE_LUNG_DC_DN	183	-0.44781	-1.65708	0.004008	0.017547
GSE41978_ID2_KO_VS_ID2_KO_AND_BIM_KO_KLRG1_LOW_EFFECTOR_CD8_TCELL_UP	188	-0.4589	-1.62134	0.004008	0.021623
GSE22025_UNTREATED_VS_TGFB1_AND_PROGESTERONE_TREATED_CD4_TCELL_DN	189	-0.45181	-1.57566	0.004008	0.027765
GSE29614_CTRL_VS_DAY7_TIV_FLU_VACCINE_PBMC_UP	149	-0.44458	-1.47266	0.004008	0.048916
GSE19198_CTRL_VS_IL21_TREATED_TCELL_6H_UP	176	-0.57276	-1.98927	0.004016	0.003172
GSE34006_WT_VS_A2AR_KO_TREG_DN	186	-0.49752	-1.93881	0.004016	0.003727
GSE360_L_MAJOR_VS_B_MALAYI_HIGH_DOSE_MAC_DN	186	-0.37741	-1.8088	0.004016	0.007318

GSE360_T_GONDII_VS_M_TUBERCULOSIS_MAC_DN	182	-0.4731	-1.74193	0.004016	0.010665
GSE25087_TREG_VS_TCONV_ADULT_UP	178	-0.47134	-1.72295	0.004016	0.012053
GSE2770_UNTREATED_VS_ACT_CD4_TCELL_6H_DN	182	-0.35887	-1.65213	0.004016	0.018041
GSE41176_WT_VS_TAK1_KO_UNSTIM_BCELL_UP	186	-0.41669	-1.64363	0.004016	0.018959
GSE10422_WT_VS_BAFF_TRANSGENIC_LN_BCELL_DN	122	-0.4733	-1.63071	0.004016	0.020463
GSE14026_TH1_VS_TH17_DN	181	-0.3776	-1.61399	0.004016	0.02247
GSE3982_BASOPHIL_VS_EFF_MEMORY_CD4_TCELL_DN	175	-0.38483	-1.59379	0.004016	0.025083
GSE21379_TFH_VS_NON_TFH_CD4_TCELL_DN	188	-0.48967	-1.58208	0.004016	0.026789
GSE37301_LYMPHOID_PRIMED_MPP_VS_RAG2_KO_NK_CELL_DN	173	-0.44901	-1.56426	0.004016	0.029555
GSE45365_WT_VS_IFNAR_KO_CD8A_DC_MCMV_INFECTION_DN	177	-0.45858	-1.53697	0.004016	0.034465
GSE13485_DAY3_VS_DAY21_YF17D_VACCINE_PBMC_DN	145	-0.50129	-1.50054	0.004016	0.041974
GSE5589_WT_VS_IL10_KO_LPS_AND_IL10_STIM_MACROPHAGE_180MIN_DN	109	-0.48174	-1.46586	0.004016	0.050599

GSE35825_UNTREATED_VS_IFNA_STIM_MACROPHAGE_DN	161	-0.51158	-1.92586	0.004024	0.003956
GSE36826_NORMAL_VS_STAPH_AUREUS_INF_IL1R_KO_SKIN_UP	186	-0.40973	-1.92	0.004024	0.00406
GSE20727_CTRL_VS_ROS_INH_AND_DNFB_ALLERGEN_TREATED_DC_UP	169	-0.34969	-1.82932	0.004024	0.006456
GSE40274_SATB1_VS_FOXP3_AND_SATB1_TRANSDUCED_ACTIVATED_CD4_TCELL_UP	162	-0.38597	-1.71215	0.004024	0.012764
GSE27786_CD4_VS_CD8_TCELL_DN	174	-0.3431	-1.70547	0.004024	0.013301
GSE17721_CTRL_VS_LPS_24H_BMDC_DN	184	-0.2917	-1.68156	0.004024	0.015266
GSE23308_CTRL_VS_CORTICOSTERONE_TREATED_MACROPHAGE_UP	182	-0.3912	-1.65299	0.004024	0.017965
GSE22601_DOUBLE_NEGATIVE_VS_CD8_SINGLE_POSITIVE_THYMOCYTE_UP	161	-0.46586	-1.58054	0.004024	0.02706
GSE24814_STAT5_KO_VS_WT_PRE_BCELL_DN	187	-0.37763	-1.89728	0.004032	0.004454
GSE29949_CD8_NEG_DC_SPLEEN_VS_DC_BRAIN_DN	184	-0.44065	-1.81686	0.004032	0.006943
GSE29949_MICROGLIA_VS_DC_BRAIN_UP	184	-0.41218	-1.77947	0.004032	0.008654
GSE5142_HTERT_TRANSDUCED_VS_CTRL_CD8_TCELL_LATE_PASSAGE_CLONE_DN	176	-0.36323	-1.77309	0.004032	0.008935

GSE25088_CTRL_VS_ROSIGLITAZONE_STIM_STAT6_KO_MACROPHAGE_UP	154	-0.40789	-1.70252	0.004032	0.013538
GSE37416_CTRL_VS_0H_F_TULARENSIS_LVS_NEUTROPHIL_UP	157	-0.47491	-1.65239	0.004032	0.018019
GSE40655_FOXO1_KO_VS_WT_NTREG_DN	170	-0.36121	-1.62258	0.004032	0.021486
GSE41176_WT_VS_TAK1_KO_UNSTIM_BCELL_DN	174	-0.50487	-1.54083	0.004032	0.033688
GSE45365_NK_CELL_VS_CD8_TCELL_DN	147	-0.4022	-1.52235	0.004032	0.037319
GSE24026_PD1_LIGATION_VS_CTRL_IN_ACT_TCELL_LINE_UP	182	-0.54224	-1.95201	0.00404	0.003557
GSE36826_NORMAL_VS_STAPH_AUREUS_INF_SKIN_DN	188	-0.44781	-1.92137	0.00404	0.004034
GSE14000_4H_VS_16H_LPS_DC_TRANSLATED_RNA_UP	173	-0.4801	-1.90235	0.00404	0.004351
GSE21360_NAIVE_VS_SECONDARY_MEMORY_CD8_TCELL_UP	171	-0.54679	-1.83953	0.00404	0.006063
GSE5589_LPS_VS_LPS_AND_IL10_STIM_IL10_KO_MACROPHAGE_45MIN_DN	179	-0.5907	-1.83865	0.00404	0.006081
GSE30971_2H_VS_4H_LPS_STIM_MACROPHAGE_WBP7_HET_UP	157	-0.36154	-1.72075	0.00404	0.012218
GSE360_L_DONOVANI_VS_L_MAJOR_MAC_DN	182	-0.4504	-1.66587	0.00404	0.016693

GSE27786_LSK_VS_LIN_NEG_CELL_DN	181	-0.36418	-1.6114	0.00404	0.022815
GSE13306_TREG_RA_VS_TCONV_RA_DN	178	-0.46308	-1.57485	0.00404	0.027869
GSE24634_IL4_VS_CTRL_TREATED_NAIVE_CD4_TCELL_DAY5_DN	189	-0.54759	-2.01683	0.004049	0.00287
GSE15330_HSC_VS_MEGAKARYOCYTE_ERYTHROID_PROGENITOR_IKAROS_KO_DN	180	-0.48074	-1.76106	0.004049	0.009569
GSE18791_UNSTIM_VS_NEWCATSLE_VIRUS_DC_18H_UP	176	-0.27013	-1.75017	0.004049	0.010145
GSE18281_SUBCAPSULAR_CORTICAL_REGION_VS_WHOLE_MEDULLA_THYMUS_DN	181	-0.49932	-1.63929	0.004049	0.019403
GSE37533_PPARG2_FOXP3_VS_FOXP3_TRANSDUCED_CD4_TCELL_PIOGLITAZONE_TREATED_UP	180	-0.39998	-1.57018	0.004049	0.028636
GSE43955_TH0_VS_TGFB_IL6_TH17_ACT_CD4_TCELL_10H_DN	186	-0.40599	-1.53024	0.004049	0.035758
GSE43955_TH0_VS_TGFB_IL6_IL23_TH17_ACT_CD4_TCELL_52H_DN	188	-0.41655	-1.8446	0.004057	0.005925
GSE23925_LIGHT_ZONE_VS_NAIVE_BCELL_UP	185	-0.4991	-1.8017	0.004057	0.007612
GSE36888_UNTREATED_VS_IL2_TREATED_TCELL_2H_DN	171	-0.46817	-1.75355	0.004057	0.009967
GSE17721_LPS_VS_POLYIC_16H_BMDC_DN	184	-0.37497	-1.71489	0.004057	0.012597

GSE31082_CD4_VS_CD8_SP_THYMOCYTE_DN	182	-0.40866	-1.64918	0.004057	0.018325
GSE22025_UNTREATED_VS_TGFB1_TREATED_CD4_TCELL_DN	185	-0.44851	-1.5715	0.004057	0.028392
GSE22886_TH1_VS_TH2_48H_ACT_DN	189	-0.40451	-1.95141	0.004065	0.003558
GSE360_LOW_DOSE_B_MALAYI_VS_M_TUBERCULOSIS_MAC_UP	190	-0.40368	-1.90365	0.004065	0.004346
GSE29949_MICROGLIA_BRAIN_VS_MONOCYTE_BONE_MARROW_DN	188	-0.43223	-1.86905	0.004065	0.005209
GSE3982_CTRL_VS_LPS_48H_DC_DN	186	-0.55369	-1.84972	0.004065	0.005786
GSE2706_R848_VS_LPS_8H_STIM_DC_DN	164	-0.53942	-1.83047	0.004065	0.006405
GSE360_LOW_DOSE_B_MALAYI_VS_M_TUBERCULOSIS_MAC_DN	189	-0.42283	-1.7854	0.004065	0.008324
GSE3203_UNTREATED_VS_IFNB_TREATED_LN_BCELL_DN	177	-0.44556	-1.64512	0.004065	0.018831
GSE3982_CTRL_VS_PMA_STIM_EOSINOPHIL_DN	178	-0.40466	-1.59381	0.004065	0.025086
GSE45365_BCELL_VS_CD8_TCELL_DN	148	-0.46275	-1.56638	0.004065	0.029239
GSE9037_WT_VS_IRAK4_KO_LPS_1H_STIM_BMDM_DN	177	-0.44594	-1.56587	0.004065	0.029337

GSE41176_UNSTIM_VS_ANTI_IGM_STIM_BCELL_6H_UP	183	-0.50587	-2.05466	0.004073	0.00266
GSE17721_0.5H_VS_24H_GARDIQUIMOD_BMDC_DN	177	-0.36558	-1.97344	0.004073	0.003348
GSE13738_RESTING_VS_BYSTANDER_ACTIVATED_CD4_TCELL_DN	181	-0.49542	-1.95006	0.004073	0.003601
GSE24726_WT_VS_E2_2_KO_PDC_DAY4_POST_DELETION_UP	186	-0.40768	-1.92752	0.004073	0.003917
GSE15330_MEGAKARYOCYTE_ERYTHROID_VS_GRANULOCYTE_MONOCYTE_PROGENITOR_UP	166	-0.55693	-1.90269	0.004073	0.00435
GSE36891_POLYIC_TLR3_VS_PAM_TLR2_STIM_PERITONEAL_MACROPHAGE_DN	153	-0.46038	-1.83941	0.004073	0.006067
GSE29949_CD8_NEG_DC_SPLEEN_VS_MONOCYTE_BONE_MARROW_UP	185	-0.37432	-1.79037	0.004073	0.008072
GSE17721_LPS_VS_POLYIC_1H_BMDC_UP	184	-0.43135	-1.68918	0.004073	0.014572
GSE28726_NAIVE_VS_ACTIVATED_VA24NEG_NKTCELL_DN	185	-0.46433	-1.67486	0.004073	0.015878
GSE32901_TH17_EMRICHED_VS_TH17_NEG_CD4_TCELL_UP	111	-0.49161	-1.66341	0.004073	0.016927
GSE16385_UNTREATED_VS_12H_ROSIGLITAZONE_TREATED_MACROPHAGE_DN	166	-0.44768	-1.60061	0.004073	0.024227
GSE27786_CD4_TCELL_VS_MONO_MAC_DN	181	-0.35934	-1.5016	0.004073	0.041715

GSE13485_CTRL_VS_DAY3_YF17D_VACCINE_PBMC_DN	185	-0.59158	-1.87062	0.004082	0.005181
GSE22601_IMMATURE_CD4_SINGLE_POSITIVE_VS_DOUBLE_POSITIVE_THYMOCYTE_DN	177	-0.47697	-1.84011	0.004082	0.006052
GSE29164_UNTREATED_VS_CD8_TCELL_TREATED_MELANOMA_DAY7_DN	181	-0.42933	-1.62154	0.004082	0.021602
GSE18281_PERIMEDULLARY_CORTICAL_REGION_VS_WHOLE_MEDULLA_THYMUS_DN	151	-0.46439	-1.597	0.004082	0.024653
GSE17186_MEMORY_VS_CD21HIGH_TRANSITIONAL_BCELL_UP	183	-0.39355	-1.52579	0.004082	0.036666
GSE22611_UNSTIM_VS_2H_MDP_STIM_NOD2_TRANSDUCED_HEK293T_CELL_UP	181	-0.34232	-1.49424	0.004082	0.043373
GSE18893_TCONV_VS_TREG_2H_CULTURE_DN	188	-0.4546	-1.96851	0.00409	0.003376
GSE3920_UNTREATED_VS_IFNA_TREATED_ENDOTHELIAL_CELL_UP	171	-0.53454	-1.87571	0.00409	0.005025
GSE1112_HY_CD8AB_VS_HY_CD8AA_THYMOCYTE_RTOC_CULTURE_UP	162	-0.52903	-1.82408	0.00409	0.006646
GSE6259_FLT3L_INDUCED_VS_WT_SPLENIC_DC_33D1_POS_DN	153	-0.49712	-1.5637	0.00409	0.02962
GSE339_EX_VIVO_VS_IN_CULTURE_CD4POS_DC_UP	181	-0.33948	-1.80668	0.004098	0.00739
GSE1460_CD4_THYMOCYTE_VS_NAIVE_CD4_TCELL_ADULT_BLOOD_UP	183	-0.39342	-1.78004	0.004098	0.008626

GSE17721_CTRL_VS_PAM3CSK4_6H_BMDC_UP	184	-0.34873	-1.76941	0.004098	0.009137
GSE27670_CTRL_VS_LMP1_TRANSDUCED_GC_BCELL_UP	185	-0.41816	-1.61764	0.004098	0.022006
GSE12505_WT_VS_E2_2_HET_PDC_UP	119	-0.48455	-1.60622	0.004098	0.023438
GSE14769_UNSTIM_VS_240MIN_LPS_BMDM_DN	180	-0.44296	-1.99591	0.004107	0.003027
GSE19888_ADENOSINE_A3R_INH_VS_TCELL_MEMBRANES_ACT_MAST_CELL_UP	181	-0.57178	-1.8834	0.004107	0.004786
GSE17721_POLYIC_VS_PAM3CSK4_4H_BMDC_UP	182	-0.35782	-1.80376	0.004107	0.007549
GSE27786_LSK_VS_NKTCELL_DN	174	-0.39103	-1.79523	0.004107	0.007863
GSE17721_CTRL_VS_PAM3CSK4_2H_BMDC_UP	174	-0.27055	-1.69069	0.004107	0.01445
GSE34156_UNTREATED_VS_6H_NOD2_LIGAND_TREATED_MONOCYTE_DN	166	-0.5383	-1.9776	0.004115	0.003329
GSE20500_CTRL_VS_RETINOIC_ACID_TREATED_CD4_TCELL_DN	182	-0.38553	-1.59076	0.004115	0.025456
GSE17186_BLOOD_VS_CORD_BLOOD_NAIVE_BCELL_UP	183	-0.33105	-1.80377	0.004124	0.007552
GSE16385_MONOCYTE_VS_12H_ROSIGLITAZONE_IL4_TREATED_MACROPHAGE_DN	186	-0.34269	-1.95201	0.004132	0.003561

GSE44955_MCSF_VS_MCSF_AND_IL27_STIM_MACROPHAGE_UP	182	-0.48649	-1.89586	0.004132	0.00449
GSE7852_LN_VS_FAT_TREG_DN	186	-0.42929	-1.7052	0.004132	0.013323
GSE22886_NEUTROPHIL_VS_MONOCYTE_UP	178	-0.48881	-1.5179	0.004132	0.038243
GSE5099_MONOCYTE_VS_CLASSICAL_M1_MACROPHAGE_UP	179	-0.38439	-1.51637	0.004132	0.038579
GSE19923_HEB_KO_VS_HEB_AND_E2A_KO_DP_THYMOCYTE_DN	187	-0.48456	-1.90116	0.004141	0.004356
GSE17721_0.5H_VS_24H_POLYIC_BMDC_UP	183	-0.37849	-1.66805	0.004141	0.016502
GSE17721_CTRL_VS_GARDIQUIMOD_0.5H_BMDC_DN	181	-0.37645	-1.60776	0.004141	0.023202
GSE29618_PRE_VS_DAY7_FLU_VACCINE_PDC_UP	184	-0.44991	-1.5121	0.004149	0.039452
GSE7219_WT_VS_NIK_NFKB2_KO_DC_UP	177	-0.52906	-1.86959	0.004158	0.005205
GSE13738_RESTING_VS_TCR_ACTIVATED_CD4_TCELL_DN	180	-0.43258	-1.84252	0.004167	0.005968
GSE15930_STIM_VS_STIM_AND_IL12_72H_CD8_T_CELL_DN	189	-0.41801	-1.63904	0.004167	0.019428
GSE19941_UNSTIM_VS_LPS_AND_IL10_STIM_IL10_KO_NFKBP50_KO_MACROPHAGE_DN	174	-0.40512	-1.57643	0.004167	0.027657

GSE20500_RETINOIC_ACID_VS_RARA_ANTAGONIST_TREATED_CD4_TCELL_UP	182	-0.42406	-1.63918	0.004175	0.019407
GSE28783_CTRL_ANTI_MIR_VS_UNTREATED_ATHEROSCLEROSIS_MACROPHAGE_DN	187	-0.45077	-1.67498	0.004193	0.015864
GSE43955_1H_VS_10H_ACT_CD4_TCELL_DN	186	-0.41582	-1.61079	0.004219	0.022876
GSE17721_LPS_VS_GARDIQUIMOD_2H_BMDC_DN	175	-0.27536	-1.82689	0.004228	0.006562
GSE14769_UNSTIM_VS_20MIN_LPS_BMDM_DN	180	-0.41665	-1.75897	0.004237	0.009663
GSE22611_NOD2_VS_MUTANT_NOD2_TRANSDUCED_HEK293T_CELL_DN	165	-0.46491	-1.66132	0.004237	0.017105
GSE2124_CTRL_VS_LYMPHOTOXIN_BETA_TREATED_MLN_DN	172	-0.35739	-1.61372	0.004237	0.022508
GSE41978_ID2_KO_AND_BIM_KO_VS_BIM_KO_KLRG1_LOW_EFFECTOR_CD8_TCELL_UP	187	-0.40005	-1.58097	0.00431	0.027002
GSE15750_DAY6_VS_DAY10_TRAF6KO_EFF_CD8_TCELL_DN	174	-0.43376	-1.82675	0.00565	0.006562
GSE25123_CTRL_VS_ROSIGLITAZONE_STIM_PPARG_KO_MACROPHAGE_UP	183	-0.61703	-1.74095	0.005693	0.010725
GSE36009_UNSTIM_VS_LPS_STIM_DC_DN	176	-0.29998	-1.85527	0.005714	0.00561
GSE41867_NAIVE_VS_DAY15_LCMV_EFFECTOR_CD8_TCELL_DN	175	-0.38629	-1.88853	0.005736	0.004645

GSE16385_UNTREATED_VS_12H_IFNG_TNF_TREATED_MACROPHAGE_UP	159	-0.43854	-1.56288	0.005736	0.029763
GSE17721_CTRL_VS_PAM3CSK4_0.5H_BMDC_DN	183	-0.40026	-1.62284	0.005747	0.021456
GSE21063_3H_VS_16H_ANTI_IGM_STIM_BCELL_DN	165	-0.3407	-1.81166	0.00578	0.007159
GSE29618_PRE_VS_DAY7_POST_TIV_FLU_VACCINE_BCELL_UP	180	-0.47024	-1.50309	0.00578	0.041398
GSE9006_HEALTHY_VS_TYPE_2_DIABETES_PBMC_AT_DX_DN	184	-0.4414	-1.75375	0.005792	0.009952
GSE6259_33D1_POS_VS_DEC205_POS_FLT3L_INDUCED_SPLENIC_DC_UP	164	-0.48557	-1.71515	0.005792	0.012588
GSE15659_RESTING_TREG_VS_NONSUPPRESSIVE_TCELL_DN	183	-0.35438	-1.55549	0.005792	0.031004
GSE5589_LPS_VS_LPS_AND_IL10_STIM_IL6_KO_MACROPHAGE_45MIN_UP	179	-0.37151	-1.83139	0.005803	0.006371
GSE18791_CTRL_VS_NEWCASTLE_VIRUS_DC_2H_DN	151	-0.47123	-1.65109	0.005814	0.018134
GSE24634_TEFF_VS_TCONV_DAY3_IN_CULTURE_DN	182	-0.62509	-1.90206	0.005825	0.004348
GSE45365_NK_CELL_VS_CD11B_DC_UP	181	-0.46436	-1.48418	0.005825	0.045853
GSE9960_HEALTHY_VS_GRAM_NEG_SEPSIS_PBMC_UP	182	-0.41731	-1.92802	0.005837	0.003913

GSE39820_TGFBETA1_IL6_VS_TGFBETA1_IL6_IL23A_TREATED_CD4_TCELL_UP	189	-0.4085	-1.90101	0.005837	0.004361
GSE6092_B_BURGDOFERI_VS_B_BURGDORFERI_AND_IFNG_STIM_ENDOTHELIAL_CELL_DN	169	-0.48928	-1.88388	0.005837	0.004774
GSE15750_WT_VS_TRAF6KO_DAY10_EFF_CD8_TCELL_UP	176	-0.32327	-1.66194	0.005837	0.017067
GSE9960_GRAM_NEG_VS_GRAM_NEG_AND_POS_SEPSIS_PBMC_UP	153	-0.45118	-1.58606	0.005837	0.026229
GSE6269_HEALTHY_VS_STAPH_AUREUS_INF_PBMC_UP	151	-0.49313	-2.01184	0.005859	0.00292
GSE29618_LAIV_VS_TIV_FLU_VACCINE_DAY7_MDC_UP	180	-0.41727	-1.7909	0.005859	0.008041
GSE36527_CD62L_HIGH_VS_CD62L_LOW_TREG_CD69_NEG_KLRG1_NEG_UP	189	-0.46022	-1.97079	0.005871	0.003371
GSE17721_CTRL_VS_GARDIQUIMOD_6H_BMDC_UP	193	-0.31518	-1.74604	0.005871	0.01044
GSE22196_HEALTHY_VS_OBESE_MOUSE_SKIN_GAMMADELTA_TCELL_UP	183	-0.55032	-2.01362	0.005882	0.002915
GSE36826_WT_VS_IL1R_KO_SKIN_STAPH_AUREUS_INF_DN	179	-0.51974	-1.96107	0.005882	0.003439
GSE41176_WT_VS_TAK1_KO_ANTI_IGM_STIM_BCELL_24H_DN	172	-0.37599	-1.92996	0.005894	0.003861
GSE10325_CD4_TCELL_VS_MYELOID_DN	189	-0.6164	-1.85972	0.005894	0.005461

GSE37319_WT_VS_RC3H1_KO_CD44LOW_CD8_TCELL_UP	131	-0.42708	-1.65892	0.005894	0.017358
GSE25123_CTRL_VS_ROSIGLITAZONE_STIM_MACROPHAGE_DN	175	-0.41052	-1.63709	0.005894	0.019692
GSE21927_SPLENIC_C26GM_TUMOROUS_VS_4T1_TUMOR_MONOCYTES_DN	165	-0.33928	-1.61526	0.005894	0.022315
GSE13411_IGM_VS_SWITCHED_MEMORY_BCELL_UP	182	-0.43911	-1.49592	0.005894	0.043006
GSE34156_UNTREATED_VS_6H_TLR1_TLR2_LIGAND_TREATED_MONOCYTE_UP	181	-0.59834	-1.83501	0.005906	0.00623
GSE37301_HEMATOPOIETIC_STEM_CELL_VS_LYMPHOID_PRIMED_MPP_UP	166	-0.30581	-1.71358	0.005906	0.012667
GSE3720_UNSTIM_VS_PMA_STIM_VD2_GAMMADELTA_TCELL_UP	157	-0.44962	-1.89096	0.005917	0.004602
GSE36888_UNTREATED_VS_IL2_TREATED_STAT5_AB_KNOCKIN_TCELL_6H_UP	171	-0.56299	-1.84225	0.005917	0.005978
GSE21360_PRIMARY_VS_QUATERNARY_MEMORY_CD8_TCELL_UP	169	-0.53641	-1.81348	0.005917	0.007089
GSE28449_WT_VS_LRF_KO_GERMINAL_CENTER_BCELL_UP	184	-0.46931	-1.65211	0.005917	0.018037
GSE34205_RSV_VS_FLU_INF_INFANT_PBMC_UP	160	-0.49582	-1.60133	0.005917	0.024172
GSE6674_UNSTIM_VS_PL2_3_STIM_BCELL_UP	141	-0.38518	-1.53564	0.005917	0.034695

GSE22443_IL2_VS_IL12_TREATED_ACT_CD8_TCELL_UP	184	-0.43091	-1.51456	0.005917	0.038971
GSE42088_UNINF_VS_LEISHMANIA_INF_DC_2H_DN	184	-0.46124	-1.78928	0.005929	0.008122
GSE31082_DN_VS_DP_THYMOCYTE_DN	185	-0.36217	-1.78906	0.005929	0.008135
GSE32986_GMCSF_AND_CURDLAN_LOWDOSE_VS_GMCSF_AND_CURDLAN_HIGHDOSE_STIM_DC_UP	179	-0.33373	-1.74029	0.005929	0.010769
GSE9006_1MONTH_VS_4MONTH_POST_TYPE_1_DIABETES_DX_PBMC_UP	182	-0.43297	-1.64079	0.005929	0.019209
GSE45365_WT_VS_IFNAR_KO_BCELL_MCMV_INFECTION_UP	149	-0.41949	-1.61805	0.005929	0.02195
GSE17721_LPS_VS_POLYIC_0.5H_BMDC_DN	184	-0.37641	-1.54506	0.005929	0.032879
GSE9988_ANTI_TREM1_VS_VEHICLE_TREATED_MONOCYTES_DN	185	-0.53925	-1.97098	0.005941	0.00337
GSE2128_C57BL6_VS_NOD_THYMOCYTE_MIMETOPE_NEGATIVE_SELECTION_DN	184	-0.42098	-1.84095	0.005941	0.006027
GSE21360_TERTIARY_VS_QUATERNARY_MEMORY_CD8_TCELL_UP	174	-0.5179	-1.82465	0.005941	0.006631
GSE5099_UNSTIM_VS_MCSF_TREATED_MONOCYTE_DAY7_DN	179	-0.3863	-1.6141	0.005941	0.022455
GSE46242_CTRL_VS_EGR2_DELETED_TH1_CD4_TCELL_UP	167	-0.41644	-1.71439	0.005952	0.012622

GSE8685_IL2_STARVED_VS_IL21_ACT_IL2_STARVED_CD4_TCELL_UP	182	-0.32415	-1.68088	0.005952	0.015325
GSE41867_NAIVE_VS_DAY8_LCMV_CLONE13_EFFECTOR_CD8_TCELL_DN	161	-0.34099	-1.61338	0.005952	0.022545
GSE24726_WT_VS_E2_2_KO_PDC_DN	183	-0.41098	-1.93407	0.005964	0.003774
GSE26890_CXCR1_NEG_VS_POS_EFFECTOR_CD8_TCELL_UP	179	-0.52156	-1.92984	0.005964	0.003858
GSE24634_IL4_VS_CTRL_TREATED_NAIVE_CD4_TCELL_DAY3_DN	188	-0.59839	-1.8803	0.005964	0.004888
GSE24142_DN2_VS_DN3_THYMOCYTE_FETAL_UP	187	-0.43646	-1.74581	0.005964	0.01045
GSE34006_A2AR_KO_VS_A2AR_AGONIST_TREATED_TREG_DN	184	-0.29464	-1.79601	0.005976	0.007813
GSE15930_NAIVE_VS_48H_IN_VITRO_STIM_CD8_TCELL_UP	185	-0.43296	-1.64474	0.005976	0.018857
GSE2405_HEAT_KILLED_LYSATE_VS_LIVE_A_PHAGOCYTOPHILUM_STIM_NEUTROPHIL_24H_DN	185	-0.37486	-1.60606	0.005976	0.023454
GSE16385_MONOCYTE_VS_12H_IFNG_TNF_TREATED_MACROPHAGE_DN	175	-0.41036	-1.5723	0.005976	0.028282
GSE21927_SPLEEN_VS_TUMOR_MONOCYTE_C57BL6_DN	157	-0.3803	-1.53449	0.005976	0.034895
GSE41867_DAY15_EFFECTOR_VS_DAY30_MEMORY_CD8_TCELL_LCMV_ARMSTRONG_UP	178	-0.36908	-1.51962	0.005976	0.037899

GSE5589_LPS_VS_LPS_AND_IL10_STIM_IL6_KO_MACROPHAGE_180MIN_UP	182	-0.34947	-1.96757	0.005988	0.003381
GSE1925_3H_VS_24H_IFNG_STIM_MACROPHAGE_DN	178	-0.38706	-1.8306	0.005988	0.006404
GSE18281_SUBCAPSULAR_CORTICAL_REGION_VS_WHOLE_CORTEX_THYMUS_UP	189	-0.39741	-1.69026	0.005988	0.014492
GSE25123_WT_VS_PPARG_KO_MACROPHAGE_DN	176	-0.47717	-2.02535	0.006	0.002829
GSE19888_CTRL_VS_A3R_ACT_TREATED_MAST_CELL_PRETREATED_WITH_A3R_INH_DN	183	-0.38551	-1.71442	0.006	0.012625
GSE15930_STIM_VS_STIM_AND_TRICHOSTATINA_24H_CD8_T_CELL_DN	178	-0.29921	-1.63771	0.006	0.019618
GSE39820_TGFBETA1_VS_TGFBETA3_IN_IL6_IL23A_TREATED_CD4_TCELL_UP	179	-0.36853	-1.6084	0.006	0.023148
GSE46606_UNSTIM_VS_CD40L_IL2_IL5_3DAY_STIMULATED_IRF4HIGH_SORTED_BCELL_DN	185	-0.46834	-1.5649	0.006	0.029479
GSE35543_IN_VIVO_NTREG_VS_CONVERTED_EX_ITREG_DN	178	-0.48671	-1.51691	0.006	0.03846
GSE2770_IL12_ACT_VS_ACT_CD4_TCELL_2H_UP	175	-0.47615	-1.76998	0.006012	0.009107
GSE18804_SPLEEN_MACROPHAGE_VS_COLON_TUMORAL_MACROPHAGE_DN	177	-0.45389	-1.74544	0.006012	0.010471
GSE17721_LPS_VS_PAM3CSK4_2H_BMDC_DN	176	-0.37786	-1.53798	0.006012	0.034259

GSE37416_CTRL_VS_0H_F_TULARENSIS_LVS_NEUTROPHIL_DN	139	-0.46108	-1.49642	0.006012	0.042904
GSE23695_CD57_POS_VS_NEG_NK_CELL_UP	176	-0.32363	-1.89433	0.006024	0.00452
GSE3982_EOSINOPHIL_VS_NEUTROPHIL_DN	179	-0.47125	-1.85949	0.006024	0.00546
GSE26343_WT_VS_NFAT5_KO_MACROPHAGE_DN	189	-0.51715	-1.85658	0.006024	0.005563
GSE46606_IRF4_KO_VS_WT_UNSTIM_BCELL_UP	183	-0.36468	-1.75176	0.006024	0.010061
GSE339_EX_VIVO_VS_IN_CULTURE_CD8POS_DC_DN	192	-0.36747	-1.71453	0.006024	0.012619
GSE13485_DAY3_VS_DAY7_YF17D_VACCINE_PBMC_UP	155	-0.42899	-1.64337	0.006024	0.018987
GSE17721_LPS_VS_GARDIQUIMOD_1H_BMDC_UP	184	-0.4278	-1.58046	0.006024	0.027063
GSE5589_LPS_AND_IL10_VS_LPS_AND_IL6_STIM_IL6_KO_MACROPHAGE_45MIN_UP	186	-0.62706	-1.94992	0.006036	0.003597
GSE17721_12H_VS_24H_LPS_BMDC_UP	186	-0.48403	-1.9278	0.006036	0.003916
GSE11057_NAIVE_CD4_VS_PBMC_CD4_TCELL_DN	188	-0.50777	-1.92268	0.006036	0.004004
GSE43955_TH0_VS_TGFB_IL6_TH17_ACT_CD4_TCELL_10H_UP	188	-0.42955	-1.82366	0.006036	0.006649

GSE40666_UNTREATED_VS_IFNA_STIM_STAT4_KO_EFFECTOR_CD8_TCELL_90MIN_UP	181	-0.46641	-1.78524	0.006036	0.008327
GSE40443_INDUCED_VS_TOTAL_TREG_DN	173	-0.46392	-1.77578	0.006036	0.008797
GSE29618_LAIV_VS_TIV_FLU_VACCINE_DAY7_PDC_UP	177	-0.29475	-1.62599	0.006036	0.021044
GSE15930_STIM_VS_STIM_AND_IL12_24H_CD8_T_CELL_DN	186	-0.42292	-1.62015	0.006036	0.021753
GSE45365_HEALTHY_VS_MCMV_INFECTION_CD11B_DC_IFNAR_KO_UP	169	-0.40924	-1.61966	0.006036	0.021786
GSE2770_TGFB_AND_IL4_ACT_VS_ACT_CD4_TCELL_48H_UP	181	-0.41492	-1.93244	0.006048	0.003803
GSE360_DC_VS_MAC_B_MALAYI_HIGH_DOSE_UP	190	-0.41472	-1.8567	0.006048	0.005559
GSE22103_LPS_VS_GMCSF_AND_IFNG_STIM_NEUTROPHIL_DN	182	-0.32225	-1.78544	0.006048	0.008329
GSE6259_FLT3L_INDUCED_DEC205_POS_DC_VS_CD4_TCELL_UP	152	-0.31136	-1.77613	0.006048	0.008785
GSE339_CD4POS_VS_CD8POS_DC_DN	187	-0.32899	-1.67639	0.006048	0.015737
GSE24814_STAT5_KO_VS_WT_PRE_BCELL_UP	189	-0.47271	-1.56361	0.006048	0.029627
GSE3565_CTRL_VS_LPS_INJECTED_DUSP1_KO_SPLENOCYTES_UP	169	-0.60253	-1.83116	0.006061	0.006374

GSE46606_IRF4MID_VS_WT_CD40L_IL2_IL5_DAY3_STIMULATED_BCELL_UP	180	-0.39927	-1.75559	0.006061	0.009841
GSE2706_UNSTIM_VS_2H_R848_DC_DN	175	-0.56786	-1.74873	0.006061	0.010238
GSE360_CTRL_VS_B_MALAYI_HIGH_DOSE_MAC_UP	181	-0.30677	-1.60301	0.006061	0.023932
GSE3039_ALPHAALPHA_CD8_TCELL_VS_B1_BCELL_UP	188	-0.48541	-1.91042	0.006073	0.004255
GSE36527_CD69_NEG_VS_POS_TREG_CD62L_LOS_KLRG1_NEG_UP	190	-0.52663	-1.87997	0.006073	0.004896
GSE5589_UNSTIM_VS_180MIN_LPS_STIM_MACROPHAGE_DN	177	-0.46368	-1.86447	0.006073	0.00533
GSE17721_PAM3CSK4_VS_GADIQUIMOD_8H_BMDC_DN	184	-0.39155	-1.81981	0.006073	0.006818
GSE16450_CTRL_VS_IFNA_12H_STIM_IMMATURE_NEURON_CELL_LINE_UP	170	-0.39158	-1.77854	0.006073	0.008662
GSE15659_CD45RA_NEG_CD4_TCELL_VS_ACTIVATED_TREG_UP	153	-0.46202	-1.59287	0.006073	0.025188
GSE2706_LPS_VS_R848_AND_LPS_8H_STIM_DC_DN	165	-0.44946	-1.50459	0.006073	0.041072
GSE22886_NAIVE_CD4_TCELL_VS_MEMORY_TCELL_DN	178	-0.42207	-1.97899	0.006085	0.003313
GSE20715_WT_VS_TLR4_KO_LUNG_UP	183	-0.50528	-1.87593	0.006085	0.005026

GSE7460_FOXP3_MUT_VS_WT_ACT_WITH_TGFB_TCONV_UP	182	-0.47653	-1.79185	0.006085	0.007996
GSE3982_DC_VS_NKCELL_UP	187	-0.46071	-1.78394	0.006098	0.008399
GSE11924_TFH_VS_TH1_CD4_TCELL_DN	175	-0.37592	-1.60843	0.006098	0.023158
GSE17721_0.5H_VS_24H_GARDIQUIMOD_BMDC_UP	176	-0.31855	-1.76002	0.00611	0.009609
GSE14415_INDUCED_TREG_VS_FOXP3_KO_INDUCED_TREG_IL2_CULTURE_UP	163	-0.5476	-1.73924	0.00611	0.010834
GSE9878_CTRL_VS_EBF_TRANSDUCED_PAX5_KO_PRO_BCELL_UP	189	-0.39308	-1.63428	0.00611	0.020037
GSE21360_NAIVE_VS_TERTIARY_MEMORY_CD8_TCELL_DN	184	-0.35813	-1.6103	0.00611	0.022928
GSE339_CD4POS_VS_CD4CD8DN_DC_IN_CULTURE_DN	191	-0.37436	-1.55706	0.00611	0.030749
GSE8384_CTRL_VS_B_ABORTUS_4H_MAC_CELL_LINE_DN	193	-0.45317	-1.89739	0.006122	0.004452
GSE17721_CTRL_VS_PAM3CSK4_4H_BMDC_UP	178	-0.33432	-1.77041	0.006122	0.009086
GSE17186_MEMORY_VS_NAIVE_BCELL_DN	184	-0.402	-1.72917	0.006122	0.011546
GSE40274_XBP1_VS_FOXP3_AND_XBP1_TRANSDUCED_ACTIVATED_CD4_TCELL_DN	181	-0.35232	-1.7113	0.006122	0.012821

GSE13522_CTRL_VS_T_CRUZI_Y_STRAIN_INF_SKIN_IFNG_KO_UP	151	-0.32524	-1.68613	0.006122	0.014821
GSE9946_MATURE_STIMULATORY_VS_PROSTAGLANDINE2_TREATED_MATURE_DC_DN	126	-0.50488	-1.57339	0.006122	0.028117
GSE20727_ROS_INH_VS_ROS_INH_AND_DNFB_ALLERGEN_TREATED_DC_UP	176	-0.5145	-1.90567	0.006135	0.00432
GSE2935_UV_INACTIVATED_VS_LIVE_SENDAI_VIRUS_INF_MACROPHAGE_DN	169	-0.62028	-1.84918	0.006135	0.005811
GSE3720_UNSTIM_VS_LPS_STIM_VD2_GAMMADELTA_TCELL_DN	136	-0.49273	-1.6016	0.006135	0.024152
GSE16522_MEMORY_VS_NAIVE_CD8_TCELL_DN	179	-0.40924	-1.59312	0.006135	0.025166
GSE27859_MACROPHAGE_VS_DC_UP	159	-0.50969	-1.90964	0.006148	0.004257
GSE43955_TGFB_IL6_VS_TGFB_IL6_IL23_TH17_ACT_CD4_TCELL_60H_DN	184	-0.45282	-1.67603	0.006148	0.015771
GSE17721_CTRL_VS_POLYIC_24H_BMDC_DN	184	-0.37873	-1.66749	0.006148	0.016561
GSE17721_CTRL_VS_LPS_0.5H_BMDC_DN	190	-0.38241	-1.59482	0.006148	0.024977
GSE39820_TGFBETA1_VS_TGFBETA3_IN_IL6_TREATED_CD4_TCELL_DN	179	-0.34457	-1.51339	0.006148	0.039182
GSE24210_IL35_TREATED_VS_RESTING_TREG_DN	179	-0.45666	-1.47166	0.006148	0.04912

GSE9037_WT_VS_IRAK4_KO_LPS_4H_STIM_BMDM_UP	185	-0.3829	-1.92457	0.00616	0.004001
GSE15330_LYMPHOID_MULTIPOTENT_VS_GRANULOCYTE_MONOCYTE_PROGENITOR_IKAROS_KO_DN	186	-0.40077	-1.83835	0.00616	0.006087
GSE27786_NKTCELL_VS_ERYTHROBLAST_UP	181	-0.2612	-1.69685	0.00616	0.014008
GSE2826_WT_VS_XID_BCELL_UP	186	-0.36105	-1.68301	0.00616	0.015135
GSE2770_IL12_VS_TGFB_AND_IL12_TREATED_ACT_CD4_TCELL_2H_DN	157	-0.39214	-1.55926	0.00616	0.030361
GSE9988_LPS_VS_LOW_LPS_MONOCYTE_UP	143	-0.46822	-1.49452	0.00616	0.043309
GSE21546_ELK1_KO_VS_SAP1A_KO_AND_ELK1_KO_DP_THYMOCYTES_UP	172	-0.4846	-1.87337	0.006173	0.005119
GSE11367_CTRL_VS_IL17_TREATED_SMOOTH_MUSCLE_CELL_DN	102	-0.45641	-1.53317	0.006173	0.03516
GSE9509_10MIN_VS_30MIN_LPS_STIM_IL10_KO_MACROPHAGE_UP	172	-0.43535	-1.91727	0.006186	0.004121
GSE7509_UNSTIM_VS_FCGRIIB_STIM_DC_DN	167	-0.64127	-1.85	0.006186	0.005778
GSE23502_BM_VS_COLON_TUMOR_HDC_KO_MYELOID_DERIVED_SUPPRESSOR_CELL_DN	182	-0.39497	-1.88064	0.006198	0.004885
GSE46606_DAY1_VS_DAY3_CD40L_IL2_IL5_STIMULATED_BCELL_DN	176	-0.43667	-1.74639	0.006198	0.010418

GSE8685_IL2_STARVED_VS_IL15_ACT_IL2_STARVED_CD4_TCELL_DN	183	-0.325	-1.59995	0.006198	0.024286
GSE32423_CTRL_VS_IL7_MEMORY_CD8_TCELL_UP	175	-0.47523	-1.55493	0.006198	0.031097
GSE3982_NEUTROPHIL_VS_BASOPHIL_DN	172	-0.33574	-1.87155	0.006211	0.005173
GSE15930_STIM_VS_STIM_AND_TRICHOSTATINA_48H_CD8_T_CELL_UP	191	-0.39169	-1.77425	0.006211	0.008884
GSE19923_HEB_KO_VS_HEB_AND_E2A_KO_DP_THYMOCYTE_UP	182	-0.31356	-1.70079	0.006211	0.013672
GSE3982_MAC_VS_BASOPHIL_UP	185	-0.3563	-1.66434	0.006224	0.016831
GSE17721_0.5H_VS_4H_GARDIQUIMOD_BMDC_UP	173	-0.28608	-1.64486	0.006224	0.018842
GSE29618_PRE_VS_DAY7_POST_LAIV_FLU_VACCINE_MDC_UP	173	-0.44338	-1.5985	0.006237	0.024482
GSE17721_POLYIC_VS_PAM3CSK4_8H_BMDC_UP	180	-0.35619	-1.75353	0.006263	0.009965
GSE32423_IL7_VS_IL4_MEMORY_CD8_TCELL_DN	178	-0.38837	-1.73621	0.006289	0.011096
GSE36476_CTRL_VS_TSST_ACT_72H_MEMORY_CD4_TCELL_OLD_UP	181	-0.42666	-1.6107	0.006303	0.022875
GSE13522_WT_VS_IFNAR_KO_SKING_T_CRUZI_Y_STRAIN_INF_DN	178	-0.3979	-1.47754	0.006329	0.047658

GSE2770_UNTREATED_VS_TGFB_AND_IL4_TREATED_ACT_CD4_TCELL_2H_UP	175	-0.30212	-1.713	0.006356	0.012706
GSE17721_12H_VS_24H_PAM3CSK4_BMDC_UP	180	-0.41023	-1.78849	0.006383	0.008151
GSE1925_3H_VS_24H_IFNG_STIM_MACROPHAGE_UP	174	-0.41063	-1.56103	0.006383	0.030073
GSE17721_LPS_VS_CPG_6H_BMDC_DN	180	-0.36381	-1.61875	0.00641	0.021868
GSE14908_ATOPIC_VS_NONATOPIC_PATIENT_HDM_STIM_CD4_TCELL_UP	179	-0.31726	-1.75224	0.006438	0.010027
GSE15330_LYMPHOID_MULTIPOTENT_VS_GRANULOCYTE_MONOCYTE_PROGENITOR_DN	173	-0.35014	-1.76411	0.006466	0.009388
GSE20500_CTRL_VS_RARA_ANTAGONIST_TREATED_CD4_TCELL_UP	186	-0.35608	-1.58903	0.00759	0.025727
GSE6092_CTRL_VS_BORRELIA_BIRGDOFERI_INF_ENDOTHELIAL_CELL_DN	144	-0.39403	-1.56258	0.00759	0.029811
GSE5099_MONOCYTE_VS_ALTERNATIVE_M2_MACROPHAGE_DN	165	-0.31857	-1.57562	0.007678	0.027764
GSE21670_UNTREATED_VS_TGFB_TREATED_STAT3_KO_CD4_TCELL_UP	184	-0.41369	-1.57558	0.007692	0.02776
GSE11884_WT_VS_FURIN_KO_NAIVE_CD4_TCELL_DN	119	-0.36976	-1.49623	0.007692	0.042939
GSE41867_LCMV_ARMSTRONG_VS_CLONE13_DAY15_EFFECTOR_CD8_TCELL_DN	148	-0.44762	-1.48412	0.007692	0.045854

GSE24972_WT_VS_IRF8_KO_MARGINAL_ZONE_SPLEEN_BCELL_DN	176	-0.47053	-1.58537	0.007707	0.026299
GSE6259_FLT3L_INDUCED_33D1_POS_DC_VS_CD8_TCELL_UP	133	-0.43182	-1.69454	0.007752	0.014191
GSE7460_FOXP3_MUT_VS_HET_ACT_TCONV_DN	186	-0.38523	-1.49674	0.007782	0.042846
GSE19888_CTRL_VS_A3R_INHIBITOR_TREATED_MAST_CELL_DN	164	-0.45747	-1.85079	0.007797	0.00575
GSE4984_UNTREATED_VS_GALECTIN1_TREATED_DC_UP	169	-0.50965	-1.8376	0.007797	0.006106
GSE360_L_DONOVANI_VS_B_MALAYI_LOW_DOSE_MAC_DN	186	-0.35498	-1.81225	0.007797	0.007128
GSE9650_NAIVE_VS_EFF_CD8_TCELL_UP	188	-0.3004	-1.69822	0.007797	0.013904
GSE19888_ADENOSINE_A3R_INH_VS_INH_PRETREAT_AND_ACT_WITH_TCELL_MEMBRANES_MAST_CELL_DN	172	-0.44453	-1.47468	0.007797	0.048387
GSE22886_NAIVE_TCELL_VS_NEUTROPHIL_DN	175	-0.53158	-1.65292	0.007813	0.017968
GSE2770_IL12_ACT_VS_ACT_CD4_TCELL_6H_DN	158	-0.39608	-1.59101	0.007813	0.025429
GSE9988_ANTI_TREM1_AND_LPS_VS_VEHICLE_TREATED_MONOCYTES_DN	181	-0.51558	-2.0487	0.007828	0.002642
GSE22886_NAIVE_CD4_TCELL_VS_NEUTROPHIL_DN	180	-0.50158	-1.6186	0.007828	0.021888

GSE11818_WT_VS_DICER_KO_TREG_UP	133	-0.467	-1.48254	0.007828	0.046272
GSE41176_UNSTIM_VS_ANTI_IGM_STIM_BCELL_1H_DN	187	-0.52805	-1.99514	0.007843	0.003048
GSE37532_VISCERAL_ADIPOSE_TISSUE_VS_LN_DERIVED_PPARG_KO_TREG_CD4_TCELL_DN	124	-0.41277	-1.66595	0.007843	0.016686
GSE40666_STAT1_KO_VS_STAT4_KO_CD8_TCELL_WITH_IFNA_STIM_90MIN_UP	172	-0.28133	-1.6537	0.007859	0.017907
GSE29618_LAIV_VS_TIV_FLU_VACCINE_DAY7_PDC_DN	181	-0.38457	-1.49772	0.007859	0.042649
GSE28237_EARLY_VS_LATE_GC_BCELL_UP	185	-0.38496	-1.46352	0.007874	0.051144
GSE37301_MULTIPOTENT_PROGENITOR_VS_RAG2_KO_NK_CELL_UP	185	-0.32739	-1.61019	0.00789	0.02293
GSE28449_WT_VS_LRF_KO_GERMINAL_CENTER_BCELL_DN	182	-0.42043	-1.59865	0.00789	0.024468
GSE36527_CD62L_HIGH_VS_CD62L_LOW_TREG_CD69_NEG_KLRG1_NEG_DN	180	-0.41183	-1.60647	0.007905	0.023406
GSE20715_0H_VS_6H_OZONE_LUNG_DN	190	-0.3945	-1.58558	0.007905	0.026276
GSE27786_LIN_NEG_VS_NEUTROPHIL_DN	182	-0.32782	-1.54308	0.007905	0.033258
GSE43955_TH0_VS_TGFB_IL6_TH17_ACT_CD4_TCELL_42H_UP	183	-0.33935	-1.7287	0.007921	0.011599

GSE22601_IMMATURE_CD4_SINGLE_POSITIVE_VS_CD4_SINGLE_POSITIVE_THYMOCYTE_UP	186	-0.36834	-1.91997	0.007937	0.004057
GSE25085_FETAL_BM_VS_ADULT_BM_SP4_THYMIC_IMPLANT_DN	183	-0.47552	-1.87826	0.007937	0.004945
GSE29615_DAY3_VS_DAY7_LAIV_FLU_VACCINE_PBMC_UP	150	-0.44148	-1.56719	0.007937	0.029123
GSE15750_WT_VS_TRAF6KO_DAY6_EFF_CD8_TCELL_DN	181	-0.42925	-1.53395	0.007937	0.034987
GSE3982_NEUTROPHIL_VS_CENT_MEMORY_CD4_TCELL_UP	186	-0.47387	-1.86141	0.007952	0.005413
GSE17974_2.5H_VS_72H_IL4_AND_ANTI_IL12_ACT_CD4_TCELL_UP	180	-0.33793	-1.77467	0.007952	0.008864
GSE25147_UNSTIM_VS_HELIOBACTER_PYLORI_LPS_STIM_MKN45_CELL_DN	156	-0.42845	-1.67197	0.007952	0.016148
GSE37301_COMMON_LYMPHOID_PROGENITOR_VS_GRAN_MONO_PROGENITOR_UP	181	-0.34841	-1.6264	0.007952	0.020996
GSE12001_MIR223_KO_VS_WT_NEUTROPHIL_DN	179	-0.27467	-1.61655	0.007952	0.02217
GSE18893_TCONV_VS_TREG_24H_CULTURE_DN	184	-0.49886	-1.59313	0.007952	0.025172
GSE20366_CD103_POS_VS_NEG_TREG_KLRG1NEG_UP	184	-0.45343	-1.5518	0.007952	0.031686
GSE16385_IFNG_TNF_VS_IL4_STIM_MACROPHAGE_ROSIGLITAZONE_TREATED_UP	158	-0.41397	-1.54582	0.007952	0.032754

GSE6092_UNSTIM_VS_IFNG_STIM_AND_B_BURGDORFERI_INF_ENDOTHELIAL_CELL_DN	181	-0.47214	-1.50557	0.007952	0.040853
GSE4142_PLASMA_CELL_VS_MEMORY_BCELL_DN	177	-0.37928	-1.55458	0.007968	0.031154
GSE36392_MAC_VS_NEUTROPHIL_IL25_TREATED_LUNG_DN	182	-0.42674	-1.48627	0.007968	0.04532
GSE3039_NKT_CELL_VS_ALPHAALPHA_CD8_TCELL_UP	178	-0.4453	-1.74385	0.007984	0.010564
GSE40666_STAT1_KO_VS_STAT4_KO_CD8_TCELL_WITH_IFNA_STIM_90MIN_DN	150	-0.50904	-1.5729	0.007984	0.028185
GSE8515_CTRL_VS_IL6_4H_STIM_MAC_UP	186	-0.32232	-1.54094	0.007984	0.033671
GSE13485_PRE_VS_POST_YF17D_VACCINATION_PBMC_DN	186	-0.58641	-1.84139	0.008	0.006011
GSE20198_IL12_IL18_VS_IFNA_TREATED_ACT_CD4_TCELL_DN	183	-0.29596	-1.64028	0.008	0.019277
GSE17721_PAM3CSK4_VS_GADIQUIMOD_0.5H_BMDC_UP	189	-0.39524	-1.57053	0.008	0.02858
GSE2124_CTRL_VS_LYMPHOTOXIN_BETA_TREATED_MLN_UP	177	-0.45449	-1.51433	0.008	0.039016
GSE29614_CTRL_VS_TIV_FLU_VACCINE_PBMC_2007_UP	145	-0.45145	-1.4092	0.008	0.067919
GSE5589_LPS_AND_IL10_VS_LPS_AND_IL6_STIM_MACROPHAGE_45MIN_DN	183	-0.44593	-1.76787	0.008016	0.009221

GSE17721_POLYIC_VS_CPG_0.5H_BMDC_DN	185	-0.31825	-1.6039	0.008016	0.023772
GSE3920_IFNB_VS_IFNG_TREATED_ENDOTHELIAL_CELL_UP	152	-0.3948	-1.58746	0.008016	0.025992
GSE19772_HCMV_INFL_VS_HCMV_INF_MONOCYTES_AND_PI3K_INHIBITION_UP	188	-0.44743	-1.54717	0.008016	0.032495
GSE19888_ADENOSINE_A3R_INH_VS_TCELL_MEMBRANES_ACT_MAST_CELL_DN	181	-0.41911	-1.5251	0.008016	0.036782
GSE15750_WT_VS_TRAF6KO_DAY10_EFF_CD8_TCELL_DN	184	-0.45369	-1.49537	0.008016	0.043127
GSE6259_CD4_TCELL_VS_CD8_TCELL_DN	164	-0.5423	-1.89592	0.008032	0.004495
GSE19888_ADENOSINE_A3R_INH_PRETREAT_AND_ACT_BY_A3R_VS_TCELL_MEMBRANES_ACT_MAST_CELL_UP	186	-0.64554	-1.78912	0.008032	0.008129
GSE11961_MARGINAL_ZONE_BCELL_VS_PLASMA_CELL_DAY7_DN	168	-0.4069	-1.69474	0.008032	0.01417
GSE2585_CD80_HIGH_VS_LOW_AIRE_KO_MTEC_DN	182	-0.42506	-1.47185	0.008032	0.049118
GSE43955_1H_VS_20H_ACT_CD4_TCELL_WITH_TGFB_IL6_DN	193	-0.49028	-1.70317	0.008048	0.013494
GSE39820_TGFBETA1_VS_TGFBETA3_IN_IL6_IL23A_TREATED_CD4_TCELL_DN	184	-0.38174	-1.59492	0.008048	0.024964
GSE16451_CTRL_VS_WEST_EQUINE_ENC_VIRUS_MATURE_NEURON_CELL_LINE_UP	181	-0.33749	-1.47418	0.008048	0.048506

GSE13485_PRE_VS_POST_YF17D_VACCINATION_PBMC_UP	165	-0.43743	-1.54236	0.008065	0.033401
GSE3982_CTRL_VS_LPS_4H_MAC_DN	190	-0.51268	-1.86419	0.008081	0.005329
GSE17721_PAM3CSK4_VS_GADIQUIMOD_16H_BMDC_DN	179	-0.38113	-1.74741	0.008081	0.010336
GSE17721_POLYIC_VS_GARDIQUIMOD_1H_BMDC_DN	182	-0.35362	-1.74245	0.008081	0.010629
GSE22601_DOUBLE_NEGATIVE_VS_IMMATURE_CD4_SP_THYMOCYTE_UP	178	-0.29894	-1.71935	0.008081	0.012296
GSE7509_DC_VS_MONOCYTE_WITH_FCGRIIB_STIM_DN	187	-0.49037	-1.82578	0.008097	0.006563
GSE3720_LPS_VS_PMA_STIM_VD1_GAMMADELTA_TCELL_UP	136	-0.51639	-1.79824	0.008114	0.007737
GSE32986_GMCSF_VS_GMCSF_AND_CURDLAN_HIGHDOSE_STIM_DC_UP	176	-0.38588	-1.70125	0.008114	0.013629
GSE34006_A2AR_KO_VS_A2AR_AGONIST_TREATED_TREG_UP	190	-0.47816	-1.87576	0.00813	0.005025
GSE11864_CSF1_VS_CSF1_PAM3CYS_IN_MAC_DN	169	-0.37129	-1.69372	0.00813	0.014255
GSE24972_WT_VS_IRF8_KO_MARGINAL_ZONE_SPLEEN_BCELL_UP	177	-0.35674	-1.68369	0.00813	0.015077
GSE16385_UNTREATED_VS_12H_ROSIGLITAZONE_IFNG_TNF_TREATED_MACROPHAGE_DN	168	-0.3822	-1.52804	0.00813	0.036187

GSE17721_CTRL_VS_LPS_4H_BMDC_UP	182	-0.26268	-1.67545	0.008147	0.015827
GSE43955_1H_VS_20H_ACT_CD4_TCELL_WITH_TGFB_IL6_UP	188	-0.31616	-1.64498	0.008147	0.018844
GSE25088_CTRL_VS_IL4_STIM_STAT6_KO_MACROPHAGE_DN	157	-0.3933	-1.56408	0.008147	0.029574
GSE39382_IL3_VS_IL3_IL33_TREATED_MAST_CELL_DN	179	-0.46813	-1.82096	0.008163	0.006774
GSE21670_STAT3_KO_VS_WT_CD4_TCELL_DN	184	-0.47318	-1.79497	0.008163	0.007869
GSE17721_LPS_VS_CPG_8H_BMDC_DN	175	-0.35076	-1.77018	0.008163	0.009102
GSE35825_UNTREATED_VS_IFNG_STIM_MACROPHAGE_UP	186	-0.51197	-1.75331	0.008163	0.009974
GSE13493_DP_VS_CD8POS_THYMOCYTE_DN	181	-0.33794	-1.56555	0.008163	0.029377
GSE16522_MEMORY_VS_NAIVE_CD8_TCELL_UP	173	-0.38241	-1.84735	0.00818	0.005854
GSE17721_CTRL_VS_LPS_8H_BMDC_UP	179	-0.3122	-1.71317	0.00818	0.012691
GSE1112_OT1_CD8AB_VS_HY_CD8AA_THYMOCYTE_RTOC_CULTURE_DN	161	-0.39115	-1.54217	0.00818	0.033434
GSE17721_POLYIC_VS_GARDIQUIMOD_24H_BMDC_DN	185	-0.32901	-1.84946	0.008197	0.005803

GSE8515_IL1_VS_IL6_4H_STIM_MAC_UP	185	-0.50139	-1.77569	0.008214	0.008797
GSE5679_CTRL_VS_PPARG_LIGAND_ROSIGLITAZONE_AND_RARA_AGONIST_AM580_TREATED_DC_DN	183	-0.45871	-1.73617	0.008214	0.011097
GSE1112_OT1_CD8AB_VS_HY_CD8AA_THYMOCYTE_RTOC_CULTURE_UP	138	-0.46686	-1.50249	0.008214	0.04155
GSE13485_CTRL_VS_DAY21_YF17D_VACCINE_PBMC_DN	139	-0.46642	-1.4717	0.008214	0.049121
GSE27786_NKCELL_VS_NKTCELL_UP	186	-0.38084	-1.83565	0.00823	0.00621
GSE17721_0.5H_VS_12H_CPG_BMDC_DN	184	-0.40279	-1.77863	0.008247	0.008669
GSE22443_IL2_VS_IL12_TREATED_ACT_CD8_TCELL_DN	190	-0.37815	-1.72714	0.008247	0.011707
GSE3982_MAC_VS_TH2_DN	183	-0.3954	-1.68429	0.008264	0.015035
GSE2706_2H_VS_8H_LPS_STIM_DC_DN	167	-0.5643	-1.82253	0.008282	0.006699
GSE27786_CD4_TCELL_VS_NKCELL_DN	186	-0.37456	-1.82008	0.008282	0.006814
GSE17721_LPS_VS_GARDIQUIMOD_8H_BMDC_UP	185	-0.36513	-1.6398	0.008282	0.01934
GSE11864_UNTREATED_VS_CSF1_IFNG_PAM3CYS_IN_MAC_DN	176	-0.39338	-1.86263	0.008299	0.005378

GSE37605_FOXP3_FUSION_GFP_VS_IRES_GFP_TREG_C57BL6_DN	165	-0.51579	-1.74238	0.008299	0.010629
GSE36888_UNTREATED_VS_IL2_TREATED_TCELL_6H_DN	179	-0.34911	-1.69223	0.008299	0.014339
GSE8384_CTRL_VS_B_ABORTUS_4H_MAC_CELL_LINE_UP	180	-0.33141	-1.68415	0.008299	0.015031
GSE45365_CD8A_DC_VS_CD11B_DC_IFNAR_KO_UP	169	-0.42279	-1.60121	0.008299	0.024168
GSE369_SOCS3_KO_VS_WT_LIVER_POST_IL6_INJECTION_UP	178	-0.32397	-1.53404	0.008299	0.034979
GSE6259_FLT3L_INDUCED_DEC205_POS_DC_VS_CD8_TCELL_DN	164	-0.54686	-1.89179	0.008316	0.004579
GSE360_CTRL_VS_L_DONOVANI_DC_DN	189	-0.46115	-1.58935	0.008333	0.025677
GSE17721_0.5H_VS_8H_GARDIQUIMOD_BMDC_UP	176	-0.29174	-1.65945	0.008351	0.017318
GSE9650_GP33_VS_GP276_LCMV_SPECIFIC_EXHAUSTED_CD8_TCELL_UP	193	-0.50165	-1.6982	0.008368	0.013901
GSE17721_CTRL_VS_CPG_1H_BMDC_DN	184	-0.42788	-1.52571	0.008386	0.036673
GSE29949_DC_BRAIN_VS_MONOCYTE_BONE_MARROW_UP	183	-0.37016	-1.78721	0.008403	0.008219
GSE17721_PAM3CSK4_VS_GADIQUIMOD_6H_BMDC_UP	181	-0.29653	-1.72117	0.008421	0.012187

GSE37533_UNTREATED_VS_PIOGLIZATONE_TREATED_CD4_TCELL_FOXP3_TRASDUCED_CD4_TCELL_UP	147	-0.37716	-1.58536	0.008421	0.026292
GSE20152_SPHK1_KO_VS_WT_HTNFA_OVERXPRESS_ANKLE_UP	183	-0.32944	-1.54915	0.008439	0.032145
GSE15930_STIM_VS_STIM_AND_IL12_24H_CD8_T_CELL_UP	180	-0.31612	-1.63562	0.008457	0.019895
GSE20715_WT_VS_TLR4_KO_24H_OZONE_LUNG_DN	189	-0.35813	-1.60922	0.009488	0.023041
GSE42724_MEMORY_VS_B1_BCELL_UP	175	-0.30333	-1.62341	0.009524	0.021368
GSE17974_0.5H_VS_72H_UNTREATED_IN_VITRO_CD4_TCELL_UP	159	-0.36946	-1.77425	0.009579	0.008888
GSE17721_0.5H_VS_4H_POLYIC_BMDC_DN	181	-0.38656	-1.82798	0.009671	0.006516
GSE9960_HEALTHY_VS_GRAM_POS_SEPSIS_PBMC_UP	163	-0.32325	-1.64404	0.00969	0.018937
GSE2706_UNSTIM_VS_2H_R848_DC_UP	162	-0.32991	-1.7174	0.009709	0.012432
GSE41867_DAY8_VS_DAY15_LCMV_CLONE13_EFFECTOR_CD8_TCELL_UP	179	-0.36072	-1.80102	0.009728	0.007633
GSE36888_UNTREATED_VS_IL2_TREATED_TCELL_6H_UP	166	-0.56478	-1.77441	0.009728	0.008882
GSE29618_MONOCYTE_VS_PDC_UP	185	-0.64338	-1.8463	0.009747	0.005873

GSE36888_STAT5_AB_KNOCKIN_VS_WT_TCELL_IL2_TREATED_17H_DN	181	-0.50524	-1.8894	0.009766	0.004624
GSE17721_CPG_VS_GARDIQUIMOD_4H_BMDC_DN	180	-0.29389	-1.65063	0.009766	0.018168
GSE20366_EX_VIVO_VS_HOMEOSTATIC_CONVERSION_TREG_UP	188	-0.29861	-1.51332	0.009766	0.039186
GSE17974_1H_VS_72H_UNTREATED_IN_VITRO_CD4_TCELL_UP	171	-0.35232	-1.81419	0.009862	0.007057
GSE10273_HIGH_IL7_VS_HIGH_IL7_AND_IRF4_IN_IRF4_8_NULL_PRE_BCELL_DN	182	-0.4356	-1.72632	0.009862	0.011785
GSE22886_NAIVE_TCELL_VS_NKCELL_DN	181	-0.43727	-1.90472	0.009881	0.004345
GSE2197_CPG_DNA_VS_UNTREATED_IN_DC_DN	179	-0.29966	-1.54059	0.009881	0.033734
GSE2770_IL12_VS_IL4_TREATED_ACT_CD4_TCELL_2H_UP	137	-0.38293	-1.67987	0.009901	0.015446
GSE4811_CLASSSICALY_ACTIVATED_VS_TYPE_2_ACTIVATED_MACROPHAGE_DN	180	-0.49135	-1.5441	0.009921	0.033065
GSE37301_CD4_TCELL_VS_RAG2_KO_NK_CELL_DN	171	-0.34986	-1.75288	0.00994	0.00999
GSE22140_GERMFREE_VS_SPF_ARTHRITIC_MOUSE_CD4_TCELL_UP	190	-0.45956	-1.7609	0.00996	0.009575
GSE17721_PAM3CSK4_VS_CPG_4H_BMDC_UP	172	-0.26768	-1.70055	0.00996	0.013686

GSE36891_UNSTIM_VS_POLYIC_TLR3_STIM_PERITONEAL_MACROPHAGE_DN	183	-0.42972	-1.77881	0.00998	0.008666
GSE19512_NAUTRAL_VS_INDUCED_TREG_DN	187	-0.42539	-1.66702	0.00998	0.016602
GSE22033_WT_VS_PPARG_KO_MEF_UP	175	-0.40763	-1.59732	0.00998	0.024623
GSE37534_UNTREATED_VS_ROSIGLITAZONE_TREATED_CD4_TCELL_PPARG1_AND_FOXP3_TRASDUCED_UP	179	-0.38669	-1.54805	0.00998	0.032349
GSE7509_DC_VS_MONOCYTE_DN	184	-0.47464	-1.84646	0.01	0.005877
GSE3982_DC_VS_EFF_MEMORY_CD4_TCELL_UP	185	-0.47181	-1.83212	0.01	0.006326
GSE18791_UNSTIM_VS_NEWCATSLE_VIRUS_DC_6H_DN	171	-0.58471	-1.78886	0.01	0.008135
GSE33162_HDAC3_KO_VS_HDAC3_KO_4H_LPS_STIM_MACROPHAGE_UP	182	-0.29735	-1.8087	0.01002	0.00732
GSE9960_GRAM_NEG_VS_GRAM_POS_SEPSIS_PBMC_UP	173	-0.45865	-1.7649	0.01002	0.009361
GSE1740_MCSF_VS_MCSF_AND_IFNG_DAY2_DERIVED_MACROPHAGE_WITH_IFNA_STIM_UP	183	-0.2949	-1.73157	0.01002	0.011416
GSE5589_IL6_KO_VS_IL10_KO_LPS_STIM_MACROPHAGE_45MIN_DN	179	-0.33225	-1.61334	0.01002	0.022545
GSE10325_MYELOID_VS_LUPUS_MYELOID_UP	184	-0.48552	-1.54156	0.01002	0.03354

GSE37301_MULTIPOTENT_PROGENITOR_VS_GRAN_MONO_PROGENITOR_DN	187	-0.42694	-1.73485	0.01004	0.011184
GSE24142_EARLY_THYMIC_PROGENITOR_VS_DN2_THYMOCYTE_ADULT_DN	187	-0.47556	-1.63927	0.01004	0.0194
GSE2706_UNSTIM_VS_8H_LPS_AND_R848_DC_UP	175	-0.30541	-1.86506	0.01006	0.005319
GSE15330_LYMPHOID_MULTIPOTENT_VS_MEGAKARYOCYTE_ERYTHROID_PROGENITOR_IKAROS_KO_DN	170	-0.55921	-1.81461	0.01006	0.007029
GSE5542_IFNG_VS_IFNA_AND_IFNG_TREATED_EPITHELIAL_CELLS_6H_DN	176	-0.29139	-1.63979	0.01006	0.019338
GSE9988_LPS_VS_LOW_LPS_MONOCYTE_DN	149	-0.435	-1.45823	0.01006	0.052577
GSE2706_2H_VS_8H_R848_AND_LPS_STIM_DC_DN	175	-0.50196	-1.75389	0.010081	0.009946
GSE17721_CPG_VS_GARDIQUIMOD_2H_BMDC_DN	187	-0.2904	-1.73992	0.010081	0.010799
GSE34392_ST2_KO_VS_WT_DAY8_LCMV_EFFECTOR_CD8_TCELL_DN	186	-0.54416	-1.78755	0.010101	0.00821
GSE9509_LPS_VS_LPS_AND_IL10_STIM_IL10_KO_MACROPHAGE_10MIN_UP	187	-0.50022	-1.65326	0.010101	0.017936
GSE17721_CTRL_VS_CPG_1H_BMDC_UP	183	-0.27757	-1.6465	0.010101	0.018668
GSE5960_TH1_VS_ANERGIC_TH1_UP	184	-0.30733	-1.57575	0.010101	0.027762

GSE27786_LSK_VS_ERYTHROBLAST_DN	169	-0.42698	-1.46046	0.010101	0.051975
GSE46606_UNSTIM_VS_CD40L_IL2_IL5_1DAY_STIMULATED_IRF4MID_SORTED_BCELL_UP	183	-0.38247	-1.8292	0.010121	0.006449
GSE14413_UNSTIM_VS_IFNB_STIM_NIH3T3_CELLS_UP	134	-0.35765	-1.62869	0.010121	0.020667
GSE10500_ARTHRITIC_SYNOVIAL_FLUID_VS_HEALTHY_MACROPHAGE_DN	133	-0.40455	-1.62645	0.010121	0.020995
GSE19923_WT_VS_HEB_KO_DP_THYMOCYTE_DN	181	-0.33175	-1.67201	0.010142	0.016152
GSE41978_WT_VS_ID2_KO_KLRG1_LOW_EFFECTOR_CD8_TCELL_DN	188	-0.43107	-1.64108	0.010142	0.019178
GSE7548_NAIVE_VS_DAY28_PCC_IMMUNIZATION_CD4_TCELL_DN	183	-0.40742	-1.51873	0.010142	0.038075
GSE21360_PRIMARY_VS_TERTIARY_MEMORY_CD8_TCELL_DN	187	-0.56571	-1.85716	0.010163	0.005542
GSE37533_PPARG1_FOXP3_VS_FOXP3_TRANSDUCED_CD4_TCELL_PIOGLITAZONE_TREATED_UP	191	-0.49556	-1.84192	0.010163	0.005986
GSE5589_LPS_AND_IL10_VS_LPS_AND_IL6_STIM_MACROPHAGE_45MIN_UP	181	-0.31306	-1.67201	0.010163	0.016157
GSE1925_CTRL_VS_24H_IFNG_STIM_MACROPHAGE_DN	168	-0.3565	-1.64733	0.010163	0.018571
GSE32034_UNTREATED_VS_ROSIGLIZATONE_TREATED_LY6C_HIGH_MONOCYTE_UP	188	-0.3764	-1.59375	0.010183	0.025086

GSE26290_CTRL_VS_AKT_INHIBITOR_TREATED_ANTI_CD3_AND_IL2_STIM_CD8_TCELL_UP	182	-0.45029	-1.53045	0.010183	0.035725
GSE37416_0H_VS_12H_F_TULARENSIS_LVS_NEUTROPHIL_DN	180	-0.35689	-1.85341	0.010204	0.005674
GSE14000_UNSTIM_VS_16H_LPS_DC_TRANSLATED_RNA_DN	174	-0.5716	-1.76153	0.010204	0.009521
GSE17721_12H_VS_24H_GARDIQUIMOD_BMDC_DN	175	-0.31403	-1.67889	0.010225	0.015533
GSE5589_UNSTIM_VS_180MIN_LPS_STIM_MACROPHAGE_UP	186	-0.29105	-1.58251	0.010225	0.026741
GSE37605_TREG_VS_TCONV_C57BL6_FOXP3_IRES_GFP_UP	117	-0.49785	-1.47049	0.010225	0.049368
GSE7852_THYMUS_VS_FAT_TREG_UP	180	-0.35906	-1.89135	0.010246	0.004589
GSE17721_PAM3CSK4_VS_CPG_0.5H_BMDC_UP	184	-0.33778	-1.57905	0.010246	0.027276
GSE36891_UNSTIM_VS_PAM_TLR2_STIM_PERITONEAL_MACROPHAGE_UP	169	-0.29444	-1.74294	0.010267	0.010622
GSE5960_TH1_VS_ANERGIC_TH1_DN	189	-0.33911	-1.69363	0.010267	0.014254
GSE25123_IL4_VS_IL4_AND_ROSIGLITAZONE_STIM_PPARG_KO_MACROPHAGE_DAY10_DN	174	-0.51198	-1.74147	0.010288	0.010699
GSE17721_CPG_VS_GARDIQUIMOD_0.5H_BMDC_DN	187	-0.30682	-1.59946	0.010288	0.02435

GSE339_EX_VIVO_VS_IN_CULTURE_CD4CD8DN_DC_DN	184	-0.35741	-1.78805	0.010331	0.008177
GSE3982_DC_VS_NEUTROPHIL_LPS_STIM_UP	184	-0.32562	-1.76735	0.010331	0.009247
GSE13484_UNSTIM_VS_12H_YF17D_VACCINE_STIM_PBMC_DN	182	-0.40401	-1.74555	0.010331	0.010467
GSE9946_IMMATURE_VS_LISTERIA_INF_MATURE_DC_DN	121	-0.42234	-1.55179	0.010331	0.031679
GSE12707_AT16L1_HYPOMORPH_VS_WT_THYMUS_DN	125	-0.44193	-1.48767	0.010373	0.044963
GSE24726_WT_VS_E2_22_KO_PDC_DAY4_POST_DELETION_DN	183	-0.42426	-1.85015	0.010395	0.005773
GSE17721_CTRL_VS_POLYIC_24H_BMDC_UP	182	-0.35261	-1.71985	0.010417	0.012263
GSE3720_VD1_VS_VD2_GAMMADELTA_TCELL_WITH_PMA_STIM_UP	180	-0.30558	-1.58021	0.010417	0.0271
GSE9946_IMMATURE_VS_PROSTAGLANDINE2_TREATED_MATURE_DC_DN	137	-0.41651	-1.44994	0.010417	0.055028
GSE19888_NO_PRETREAT_VS_ADENOSINE_A3R_INHIBITOR_PRETREATED_MAST_CELL_TCELL_MEMBRANES_ACT_DN	182	-0.39429	-1.86363	0.010438	0.005345
GSE22103_UNSTIM_VS_LPS_STIM_NEUTROPHIL_UP	183	-0.30508	-1.71218	0.010504	0.012764
GSE36392_TYPE_2_MYELOID_VS_MAC_IL25_TREATED_LUNG_UP	186	-0.46325	-1.45437	0.010504	0.053735

GSE9960_HEALTHY_VS_SEPSIS_PBMC_UP	170	-0.38916	-1.51933	0.010526	0.037944
GSE7831_1H_VS_4H_CPG_STIM_PDC_DN	183	-0.43755	-1.57617	0.010753	0.027694
GSE43863_TFH_VS_LY6C_INT_CXCR5POS_EFFECTOR_CD4_TCELL_UP	188	-0.32199	-1.63439	0.010776	0.020027
GSE37301_MULTIPOTENT_PROGENITOR_VS_GRAN_MONO_PROGENITOR_UP	184	-0.38523	-1.58947	0.010846	0.025664
GSE9946_IMMATURE_VS_LISTERIA_INF_MATURE_DC_UP	128	-0.45475	-1.52117	0.011194	0.037568
GSE23321_CD8_STEM_CELL_MEMORY_VS_NAIVE_CD8_TCELL_DN	165	-0.29612	-1.73805	0.011429	0.010921
GSE29164_CD8_TCELL_VS_CD8_TCELL_AND_IL12_TREATED_MELANOMA_DAY7_UP	168	-0.3723	-1.83709	0.01145	0.00614
GSE40274_SATB1_VS_FOXP3_AND_SATB1_TRANSDUCED_ACTIVATED_CD4_TCELL_DN	113	-0.39733	-1.48468	0.011494	0.045744
GSE17721_CTRL_VS_PAM3CSK4_8H_BMDC_UP	181	-0.29172	-1.62981	0.011628	0.02055
GSE18893_TCONV_VS_TREG_2H_TNF_STIM_UP	180	-0.34601	-1.50999	0.01165	0.039894
GSE18791_CTRL_VS_NEWCASTLE_VIRUS_DC_10H_DN	177	-0.56781	-1.84664	0.011696	0.005883
GSE20198_IL12_VS_IFNA_TREATED_ACT_CD4_TCELL_DN	178	-0.31833	-1.66962	0.011696	0.016376

GSE25123_IL4_VS_IL4_AND_ROSIGLITAZONE_STIM_MACROPHAGE_DAY10_DN	170	-0.35543	-1.54356	0.011696	0.033168
GSE43955_TGFB_IL6_VS_TGFB_IL6_IL23_TH17_ACT_CD4_TCELL_52H_UP	183	-0.36868	-1.58341	0.011719	0.026617
GSE9946_LISTERIA_INF_MATURE_VS_PROSTAGLANDINE2_TREATED_MATURE_DC_UP	130	-0.39302	-1.55035	0.011742	0.0319
GSE28130_ACTIVATED_VS_INDUCEED_TREG_DN	187	-0.4139	-1.50378	0.011742	0.04124
GSE18791_UNSTIM_VS_NEWCATSLE_VIRUS_DC_10H_DN	171	-0.57155	-1.78421	0.011765	0.008388
GSE25123_WT_VS_PPARG_KO_MACROPHAGE_UP	173	-0.55939	-1.78008	0.011765	0.008628
GSE2770_TGFB_AND_IL4_ACT_VS_ACT_CD4_TCELL_2H_DN	179	-0.50517	-1.86735	0.011788	0.00525
GSE3920_UNTREATED_VS_IFNG_TREATED_ENDOTHELIAL_CELL_UP	142	-0.44958	-1.57458	0.011788	0.027908
GSE41176_WT_VS_TAK1_KO_ANTI_IGM_STIM_BCELL_6H_DN	191	-0.49238	-1.97898	0.011811	0.003309
GSE22601_IMMATURE_CD4_SINGLE_POSITIVE_VS_CD8_SINGLE_POSITIVE_THYMOCYTE_DN	183	-0.4969	-1.88051	0.011811	0.004888
GSE10325_BCELL_VS_MYELOID_DN	187	-0.61449	-1.75578	0.011811	0.009833
GSE21678_WT_VS_FOXO1_FOXO3_KO_TREG_UP	166	-0.43726	-1.7198	0.011834	0.012264

GSE2706_UNSTIM_VS_2H_LPS_DC_UP	154	-0.4084	-1.65908	0.011834	0.017354
GSE26351_UNSTIM_VS_BMP_PATHWAY_STIM_HEMATOPOIETIC_PROGENITORS_DN	168	-0.36553	-1.53005	0.011834	0.035782
GSE22140_GERMFREE_VS_SPF_ARTHRITIC_MOUSE_CD4_TCELL_DN	183	-0.54047	-1.94065	0.011858	0.003732
GSE13485_DAY7_VS_DAY21_YF17D_VACCINE_PBMC_UP	183	-0.47844	-1.82395	0.011858	0.006647
GSE1925_CTRL_VS_IFNG_PRIMED_MACROPHAGE_3H_IFNG_STIM_UP	163	-0.29971	-1.74417	0.011858	0.010558
GSE5589_LPS_AND_IL10_VS_LPS_AND_IL6_STIM_IL6_KO_MACROPHAGE_45MIN_DN	181	-0.30629	-1.64415	0.011858	0.018938
GSE17186_BLOOD_VS_CORD_BLOOD_CD21LOW_TRANSITIONAL_BCELL_DN	187	-0.33338	-1.68945	0.011881	0.014555
GSE27786_BCELL_VS_CD4_TCELL_UP	177	-0.2889	-1.62191	0.011881	0.021569
GSE2405_HEAT_KILLED_LYSATE_VS_LIVE_A_PHAGOCYTOPHILUM_STIM_NEUTROPHIL_24H_UP	185	-0.47241	-1.73481	0.011905	0.011181
GSE20151_CTRL_VS_FUSOBACT_NUCLEATUM_NEUTROPHIL_UP	182	-0.40301	-1.53195	0.011905	0.035375
GSE24634_TEFF_VS_TCONV_DAY7_IN_CULTURE_DN	188	-0.55229	-1.86937	0.011928	0.005202
GSE24634_TREG_VS_TCONV_POST_DAY3_IL4_CONVERSION_DN	188	-0.56658	-1.86794	0.011928	0.005235

GSE37416_12H_VS_24H_F_TULARENSIS_LVS_NEUTROPHIL_DN	172	-0.31918	-1.73088	0.011928	0.011454
GSE40274_FOXP3_VS_FOXP3_AND_SATB1_TRANSDUCED_ACTIVATED_CD4_TCELL_UP	123	-0.42912	-1.36835	0.011928	0.082787
GSE25890_CTRL_VS_IL33_IL7_TREATED_NUOCYTES_DN	188	-0.29774	-1.70787	0.011952	0.013077
GSE21546_SAP1A_KO_VS_SAP1A_KO_AND_ELK1_KO_DP_THYMOCYTES_UP	179	-0.38352	-1.67007	0.011952	0.016332
GSE41176_UNSTIM_VS_ANTI_IGM_STIM_TAK1_KO_BCELL_1H_DN	180	-0.4142	-1.54573	0.011952	0.032766
GSE21380_NON_TFH_VS_GERMINAL_CENTER_TFH_CD4_TCELL_UP	182	-0.40679	-1.50628	0.011952	0.040693
GSE16450_CTRL_VS_IFNA_12H_STIM_IMMATURE_NEURON_CELL_LINE_DN	157	-0.40149	-1.49864	0.011952	0.042421
GSE17721_0.5H_VS_24H_LPS_BMDC_UP	181	-0.36405	-1.59234	0.011976	0.02523
GSE9006_1MONTH_VS_4MONTH_POST_TYPE_1_DIABETES_DX_PBMC_DN	177	-0.40026	-1.48797	0.011976	0.044888
GSE13887_RESTING_VS_ACT_CD4_TCELL_UP	92	-0.44219	-1.46512	0.011976	0.050794
GSE2706_UNSTIM_VS_2H_LPS_AND_R848_DC_DN	172	-0.59438	-1.74693	0.012	0.010379
GSE5589_LPS_VS_LPS_AND_IL6_STIM_IL10_KO_MACROPHAGE_45MIN_UP	182	-0.44338	-1.48141	0.012	0.046582

GSE16450_CTRL_VS_IFNA_12H_STIM_MATURE_NEURON_CELL_LINE_DN	178	-0.39003	-1.82446	0.012024	0.00663
GSE43956_WT_VS_SGK1_KO_TH17_DIFFERENTIATED_CD4_TCELL_DN	184	-0.39538	-1.78505	0.012024	0.008332
GSE3920_IFNB_VS_IFNG_TREATED_ENDOTHELIAL_CELL_DN	166	-0.32174	-1.77198	0.012024	0.008999
GSE41176_WT_VS_TAK1_KO_ANTI_IGM_STIM_BCELL_6H_UP	185	-0.40894	-1.59738	0.012024	0.024617
GSE41978_WT_VS_ID2_KO_KLRG1_LOW_EFFECTOR_CD8_TCELL_UP	190	-0.44953	-1.50717	0.012024	0.04053
GSE34156_UNTREATED_VS_24H_NOD2_LIGAND_TREATED_MONOCYTE_DN	185	-0.56441	-1.81158	0.012048	0.007163
GSE7831_UNSTIM_VS_CPG_STIM_PDC_4H_UP	184	-0.42705	-1.79753	0.012048	0.00776
GSE17721_CTRL_VS_LPS_2H_BMDC_UP	180	-0.31084	-1.65223	0.012048	0.018034
GSE15659_NAIVE_CD4_TCELL_VS_ACTIVATED_TREG_DN	181	-0.34972	-1.49056	0.012048	0.044276
GSE37416_12H_VS_24H_F_TULARENSIS_LVS_NEUTROPHIL_UP	176	-0.46285	-1.95775	0.012072	0.003437
GSE22935_WT_VS_MYD88_KO_MACROPHAGE_UP	188	-0.55846	-1.7943	0.012072	0.007906
GSE39820_CTRL_VS_TGFBETA1_IL6_CD4_TCELL_UP	186	-0.44004	-1.77007	0.012072	0.009109

GSE43955_TH0_VS_TGFB_IL6_TH17_ACT_CD4_TCELL_4H_UP	188	-0.42277	-1.73305	0.012072	0.011302
GSE29618_PRE_VS_DAY7_POST_TIV_FLU_VACCINE_MONOCYTE_UP	174	-0.41751	-1.58858	0.012072	0.025794
GSE21063_WT_VS_NFATC1_KO_3H_ANTI_IGM_STIM_BCELL_DN	181	-0.56919	-1.96802	0.012097	0.00337
GSE34156_NOD2_LIGAND_VS_TLR1_TLR2_LIGAND_6H_TREATED_MONOCYTE_UP	190	-0.45372	-1.84174	0.012097	0.005993
GSE44649_WT_VS_MIR155_KO_ACTIVATED_CD8_TCELL_UP	183	-0.43898	-1.83415	0.012097	0.006251
GSE360_HIGH_DOSE_B_MALAYI_VS_M_TUBERCULOSIS_DC_DN	189	-0.5213	-1.77169	0.012097	0.009021
GSE13484_3H_UNSTIM_VS_YF17D_VACCINE_STIM_PBMC_DN	189	-0.50304	-1.75553	0.012097	0.009845
GSE43955_1H_VS_60H_ACT_CD4_TCELL_UP	186	-0.44294	-1.83789	0.012121	0.006101
GSE36888_UNTREATED_VS_IL2_TREATED_STAT5_AB_KNOCKIN_TCELL_17H_UP	160	-0.46876	-1.65389	0.012121	0.017886
GSE28737_WT_VS_BCL6_HET_MARGINAL_ZONE_BCELL_DN	175	-0.43742	-1.63667	0.012121	0.019762
GSE24634_TREG_VS_TCONV_POST_DAY10_IL4_CONVERSION_DN	193	-0.59946	-1.79446	0.012146	0.0079
GSE22935_UNSTIM_VS_24H_MBOVIS_BCG_STIM_MACROPHAGE_DN	185	-0.37672	-1.63148	0.012146	0.020376

GSE29618_BCELL_VS_MDC_DN	187	-0.50846	-1.92651	0.01217	0.003944
GSE30971_WBP7_HET_VS_KO_MACROPHAGE_2H_LPS_STIM_UP	177	-0.54382	-1.84723	0.01217	0.005856
GSE42021_CD24INT_VS_CD24LOW_TCONV_THYMUS_DN	183	-0.44334	-1.7137	0.01217	0.012666
GSE38696_LIGHT_ZONE_VS_DARK_ZONE_BCELL_DN	130	-0.40981	-1.47161	0.01217	0.049105
GSE39820_TGFBETA1_IL6_VS_TGFBETA1_IL6_IL23A_TREATED_CD4_TCELL_DN	184	-0.39534	-1.68263	0.012195	0.015176
GSE39820_CTRL_VS_TGFBETA1_IL6_IL23A_CD4_TCELL_DN	180	-0.32918	-1.65262	0.012195	0.017999
GSE43955_TH0_VS_TGFB_IL6_TH17_ACT_CD4_TCELL_60H_UP	193	-0.35377	-1.48467	0.012195	0.045734
GSE22589_SIV_VS_HIV_AND_SIV_INFECTED_DC_DN	178	-0.37032	-1.4464	0.012195	0.055969
GSE10273_HIGH_VS_LOW_IL7_TREATED_IRF4_8_NULL_PRE_BCELL_UP	181	-0.37647	-1.72591	0.01222	0.011818
GSE30971_WBP7_HET_VS_KO_MACROPHAGE_2H_LPS_STIM_DN	179	-0.5232	-1.68171	0.01222	0.015258
GSE17721_CTRL_VS_GARDIQUIMOD_2H_BMDC_UP	175	-0.34443	-1.61143	0.01222	0.022817
GSE36826_NORMAL_VS_STAPH_AUREUS_INF_SKIN_UP	179	-0.41258	-1.8117	0.012245	0.007161

GSE3982_MAC_VS_EFF_MEMORY_CD4_TCELL_UP	180	-0.40915	-1.81042	0.012245	0.007223
GSE32986_CURDLAN_LOWDOSE_VS_CURDLAN_HIGHDOSE_STIM_DC_DN	183	-0.5038	-1.80668	0.012245	0.007394
GSE3982_MAC_VS_NKCELL_UP	184	-0.44224	-1.7742	0.012245	0.008887
GSE5589_UNSTIM_VS_180MIN_LPS_AND_IL10_STIM_MACROPHAGE_UP	178	-0.29332	-1.69273	0.012245	0.014317
GSE29949_CD8_NEG_DC_SPLEEN_VS_CD8_POS_DC_SPLEEN_DN	183	-0.39689	-1.57264	0.012245	0.028225
GSE43700_UNTREATED_VS_IL10_TREATED_PBMC_DN	192	-0.36496	-1.56059	0.012245	0.030139
GSE21546_UNSTIM_VS_ANTI_CD3_STIM_DP_THYMOCYTES_UP	170	-0.40234	-1.59753	0.01227	0.0246
GSE13306_RA_VS_UNTREATED_MEM_CD4_TCELL_DN	172	-0.42012	-1.59403	0.01227	0.025062
GSE12003_4D_VS_8D_CULTURE_BM_PROGENITOR_UP	155	-0.32347	-1.56445	0.01227	0.029524
GSE26030_TH1_VS_TH17_RESTIMULATED_DAY5_POST_POLARIZATION_UP	189	-0.40625	-1.69716	0.012295	0.013983
GSE9960_HEALTHY_VS_GRAM_NEG_AND_POS_SEPSIS_PBMC_UP	152	-0.45465	-1.43878	0.012295	0.058438
GSE26727_WT_VS_KLF2_KO_LPS_STIM_MACROPHAGE_DN	188	-0.38093	-1.74352	0.01232	0.010579

GSE360_L_DONOVANI_VS_B_MALAYI_LOW_DOSE_DC_UP	189	-0.47748	-1.67327	0.01232	0.016053
GSE17721_12H_VS_24H_GARDIQUIMOD_BMDC_UP	186	-0.42866	-1.66194	0.01232	0.017062
GSE43955_TH0_VS_TGFB_IL6_TH17_ACT_CD4_TCELL_52H_UP	184	-0.37576	-1.54711	0.01232	0.032498
GSE43955_TH0_VS_TGFB_IL6_TH17_ACT_CD4_TCELL_30H_UP	189	-0.42619	-1.57017	0.012346	0.028628
GSE19401_PAM2CSK4_VS_RETINOIC_ACID_STIM_FOLLICULAR_DC_UP	180	-0.31711	-1.56217	0.012346	0.029875
GSE369_PRE_VS_POST_IL6_INJECTION_IFNG_WT_LIVER_UP	172	-0.39293	-1.47175	0.012397	0.049132
GSE19198_CTRL_VS_IL21_TREATED_TCELL_1H_DN	179	-0.44203	-1.77103	0.012422	0.009053
GSE557_WT_VS_I_AB_KO_DC_DN	175	-0.31587	-1.61117	0.012422	0.022836
GSE37416_0H_VS_6H_F_TULARENSIS_LVS_NEUTROPHIL_DN	185	-0.39096	-1.84583	0.012448	0.005885
GSE26912_TUMORICIDAL_VS_CTRL_MACROPHAGE_UP	188	-0.35793	-1.55318	0.0125	0.031413
GSE25088_CTRL_VS_IL4_AND_ROSIGLITAZONE_STIM_STAT6_KO_MACROPHAGE_DN	161	-0.38512	-1.66571	0.012552	0.016701
GSE24210_TCONV_VS_TREG_DN	173	-0.33545	-1.49711	0.012552	0.042757

GSE36392_EOSINOPHIL_VS_NEUTROPHIL_IL25_TREATED_LUNG_DN	183	-0.29634	-1.68487	0.012632	0.014972
GSE25085_FETAL_LIVER_VS_FETAL_BM_SP4_THYMIC_IMPLANT_UP	178	-0.37435	-1.75006	0.012658	0.010148
GSE12198_NK_VS_NK_ACT_EXPANSION_SYSTEM_DERIVED_NK_CELL_DN	184	-0.29481	-1.66121	0.012658	0.017115
GSE14769_20MIN_VS_360MIN_LPS_BMDM_DN	184	-0.38484	-1.66278	0.012876	0.01699
GSE21927_C26GM_VS_4T1_TUMOR_MONOCYTE_BALBC_UP	175	-0.34708	-1.56557	0.013233	0.029383
GSE21063_CTRL_VS_ANTI_IGM_STIM_BCELL_NFATC1_KO_8H_UP	175	-0.40509	-1.77008	0.013436	0.009112
GSE41978_WT_VS_ID2_KO_AND_BIM_KO_KLRG1_LOW_EFFECTOR_CD8_TCELL_DN	183	-0.41317	-1.56987	0.013436	0.028667
GSE7509_DC_VS_MONOCYTE_WITH_FCGRIIB_STIM_UP	180	-0.25652	-1.66942	0.013462	0.016395
GSE30971_WBP7_HET_VS_KO_MACROPHAGE_UP	182	-0.38964	-1.59873	0.013487	0.024462
GSE6259_DEC205_POS_DC_VS_BCELL_DN	163	-0.45818	-1.90102	0.01354	0.004362
GSE40274_EOS_VS_FOXP3_AND_EOS_TRANSDUCED_ACTIVATED_CD4_TCELL_UP	144	-0.27511	-1.53197	0.013592	0.035383
GSE26488_CTRL_VS_PEPTIDE_INJECTION_HDAC7_DELTAP_TG_OT2_THYMOCYTE_UP	194	-0.44235	-1.50316	0.013592	0.041393

GSE17721_CTRL_VS_LPS_1H_BMDC_UP	17<br>0	-<br>0.245<br>99	-<br>1.489<br>44	0.0135<br>92	0.0445<br>22
GSE30083_SP3_VS_SP4_THYMOCYTE_UP	18<br>2	-<br>0.415<br>61	-<br>1.477<br>91	0.0136<br>19	0.0475<br>57
GSE17721_LPS_VS_GARDIQUIMOD_8H_BMDC_DN	18<br>7	-<br>0.367<br>83	-<br>1.748<br>45	0.0136<br>45	0.0102<br>58
GSE18893_TCONV_VS_TREG_24H_TNF_STIM_DN	17<br>1	-<br>0.328<br>11	-<br>1.618<br>82	0.0136<br>45	0.0218<br>7
GSE36527_CD69_NEG_VS_POS_TREG_CD62L_LOS_KLRG1_NEG_DN	18<br>0	-<br>0.358<br>74	-<br>1.846<br>23	0.0137<br>52	0.0058<br>74
GSE41867_DAY8_VS_DAY15_LCMV_ARMSTRONG_EFFECTOR_CD8_TCELL_UP	14<br>2	-<br>0.444<br>15	-<br>1.413<br>97	0.0137<br>52	0.0663<br>71
GSE45365_HEALTHY_VS_MCMV_INFECTION_CD11B_DC_UP	15<br>7	-<br>0.412<br>6	-<br>1.712<br>3	0.0137<br>8	0.0127<br>59
GSE15324_ELF4_KO_VS_WT_NAIVE_CD8_TCELL_DN	17<br>8	-<br>0.393<br>42	-<br>1.386<br>44	0.0137<br>8	0.0759<br>8
GSE18791_CTRL_VS_NEWCASTLE_VIRUS_DC_8H_DN	17<br>7	-<br>0.571<br>17	-<br>1.768<br>3	0.0138<br>34	0.0091<br>89
GSE23505_IL6_IL1_VS_IL6_IL1_IL23_TREATED_CD4_TCELL_DN	17<br>9	-<br>0.387<br>4	-<br>1.716<br>28	0.0138<br>61	0.0125<br>02
GSE8685_IL2_ACT_IL2_STARVED_VS_IL21_ACT_IL2_STARVED_CD4_TCELL_UP	16<br>2	-<br>0.417<br>3	-<br>1.668<br>76	0.0138<br>61	0.0164<br>57
GSE369_PRE_VS_POST_IL6_INJECTION_SOCS3_KO_LIVER_DN	18<br>8	-<br>0.437<br>72	-<br>1.946<br>86	0.0138<br>89	0.0036<br>36

GSE26343_UNSTIM_VS_LPS_STIM_NFAT5_KO_MACROPHAGE_UP	184	-0.37266	-1.7539	0.013889	0.009948
GSE29618_BCELL_VS_MONOCYTE_DN	187	-0.54843	-1.83489	0.013944	0.006237
GSE36009_WT_VS_NLRP10_KO_DC_LPS_STIM_UP	180	-0.28785	-1.67858	0.013944	0.01556
GSE9960_GRAM_POS_VS_GRAM_NEG_AND_POS_SEPSIS_PBMC_UP	186	-0.29906	-1.76846	0.013972	0.009183
GSE13306_RA_VS_UNTREATED_TCONV_UP	180	-0.34799	-1.54037	0.013972	0.033774
GSE22140_HEALTHY_VS_ARTHRITIC_GERMFREE_MOUSE_CD4_TCELL_UP	188	-0.45521	-1.96692	0.014	0.0034
GSE22886_NAIVE_CD8_TCELL_VS_MONOCYTE_DN	194	-0.59428	-1.78967	0.014	0.008108
GSE42021_CD24INT_VS_CD24LOW_TREG_THYMUS_DN	178	-0.46056	-1.67594	0.014	0.015776
GSE42088_UNINF_VS_LEISHMANIA_INF_DC_8H_DN	176	-0.41545	-1.47888	0.014	0.047282
GSE6269_FLU_VS_E_COLI_INF_PBMC_UP	144	-0.46178	-1.89576	0.014028	0.004486
GSE21927_SPLEEN_VS_C26GM_TUMOR_MONOCYTE_BALBC_DN	170	-0.36636	-1.57808	0.014056	0.027412
GSE24574_BCL6_LOW_TFH_VS_TCONV_CD4_TCELL_UP	179	-0.402	-1.4994	0.014056	0.042257

GSE14000_UNSTIM_VS_4H_LPS_DC_TRANSLATED_RNA_DN	176	-0.59157	-1.73277	0.014085	0.011321
GSE1432_1H_VS_6H_IFNG_MICROGLIA_UP	181	-0.32675	-1.53883	0.014085	0.0341
GSE46606_UNSTIM_VS_CD40L_IL2_IL5_1DAY_STIMULATED_IRF4HIGH_SORTED_BCELL_DN	183	-0.45656	-1.73901	0.014113	0.010847
GSE20198_UNTREATED_VS_IL12_IL18_TREATED_ACT_CD4_TCELL_DN	174	-0.29256	-1.55141	0.014113	0.031745
GSE23502_WT_VS_HDC_KO_MYELOID_DERIVED_SUPPRESSOR_CELL_COLON_TUMOR_DN	180	-0.49175	-1.52256	0.014113	0.03728
GSE27859_MACROPHAGE_VS_CD11C_INT_F480_HI_MACROPHAGE_UP	164	-0.41842	-1.83858	0.014141	0.00608
GSE26559_TCF1_KO_VS_WT_LIN_NEG_CELL_UP	179	-0.37154	-1.81726	0.014141	0.006931
GSE43260_BTLA_POS_VS_NEG_INTRATUMORAL_CD8_TCELL_DN	179	-0.29827	-1.7975	0.014141	0.007754
GSE23308_WT_VS_MINERALCORTICOID_REC_KO_MACROPHAGE_CORTICOSTERONE_TREATED_DN	179	-0.35113	-1.62553	0.014141	0.021092
GSE37534_UNTREATED_VS_PIOGLITAZONE_TREATED_CD4_TCELL_PPARG1_AND_FOXP3_TRASDUCED_UP	171	-0.3107	-1.52989	0.014141	0.035807
GSE10463_CD40L_AND_VA347_VS_CD40L_IN_DC_UP	170	-0.35866	-1.50733	0.014141	0.040499
GSE36009_WT_VS_NLRP10_KO_DC_DN	180	-0.38289	-1.895	0.01417	0.004508

GSE3982_MAST_CELL_VS_MAC_DN	174	-0.43456	-1.79095	0.01417	0.008042
GSE9037_CTRL_VS_LPS_1H_STIM_BMDM_UP	172	-0.32886	-1.58585	0.01417	0.026246
GSE32423_CTRL_VS_IL7_IL4_MEMORY_CD8_TCELL_DN	181	-0.34399	-1.52515	0.01417	0.036776
GSE18281_CORTICAL_THYMOCYTE_VS_WHOLE_CORTEX_THYMUS_UP	187	-0.47042	-1.46063	0.01417	0.05195
GSE32423_CTRL_VS_IL7_MEMORY_CD8_TCELL_DN	181	-0.38742	-1.42077	0.01417	0.064082
GSE17721_CTRL_VS_PAM3CSK4_8H_BMDC_DN	186	-0.36352	-1.58236	0.014199	0.026763
GSE18281_CORTEX_VS_MEDULLA_THYMUS_UP	161	-0.34913	-1.52305	0.014199	0.037201
GSE36078_UNTREATED_VS_AD5_T425A_HEXON_INF_IL1R_KO_MOUSE_LUNG_DC_UP	174	-0.38636	-1.44689	0.014199	0.055839
GSE17974_0H_VS_2H_IN_VITRO_ACT_CD4_TCELL_DN	170	-0.3676	-1.63629	0.014257	0.019815
GSE38681_WT_VS_LYL1_KO_LYMPHOID_PRIMED_MULTIPOTENT_PROGENITOR_DN	190	-0.51802	-1.84452	0.014286	0.005922
GSE21379_WT_VS_SAP_KO_TFH_CD4_TCELL_UP	181	-0.47341	-1.61002	0.014286	0.022947
GSE22935_WT_VS_MYD88_KO_MACROPHAGE_48H_MBOVIS_BCG_STIM_UP	184	-0.43027	-1.59293	0.014286	0.025193

GSE15659_NAIVE_VS_PTPRC_NEG_CD4_TCELL_DN	184	-0.34451	-1.41259	0.014286	0.066783
GSE11924_TH1_VS_TH17_CD4_TCELL_UP	181	-0.39237	-1.38966	0.014286	0.074851
GSE37301_RAG2_KO_VS_RAG2_AND_ETS1_KO_NK_CELL_DN	184	-0.29689	-1.523	0.014315	0.037203
GSE15659_NAIVE_CD4_TCELL_VS_NONSUPPRESSIVE_TCELL_DN	183	-0.34263	-1.46038	0.014315	0.051986
GSE17721_LPS_VS_GARDIQUIMOD_24H_BMDC_DN	178	-0.36687	-1.68323	0.014344	0.015125
GSE37301_LYMPHOID_PRIMED_MPP_VS_COMMON_LYMPHOID_PROGENITOR_DN	188	-0.32952	-1.5664	0.014344	0.029245
GSE1432_1H_VS_24H_IFNG_MICROGLIA_DN	181	-0.50944	-1.84511	0.014374	0.005912
GSE12963_UNINF_VS_ENV_AND_NEF_AND_VPR_DEFICIENT_HIV1_INF_CD4_TCELL_UP	129	-0.37527	-1.51763	0.014374	0.038309
GSE27786_CD8_TCELL_VS_ERYTHROBLAST_DN	182	-0.43125	-1.48951	0.014374	0.044524
GSE1432_CTRL_VS_IFNG_24H_MICROGLIA_DN	186	-0.57554	-1.82582	0.014403	0.006568
GSE22886_CTRL_VS_LPS_24H_DC_DN	182	-0.59566	-1.87162	0.014463	0.005175
GSE22935_UNSTIM_VS_24H_MBOVIS_BCG_STIM_MYD88_KO_MACROPHAGE_UP	184	-0.32765	-1.7653	0.014463	0.009339

GSE29949_CD8_POS_DC_SPLEEN_VS_DC_BRAIN_UP	184	-0.3486	-1.66561	0.014463	0.01671
GSE8921_UNSTIM_0H_VS_TLR1_2_STIM_MONOCYTE_24H_UP	169	-0.45979	-1.48995	0.014463	0.044408
GSE16386_IL4_VS_IL4_AND_ROSIGLITAZONE_STIM_MACROPHAGE_6H_DN	165	-0.41655	-1.40446	0.014463	0.069546
GSE6674_UNSTIM_VS_ANTI_IGM_STIM_BCELL_UP	151	-0.38385	-1.88674	0.014493	0.004701
GSE33162_HDAC3_KO_VS_HDAC3_KO_MACROPHAGE_UP	184	-0.36033	-1.60842	0.014493	0.023154
GSE8621_UNSTIM_VS_LPS_PRIMED_UNSTIM_MACROPHAGE_UP	177	-0.337	-1.56676	0.014493	0.029184
GSE15330_HSC_VS_MEGAKARYOCYTE_ERYTHROID_PROGENITOR_IKAROS_KO_UP	183	-0.36147	-1.75785	0.014553	0.009723
GSE19923_E2A_KO_VS_E2A_AND_HEB_KO_DP_THYMOCYTE_DN	188	-0.42577	-1.71253	0.014583	0.012748
GSE17974_0H_VS_4H_IN_VITRO_ACT_CD4_TCELL_DN	175	-0.39263	-1.64366	0.014614	0.018961
GSE15330_MEGAKARYOCYTE_ERYTHROID_VS_GRANULOCYTE_MONOCYTE_PROGENITOR_IKAROS_KO_DN	179	-0.37513	-1.689	0.014799	0.014593
GSE9509_10MIN_VS_30MIN_LPS_AND_IL10_STIM_IL10_KO_MACROPHAGE_DN	152	-0.40558	-1.65114	0.014925	0.01813
GSE6269_E_COLI_VS_STREP_PNEUMO_INF_PBMC_DN	151	-0.50945	-1.72953	0.014981	0.011528

GSE22886_NAIVE_CD4_TCELL_VS_MEMORY_TCELL_UP	170	-0.39239	-1.46718	0.015123	0.050264
GSE42088_2H_VS_24H_LEISHMANIA_INF_DC_UP	181	-0.51788	-1.88633	0.015238	0.00471
GSE21546_WT_VS_SAP1A_KO_ANTI_CD3_STIM_DP_THYMOCYTES_DN	182	-0.32825	-1.66275	0.015355	0.016988
GSE16385_MONOCYTE_VS_12H_IFNG_TNF_TREATED_MACROPHAGE_UP	180	-0.2924	-1.61364	0.015504	0.022512
GSE37605_C57BL6_VS_NOD_FOXP3_FUSION_GFP_TREG_UP	106	-0.48921	-1.43846	0.015564	0.058503
GSE21546_SAP1A_KO_VS_SAP1A_KO_AND_ELK1_KO_DP_THYMOCYTES_DN	177	-0.37639	-1.56571	0.015656	0.029362
GSE21033_1H_VS_12H_POLYIC_STIM_DC_DN	131	-0.35457	-1.55752	0.015873	0.03067
GSE43955_10H_VS_30H_ACT_CD4_TCELL_DN	190	-0.33612	-1.45991	0.015873	0.052105
GSE22935_24H_VS_48H_MBOVIS_BCG_STIM_MYD88_KO_MACROPHAGE_DN	186	-0.41055	-1.46933	0.015905	0.049652
GSE7831_UNSTIM_VS_INFLUENZA_STIM_PDC_1H_UP	177	-0.37394	-1.60818	0.016032	0.023164
GSE22611_MUTANT_NOD2_TRANSDUCED_VS_CTRL_HEK293T_STIMULATED_WITH_MDP_6H_DN	182	-0.34513	-1.53034	0.016032	0.035744
GSE30971_WBP7_HET_VS_KO_MACROPHAGE_DN	179	-0.53551	-1.67832	0.016064	0.015574

GSE360_L_DONOVANI_VS_M_TUBERCULOSIS_MAC_DN	189	-0.45264	-1.64384	0.016064	0.018958
GSE6259_33D1_POS_DC_VS_CD8_TCELL_UP	131	-0.44652	-1.43874	0.016064	0.058434
GSE360_CTRL_VS_M_TUBERCULOSIS_DC_DN	189	-0.51306	-1.69478	0.016129	0.014172
GSE34179_THPOK_KO_VS_WT_VA14I_NKTCELL_DN	181	-0.41396	-1.5008	0.016129	0.041922
GSE14769_UNSTIM_VS_120MIN_LPS_BMDM_DN	191	-0.51364	-1.78536	0.016162	0.008322
GSE27786_NKCELL_VS_NEUTROPHIL_UP	181	-0.26641	-1.62011	0.016194	0.021744
GSE19825_NAIVE_VS_DAY3_EFF_CD8_TCELL_UP	181	-0.50445	-1.49711	0.016194	0.042768
GSE22025_PROGESTERONE_VS_TGFB1_AND_PROGESTERONE_TREATED_CD4_TCELL_UP	181	-0.36901	-1.73731	0.01626	0.011002
GSE34156_NOD2_LIGAND_VS_TLR1_TLR2_LIGAND_6H_TREATED_MONOCYTE_DN	177	-0.56521	-1.70681	0.01626	0.013175
GSE17721_POLYIC_VS_PAM3CSK4_16H_BMDC_DN	172	-0.28002	-1.65471	0.01626	0.01781
GSE45365_HEALTHY_VS_MCMV_INFECTION_CD8A_DC_IFNAR_KO_DN	188	-0.44017	-1.81654	0.016327	0.006943
GSE32986_GMCSF_VS_GMCSF_AND_CURDLAN_LOWDOSE_STIM_DC_UP	177	-0.42666	-1.74994	0.016327	0.010164

GSE2770_TGFB_AND_IL4_ACT_VS_ACT_CD4_TCELL_2H_UP	175	-0.33242	-1.71753	0.016327	0.012424
GSE23398_WT_VS_IL2_KO_CD4_TCELL_SCURFY_MOUSE_UP	185	-0.34433	-1.60529	0.016327	0.023565
GSE37534_GW1929_VS_ROSIGLITAZONE_TREATED_CD4_TCELL_PPARG1_FOXP3_TRANSDUCED_UP	146	-0.35976	-1.58517	0.016327	0.02632
GSE3203_WT_VS_IFNAR1_KO_INFLUENZA_INFECTED_LN_BCELL_DN	172	-0.39487	-1.50775	0.016327	0.040411
GSE7548_DAY7_VS_DAY28_PCC_IMMUNIZATION_CD4_TCELL_UP	189	-0.36782	-1.55743	0.01636	0.03068
GSE29617_CTRL_VS_DAY7_TIV_FLU_VACCINE_PBMC_2008_DN	158	-0.38267	-1.70323	0.016393	0.013501
GSE45365_NK_CELL_VS_CD8A_DC_MCMV_INFECTION_DN	165	-0.40015	-1.54871	0.016393	0.032215
GSE22935_WT_VS_MYD88_KO_MACROPHAGE_48H_MBOVIS_BCG_STIM_DN	181	-0.48329	-1.80617	0.016495	0.007421
GSE42724_NAIVE_VS_MEMORY_BCELL_DN	179	-0.38668	-1.76208	0.016495	0.009487
GSE9988_ANTI_TREM1_VS_LPS_MONOCYTE_UP	177	-0.4102	-1.76472	0.016529	0.00937
GSE2706_UNSTIM_VS_8H_LPS_AND_R848_DC_DN	173	-0.53595	-1.73021	0.016598	0.011508
GSE360_CTRL_VS_M_TUBERCULOSIS_MAC_DN	187	-0.37292	-1.61243	0.016701	0.022668

GSE28726_NAIVE_CD4_TCELL_VS_NAIVE_NKTCELL_UP	191	-0.41044	-1.79811	0.016771	0.00774
GSE15330_LYMPHOID_MULTIPOTENT_VS_GRANULOCYTE_MONOCYTE_PROGENITOR_UP	179	-0.39279	-1.54488	0.016842	0.032907
GSE29614_DAY3_VS_DAY7_TIV_FLU_VACCINE_PBMC_UP	156	-0.44483	-1.3988	0.016949	0.071614
GSE2405_HEAT_KILLED_VS_LIVE_A_PHAGOCYTOPHILUM_STIM_NEUTROPHIL_9H_UP	183	-0.32987	-1.71221	0.017058	0.012767
GSE1791_CTRL_VS_NEUROMEDINU_IN_T_CELL_LINE_3H_DN	167	-0.34024	-1.56377	0.017131	0.029614
GSE19888_ADENOSINE_A3R_ACT_VS_TCELL_MEMBRANES_ACT_IN_MAST_CELL_DN	153	-0.30776	-1.57007	0.017408	0.028631
GSE10325_BCELL_VS_LUPUS_BCELL_UP	177	-0.43918	-1.49893	0.017408	0.042345
GSE33513_TCF7_KO_VS_HET_EARLY_THYMIC_PROGENITOR_DN	178	-0.29194	-1.4745	0.017544	0.048421
GSE19888_ADENOSINE_A3R_INH_PRETREAT_AND_ACT_BY_A3R_VS_A3R_INH_AND_TCELL_MEMBRANES_ACT_MAST_CELL_UP	166	-0.41416	-1.81655	0.017578	0.006946
GSE40493_BCL6_KO_VS_WT_TREG_DN	103	-0.49124	-1.41001	0.017578	0.067701
GSE27786_ERYTHROBLAST_VS_MONO_MAC_DN	182	-0.26204	-1.57731	0.017647	0.027509
GSE22313_HEALTHY_VS_SLE_MOUSE_CD4_TCELL_UP	171	-0.36611	-1.49325	0.017647	0.043617

GSE29618_MONOCYTE_VS_PDC_DAY7_FLU_VACCINE_UP	191	-0.59794	-1.81588	0.017682	0.006961
GSE38697_LIGHT_ZONE_VS_DARK_ZONE_BCELL_DN	117	-0.48896	-1.45344	0.017682	0.053988
GSE12003_4D_VS_8D_CULTURE_MIR223_KO_BM_PROGENITOR_DN	153	-0.39914	-1.63605	0.017751	0.019846
GSE29618_BCELL_VS_MDC_DAY7_FLU_VACCINE_DN	185	-0.55547	-1.86362	0.017787	0.005343
GSE14308_TH2_VS_INDUCED_TREG_DN	179	-0.2839	-1.51206	0.017822	0.039446
GSE29618_MONOCYTE_VS_MDC_UP	189	-0.5892	-1.76548	0.017857	0.00933
GSE9988_ANTI_TREM1_VS_LOW_LPS_MONOCYTE_DN	174	-0.5936	-1.75422	0.017893	0.009927
GSE4748_CTRL_VS_LPS_AND_CYANOBACTERIUM_LPSLIKE_STIM_DC_3H_DN	195	-0.35117	-1.5639	0.017893	0.029602
GSE17721_CPG_VS_GARDIQUIMOD_6H_BMDC_DN	182	-0.25707	-1.55434	0.017893	0.031195
GSE9037_CTRL_VS_LPS_1H_STIM_IRAK4_KO_BMDM_DN	180	-0.34233	-1.45049	0.017928	0.054884
GSE13485_DAY1_VS_DAY3_YF17D_VACCINE_PBMC_UP	149	-0.45716	-1.38763	0.017928	0.075569
GSE42021_CD24INT_TREG_VS_CD24INT_TCONV_THYMUS_UP	182	-0.3233	-1.79954	0.017964	0.007687

GSE18893_CTRL_VS_TNF_TREATED_TREG_2H_UP	177	-0.36667	-1.78507	0.017964	0.008333
GSE10422_WT_VS_BAFF_TRANSGENIC_LN_BCELL_UP	155	-0.31575	-1.5934	0.017964	0.025122
GSE40666_STAT1_KO_VS_STAT4_KO_CD8_TCELL_DN	145	-0.4505	-1.39896	0.017964	0.071566
GSE37532_TREG_VS_TCONV_CD4_TCELL_FROM_VISCERAL_ADIPOSE_TISSUE_UP	128	-0.46718	-1.41382	0.018036	0.066398
GSE11961_MARGINAL_ZONE_BCELL_VS_GERMINAL_CENTER_BCELL_DAY7_DN	177	-0.40126	-1.62417	0.018072	0.021281
GSE16522_ANTI_CD3CD28_STIM_VS_UNSTIM_MEMORY_CD8_TCELL_UP	185	-0.32843	-1.50253	0.018109	0.04155
GSE21360_PRIMARY_VS_TERTIARY_MEMORY_CD8_TCELL_UP	189	-0.35065	-1.80751	0.018145	0.007363
GSE12507_PDC_CELL_LINE_VS_IMMATUE_T_CELL_LINE_DN	126	-0.44242	-1.46349	0.018182	0.051122
GSE3691_CONVENTIONAL_VS_PLASMACYTOID_DC_SPLEEN_DN	185	-0.39738	-1.44801	0.018219	0.055558
GSE17721_LPS_VS_PAM3CSK4_12H_BMDC_DN	180	-0.29509	-1.57175	0.018256	0.028371
GSE32986_UNSTIM_VS_CURDLAN_LOWDOSE_STIM_DC_DN	184	-0.42909	-1.76573	0.018293	0.009327
GSE9946_LISTERIA_INF_MATURE_VS_PROSTAGLANDINE2_TREATED_MATURE_DC_DN	110	-0.47881	-1.43235	0.018293	0.060473

GSE3039_CD4_TCELL_VS_NKT_CELL_DN	177	-0.36197	-1.66052	0.01833	0.017188
GSE35685_CD34POS_CD10NEG_CD62LPOS_VS_CD34POS_CD10POS_BONE_MARROW_DN	191	-0.45194	-1.80846	0.018405	0.007328
GSE16522_ANTI_CD3CD28_STIM_VS_UNSTIM_NAIVE_CD8_TCELL_DN	182	-0.2766	-1.56422	0.01848	0.029557
GSE2770_IL12_AND_TGFB_ACT_VS_ACT_CD4_TCELL_6H_DN	186	-0.44554	-1.75667	0.018595	0.009796
GSE46025_WT_VS_FOXO1_KO_KLRG1_LOW_CD8_EFFECTOR_TCELL_DN	163	-0.34056	-1.57906	0.018595	0.027283
GSE29618_PRE_VS_DAY7_POST_TIV_FLU_VACCINE_MDC_UP	185	-0.38592	-1.52419	0.018595	0.036972
GSE14000_UNSTIM_VS_16H_LPS_DC_DN	181	-0.47841	-1.86019	0.018634	0.005444
GSE5463_CTRL_VS_DEXAMETHASONE_TREATED_THYMOCYTE_DN	183	-0.34156	-1.55969	0.018634	0.030313
GSE11961_GERMINAL_CENTER_BCELL_DAY7_VS_PLASMA_CELL_DAY7_UP	187	-0.41467	-1.5451	0.018711	0.032883
GSE17721_POLYIC_VS_CPG_8H_BMDC_DN	177	-0.28975	-1.62057	0.018868	0.02172
GSE3982_NEUTROPHIL_VS_NKCELL_DN	184	-0.29749	-1.61776	0.019027	0.021993
GSE34156_NOD2_LIGAND_VS_TLR1_TLR2_LIGAND_24H_TREATED_MONOCYTE_DN	163	-0.34508	-1.67896	0.019157	0.015529

GSE5589_WT_VS_IL6_KO_LPS_STIM_MACROPHAGE_45MIN_UP	175	-0.31623	-1.62133	0.019157	0.021623
GSE42724_MEMORY_BCELL_VS_PLASMABLAST_DN	179	-0.25672	-1.57804	0.019157	0.027412
GSE27786_CD8_TCELL_VS_NEUTROPHIL_DN	176	-0.34701	-1.52414	0.019417	0.036971
GSE16266_LPS_VS_HEATSHOCK_AND_LPS_STIM_MEF_DN	171	-0.36241	-1.72041	0.019493	0.012237
GSE13485_DAY1_VS_DAY7_YF17D_VACCINE_PBMC_UP	145	-0.47409	-1.40118	0.019569	0.070743
GSE6259_DEC205_POS_DC_VS_BCELL_UP	147	-0.46963	-1.46976	0.019685	0.04954
GSE4748_CYANOBACTERIUM_LPSLIKE_VS_LPS_AND_CYANOBACTERIUM_LPSLIKE_STIM_DC_3H_DN	160	-0.46371	-1.46361	0.019685	0.05115
GSE21670_UNTREATED_VS_TGFB_TREATED_CD4_TCELL_UP	182	-0.45806	-1.4237	0.019685	0.063101
GSE21033_3H_VS_12H_POLYIC_STIM_DC_DN	131	-0.39239	-1.4066	0.019685	0.06874
GSE1460_NAIVE_CD4_TCELL_CORD_BLOOD_VS_THYMIC_STROMAL_CELL_UP	171	-0.37312	-1.47055	0.019763	0.049363
GSE3982_NEUTROPHIL_VS_BCELL_DN	179	-0.31698	-1.74412	0.019841	0.010557
GSE2770_IL12_AND_TGFB_VS_IL4_TREATED_ACT_CD4_TCELL_2H_UP	184	-0.34306	-1.67999	0.019881	0.015433

GSE20198_UNTREATED_VS_IL12_TREATED_ACT_CD4_TCELL_UP	173	-0.27378	-1.5917	0.019881	0.025332
GSE29164_UNTREATED_VS_CD8_TCELL_TREATED_MELANOMA_DAY3_UP	182	-0.29565	-1.65268	0.01992	0.017997
GSE5589_WT_VS_IL6_KO_LPS_AND_IL10_STIM_MACROPHAGE_180MIN_DN	138	-0.38272	-1.57446	0.01992	0.02793
GSE40274_CTRL_VS_IRF4_TRANSDUCED_ACTIVATED_CD4_TCELL_DN	160	-0.34023	-1.5464	0.01992	0.03264
GSE13762_CTRL_VS_125_VITAMIND_DAY5_DC_DN	129	-0.40702	-1.39408	0.01992	0.073357
GSE9988_LPS_VS_VEHICLE_TREATED_MONOCYTE_DN	180	-0.45704	-1.96647	0.01996	0.003401
GSE22886_NAIVE_TCELL_VS_MONOCYTE_DN	192	-0.56523	-1.7695	0.02008	0.009134
GSE40273_EOS_KO_VS_WT_TREG_UP	178	-0.3256	-1.74615	0.02008	0.010439
GSE2770_TGFB_AND_IL4_VS_IL4_TREATED_ACT_CD4_TCELL_48H_UP	180	-0.37857	-1.73279	0.02008	0.011325
GSE13484_UNSTIM_VS_YF17D_VACCINE_STIM_PBMC_UP	177	-0.23519	-1.66831	0.02008	0.016492
GSE43955_TH0_VS_TGFB_IL6_TH17_ACT_CD4_TCELL_20H_UP	183	-0.35577	-1.54282	0.02008	0.033316
GSE17721_12H_VS_24H_POLYIC_BMDC_UP	178	-0.37411	-1.64259	0.020121	0.019044

GSE360_T_GONDII_VS_B_MALAYI_HIGH_DOSE_MAC_UP	185	-0.3825	-1.57011	0.020243	0.028632
GSE1791_CTRL_VS_NEUROMEDINU_IN_T_CELL_LINE_6H_UP	187	-0.42174	-1.71872	0.020284	0.012342
GSE37605_C57BL6_VS_NOD_FOXP3_IRES_GFP_TREG_UP	112	-0.46975	-1.41673	0.020284	0.065434
GSE5589_WT_VS_IL6_KO_LPS_AND_IL10_STIM_MACROPHAGE_180MIN_UP	102	-0.42762	-1.40841	0.020325	0.068162
GSE22140_GERMFREE_VS_SPF_MOUSE_CD4_TCELL_UP	192	-0.52892	-1.78189	0.020367	0.008502
GSE37533_PPARG2_FOXP3_VS_FOXP3_TRANSDUCED_CD4_TCELL_DN	185	-0.54119	-1.79049	0.020408	0.008066
GSE17580_UNINFECTED_VS_S_MANSONI_INF_TREG_DN	176	-0.37169	-1.61496	0.020408	0.022355
GSE7400_CTRL_VS_CSF3_IN_VIVO_TREATED_PBMC_DN	177	-0.38374	-1.4722	0.02045	0.049022
GSE16755_CTRL_VS_IFNA_TREATED_MAC_DN	178	-0.53861	-1.77699	0.020492	0.008742
GSE5589_LPS_VS_LPS_AND_IL10_STIM_IL10_KO_MACROPHAGE_45MIN_UP	181	-0.3242	-1.70219	0.020492	0.013551
GSE39820_CTRL_VS_TGFBETA3_IL6_CD4_TCELL_UP	179	-0.39655	-1.69592	0.020492	0.014085
GSE36476_YOUNG_VS_OLD_DONOR_MEMORY_CD4_TCELL_72H_TSST_ACT_UP	177	-0.41624	-1.39943	0.020492	0.071418

GSE37534_GW1929_VS_PIOGLITAZONE_TREATED_CD4_TCELL_PPARG1_FOXP3_TRANSDUCED_DN	179	-0.3399	-1.72076	0.020534	0.012222
GSE17721_CTRL_VS_GARDIQUIMOD_12H_BMDC_UP	183	-0.32956	-1.54177	0.020619	0.033506
GSE7509_DC_VS_MONOCYTE_UP	183	-0.4101	-1.75951	0.020661	0.009632
GSE41176_UNSTIM_VS_ANTI_IGM_STIM_TAK1_KO_BCELL_24H_DN	183	-0.49065	-1.66112	0.020661	0.017122
GSE7460_CTRL_VS_TGFB_TREATED_ACT_CD8_TCELL_UP	186	-0.39038	-1.58513	0.020661	0.026319
GSE21033_CTRL_VS_POLYIC_STIM_DC_24H_DN	138	-0.33143	-1.54159	0.020661	0.033539
GSE37336_LY6C_POS_VS_NEG_NAIVE_CD4_TCELL_UP	157	-0.43242	-1.52229	0.020661	0.037327
GSE42021_TREG_PLN_VS_CD24LO_TREG_THYMUS_DN	186	-0.4719	-1.67516	0.02079	0.015854
GSE3039_CD4_TCELL_VS_ALPHAALPHA_CD8_TCELL_DN	178	-0.38121	-1.50204	0.02079	0.04163
GSE37533_PPARG1_FOXP3_VS_FOXP3_TRANSDUCED_CD4_TCELL_UP	172	-0.29289	-1.42397	0.02079	0.063027
GSE4590_SMALL_VS_VPREB_POS_LARGE_PRE_BCELL_UP	163	-0.3663	-1.65168	0.021033	0.018088
GSE28737_BCL6_HET_VS_BCL6_KO_FOLLICULAR_BCELL_DN	186	-0.40612	-1.66168	0.021097	0.017083

GSE11924_TFH_VS_TH17_CD4_TCELL_UP	177	-0.33432	-1.70252	0.021195	0.013533
GSE29618_MONOCYTE_VS_MDC_DAY7_FLU_VACCINE_UP	192	-0.55681	-1.7493	0.021484	0.010193
GSE9650_NAIVE_VS_EFF_CD8_TCELL_DN	186	-0.50072	-1.77363	0.021526	0.008905
GSE12839_CTRL_VS_IL12_TREATED_PBMC_DN	118	-0.45808	-1.46506	0.021526	0.050785
GSE42021_TCONV_PLN_VS_CD24INT_TCONV_THYMUS_UP	180	-0.29039	-1.49214	0.021552	0.043881
GSE14413_UNSTIM_VS_IFNB_STIM_RAW264_CELLS_DN	114	-0.49218	-1.42944	0.021569	0.061378
GSE11961_MARGINAL_ZONE_BCELL_VS_MEMORY_BCELL_DAY7_DN	184	-0.46875	-1.47894	0.021611	0.04728
GSE21063_CTRL_VS_ANTI_IGM_STIM_BCELL_NFATC1_KO_16H_DN	176	-0.32722	-1.59276	0.021739	0.0252
GSE27786_CD8_TCELL_VS_NKCELL_DN	181	-0.35797	-1.61562	0.021825	0.022265
GSE2706_R848_VS_R848_AND_LPS_2H_STIM_DC_UP	160	-0.35009	-1.44782	0.021869	0.055596
GSE16385_ROSIGLITAZONE_VS_UNTREATED_IFNG_TNF_STIM_MACROPHAGE_DN	180	-0.29587	-1.6146	0.021956	0.022386
GSE27786_LIN_NEG_VS_ERYTHROBLAST_DN	179	-0.36959	-1.35848	0.021956	0.086956

GSE22935_WT_VS_MYD88_KO_MACROPHAGE_12H_MBOVIS_BCG_STIM_DN	188	-0.38825	-1.55132	0.022026	0.031751
GSE42021_TREG_PLN_VS_TREG_PRECURSORS_THYMUS_UP	176	-0.35317	-1.44224	0.022044	0.0573
GSE37534_UNTREATED_VS_ROSIGLITAZONE_TREATED_CD4_TCELL_PPARG1_AND_FOXP3_TRASDUCED_DN	184	-0.46647	-1.74133	0.022088	0.010707
GSE1432_CTRL_VS_IFNG_6H_MICROGLIA_DN	187	-0.52449	-1.82051	0.022133	0.006792
GSE19941_IL10_KO_VS_IL10_KO_AND_NFKBP50_KO_UNSTIM_MACROPHAGE_UP	191	-0.40679	-1.40953	0.022222	0.067836
GSE21360_NAIVE_VS_QUATERNARY_MEMORY_CD8_TCELL_DN	183	-0.45772	-1.77347	0.022312	0.008913
GSE15330_LYMPHOID_MULTIPOTENT_VS_PRO_BCELL_DN	180	-0.3513	-1.70336	0.022312	0.013494
GSE29618_PRE_VS_DAY7_POST_TIV_FLU_VACCINE_PDC_DN	175	-0.24511	-1.59845	0.022312	0.024487
GSE11961_FOLLICULAR_BCELL_VS_PLASMA_CELL_DAY7_UP	179	-0.37969	-1.49023	0.022312	0.044351
GSE18281_SUBCAPSULAR_VS_CENTRAL_CORTICAL_REGION_OF_THYMUS_UP	162	-0.34935	-1.63021	0.022358	0.020517
GSE22886_DAY1_VS_DAY7_MONOCYTE_IN_CULTURE_UP	187	-0.49553	-1.72744	0.022403	0.01169
GSE22601_DOUBLE_NEGATIVE_VS_CD4_SINGLE_POSITIVE_THYMOCYTE_DN	181	-0.32599	-1.68287	0.022403	0.015144

GSE28237_FOLLICULAR_VS_LATE_GC_BCELL_DN	178	-0.37141	-1.65005	0.022449	0.018249
GSE5503_LIVER_DC_VS_SPLEEN_DC_ACTIVATED_ALLOGENIC_TCELL_UP	179	-0.37741	-1.47144	0.022449	0.049133
GSE24142_ADULT_VS_FETAL_DN2_THYMOCYTE_DN	183	-0.33422	-1.39538	0.022449	0.072893
GSE43863_TH1_VS_TFH_EFFECTOR_CD4_TCELL_UP	191	-0.38493	-1.39053	0.022514	0.074599
GSE25085_FETAL_BM_VS_ADULT_BM_SP4_THYMIC_IMPLANT_UP	177	-0.45059	-1.80261	0.022541	0.007587
GSE41867_NAIVE_VS_DAY30_LCMV_ARMSTRONG_MEMORY_CD8_TCELL_UP	174	-0.31845	-1.73189	0.022541	0.01139
GSE42724_MEMORY_VS_B1_BCELL_DN	171	-0.51922	-1.69717	0.022587	0.013985
GSE26351_UNSTIM_VS_BMP_PATHWAY_STIM_HEMATOPOIETIC_PROGENITORS_UP	180	-0.34972	-1.66418	0.022587	0.016846
GSE1791_CTRL_VS_NEUROMEDINU_IN_T_CELL_LINE_6H_DN	174	-0.33581	-1.76046	0.022599	0.009592
GSE2405_0H_VS_3H_A_PHAGOCYTOPHILUM_STIM_NEUTROPHIL_UP	190	-0.50212	-1.68178	0.022634	0.015259
GSE20151_CTRL_VS_FUSOBACT_NUCLEATUM_NEUTROPHIL_DN	179	-0.30858	-1.667	0.022634	0.016599
GSE14308_TH1_VS_INDUCED_TREG_DN	172	-0.28163	-1.45708	0.02268	0.052894

GSE9006_TYPE_1_VS_TYPE_2_DIABETES_PBMC_AT_DX_DN	181	-0.38989	-1.76296	0.022727	0.009467
GSE34156_TLR1_TLR2_LIGAND_VS_NOD2_AND_TLR1_TLR2_LIGAND_24H_TREATED_MONOCYTE_UP	185	-0.57811	-1.68772	0.022727	0.014704
GSE2706_UNSTIM_VS_8H_LPS_DC_DN	170	-0.48751	-1.67195	0.022727	0.016145
GSE17721_CTRL_VS_GARDIQUIMOD_24H_BMDC_DN	181	-0.32302	-1.62705	0.022727	0.020903
GSE38696_LIGHT_ZONE_VS_DARK_ZONE_BCELL_UP	171	-0.32525	-1.75886	0.02277	0.009666
GSE7219_WT_VS_NIK_NFKB2_KO_LPS_AND_ANTI_CD40_STIM_DC_UP	182	-0.40509	-1.47126	0.022774	0.049161
GSE12845_IGD_NEG_BLOOD_VS_NAIVE_TONSIL_BCELL_UP	181	-0.36094	-1.77653	0.022869	0.008768
GSE17974_0.5H_VS_72H_IL4_AND_ANTI_IL12_ACT_CD4_TCELL_UP	174	-0.34764	-1.73371	0.022945	0.011245
GSE10240_CTRL_VS_IL22_STIM_PRIMARY_BRONCHIAL_EPITHELIAL_CELLS_DN	178	-0.26839	-1.52838	0.022965	0.03612
GSE37534_UNTREATED_VS_PIOGLITAZONE_TREATED_CD4_TCELL_PPARG1_AND_FOXP3_TRASDUCED_DN	188	-0.48834	-1.75311	0.023013	0.009983
GSE3039_ALPHAALPHA_CD8_TCELL_VS_B2_BCELL_DN	180	-0.38432	-1.65585	0.023061	0.017685
GSE17721_0.5H_VS_8H_PAM3CSK4_BMDC_DN	185	-0.28384	-1.44932	0.023061	0.055143

GSE1432_6H_VS_24H_IFNG_MICROGLIA_UP	181	-0.39152	-1.69195	0.023109	0.014352
GSE9960_HEALTHY_VS_GRAM_NEG_SEPSIS_PBMC_DN	177	-0.48392	-1.71895	0.023158	0.012331
GSE45365_NK_CELL_VS_CD8A_DC_DN	174	-0.42754	-1.42944	0.023301	0.061393
GSE13485_DAY3_VS_DAY21_YF17D_VACCINE_PBMC_UP	175	-0.37492	-1.77876	0.023438	0.008666
GSE18791_UNSTIM_VS_NEWCATSLE_VIRUS_DC_1H_UP	145	-0.45708	-1.38991	0.023622	0.074766
GSE16385_MONOCYTE_VS_12H_ROSIGLITAZONE_IFNG_TNF_TREATED_MACROPHAGE_DN	176	-0.31245	-1.61753	0.023656	0.022021
GSE4748_CTRL_VS_LPS_STIM_DC_3H_DN	179	-0.3424	-1.51425	0.023669	0.039023
GSE10325_LUPUS_BCELL_VS_LUPUS_MYELOID_DN	192	-0.57844	-1.75665	0.023715	0.009797
GSE37533_PPARG1_FOXP3_VS_FOXP3_TRANSDUCED_CD4_TCELL_DN	188	-0.54759	-1.7747	0.024048	0.008865
GSE29164_UNTREATED_VS_CD8_TCELL_AND_IL12_TREATED_MELANOMA_DAY7_UP	174	-0.31856	-1.67398	0.024096	0.015977
GSE30971_CTRL_VS_LPS_STIM_MACROPHAGE_WBP7_KO_4H_UP	169	-0.49854	-1.60701	0.024096	0.023333
GSE18791_UNSTIM_VS_NEWCATSLE_VIRUS_DC_2H_DN	145	-0.41953	-1.47015	0.024119	0.049445

GSE25846_IL10_POS_VS_NEG_CD8_TCELL_DAY7_POST_CORONAVIRUS_BRAIN_UP	174	-0.39292	-1.38942	0.024145	0.074935
GSE17721_CTRL_VS_POLYIC_6H_BMDC_UP	177	-0.27631	-1.65695	0.024242	0.01756
GSE29617_CTRL_VS_DAY7_TIV_FLU_VACCINE_PBMC_2008_UP	176	-0.44628	-1.5916	0.024242	0.025343
GSE22025_UNTREATED_VS_TGFB1_AND_PROGESTERONE_TREATED_CD4_TCELL_UP	185	-0.40771	-1.40962	0.024291	0.067815
GSE17301_CTRL_VS_48H_ACD3_ACD28_IFNA2_STIM_CD8_TCELL_UP	181	-0.36648	-1.68622	0.024341	0.014819
GSE15139_GMCSF_TREATED_VS_UNTREATED_NEUTROPHILS_UP	176	-0.28504	-1.48106	0.02444	0.046682
GSE6269_HEALTHY_VS_FLU_INF_PBMC_DN	149	-0.48295	-1.76471	0.02449	0.009367
GSE42021_TCONV_PLN_VS_CD24LO_TCONV_THYMUS_UP	184	-0.3239	-1.46086	0.02449	0.051889
GSE17721_CTRL_VS_CPG_24H_BMDC_DN	183	-0.3206	-1.5045	0.02454	0.041075
GSE22443_NAIVE_VS_ACT_AND_IL2_TREATED_CD8_TCELL_DN	188	-0.40918	-1.44968	0.02454	0.055063
GSE4748_CTRL_VS_CYANOBACTERIUM_LPSLIKE_STIM_DC_1H_DN	182	-0.34222	-1.47357	0.02459	0.048662
GSE41978_ID2_KO_VS_BIM_KO_KLRG1_LOW_EFFECTOR_CD8_TCELL_UP	188	-0.50125	-1.72268	0.024793	0.01208

GSE25088_IL4_VS_IL4_AND_ROSIGLITAZONE_STIM_STAT6_KO_MACROPHAGE_DAY10_DN	180	-0.51148	-1.8009	0.024845	0.007638
GSE19401_UNSTIM_VS_RETINOIC_ACID_STIM_FOLLICULAR_DC_UP	188	-0.44895	-1.71805	0.024845	0.012392
GSE16385_IFNG_TNF_VS_IL4_STIM_MACROPHAGE_ROSIGLITAZONE_TREATED_DN	179	-0.30885	-1.64319	0.025	0.018989
GSE25123_CTRL_VS_ROSIGLITAZONE_STIM_MACROPHAGE_UP	182	-0.29526	-1.69294	0.025243	0.014307
GSE40274_CTRL_VS_FOXP3_AND_SATB1_TRANSDUCED_ACTIVATED_CD4_TCELL_DN	138	-0.42738	-1.44983	0.025341	0.05503
GSE9316_CD4_TCELL_BALBC_VS_TH17_ENRI_CD4_TCELL_SKG_PMA_IONO_STIM_FR4NEG_UP	186	-0.36157	-1.41282	0.025341	0.06672
GSE5503_PLN_DC_VS_SPLEEN_DC_ACTIVATED_ALLOGENIC_TCELL_UP	184	-0.33303	-1.42533	0.02537	0.062606
GSE43863_NAIVE_VS_MEMORY_TH1_CD4_TCELL_D150_LCMV_DN	141	-0.41595	-1.38038	0.025424	0.078219
GSE34156_UNTREATED_VS_24H_TLR1_TLR2_LIGAND_TREATED_MONOCYTE_DN	155	-0.34073	-1.53559	0.02544	0.034696
GSE37301_GRANULOCYTE_MONOCYTE_PROGENITOR_VS_RAG2_KO_NK_CELL_UP	171	-0.2613	-1.57154	0.025532	0.028396
GSE17721_CTRL_VS_GARDIQUIMOD_6H_BMDC_DN	188	-0.31695	-1.55683	0.025532	0.030779
GSE10856_CTRL_VS_TNFRSF6B_IN_MACROPHAGE_DN	160	-0.30916	-1.42798	0.025641	0.061792

GSE26030_TH1_VS_TH17_RESTIMULATED_DAY5_POST_POLARIZATION_DN	180	-0.26523	-1.50064	0.025794	0.041965
GSE25088_IL4_VS_IL4_AND_ROSIGLITAZONE_STIM_MACROPHAGE_DAY10_UP	187	-0.39451	-1.56917	0.025896	0.028762
GSE27786_NKTCELL_VS_MONO_MAC_UP	172	-0.26873	-1.5766	0.025918	0.027635
GSE9988_ANTI_TREM1_VS_LPS_MONOCYTE_DN	172	-0.57734	-1.72661	0.026	0.011754
GSE9988_ANTI_TREM1_VS_ANTI_TREM1_AND_LPS_MONOCYTE_DN	163	-0.5523	-1.68816	0.026	0.014673
GSE39864_WT_VS_GATA3_KO_TREG_UP	191	-0.41369	-1.56536	0.026	0.029399
GSE40274_CTRL_VS_HELIOS_TRANSDUCED_ACTIVATED_CD4_TCELL_DN	165	-0.3287	-1.51367	0.026	0.039155
GSE15324_ELF4_KO_VS_WT_ACTIVATED_CD8_TCELL_UP	181	-0.29038	-1.49018	0.026052	0.044352
GSE27786_BCELL_VS_ERYTHROBLAST_DN	179	-0.40479	-1.38401	0.026052	0.076879
GSE22935_WT_VS_MYD88_KO_MACROPHAGE_12H_MBOVIS_BCG_STIM_UP	184	-0.39206	-1.5348	0.026104	0.034856
GSE6875_TCONV_VS_TREG_DN	177	-0.3866	-1.41591	0.026157	0.065707
GSE17721_POLYIC_VS_PAM3CSK4_24H_BMDC_DN	176	-0.28994	-1.53627	0.02621	0.034573

GSE42021_TCONV_PLN_VS_CD24LO_TCONV_THYMUS_DN	185	-0.36953	-1.52896	0.02621	0.036005
GSE45365_WT_VS_IFNAR_KO_CD8A_DC_DN	183	-0.43302	-1.73841	0.026263	0.010896
GSE12845_IGD_POS_BLOOD_VS_NAIVE_TONSIL_BCELL_DN	184	-0.35162	-1.69118	0.026263	0.014405
GSE4748_CTRL_VS_LPS_AND_CYANOBACTERIUM_LPSLIKE_STIM_DC_3H_UP	181	-0.40563	-1.63765	0.026263	0.019619
GSE12707_AT16L1_HYPOMORPH_VS_WT_THYMUS_UP	124	-0.37428	-1.44161	0.026263	0.057481
GSE35825_IFNA_VS_IFNG_STIM_MACROPHAGE_UP	155	-0.45758	-1.69006	0.026369	0.014509
GSE3982_MAST_CELL_VS_CENT_MEMORY_CD4_TCELL_UP	185	-0.35732	-1.65087	0.026369	0.018152
GSE39820_CTRL_VS_TGFBETA1_IL6_CD4_TCELL_DN	189	-0.32844	-1.73565	0.026423	0.011128
GSE13484_UNSTIM_VS_YF17D_VACCINE_STIM_PBMC_DN	188	-0.55396	-1.7324	0.026531	0.01135
GSE27786_LIN_NEG_VS_NEUTROPHIL_UP	165	-0.27597	-1.51889	0.026531	0.038044
GSE3982_MAST_CELL_VS_BASOPHIL_UP	173	-0.31762	-1.46687	0.026531	0.05033
GSE17721_POLYIC_VS_CPG_2H_BMDC_UP	180	-0.23113	-1.51111	0.026639	0.039656

GSE16385_MONOCYTE_VS_12H_ROSIGLITAZONE_IL4_TREATED_MACROPHAGE_UP	179	-0.31375	-1.46816	0.026694	0.049992
GSE3720_VD1_VS_VD2_GAMMADELTA_TCELL_UP	179	-0.34112	-1.38434	0.026694	0.076785
GSE29617_DAY3_VS_DAY7_TIV_FLU_VACCINE_PBMC_2008_DN	154	-0.40314	-1.57691	0.026769	0.027581
GSE15330_HSC_VS_PRO_BCELL_DN	174	-0.28325	-1.51283	0.026804	0.039288
GSE7568_CTRL_VS_3H_TGFB_TREATED_MACROPHAGES_WITH_IL4_AND_DEXAMETHASONE_DN	157	-0.31944	-1.63251	0.026871	0.02024
GSE42021_TREG_PLN_VS_TREG_PRECURSORS_THYMUS_DN	192	-0.50509	-1.74567	0.026915	0.010465
GSE18791_CTRL_VS_NEWCASTLE_VIRUS_DC_1H_DN	150	-0.38273	-1.4164	0.026975	0.065546
GSE17721_0.5H_VS_12H_CPG_BMDC_UP	177	-0.28753	-1.58741	0.027027	0.025993
GSE10500_ARTHRITIC_SYNOVIAL_FLUID_VS_HEALTHY_MACROPHAGE_UP	136	-0.33959	-1.53285	0.027083	0.03523
GSE13485_CTRL_VS_DAY7_YF17D_VACCINE_PBMC_UP	156	-0.37504	-1.42351	0.027184	0.063164
GSE29618_BCELL_VS_MONOCYTE_DAY7_FLU_VACCINE_DN	187	-0.53049	-1.76349	0.027397	0.009428
GSE9988_LOW_LPS_VS_ANTI_TREM1_AND_LPS_MONOCYTE_DN	169	-0.40063	-1.62407	0.027778	0.02129

GSE21360_SECONDARY_VS_QUATERNARY_MEMORY_CD8_TCELL_DN	156	-0.40989	-1.57334	0.027778	0.028119
GSE7568_CTRL_VS_3H_TGFB_TREATED_MACROPHAGES_WITH_IL4_AND_DEXAMETHASONE_UP	143	-0.39205	-1.33949	0.027833	0.095054
GSE17721_0.5H_VS_4H_CPG_BMDC_UP	183	-0.2639	-1.58709	0.027837	0.026051
GSE45837_WT_VS_GFI1_KO_PDC_UP	166	-0.3957	-1.46704	0.027888	0.050297
GSE4984_UNTREATED_VS_LPS_TREATED_DC_DN	158	-0.31277	-1.46349	0.027888	0.051136
GSE29614_CTRL_VS_DAY3_TIV_FLU_VACCINE_PBMC_UP	149	-0.39996	-1.38237	0.027888	0.077478
GSE13887_HEALTHY_VS_LUPUS_RESTING_CD4_TCELL_DN	113	-0.37538	-1.49752	0.027944	0.042687
GSE17721_LPS_VS_CPG_24H_BMDC_UP	180	-0.33816	-1.44359	0.027944	0.056864
GSE10325_LUPUS_CD4_TCELL_VS_LUPUS_MYELOID_DN	189	-0.59557	-1.70724	0.028112	0.013133
GSE24102_GRANULOCYSTIC_MDSC_VS_NEUTROPHIL_DN	186	-0.34338	-1.61481	0.028226	0.022368
GSE17721_CTRL_VS_PAM3CSK4_4H_BMDC_DN	181	-0.33638	-1.43872	0.028226	0.058425
GSE5099_MONOCYTE_VS_ALTERNATIVE_M2_MACROPHAGE_UP	151	-0.36485	-1.37131	0.028226	0.08156

GSE18281_SUBCAPSULAR_VS_CENTRAL_CORTICAL_REGION_OF_THYMUS_DN	187	-0.50416	-1.71071	0.02834	0.012873
GSE6259_FLT3L_INDUCED_DEC205_POS_DC_VS_CD4_TCELL_DN	125	-0.44558	-1.377	0.02834	0.079419
GSE37533_PPARG1_FOXP3_VS_PPARG2_FOXP3_TRANSDUCED_CD4_TCELL_PIOGLITAZONE_TREATED_UP	177	-0.28502	-1.35375	0.028513	0.08875
GSE14000_UNSTIM_VS_4H_LPS_DC_DN	177	-0.59488	-1.75294	0.02863	0.009993
GSE22886_NAIVE_TCELL_VS_DC_DN	192	-0.43872	-1.79547	0.028747	0.007859
GSE6269_HEALTHY_VS_STAPH_AUREUS_INF_PBMC_DN	152	-0.39181	-1.49317	0.028866	0.043629
GSE6674_CPG_VS_CPG_AND_ANTI_IGM_STIM_BCELL_DN	170	-0.34167	-1.5822	0.028926	0.02678
GSE9988_ANTI_TREM1_VS_LOW_LPS_MONOCYTE_UP	178	-0.39185	-1.67293	0.029046	0.016078
GSE23308_CTRL_VS_CORTICOSTERONE_TREATED_MACROPHAGE_DN	184	-0.36811	-1.55968	0.029046	0.030306
GSE21546_WT_VS_ELK1_KO_DP_THYMOCYTES_DN	174	-0.33824	-1.40675	0.02907	0.068702
GSE40274_IRF4_VS_FOXP3_AND_IRF4_TRANSDUCED_ACTIVATED_CD4_TCELL_DN	109	-0.43566	-1.37894	0.02907	0.07874
GSE339_CD4POS_VS_CD8POS_DC_IN_CULTURE_DN	187	-0.30269	-1.53876	0.029228	0.034104

GSE17974_2H_VS_72H_UNTREATED_IN_VITRO_CD4_TCELL_UP	171	-0.30994	-1.53733	0.029297	0.034395
GSE42021_TREG_VS_TCONV_PLN_UP	187	-0.54491	-1.72799	0.029412	0.011652
GSE14308_TH17_VS_NAIVE_CD4_TCELL_UP	176	-0.28294	-1.45927	0.029412	0.052295
GSE16450_IMMATURE_VS_MATURE_NEURON_CELL_LINE_6H_IFNA_STIM_DN	181	-0.37813	-1.38844	0.029528	0.075313
GSE22886_IL2_VS_IL15_STIM_NKCELL_DN	168	-0.29034	-1.4678	0.029661	0.050094
GSE29617_CTRL_VS_TIV_FLU_VACCINE_PBMC_2008_UP	156	-0.40418	-1.59479	0.029703	0.024975
GSE8621_UNSTIM_VS_LPS_PRIMED_AND_LPS_STIM_MACROPHAGE_UP	178	-0.34955	-1.48844	0.029915	0.044755
GSE27786_BCELL_VS_ERYTHROBLAST_UP	176	-0.23724	-1.48762	0.03012	0.044965
GSE3982_MAC_VS_BCELL_UP	188	-0.39881	-1.56681	0.030426	0.029194
GSE17974_CTRL_VS_ACT_IL4_AND_ANTI_IL12_1H_CD4_TCELL_UP	163	-0.32198	-1.4186	0.030593	0.064784
GSE19198_1H_VS_6H_IL21_TREATED_TCELL_DN	186	-0.46926	-1.70878	0.030612	0.013025
GSE37301_MULTIPOTENT_PROGENITOR_VS_COMMON_LYMPHOID_PROGENITOR_DN	186	-0.30234	-1.60438	0.030702	0.023696

GSE30971_CTRL_VS_LPS_STIM_MACROPHAGE_WBP7_KO_2H_UP	168	-0.51468	-1.58005	0.030738	0.02712
GSE9601_NFKB_INHIBITOR_VS_PI3K_INHIBITOR_TREATED_HCMV_INF_MONOCYTE_DN	187	-0.47566	-1.59463	0.030992	0.02498
GSE1925_CTRL_VS_IFNG_PRIMED_MACROPHAGE_24H_IFNG_STIM_DN	187	-0.34952	-1.55844	0.031056	0.030501
GSE37301_LYMPHOID_PRIMED_MPP_VS_PRO_BCELL_UP	182	-0.26945	-1.38237	0.031056	0.07746
GSE33162_HDAC3_KO_VS_HDAC3_KO_MACROPHAGE_DN	180	-0.38679	-1.65475	0.03112	0.017809
GSE11864_CSF1_VS_CSF1_IFNG_PAM3CYS_IN_MAC_UP	173	-0.24659	-1.42723	0.031558	0.061996
GSE18281_CORTICAL_VS_MEDULLARY_THYMOCYTE_UP	193	-0.47778	-1.67026	0.031683	0.016329
GSE22886_NAIVE_CD4_TCELL_VS_MONOCYTE_DN	195	-0.55752	-1.74285	0.032	0.010618
GSE10147_IL3_VS_IL3_AND_CPG_STIM_PDC_DN	152	-0.26894	-1.4717	0.03212	0.049131
GSE2706_R848_VS_R848_AND_LPS_2H_STIM_DC_DN	168	-0.49265	-1.54997	0.032129	0.031974
GSE12003_MIR223_KO_VS_WT_BM_PROGENITOR_4D_CULTURE_DN	188	-0.41187	-1.41367	0.032258	0.066435
GSE22886_NAIVE_CD4_TCELL_VS_NKCELL_DN	189	-0.33555	-1.69601	0.032323	0.01408

GSE20198_IL12_VS_IL12_IL18_TREATED_ACT_CD4_TCELL_UP	180	-0.31258	-1.62509	0.032389	0.021149
GSE13522_CTRL_VS_T_CRUZI_Y_STRAIN_INF_SKIN_129_MOUSE_UP	123	-0.42114	-1.40425	0.032454	0.069614
GSE7548_NAIVE_VS_DAY7_PCC_IMMUNIZATION_CD4_TCELL_DN	189	-0.48647	-1.74228	0.03252	0.010633
GSE46606_UNSTIM_VS_CD40L_IL2_IL5_3DAY_STIMULATED_IRF4_KO_BCELL_DN	164	-0.36459	-1.64713	0.03252	0.018596
GSE24972_MARGINAL_ZONE_BCELL_VS_FOLLICULAR_BCELL_IRF8_KO_UP	182	-0.36065	-1.50921	0.03252	0.040079
GSE19923_WT_VS_E2A_KO_DP_THYMOCYTE_UP	187	-0.3957	-1.47475	0.03252	0.048382
GSE7459_UNTREATED_VS_IL6_TREATED_ACT_CD4_TCELL_DN	128	-0.41293	-1.38638	0.032587	0.075986
GSE6259_33D1_POS_DC_VS_CD4_TCELL_UP	164	-0.39458	-1.53463	0.032653	0.034873
GSE27241_WT_CTRL_VS_DIGOXIN_TREATED_RORGT_KO_CD4_TCELL_IN_TH17_POLARIZING_CONDITIONS_DN	161	-0.31836	-1.63064	0.03272	0.020469
GSE24634_IL4_VS_CTRL_TREATED_NAIVE_CD4_TCELL_DAY7_DN	175	-0.40343	-1.6247	0.03272	0.021205
GSE2585_CD80_HIGH_VS_LOW_MTEC_UP	183	-0.42819	-1.35541	0.032755	0.088139
GSE7509_FCGRIIB_VS_TNFA_IL1B_IL6_PGE_STIM_DC_DN	134	-0.45842	-1.55533	0.032882	0.031033

GSE41176_WT_VS_TAK1_KO_ANTI_IGM_STIM_BCELL_3H_UP	176	-0.37872	-1.42332	0.032882	0.063216
GSE6269_HEALTHY_VS_STAPH_PNEUMO_INF_PBMC_DN	161	-0.47756	-1.6309	0.032946	0.020441
GSE11864_UNTREATED_VS_CSF1_PAM3CYS_IN_MAC_DN	169	-0.26479	-1.44759	0.03301	0.055659
GSE37416_0H_VS_3H_F_TULARENSIS_LVS_NEUTROPHIL_DN	182	-0.33273	-1.66024	0.033126	0.017217
GSE15330_WT_VS_IKAROS_KO_MEGAKARYOCYTE_ERYTHROID_PROGENITOR_UP	179	-0.249	-1.52159	0.033126	0.037481
GSE43955_1H_VS_20H_ACT_CD4_TCELL_DN	186	-0.3751	-1.50661	0.033333	0.040623
GSE7596_AKT_TRANSD_VS_CTRL_CD4_TCONV_WITH_TGFB_UP	151	-0.33947	-1.58281	0.033399	0.026707
GSE41867_MEMORY_VS_EXHAUSTED_CD8_TCELL_DAY30_LCMV_DN	183	-0.27455	-1.52361	0.033399	0.037087
GSE360_HIGH_DOSE_B_MALAYI_VS_M_TUBERCULOSIS_MAC_DN	185	-0.46206	-1.60051	0.033403	0.02424
GSE29618_PRE_VS_DAY7_FLU_VACCINE_BCELL_DN	177	-0.27532	-1.39149	0.033465	0.074242
GSE26343_UNSTIM_VS_LPS_STIM_MACROPHAGE_UP	183	-0.40117	-1.64324	0.033613	0.018989
GSE37416_CTRL_VS_12H_F_TULARENSIS_LVS_NEUTROPHIL_DN	165	-0.33888	-1.69158	0.033827	0.014382

GSE13306_LAMINA_PROPRIA_VS_SPLEEN_TREG_DN	180	-0.37029	-1.36106	0.034068	0.085846
GSE3400_UNTREATED_VS_IFNB_TREATED_MEF_UP	166	-0.3351	-1.64968	0.034274	0.018288
GSE21546_WT_VS_ELK1_KO_ANTI_CD3_STIM_DP_THYMOCYTES_UP	175	-0.34852	-1.41707	0.034274	0.065328
GSE13485_CTRL_VS_DAY7_YF17D_VACCINE_PBMC_DN	192	-0.58283	-1.66606	0.034483	0.016684
GSE21546_WT_VS_SAP1A_KO_DP_THYMOCYTES_UP	178	-0.5393	-1.75853	0.034553	0.009688
GSE15735_2H_VS_12H_HDAC_INHIBITOR_TREATED_CD4_TCELL_DN	189	-0.40202	-1.57811	0.034553	0.027415
GSE42724_NAIVE_BCELL_VS_PLASMABLAST_UP	175	-0.55997	-1.69638	0.034623	0.014058
GSE10325_CD4_TCELL_VS_LUPUS_CD4_TCELL_DN	185	-0.51083	-1.63256	0.034694	0.020239
GSE7831_1H_VS_4H_INFLUENZA_STIM_PDC_UP	182	-0.32499	-1.53211	0.034694	0.035363
GSE33425_CD161_INT_VS_NEG_CD8_TCELL_UP	190	-0.36836	-1.76566	0.034765	0.009327
GSE37301_COMMON_LYMPHOID_PROGENITOR_VS_CD4_TCELL_UP	177	-0.29717	-1.46512	0.034783	0.050782
GSE37533_UNTREATED_VS_PIOGLIZATONE_TREATED_CD4_TCELL_PPARG1_AND_FOXP3_TRASDUCED_UP	188	-0.51855	-1.71885	0.034908	0.012339

GSE21927_SPLEEN_C57BL6_VS_4T1_TUMOR_BALBC_MONOCYTES_DN	187	-0.43724	-1.71651	0.034908	0.012487
GSE22886_NAIVE_BCELL_VS_NEUTROPHIL_DN	183	-0.53933	-1.72426	0.035088	0.011948
GSE21927_HEALTHY_VS_TUMOROUS_BALBC_MOUSE_MONOCYTE_UP	162	-0.30404	-1.39916	0.035088	0.071511
GSE6259_FLT3L_INDUCED_DEC205_POS_DC_VS_BCELL_DN	147	-0.42349	-1.57515	0.035124	0.027823
GSE360_HIGH_VS_LOW_DOSE_B_MALAYI_MAC_UP	183	-0.31326	-1.50901	0.035124	0.040117
GSE39152_CD103_NEG_VS_POS_MEMORY_CD8_TCELL_DN	190	-0.30616	-1.47558	0.035124	0.048177
GSE45365_HEALTHY_VS_MCMV_INFECTION_BCELL_IFNAR_KO_DN	169	-0.34531	-1.45391	0.035124	0.053876
GSE18791_UNSTIM_VS_NEWCATSLE_VIRUS_DC_1H_DN	162	-0.36342	-1.44503	0.035491	0.056428
GSE13547_WT_VS_ZFX_KO_BCELL_ANTI_IGM_STIM_12H_UP	135	-0.45051	-1.37201	0.035573	0.081314
GSE22611_UNSTIM_VS_2H_MDP_STIM_NOD2_TRANSDUCED_HEK293T_CELL_DN	180	-0.32527	-1.36578	0.035639	0.08388
GSE43955_TH0_VS_TGFB_IL6_TH17_ACT_CD4_TCELL_1H_DN	187	-0.36421	-1.42696	0.035644	0.062082
GSE28737_BCL6_HET_VS_BCL6_KO_MARGINAL_ZONE_BCELL_DN	182	-0.31644	-1.5446	0.035714	0.032958

GSE28737_WT_VS_BCL6_KO_MARGINAL_ZONE_BCELL_DN	174	-0.33419	-1.56967	0.035785	0.028684
GSE14769_UNSTIM_VS_60MIN_LPS_BMDM_UP	173	-0.29797	-1.55624	0.035928	0.030884
GSE37301_LYMPHOID_PRIMED_MPP_VS_GRAN_MONO_PROGENITOR_DN	174	-0.23865	-1.52123	0.035941	0.037557
GSE3982_MAST_CELL_VS_EFF_MEMORY_CD4_TCELL_UP	180	-0.29524	-1.46861	0.036017	0.049856
GSE11367_CTRL_VS_IL17_TREATED_SMOOTH_MUSCLE_CELL_UP	123	-0.39446	-1.3799	0.036122	0.078383
GSE32901_TH1_VS_TH17_ENRICHED_CD4_TCELL_DN	146	-0.30811	-1.45303	0.036145	0.054101
GSE1432_1H_VS_24H_IFNG_MICROGLIA_UP	193	-0.41162	-1.6484	0.036217	0.01842
GSE14699_DELETIONAL_TOLERANCE_VS_ACTIVATED_CD8_TCELL_UP	170	-0.43619	-1.61812	0.036217	0.021952
GSE1925_CTRL_VS_IFNG_PRIMED_MACROPHAGE_DN	187	-0.33546	-1.55272	0.036325	0.031498
GSE27786_NKTCELL_VS_NEUTROPHIL_UP	175	-0.23488	-1.51361	0.036437	0.039158
GSE27786_BCELL_VS_MONO_MAC_DN	181	-0.33663	-1.33654	0.036437	0.09637
GSE17721_LPS_VS_GARDIQUIMOD_16H_BMDC_DN	182	-0.34416	-1.63358	0.036481	0.02012

GSE46606_IRF4HIGH_VS_WT_CD40L_IL2_IL5_DAY3_STIMULATED_BCELL_UP	185	-0.29477	-1.52554	0.036585	0.036706
GSE12845_PRE_GC_VS_DARKZONE_GC_TONSIL_BCELL_DN	172	-0.24769	-1.51585	0.03666	0.038677
GSE19772_CTRL_VS_HCMV_INF_MONOCYTES_AND_PI3K_INHIBITION_UP	163	-0.31423	-1.42804	0.036735	0.06178
GSE42021_CD24HI_VS_CD24LOW_TREG_THYMUS_UP	183	-0.29173	-1.41401	0.036735	0.066373
GSE36078_UNTREATED_VS_AD5_INF_MOUSE_LUNG_DC_DN	174	-0.39648	-1.52756	0.037037	0.036302
GSE18791_CTRL_VS_NEWCASTLE_VIRUS_DC_4H_DN	165	-0.49324	-1.62089	0.037109	0.02168
GSE17721_LPS_VS_GARDIQUIMOD_6H_BMDC_DN	180	-0.2767	-1.5366	0.03719	0.034528
GSE6259_FLT3L_INDUCED_33D1_POS_DC_VS_BCELL_DN	181	-0.32252	-1.57157	0.037255	0.028398
GSE15659_CD45RA_NEG_CD4_TCELL_VS_RESTING_TREG_DN	178	-0.32362	-1.36013	0.037255	0.086222
GSE41176_WT_VS_TAK1_KO_ANTI_IGM_STIM_BCELL_3H_DN	187	-0.39133	-1.69207	0.037267	0.014347
GSE2128_C57BL6_VS_NOD_CD4CD8_DP_THYMOCYTE_UP	181	-0.37575	-1.60817	0.037328	0.023159
GSE22140_HEALTHY_VS_ARTHRITIC_GERMFREE_MOUSE_CD4_TCELL_DN	189	-0.48601	-1.69296	0.037344	0.014316

GSE29618_PRE_VS_DAY7_FLU_VACCINE_BCELL_UP	181	-0.39982	-1.4377	0.037402	0.058717
GSE2770_IL12_AND_TGFB_ACT_VS_ACT_CD4_TCELL_2H_UP	179	-0.32109	-1.60835	0.038	0.023152
GSE14908_ATOPIC_VS_NONATOPIC_PATIENT_HDM_STIM_CD4_TCELL_DN	180	-0.31427	-1.42738	0.038055	0.061955
GSE3720_UNSTIM_VS_PMA_STIM_VD1_GAMMADELTA_TCELL_DN	120	-0.40915	-1.3881	0.038076	0.075422
GSE17721_0.5H_VS_12H_PAM3CSK4_BMDC_DN	178	-0.31657	-1.64898	0.038136	0.018338
GSE21063_WT_VS_NFATC1_KO_8H_ANTI_IGM_STIM_BCELL_DN	178	-0.27595	-1.59362	0.038462	0.0251
GSE25123_ROSIGLITAZONE_VS_IL4_AND_ROSIGLITAZONE_STIM_MACROPHAGE_DAY10_DN	111	-0.37094	-1.37722	0.038462	0.079353
GSE21360_SECONDARY_VS_QUATERNARY_MEMORY_CD8_TCELL_UP	177	-0.5366	-1.62067	0.03854	0.021711
GSE19401_UNSTIM_VS_PAM2CSK4_STIM_FOLLICULAR_DC_UP	181	-0.30908	-1.39006	0.038618	0.07473
GSE6259_FLT3L_INDUCED_33D1_POS_DC_VS_CD4_TCELL_DN	155	-0.39077	-1.57761	0.038697	0.027475
GSE1432_1H_VS_6H_IFNG_MICROGLIA_DN	181	-0.44338	-1.68137	0.038855	0.015274
GSE37533_PPARG1_FOXP3_VS_PPARG2_FOXP3_TRANSDUCED_CD4_TCELL_PIOGLITAZONE_TREATED_DN	188	-0.52219	-1.7839	0.038934	0.008399

GSE17721_PAM3CSK4_VS_CPG_1H_BMDC_UP	185	-0.32021	-1.43199	0.039063	0.060554
GSE14769_40MIN_VS_360MIN_LPS_BMDM_DN	187	-0.31207	-1.48411	0.039216	0.045845
GSE43955_TH0_VS_TGFB_IL6_TH17_ACT_CD4_TCELL_1H_UP	184	-0.37931	-1.50678	0.039256	0.040609
GSE17721_CTRL_VS_POLYIC_8H_BMDC_UP	174	-0.29857	-1.55614	0.039337	0.030886
GSE21678_WT_VS_FOXO1_FOXO3_KO_TREG_DN	170	-0.313	-1.46546	0.039666	0.050712
GSE33425_CD8_ALPHAALPHA_VS_ALPHABETA_CD161_HIGH_TCELL_DN	182	-0.44937	-1.56292	0.039749	0.029762
GSE43956_WT_VS_SGK1_KO_TH17_DIFFERENTIATED_CD4_TCELL_UP	185	-0.37541	-1.35716	0.039749	0.087395
GSE14000_TRANSLATED_RNA_VS_MRNA_16H_LPS_DC_UP	147	-0.35273	-1.34369	0.039848	0.093168
GSE19888_NO_PRETREAT_VS_ADENOSINE_A3R_INHIBITOR_PRETREATED_MAST_CELL_TCELL_MEMBRANES_ACT_UP	182	-0.26681	-1.39334	0.039916	0.073558
GSE8921_UNSTIM_VS_TLR1_2_STIM_MONOCYTE_24H_DN	185	-0.32239	-1.52599	0.040161	0.036624
GSE360_L_MAJOR_VS_M_TUBERCULOSIS_DC_UP	182	-0.29403	-1.37647	0.040161	0.079598
GSE13485_DAY3_VS_DAY7_YF17D_VACCINE_PBMC_DN	173	-0.5328	-1.57948	0.040241	0.027215

GSE9006_HEALTHY_VS_TYPE_1_DIABETES_PBMC_4MONTH_POST_DX_DN	175	-0.31024	-1.40759	0.040323	0.068455
GSE40274_FOXP3_VS_FOXP3_AND_XBP1_TRANSDUCED_ACTIVATED_CD4_TCELL_UP	141	-0.39124	-1.34872	0.040323	0.090988
GSE16522_ANTI_CD3CD28_STIM_VS_UNSTIM_NAIVE_CD8_TCELL_UP	170	-0.30493	-1.34148	0.040404	0.094152
GSE2405_0H_VS_6H_A_PHAGOCYTOPHILUM_STIM_NEUTROPHIL_UP	182	-0.25941	-1.52533	0.040462	0.03674
GSE3982_DC_VS_MAC_DN	185	-0.43718	-1.46053	0.040568	0.051967
GSE1925_CTRL_VS_24H_IFNG_STIM_IFNG_PRIMED_MACROPHAGE_DN	190	-0.35186	-1.54782	0.04065	0.032382
GSE26669_CTRL_VS_COSTIM_BLOCK_MLR_CD4_TCELL_UP	187	-0.34677	-1.5277	0.04065	0.03628
GSE21380_NON_TFH_VS_GERMINAL_CENTER_TFH_CD4_TCELL_DN	180	-0.35193	-1.65786	0.041068	0.017468
GSE8835_HEALTHY_VS_CLL_CD8_TCELL_UP	180	-0.26931	-1.47163	0.041068	0.049113
GSE26030_TH1_VS_TH17_RESTIMULATED_DAY15_POST_POLARIZATION_UP	177	-0.45839	-1.59806	0.041152	0.024543
GSE44649_NAIVE_VS_ACTIVATED_CD8_TCELL_MIR155_KO_UP	182	-0.26995	-1.52105	0.041152	0.037592
GSE20754_WT_VS_TCF1_KO_MEMORY_CD8_TCELL_UP	181	-0.29391	-1.56884	0.041408	0.028813

GSE6674_ANTI_IGM_VS_CPG_STIM_BCELL_DN	181	-0.33506	-1.56094	0.041408	0.030079
GSE17721_CTRL_VS_CPG_0.5H_BMDC_UP	191	-0.28579	-1.54003	0.04175	0.033845
GSE22886_NAIVE_CD8_TCELL_VS_DC_DN	187	-0.42024	-1.83973	0.042	0.006067
GSE9601_UNTREATED_VS_NFKB_INHIBITOR_TREATED_HCMV_INF_MONOCYTE_DN	167	-0.35375	-1.61258	0.042	0.022647
GSE21927_SPLEEN_VS_TUMOR_MONOCYTE_BALBC_DN	172	-0.32112	-1.40695	0.042065	0.068649
GSE14000_4H_VS_16H_LPS_DC_DN	161	-0.26145	-1.59196	0.042254	0.025292
GSE37416_0H_VS_48H_F_TULARENSIS_LVS_NEUTROPHIL_DN	177	-0.2691	-1.52084	0.042308	0.037622
GSE18791_CTRL_VS_NEWCASTLE_VIRUS_DC_6H_DN	182	-0.50928	-1.66758	0.04277	0.016555
GSE37301_HEMATOPOIETIC_STEM_CELL_VS_CD4_TCELL_UP	162	-0.54365	-1.56825	0.042857	0.028911
GSE2405_S_AUREUS_VS_A_PHAGOCYTOPHILUM_NEUTROPHIL_DN	183	-0.36256	-1.58078	0.043053	0.02702
GSE3920_IFNA_VS_IFNG_TREATED_ENDOTHELIAL_CELL_UP	157	-0.38338	-1.6157	0.043071	0.022262
GSE14769_UNSTIM_VS_80MIN_LPS_BMDM_DN	184	-0.48274	-1.63188	0.043121	0.020326

GSE33292_DN3_THYMOCYTE_VS_TCELL_LYMPHOMA_FROM_TCF1_KO_DN	186	-0.35866	-1.38688	0.043299	0.075848
GSE32986_CURDLAN_LOWDOSE_VS_GMCSF_AND_CURDLAN_LOWDOSE_STIM_DC_DN	178	-0.33661	-1.65089	0.043478	0.018156
GSE27786_LSK_VS_BCELL_UP	171	-0.26736	-1.39656	0.043659	0.072468
GSE35543_IN_VITRO_ITREG_VS_CONVERTED_EX_ITREG_DN	178	-0.34912	-1.39553	0.043738	0.072859
GSE10273_LOW_IL7_VS_HIGH_IL7_AND_IRF4_IN_IRF4_8_NULL_PRE_BCELL_UP	182	-0.35458	-1.41397	0.04375	0.066356
GSE12963_UNINF_VS_ENV_AND_NEF_DEFICIENT_HIV1_INF_CD4_TCELL_UP	136	-0.37673	-1.4192	0.043912	0.064607
GSE40666_STAT1_KO_VS_STAT4_KO_CD8_TCELL_UP	170	-0.25625	-1.51706	0.043933	0.03843
GSE20715_0H_VS_6H_OZONE_TLR4_KO_LUNG_DN	186	-0.34945	-1.49565	0.044	0.04307
GSE1432_CTRL_VS_IFNG_6H_MICROGLIA_UP	188	-0.26548	-1.46242	0.044088	0.051425
GSE360_L_DONOVANI_VS_B_MALAYI_HIGH_DOSE_MAC_UP	181	-0.40817	-1.56678	0.044118	0.029191
GSE7218_UNSTIM_VS_ANTIGEN_STIM_THROUGH_IGM_BCELL_UP	115	-0.41443	-1.37153	0.044355	0.081482
GSE21379_WT_VS_SAP_KO_CD4_TCELL_UP	182	-0.32301	-1.38388	0.044444	0.076891

GSE2770_IL4_ACT_VS_ACT_CD4_TCELL_6H_DN	177	-0.32803	-1.70358	0.044534	0.013479
GSE36826_WT_VS_IL1R_KO_SKIN_DN	179	-0.41161	-1.65579	0.044534	0.017684
GSE8921_UNSTIM_VS_TLR1_2_STIM_MONOCYTE_6H_DN	173	-0.39551	-1.43391	0.044534	0.059953
GSE9316_IL6_KO_VS_IFNG_KO_INVIVO_EXPANDED_CD4_TCELL_DN	185	-0.52122	-1.62041	0.044586	0.021727
GSE17721_LPS_VS_POLYIC_2H_BMDC_DN	183	-0.23789	-1.4951	0.044625	0.043184
GSE21927_EL4_VS_MCA203_TUMOR_MONOCYTES_UP	151	-0.44807	-1.46353	0.044807	0.051157
GSE34156_UNTREATED_VS_6H_NOD2_AND_TLR1_TLR2_LIGAND_TREATED_MONOCYTE_UP	153	-0.34879	-1.36197	0.045175	0.085468
GSE21360_NAIVE_VS_QUATERNARY_MEMORY_CD8_TCELL_UP	188	-0.43765	-1.69442	0.045267	0.01419
GSE37605_C57BL6_VS_NOD_FOXP3_FUSION_GFP_TCONV_DN	172	-0.24752	-1.5109	0.045267	0.039693
GSE28726_NAIVE_VS_ACTIVATED_NKTCELL_DN	186	-0.35572	-1.65422	0.045643	0.017864
GSE42021_TREG_PLN_VS_CD24INT_TREG_THYMUS_DN	189	-0.52245	-1.70381	0.045833	0.013464
GSE41867_DAY6_VS_DAY15_LCMV_CLONE13_EFFECTOR_CD8_TCELL_DN	151	-0.40273	-1.29055	0.046243	0.119103

GSE2706_UNSTIM_VS_2H_LPS_AND_R848_DC_UP	163	-0.31124	-1.52895	0.046371	0.035996
GSE21546_WT_VS_SAP1A_KO_DP_THYMOCYTES_DN	158	-0.31771	-1.32011	0.046371	0.104162
GSE46606_UNSTIM_VS_CD40L_IL2_IL5_1DAY_STIMULATED_IRF4_KO_BCELL_DN	171	-0.33455	-1.42328	0.046843	0.063198
GSE11924_TFH_VS_TH2_CD4_TCELL_UP	175	-0.33023	-1.64143	0.046875	0.019152
GSE16385_MONOCYTE_VS_12H_ROSIGLITAZONE_IFNG_TNF_TREATED_MACROPHAGE_UP	177	-0.28102	-1.41154	0.046875	0.067149
GSE3039_CD4_TCELL_VS_ALPHABETA_CD8_TCELL_DN	184	-0.31405	-1.44709	0.04721	0.055797
GSE2770_IL12_AND_TGFB_VS_IL4_TREATED_ACT_CD4_TCELL_2H_DN	168	-0.29384	-1.5978	0.047337	0.024575
GSE4590_PRE_BCELL_VS_LARGE_PRE_BCELL_DN	151	-0.29037	-1.45447	0.047337	0.053713
GSE5589_WT_VS_IL6_KO_LPS_AND_IL6_STIM_MACROPHAGE_45MIN_DN	185	-0.38237	-1.50242	0.047619	0.041541
GSE19401_RETINOIC_ACID_VS_RETINOIC_ACID_AND_PAM2CSK4_STIM_FOLLICULAR_DC_DN	182	-0.27514	-1.38145	0.047809	0.077793
GSE45382_UNTREATED_VS_TGFB_TREATED_MACROPHAGES_DN	170	-0.39391	-1.50894	0.047817	0.040121
GSE27786_NKTCELL_VS_MONO_MAC_DN	188	-0.32928	-1.38646	0.047917	0.075992

GSE17721_LPS_VS_CPG_16H_BMDC_DN	187	-0.31744	-1.40806	0.048017	0.068297
GSE8515_IL1_VS_IL6_4H_STIM_MAC_DN	182	-0.42041	-1.3919	0.048077	0.074125
GSE42088_UNINF_VS_LEISHMANIA_INF_DC_4H_UP	180	-0.35876	-1.59839	0.04829	0.02449
GSE2405_HEAT_KILLED_VS_LIVE_A_PHAGOCYTOPHILUM_STIM_NEUTROPHIL_9H_DN	186	-0.37391	-1.42668	0.04829	0.062167
GSE360_LOW_DOSE_B_MALAYI_VS_M_TUBERCULOSIS_DC_DN	191	-0.43827	-1.59478	0.048387	0.024971
GSE14308_TH1_VS_NATURAL_TREG_UP	177	-0.26817	-1.35053	0.048485	0.090226
GSE22611_NOD2_TRANSD_VS_CTRL_TRANSD_HEK293_MDP_STIM_2H_DN	125	-0.47766	-1.35802	0.048638	0.087117
GSE42021_CD24HI_VS_CD24INT_TREG_THYMUS_DN	185	-0.521	-1.7084	0.04888	0.013044
GSE19923_WT_VS_HEB_AND_E2A_KO_DP_THYMOCYTE_DN	185	-0.41668	-1.60808	0.04888	0.023165
GSE22611_MUTANT_NOD2_VS_CTRL_TRANSDUCED_HEK293T_CELL_DN	168	-0.37117	-1.3673	0.049485	0.083224

Table S9. Correlations of risk score with 22 kinds of TICs.

TICs	R	P-value
B cells memory	-0.3723	6.00E-18
Mast cells resting	-0.3137	6.35E-13
Macrophages M1	-0.2452	2.61E-08
Dendritic cells resting	-0.2424	3.80E-08
Macrophages M0	0.2168	9.42E-07
NK cells activated	0.2068	2.96E-06
Dendritic cells activated	0.2057	3.36E-06
Neutrophils	0.1672	0.00017
T cells gamma delta	-0.148	0.00088
Mast cells activated	0.1408	0.00156
T cells CD8	-0.1255	0.00488
B cells naïve	0.1226	0.00595
Plasma cells	0.1211	0.0066
NK cells resting	0.1052	0.01843
T cells regulatory (Tregs)	0.0919	0.0396
T cells CD4 memory activated	-0.0772	0.0841
T cells CD4 naive	0.0658	0.14084
Macrophages M2	-0.0507	0.25638
Eosinophils	0.043	0.33615
T cells follicular helper	-0.0375	0.40196
Monocytes	-0.0333	0.45609
T cells CD4 memory resting	-0.0068	0.87892
